# Engineered Enzymes Enable Selective *N*‐Alkylation of Pyrazoles With Simple Haloalkanes

**DOI:** 10.1002/anie.202014239

**Published:** 2021-01-21

**Authors:** Ludwig L. Bengel, Benjamin Aberle, Alexander‐N. Egler‐Kemmerer, Samuel Kienzle, Bernhard Hauer, Stephan C. Hammer

**Affiliations:** ^1^ Faculty of Chemistry, Organic Chemistry and Biocatalysis Bielefeld University Universitätsstraße 25 33615 Bielefeld Germany; ^2^ Department of Technical Biochemistry Institute of Biochemistry and Technical Biochemistry University of Stuttgart Allmandring 31 70569 Stuttgart Germany

**Keywords:** alkylation, biocatalysis, enzyme cofactors, enzyme engineering, pyrazoles

## Abstract

Selective alkylation of pyrazoles could solve a challenge in chemistry and streamline synthesis of important molecules. Here we report catalyst‐controlled pyrazole alkylation by a cyclic two‐enzyme cascade. In this enzymatic system, a promiscuous enzyme uses haloalkanes as precursors to generate non‐natural analogs of the common cosubstrate S‐adenosyl‐l‐methionine. A second engineered enzyme transfers the alkyl group in highly selective C−N bond formations to the pyrazole substrate. The cosubstrate is recycled and only used in catalytic amounts. Key is a computational enzyme‐library design tool that converted a promiscuous methyltransferase into a small enzyme family of pyrazole‐alkylating enzymes in one round of mutagenesis and screening. With this enzymatic system, pyrazole alkylation (methylation, ethylation, propylation) was achieved with unprecedented regioselectivity (>99 %), regiodivergence, and in a first example on preparative scale.

## Graphical abstract

Biological alkylation is highly selective, yet it depends on complex leaving groups. Now, promiscuous and engineered enzymes achieve selective enzymatic alkylation using simple haloalkanes.

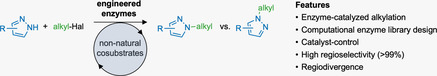

## Introduction

**Figure 1:**
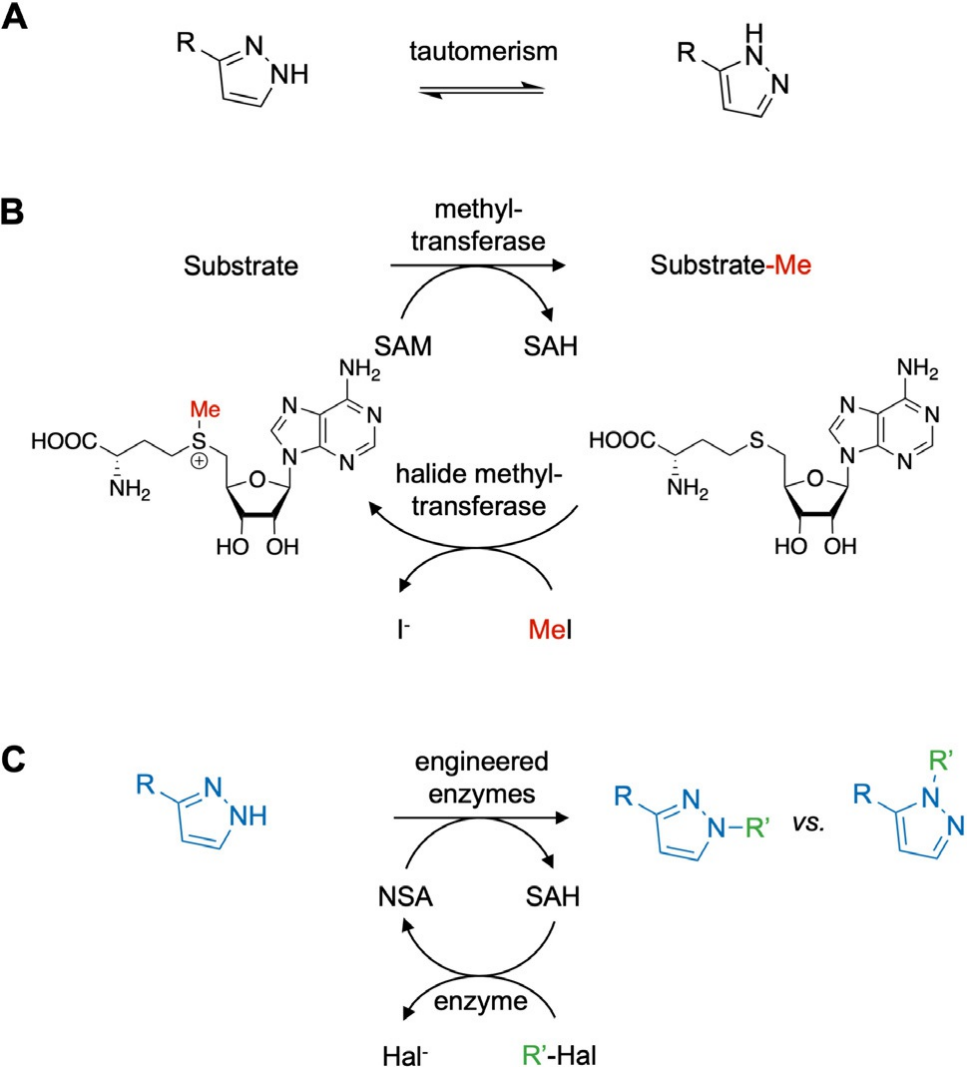
(**A**) Pyrazole structures can be described as two different tautomers. Tautomerization results in *N*‐atoms with very similar reactivity that are challenging to discriminate in chemical reactions. (**B**) SAM‐dependent methyltransferases can transfer methyl groups with very high selectivity to complex substrates. In this reactions *S*‐adenosyl‐l‐methionine (SAM) serves as cosubstrate and methyl donor. *S*‐adenosyl‐l‐homocysteine (SAH) is generated as byproduct and can be used by halide methyltransferases to regenerate SAM using iodomethane as methyl source.^[12]^ (**C**) The research outlined here uses promiscuous halide methyltransferases to generate and recycle non‐natural SAM analogs (NSA) from simple haloalkanes. In combination with engineered alkyl transferases highly selective pyrazole alkylation is envisioned.

The regioselective alkylation and functionalization of molecules bearing multiple heteroatoms with similar properties is a particular challenge in synthesis.^[1]^ This is especially true for *N*‐heterocyclic compounds such as pyrazoles, triazoles or pyridones where tautomerization leads to heteroatoms with comparable reactivity. Alkylation of such *N*‐heterocyclic compounds is under substrate control and produces product mixtures that are often laborious to separate (Figure 1 A and Figure S1).^[2]^ Selective alkylation depends on protecting group strategies^[3]^ and a general catalyst‐controlled alkylation does not exist.[^4^, ^5^] Given the significant number of C−N alkylations conducted in medicinal chemistry[^6^, ^7^] and based on the importance of such *N*‐alkylated heterocycles in biologically active agents (Figure S2),^[8]^ it becomes clear why selective alkylation methods are on top of many wish lists for organic chemistry.[^4^, ^5^, ^9^] New methods that form such *C*−*N* bonds selectively could shorten current synthetic routes and even make new molecules accessible that were previously difficult to prepare.

In nature, selective heteroatom alkylation is carried out by various well‐known enzymes with unprecedented precision and activity.[^10^, ^11^] Prenyl‐, methyl‐ and glycosyltransferases assemble *C*‐heteroatom bonds with high selectivity on a large set of substrates including small molecules, peptides, proteins and DNA. Enzymes achieve excellent selectivities in such challenging reactions because their active sites offer a high level of molecular recognition to accurately pre‐organize substrates prior to catalysis. A broad application of alkyl transferases in synthesis is, however, hampered by the complexity of the used alkyl donors.

Enzyme‐catalyzed selective heteroatom alkylation largely depends on alkyl donors with leaving groups that are synthetically difficult to access, unstable and/or lead to low atom economy in the overall reaction (Figure S3). If we were able to combine enzymatic alkylation with the utilization of simple and readily available alkyl donors, solutions for many challenging reactions could be envisioned.

Recently, significant progress towards enzymatic alkylation chemistry using “off the shelf” alkylation reagents has been made, in particular exploiting SAM‐dependent methyltransferases (MTs). MTs use the cosubstrates *S*‐adenosyl‐l‐methionine (SAM) and a natural carboxy‐analog^[17]^ of SAM in highly selective methylation and carboxymethylation reactions. Multiple studies have further demonstrated that MTs are functional with non‐natural SAM analogs (NSA) to selectively transfer a huge variety of alkyl groups.[^11^, ^18^] However, the synthesis of NSA is currently not particularly straight forward (Figure S4). Several elegant strategies for enzymatic synthesis of NSA have been developed.[^19^, ^20^, ^21^] These approaches still depend on the laboratory synthesis of methionine analogs as NSA precursors that limits their application.[^11^, ^18^, ^22^] Liao and Seebeck have recently shown that SAM can be enzymatically synthesized and recycled (Figure 1 B).^[12]^ In this cyclic enzyme cascade, a halide methyltransferase charges *S*‐adenosyl‐l‐homocysteine (SAH) with iodomethane to generate SAM. A second MT uses SAM to methylate a substrate with high selectivity. At the same time SAH is regenerated and is therefore only required in catalytic concentrations to shuttle the methyl group between both enzymes. Currently, there is very little evidence that this system can be used beyond methylation to synthesize and recycle NSA directly from simple haloalkanes (Figure S4).

Here we report selective *N*‐alkylation of pyrazoles by engineered enzymes in a cyclic two enzyme cascade (Figure 1 C). The method was implemented based on two major findings. First, an identified promiscuous halide methyltransferase can synthesize and recycle NSA using haloalkanes as sole stoichiometric reagents. Second, a computational enzyme library design tool^[13]^ successfully transformed a second promiscuous MT into a pyrazole‐alkylating enzyme family in one round of mutagenesis and screening. The enzyme system catalyzed desired *C*−*N* bond formations with unprecedented selectivities using simple haloalkanes as starting materials.

## Results and Discussion

### Enzyme Engineering to Generate Pyrazole Methyltransferases

**Figure 2:**
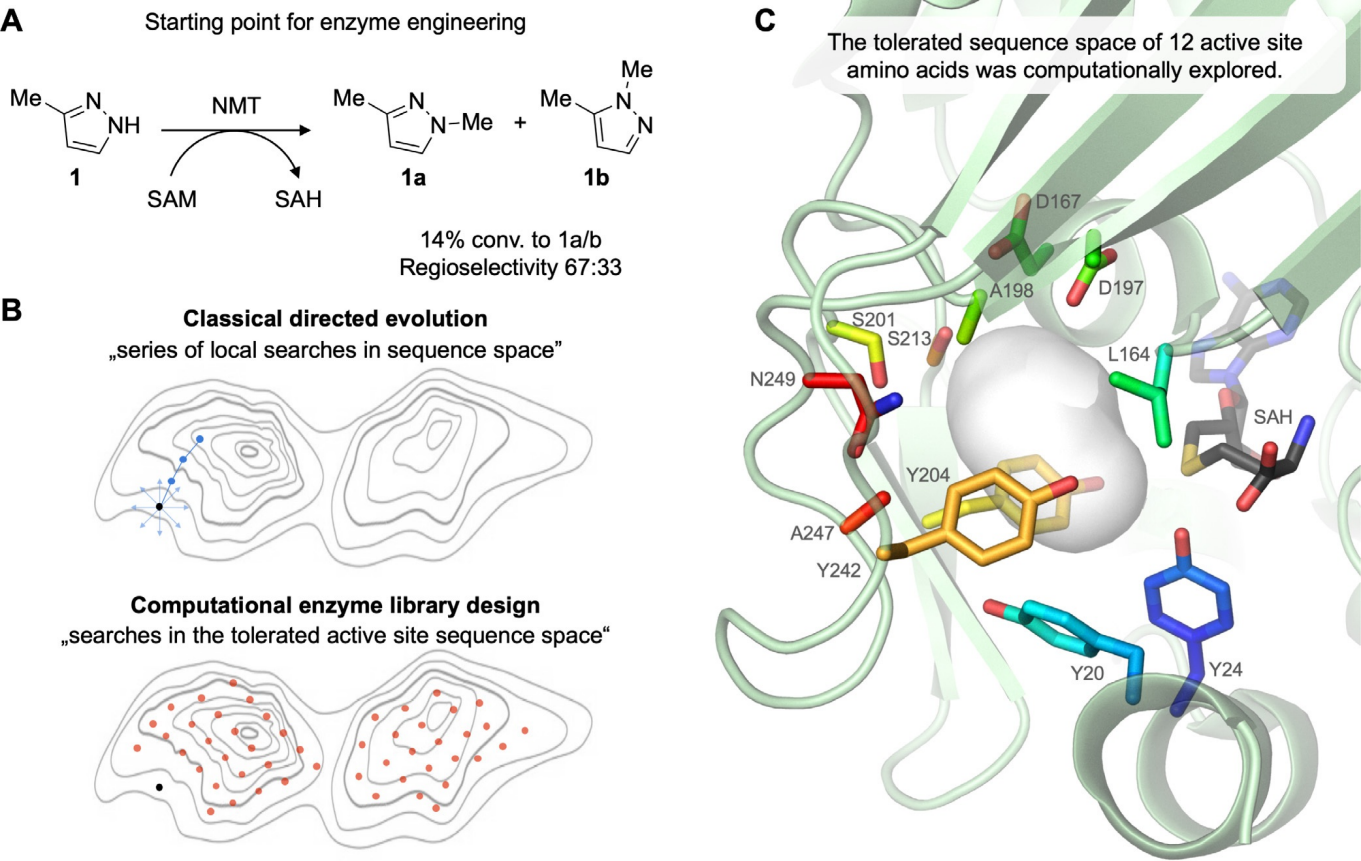
Engineering of NMT. (**A**) Promiscuous activity of *homo sapiens* nicotinamide *N*‐methyltransferase (NMT) in the methylation of 3‐methylpyrazole (**1**). (**B**) Enzyme engineering of NMT was performed using FuncLib,^[13]^ a computational enzyme library design tool that calculates the sequence space of tolerated active site mutations. An advantage to classical directed evolution approaches such as iterative saturation mutagenesis^[14]^ is that FuncLib allows large and meaningful steps in sequence space by introducing multiple active site mutations simultaneously. Figure 2 B is inspired by recently published, excellent reviews.[^15^, ^16^] (**C**) Structure of NMT (pdb 2iip): Active site amino acids that have been randomized using FuncLib are shown as colored sticks. Cocrystallized SAH is shown as black sticks and the shape of the substrate binding pocket is illustrated as grey shadow.

Because pyrazoles are extremely rare in nature,^[23]^ pyrazole MTs are currently not known. As a result, we aimed to access this enzyme function by engineering a promiscuous natural MT. In order to identify a suitable starting point for enzyme engineering, we examined several MTs for promiscuity towards pyrazole methylation using SAM as cosubstrate. In particular, we studied the *homo sapiens* nicotinamide *N*‐methyltransferase,^[24]^ phenylethanolamine *N*‐methyltransferase^[25]^ and histamine *N*‐methyltransferase[^26^, ^27^] due to their reported substrate flexibility. Out of these 3 enzymes, only the *homo sapiens* nicotinamide *N*‐methyltransferase (NMT) revealed promiscuous activity with 3‐methylpyrazole (**1**) as substrate (Figure 2 A). The respective products were formed with 14 % as mixture of regioisomers (67:33 for **1 a**:**1 b**). This illustrates that wild‐type NMT can hardly discriminate between the two *N*‐atoms of **1**.

We sought to improve the enzyme's promiscuous activity by using computational enzyme library design.^[16]^ While traditional directed evolution protocols optimize promiscuous activity for one substrate towards one chosen product,^[28]^ we aimed to generate a whole panel of transferases with activity for a broad range of substrates potentially accessing both product isomers with high selectivity. To this end, we used a recently developed algorithm by Sarel Fleishman and co‐workers called FuncLib.^[13]^ This protein design method can efficiently calculate the theoretically tolerated sequence space in enzyme active sites.^[28]^ Phylogenetic and atomistic calculations are combined to analyze the stability of multiple active site mutations *in silico*. We have chosen 12 active site amino acids of NMT to computationally explore the stability of multiple mutations in the active site. These 12 amino acids build the substrate binding pocket but do not directly interact with the SAM cosubstrate (Figure 2 C and Figure S5). The FuncLib algorithm was applied as described in the supporting information.

In short, the phylogenetic analysis and mutational stability calculations at these 12 amino acid positions reduced the active site sequence space to 473.294 possible variants. This pool was then further analyzed in silico by calculating the stability of the designs bearing 3–5 active site mutations. The most stable designs were clustered based on their sequence diversity (cluster criterion: amino acid difference ≥3). We decided to test the top 50 sequences, all bearing 3–5 active site mutations and differ in at least 3 residues (see Figure S6). In this way, large and potentially meaningful steps in the active site sequence space can be experimentally investigated without depending on ultrahigh‐throughput screening methods^[29]^ (Figure 2 B).

**Figure 3:**
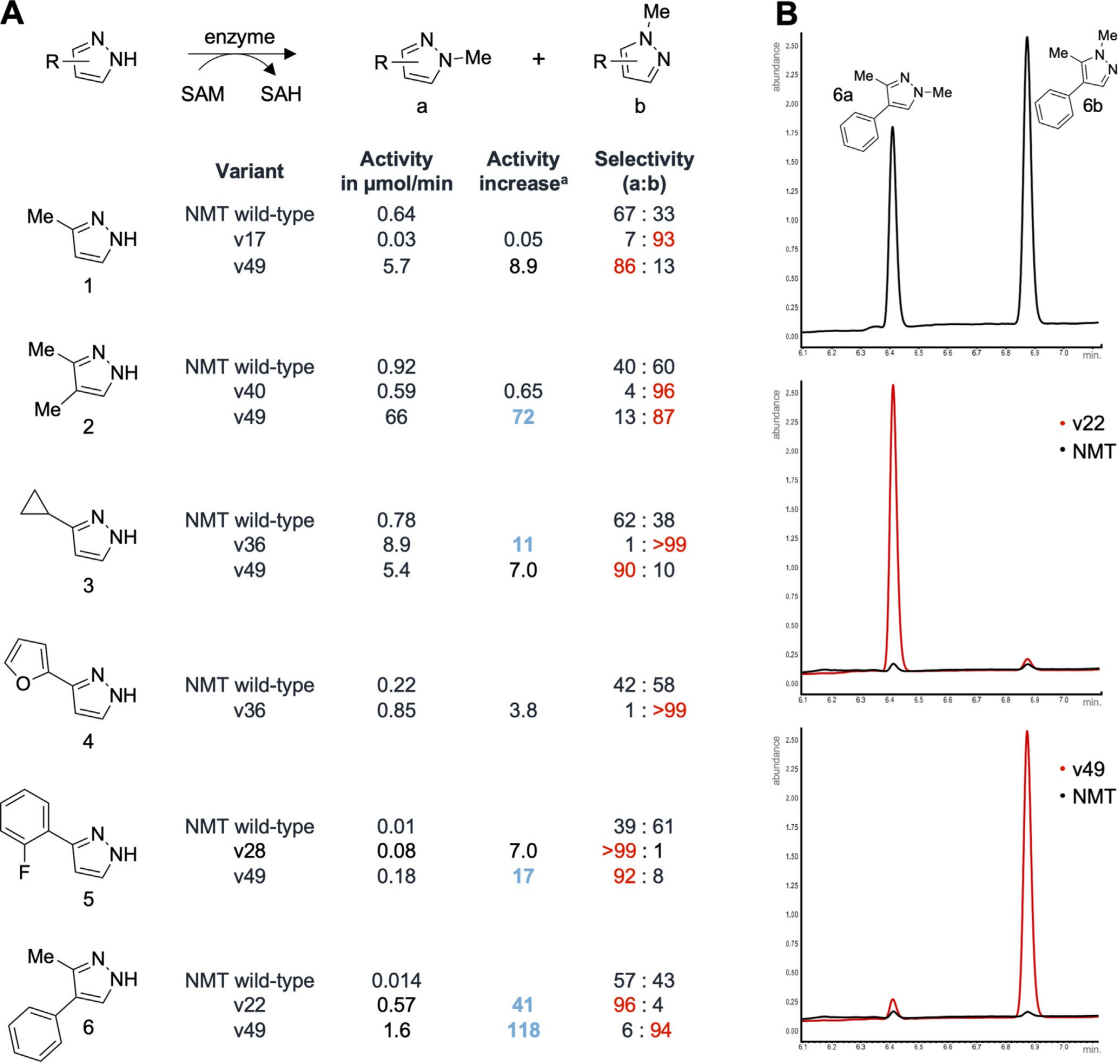
(**A**) Selected variants were characterized by analyzing the initial rates using 2 mM pyrazole, 2 mM SAM and 50 μM enzyme. All enzymatic reactions were performed in triplicates. Please note that all chemical methylations using reagents as well as reactions with NMT wild‐type generate product mixtures (see Figure S1 and S15–S20). ^a)^ Activity increase is calculated by dividing the initial rate of the variant by the initial rate of NMT wild‐type. (**B**) As an example, GC chromatograms for the conversion of **6** with selected variants (red) are shown and compared to wildtype (black) as well as product standards (top). These examples demonstrate the efficiency of FuncLib to convert a promiscuous starting point (NMT wild‐type) into highly selective variants with significant increase in activity in just one round of mutagenesis and screening. GC retention time 6a: 6.4 minutes. GC retention time 6b: 6.9 minutes (see Figure S20).

The enzyme panel (v1–v50) was bought as synthetic DNA, produced in *E. coli* and tested for methyl transfer activity using a set of six structurally different pyrazoles as substrates (Figure 3). Screening of the library was performed using cell lysate in deep‐well plate format with SAM as cosubstrate (Figure S7). The performance of the variants was analyzed using gas chromatography. Even though the designs introduced 3–5 active site mutations simultaneously, >90 % of the designs were active and converted at least one pyrazole substrate (Figure S8–S13). Depending on the substrate, 10–30 % of the variants showed increased activity and/or selectivity compared to the NMT wild‐type enzyme. Next, we purified a selected set of the best enzymes (Figure S14) to characterize the selectivities and activities in detail. The selected pyrazole MTs perform *C*−*N* bond formation with very high catalytic control, in some cases with regioselectivities of >99 % (Figure 3 and Figure S15–S20). Catalyst control could even be demonstrated for **1** and 3,4‐dimethylpyrazole (**2**) as substrates for which the pyrazole tautomers differ only in the relative position of a small methyl group (see Figure 1 A). High regioselectivities and in part regiodivergence were also found for 3‐substituted pyrazoles bearing cyclopropyl (**3**) and (hetero)‐aromatic groups (**4** and **5**) as well as for pyrazoles with bulky substituents on the 4‐position (**6**) (see Figure 3 and Figure S15–S20 for details). Please note that NMT wild‐type (Figure 3) as well as chemical methylations with standard reagents (Figure S1) generate product mixtures for all the tested pyrazole substrates.[^3^, ^30^] There are currently no catalysts known that control pyrazole methylation or alkylation with such precision.[^4^, ^5^]

In addition to regiocontrol, we studied the activities of the selected pyrazole MTs using stoichiometric amounts of SAM as cosubstrate. Because MTs in general and the *homo sapiens* nicotinamide *N*‐methyltransferase in particular are known to suffer from inhibition of the SAH by‐product (Figure 1 B),[^31^, ^32^] we decided to validate activity based on initial rate determination using uniform substrate and enzyme concentrations (Figure 3). Activity comparison by initial rate determination is most likely not biased by potential SAH inhibition. In general, many designs showed activity enhancements by a factor >10 compared to the NMT wild‐type (Figure 3 and Figure S21–S26). In some cases, very high increase in activity up to a factor of 72 and 118 was found. This is remarkable because the variants performed with excellent selectivities at the same time. Such activity enhancements are outstanding and usually not observed with conventional mutagenesis strategies.^[33]^ Iterative saturation mutagenesis or random mutagenesis often show activity enhancements by a factor of 2–4 per round of evolution.[^28^, ^34^, ^35^, ^36^, ^37^]

Six different enzyme variants (v17, v22, v28, v36, v40, v49) with increased activity and selectivity for the six pyrazole substrates were studied in detail (Figure 3). In these six designs, 7 out of the 12 target active site amino acids were mutated (Figure S6). Three positions (D167, S201, S213) were altered in all of the six variants and two positions (A198, N249) were mutated in ≥50 %. Interestingly, these five amino acids are spatially all located next to each other (see top of the active site in Figure 2 C) and might generate the binding pocket for the different substituents on the pyrazole ring. These mutable amino acids are part of the loops connecting the 4–5, 5–6 and 6–7 β‐strands of the methyltransferase Rossmann‐fold.^[38]^ The introduced beneficial mutations at these five hot‐spot positions are very diverse (D167C/H, A198L/T, S201A/C/I/Q, S213A/C/H/M and N249C/A) and general beneficial mutations for pyrazole methylation cannot be identified. As recently reported, the multiple mutations introduced by FuncLib often show non‐additive behavior.^[13]^ Such epistasis explains large steps in activity and selectivity, but makes it difficult to generalize the mode of action. We believe that the single set of mutations in each top variant enables selective binding of the pyrazole substrate in a reactive conformation. This is in agreement with literature highlighting that methyltransferases bind their substrates in a near‐attack conformation to achieve efficient catalysis.^[39]^

### Identification of Promiscuous Halide Methyltransferases

After establishing the selective methylation of pyrazoles, we aimed to expand the system towards selective alkylation. The remarkable SAM cofactor recycling system by Liao and Seebeck^[12]^ (Figure 1 B) has the potential to enzymatically synthesize and recycle non‐natural SAM analogs (NSA) from readily available haloalkanes as alkyl donors.^[40]^ While this system is currently limited to methylation,[^12^, ^41^, ^42^] we envisioned to expand its application towards general alkyl transfer. Key in this endeavor is to find promiscuous halide methyl transferases (HMT) that accept different haloalkanes apart from iodomethane as substrates. This would enable synthesis and recycling of NSAs directly from SAH (see Figure 1 B). As a step in this direction, we report here a fungal HMT from *Aspergillus clavatus* that accepts haloethane, ‐propane and ‐butane as substrates for NSA synthesis. In the following, we will refer to this enzyme as NSA‐synthase. The NSA‐synthase was identified by studying the substrate scope of six literature‐known HMTs, including enzymes from bacterial, fungal und plant origin (Figure S28). The enzymes were bought as synthetic DNA and produced in the SAH‐nucleosidase deficient *E. coli* strain JW0155.^[12]^ Substrate scope screening was performed with cell lysates using SAH and several haloalkanes as substrates (haloalkanes include iodomethane, iodoethane, bromoethane, 1‐iodopropane, 1‐iodobutane and 1‐bromobutane, see Figure S28). While most of the tested HMTs were very restrictive accepting only iodomethane as substrate, the identified NSA‐synthase converted haloethanes, ‐propanes and ‐butanes. Notably, next to iodoalkanes the corresponding bromoalkanes are also substrates of the NSA‐synthase. Control experiments without enzyme, SAH or alkyl donor confirmed that the NSA formation is enzyme‐catalyzed (Figure S29). As a general trend, we observed that the activity of enzymatic NSA synthesis decreased with increasing alkyl chain length of the haloalkanes (Figure S30). While the ethyl analog was synthesized with >80 % product formation, the propyl analog was generated with 12 % and the butyl analog with 3.6 %. After confirming the formation of the NSA by mass spectrometry (Figure S31), we aimed to combine the NSA‐synthase with the engineered pyrazole MTs to study enzymatic pyrazole alkylation.

### Enzymatic Pyrazole Alkylation Using Simple Haloalkanes

**Figure 4:**
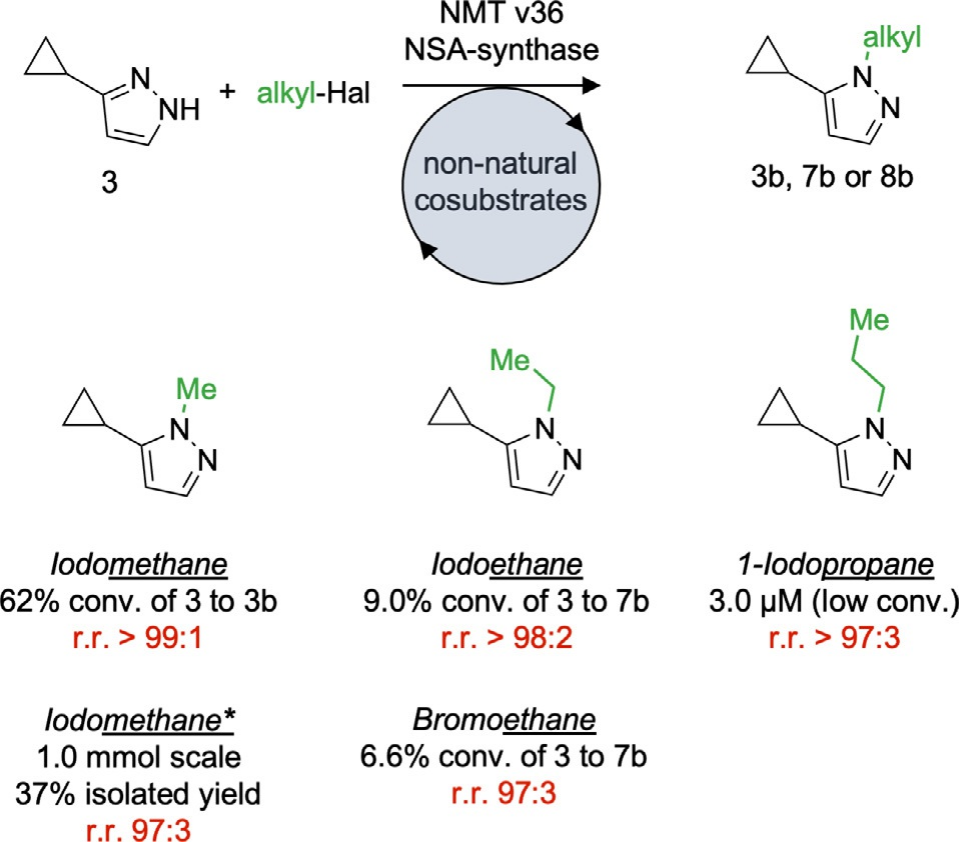
Enzymatic selective alkylation of pyrazoles using haloalkanes. v36 and NSA‐synthase (50 μM each) was used as catalyst. The reactions were performed with 2 mM pyrazole (3) as substrate, 10 equiv. of haloalkane and 30 °C (iodo‐, bromoethane and iodopropane) as well as 37 °C (iodomethane). The reaction time was 30 h using iodomethane, 40 h in the iodomethane preparative scale reaction, 72 h in using haloethanes and 50 h with 1‐iodopropane. *This reaction was performed on preparative scale (1.0 mmol), please see SI for details. Regioselectivity is described as regioisomeric ratio (r.r.).

Since NSA formation was most efficient for ethyl and propyl analogs (Figure S30), we focussed our proof‐of‐concept studies on the enzymatic ethylation and propylation using enzyme v36 and 3‐cyclopropylpyrazol (**3**) as substrate (Figure 3). This enzyme‐substrate combination was selected based on the high activity and selectivity in pyrazole methylation (Figure S27). To set up the cyclic enzyme cascade consisting of v36 and the NSA‐synthase, we used iodomethane as alkyl donor and studied the productivity of the system using different SAH concentrations. Surprisingly, our first experiments revealed that residual SAH bound to the purified enzymes (v36 and NSA‐synthase) was sufficient for efficient methylation of **3**. Further addition of SAH (0.05 equiv. with respect to the substrate) did not change the productivity of the system (Figure S32). Next, we examined the formation of the methylated product as a function of iodomethane excess. Highest product formation of **3 b** (62 %, Figure 4) was achieved using 10 equivalents of iodomethane. However, equimolar concentrations of iodomethane with respect to the substrate **3** reduced the product formation only slightly to 47 % (Figure S33). In all cases **3** was methylated with very high regioselectivity (>99 %) to generate 1‐methyl‐5‐cyclopropylpyrazole (**3 b**). To demonstrate that enzymatic pyrazole alkylation can be performed on a preparative scale (1.0 mmol), the methylated pyrazole **3 b** was synthesized using iodomethane as alkyl donor (Figure S34). The product was generated with 37 % isolated yield and with very high regioselectivity (97 %). 55 % of pure starting material was recovered after the reaction.

Finally, we aimed to prove general enzymatic alkyl transfer using iodoethane, bromoethane and 1‐iodopropane as starting material. Even though v36 was originally selected for methyl transfer, **3** was ethylated and propylated using the enzyme system (Figure 4). The enzymatic alkylation of **3** generated 1‐ethyl‐5‐cyclopropylpyrazole (**7 b**) and 1‐propyl‐5‐cyclopropylpyrazole (**8 b**) with very high regioselectivites of >98 % and >97 % (Figure S35 and S36). The moderate to low yields achieved in these initial enzyme cascade reactions might originate from several causes and require detailed analysis. In a simple scenario, v36 is efficient in methylation but inefficient in ethylation and propylation of **3**. It is believed that SAM‐dependent methyltransferases function by binding the substrate and cosubstrate in a reactive conformation, pre‐aligning the orbitals for efficient and selective catalysis.^[39]^ A variant chosen for methylation may therefore be unproductive in binding pyrazole **3** in the same reactive conformation in the presence of a larger ethyl or propyl group. Alternatively, the methyltransferase could suffer from product inhibition. Finally, the alkylation of SAH can produce NSA in two epimeric forms as such sulfonium species have a stereochemically active lone pair. It is currently not clear which epimer is generated by the NSA synthase, and it is also unknown whether the engineered methyltransferases such as v36 accept both epimers as cosubstrate. These questions will be addressed in future studies.

## Conclusion

Our studies highlight a potentially generalizable approach to obtain catalyst‐control in challenging alkylation reactions. In particular, we show that selective alkylation of pyrazoles can be achieved using simple haloalkanes, engineered enzymes and an expanded cosubstrate pool. An important step was the identification of a promiscuous halide methyltransferase (named NSA‐synthase) that enzymatically generates and recycles non‐natural analogs of SAM. An additional central part was the engineering of a second promiscuous transferase that is active on pyrazoles as substrates. We believe that this approach together with important data published elsewhere[^12^, ^42^] will open up interesting new avenues for enzyme engineering to enable selective *C*‐heteroatom bond formation in various complex molecules. Many of these reactions are highly desired and have so far eluded catalytic‐selective synthesis.[^4^, ^5^, ^9^]

The research outlined here also supports that computational enzyme library design tools such as FuncLib^[13]^ are very effective. Over 90 % of the designs are active, 10–30 % of the variants were more active and/or selective than the wildtype enzyme and we identified highly selective variants with significant activity improvements at the same time. The strength of FuncLib is to successfully introduce multiple active site mutations simultaneously. This enables large steps in sequence space accompanied with drastic increase in activity and selectivity. FuncLib transformed a promiscuous starting point (NMT wild‐type) into a small pyrazole alkylating enzyme family in just one round of mutagenesis and screening. This artificial enzyme family accepts structurally diverse pyrazoles as substrates, is highly regioselective and in many cases regiodivergent (Figure 3). It will be interesting to see whether computational enzyme library design tools can be combined with machine learning^[15]^ or performed iteratively to further speed up directed enzyme evolution.^[43]^

## Conflict of interest

The authors declare no conflict of interest.

## Supporting information

As a service to our authors and readers, this journal provides supporting information supplied by the authors. Such materials are peer reviewed and may be re‐organized for online delivery, but are not copy‐edited or typeset. Technical support issues arising from supporting information (other than missing files) should be addressed to the authors.

Supplementary
