# Supplementary material for: Engineered Enzymes Enable Selective N‐Alkylation of Pyrazoles With Simple Haloalkanes
Source: Angew Chem Int Ed Engl. 2021 Jan 21;60(10):5554–60. doi: 10.1002/anie.202014239 (PMC7986378; doi:10.1002/anie.202014239)
Supplement: Supplementary file 1 — Supplementary [file ANIE-60-5554-s001.pdf]

Angewandte Chemie
Eine Zeitschrift der Gesellschaft Deutscher Chemiker

Supporting Information

Engineered Enzymes Enable Selective *N*-Alkylation of Pyrazoles With Simple Haloalkanes

*Ludwig L. Bengel, Benjamin Aberle, Alexander-N. Egler-Kemmerer, Samuel Kienzle, Bernhard Hauer, and Stephan C. Hammer**

anie_202014239_sm_miscellaneous_information.pdf

This PDF file includes:

I. Materials and methods 2

II. General procedures 7

III. Supporting figures 12

IV. Calibration curves for initial rate measurements, enzymatic ethylation and propylation ... 51

V. Chemical synthesis of product standards 55

VI. NMR spectra of product standards 59

VII. NMR analysis of enzymatic scale-up methylation of 3-cycloproyplpyrazole (3) 80

VIII. HR-ESI-MS analysis of product standards. 82

IX. Enzyme sequences 97

X. References 109

Supporting Information

I. Materials and methods

(**A**) All chemicals and solvents, as not described otherwise, were purchased from commercial suppliers (Sigma Aldrich, Abcr, Alfa Aesar, Apollo Scientific, Fisher Scientific, Enamine, Fluorochem) and used without additional purification. *S*-adenosyl-L-methionine disulfat tosylat was purchased from Abcr (cat-#: AB436584), *S*-adenosyl-L-homocysteine was purchased from Sigma-Aldrich (cat-#: A9384), Lysozyme was purchased from Roth® (cat-#: 8259.3) and DNaseI from Gold Biotechnology® (cat-#: D-300-5).

(**B**) All NMR spectra were recorded on a Bruker 500 MHz instrument in $CDCl_3$ and referenced to tetramethylsilane. Data for ^{1}H NMR are reported in the conventional form: chemical shift (δ ppm), multiplicity (d = doublet, dd = doublet of doublets, t = triplet, q = quartet, m = multiplet), coupling constant (Hz), integration. Data for ^{13}C NMR are reported in terms of chemical shift. The correct assignment of the chemical shifts was confirmed by two-dimensional correlation measurements, including heteronuclear single quantum coherence (HSQC), heteronuclear multiple bond coherence (HMBC) and homonuclear correlation spectroscopy (COSY).

(**C**) High resolution mass spectra were measured using electrospray ionization in positive mode on a microTOFQ Bruker Daltonics, LC-coupled with an Agilent 1200 series. Scan started from 50 m/z and ended at 1500 m/z. Capillary was set at 4500 V, End Plate Offset at -500 V, Collision Cell RF at 180.0 Vpp, Nubilizer at 0.4 bar, Dry heater at 200°C, Dry gas at 4.0 l/min.

(**D**) Gas chromatography (GC) was performed either with flame ionization detection (FID) or mass detection (MS). 2-methylpyridine (in ddH_20) was used as internal standard for the substrate screening and endpoint measurements; p-xylene (in DMSO) was used as internal standard for initial rates measurements of enzymatic reactions.
GC-MS analyses of the 24-DWPs screening was conducted; as not otherwise described; with a Shimadzu GC2010 instrument equipped with a Shimadzu QP2010 mass spectrometer using a Phenomenex ZB-5MSi column (30 m x 0.25 mm, 0.25 µm film, part number 7HG-G018-11) with helium as carrier gas and pressure (26.7 kPa) as control mode. Injector temperature: 250°C. Split mode with a split ratio of 10 at 1 µl injection. Ion source temperature: 200°C, Interface temperature: 280°C.
GC-FID analyses for endpoint and initial rates measurements were conducted; as not otherwise described; with a Shimadzu GC2010 Plus instrument using an Agilent J&W DB-

WAX column (30 m x 0.25 mm, 0.25 µm film, part number 122-7032) with hydrogen as carrier gas and linear velocity (41 cm/s) as flow control mode. Injector temperature: 250°C. Split mode with a split ratio of 15 at 1 µl injection. Detector temperature: 260°C.

GC-FID analyses for the measurement of selectivities of chemical syntheses were conducted; as not otherwise described; with a Shimadzu GC2010 Plus instrument using a Phenomenex ZB-5 column (30 m x 0.25 mm, 0.25 µm film, part number 7HK-G002-28) with hydrogen as carrier gas and linear velocity (41 cm/s) as flow control mode. Injector temperature: 250°C. Split mode with a split ratio of 50 at 1 µl injection. Detector temperature: 315°C.

1) **3-methylpyrazole (1), 1,3-dimethylpyrazole (1a), 1,5-dimethylpyrazole (1b)**

GC-FID analyses of screening, endpoint measurements and selectivity of chemical synthesis: GC-FID analyses were conducted with a Shimadzu GC2010 Plus instrument using an Agilent J&W DB-WAX column (30 m x 0.25 mm, 0.25 µm film, part number 122-7032) with hydrogen as carrier gas and linear velocity (33.1 cm/s) as flow control mode. Injector temperature: 200°C. Split mode with a split ratio of 15 at 1 µl injection. Detector temperature: 260°C. Oven temperature: 120°C, uphold 1 min, 17.5°C/min to 140°C, 40°C/min to 230°C uphold 3 min.

GC-FID analyses of initial rates measurements: Oven temperature: 110°C uphold 2 min, 17.5°C/min to 135°C, 40°C/min to 230°C uphold 3 min.

2) **3,4-dimethylpyrazole (2), 1,3,4-trimethylpyrazole (2a), 1,4,5-trimethylpyrazole (2b)**

GC-MS analyses of 24-DWP screening: Oven temperature: 60°C uphold 4 min, 15°C/min to 85°C, 25°C/min to 170°C, 50°C/min to 320°C uphold 3 min. SIM mode with 93 m/z, 95 m/z and 109 m/z.

GC-FID analyses of endpoint and initial rates measurements: Oven temperature: 110°C uphold 2 min, 25°C/min to 120°C, 50°C/min to 230°C, uphold 2 min.

GC-FID analyses of selectivity measurement of chemical synthesis: Oven temperature: 85°C, uphold 2 min, 25°C/min to 120°C, 15°C/min to 140°C.

3) **3-cyclopropylpyrazole (3), 1-methyl-3-cyclopropylpyrazole (3a), 1-methyl-5-cyclopropyl-pyrazole (3b)**

GC-MS analyses of 24-DWP screening: Oven temperature: 110°C, uphold 2 min, 50°C/min to 280°C, uphold 2 min. SIM mode with 93 m/z, 108 m/z and 122 m/z.

GC-FID analyses of endpoint measurements: Oven temperature: 110°C, uphold 2 min, 25°C/min to 120°C, uphold 3.5 min, 50°C/min to 230°C, uphold 2 min.

GC-FID analyses of Initial rates measurement: Oven temperature: 110°C, uphold 3 min, 25°C/min to 135°C, uphold 3 min, 50°C/min to 230°C, uphold 2.5 min.

GC-FID analyses of selectivity measurement of chemical synthesis: Oven temperature: 110°C, uphold, 2 min, 50°C/min to 280°C, uphold 2 min.

4) **3-cyclopropylpyrazole (3), 1-ethyl-3-cyclopropylpyrazole (7a), 1-ethyl-5-cyclopropylpyrazole (7b)**

GC-FID analyses of endpoint measurements and of selectivity measurement of chemical synthesis: Oven temperature: 110°C, uphold 3 min, 25°C/min to 135°C, uphold 3 min, 50°C/min to 230°C, uphold 2.5 min. Internal standard was p-xylene.

5) **3-cyclopropylpyrazole (3), 1-propyl-3-cyclopropylpyrazole (8a), 1-propyl-5-cyclopropyl-pyrazole (8b)**

GC-MS analyses of endpoint measurements: GC-MS analyses were conducted with an Agilent 8860 GC instrument equipped with an Agilent 5977B mass spectrometer using an Agilent DB-HeavyWAX column (25 m x 0.25 mm, 0.20 µm film, part number 122-7127) with helium as carrier gas and constant pressure as control mode. Split mode with a split ratio of 40 at 1 µl injection. Oven temperature: 80°C, uphold 2.25 min, 40°C/min to 270°C, uphold 3 min. Internal standard was p-xylene.

GC-FID analyses of selectivity measurement of chemical synthesis: Oven temperature: 110°C, uphold 3 min, 25°C/min to 135°C, uphold 3 min, 50°C/min to 230°C, uphold 2.5 min.

6) **3(2-furyl)pyrazole (4), 1-methyl-3(2-furyl)pyrazole (4a), 1-methyl-5(2-furyl)pyrazole (4b)**

GC-MS analyses of 24-DWP screening: Oven temperature: 110°C, uphold 2 min, 50°C/min to 280°C, uphold 2 min. SIM mode with 93 m/z, 134 m/z and 148 m/z.

GC-FID analyses of endpoint measurements: Oven temperature: 100°C, uphold 2.25 min, 50°C/min to 230°C, uphold 4.5 min.

GC-FID analyses of initial rates measurement: Oven temperature: 100°C, uphold 2.25 min, 50°C/min to 230°C, uphold 6.5 min.

GC-FID analyses of selectivity measurement of chemical synthesis: Oven temperature: 110°C, uphold 2 min, 50°C/min to 280°C, uphold 2 min.

7) **3(2-fluorophenyl)pyrazole (5), 1-methyl-3(2-fluorophenyl)pyrazole (5a), 1-methyl-5(2-fluorophenyl)-pyrazole (5b)**

GC-MS analyses of 24-DWP screening: Oven temperature: 110°C, uphold 2 min, 50°C/min to 280°C, uphold for 2 min. SIM mode with 93 m/z, 162 m/z and 176 m/z.

GC-FID analyses of endpoint measurements: Oven temperature: 100°C, uphold 2.25 min, 50°C/min to 230°C, uphold 6 min.

GC-FID analyses of initial rates measurement: Oven temperature: 100°C, uphold 2.25 min, 50°C/min to 230°C, uphold 9 min.

GC-FID analyses of selectivity measurement of chemical synthesis: Oven temperature: 110°C, uphold 2 min, 50°C/min to 280°C, uphold 2 min.

8) **3-methyl-4-phenylpyrazole (6), 1,3-dimethyl-4-phenylpyrazole (6a), 1,5-dimethyl-4-phenylpyrazole (6b)**

GC-MS analyses of 24-DWP screening: Oven temperature: 110°C, uphold 2 min, 50°C/min to 280°C, uphold 2 min. SIM mode with 93 m/z, 158 m/z and 172 m/z.

GC-FID analyses of endpoint measurements: Oven temperature: 100°C, uphold 2.25 min, 50°C/min to 230°C, uphold 8 min.

GC-FID analyses of initial rates measurement: Oven temperature: 100°C, uphold 2.25 min, 50°C/min to 230°C, uphold 12.75 min.

GC-FID analyses of selectivity measurement of chemical synthesis: Oven temperature: 110°C, uphold 2 min, 50°C/min to 280°C, uphold 2 min.

(**E**) Liquid chromatography (LC) Analysis

Detection and quantification of SAH, SAM and alkyl SAM analogs. High pressure liquid chromatography (HPLC) analyses were conducted with an Agilent 1200 series instrument equipped with a diode-array detector using a Phenomenex Luna® 5 µm SCX 100 Å (100 x 4.6 mm, part number 00D-4398-E0) with buffer A (20 mM KP_i pH 2.5, 25% (v/v) ACN) and buffer B (20 mM KP_i pH 2.5, 25% (v/v) ACN, 400 mM potassium chloride) and detection at 260 nm. Column temperature: 40°C at a flow rate of 0.5 ml/min with 4 µl injection. Gradient: 10% B, 8 min to 90% B, uphold 2 min, 4 min to 10% B, up hold for 3 min.

Mass detection of SAH, SAM and alkyl SAM analogs. Liquid chromatography mass spectroscopy (LC/MS) analyses were conducted with an Agilent 1260 infinity instrument

equipped with a quadrupole LC/MS 6130 detector using a Merck KGaA ZIC®-pHILIC column (50 x 4.6 mm, part number 1.50463.0001) with buffer A (90% ACN, 20 mM ammonium formate, pH 2.8) and buffer B (10% ACN, 20 mM ammonium formate, pH 2.8) and detection at 260 nm via DAD and 250-600 m/z in positive mode via MS-detector. Flow rate of 1 ml/min with 40 µl injection. Gradient: 20% B, uphold 1 min, 11 min to 75% B, 2 min to 100% B, uphold 2 min, 4 min to 20% B, uphold 10 min.

II. General procedures

(A) Cloning of wtNMT, FuncLib variants and HMTs:

Homo sapiens nicotinamide *N*-methyltransferase (wtNNMT, UniProtKB: P40261) was codon optimized for *E.coli* (https://eu.idtdna.com), ordered at Invitrogen (Thermo Fischer Scientific, California, USA) and inserted into pET28 a(+) plasmid, containing a C-terminal His_6-tag with a LE-linker, by Gibson Assembly[1]. The plasmid was transformed into *E.cloni* strain BL21 (DE3) via heat shock transformation.

FuncLib variants were constructed by Gibson Assembly, overlap extension PCR[2] and QuikChange-PCR with the template of pET28 a(+)::wtNMT. 300 bp Gibson Assembly fragments that cover the mutations L164X, D167X, D197X, A198X, S201X, Y204X, S213X, Y242X, A247X and N249X were ordered at TWIST Bioscience (California, USA). Single mutations at Y20 and Y24 were performed by overlap extension PCR; double mutations for this position were done by QuikChange-PCR. The plasmid were transformed into *E.cloni* strain BL21 (DE3) via heat shock transformation.

NSA-synthase *Aspergillus clavatus* halide methyl transferase *(acl*HMT, UniProtKB: A1CIS5), *Chloracidobacterium thermophilum* HMT (*cth*HMT, UniProtKB: G2LF24), *Arabidopsis thaliana* HMT (*ath*HMT, UniProtKB: Q6AWU6), *Batis maritima* HMT (*bma*HMT, UniProtKB: Q7ZSZ7), *Burkholderia xenovorans* (*bxe*HMT, NCBI: WP_011486779) and *Synechococcus elongatus* (*sel*HMT, UniProtKB: Q31S13), were codon optimized for *E.coli* (https://eu.idtdna.com), ordered at TWIST Bioscience (California, USA) and inserted into a pBAD33 vector containing a C-terminal His_6-tag with a LE-linker, by Gibson Assembly. The plasmids were transformed into the SAHN-knockout *E.coli* strain JW0155 (Keio-collection[3]) via heat shock transformation.

(B) Creation of FuncLib library:

Analysis of crystal structures of wtNMT (PDB: 2iip, 3rod, 5yif. 6b1a, 6chh) showed no structural difference in the active site of wtNMT crystals, even with different substrates bound. We identified the residues of the substrate-binding pocket 5 Å around the bound inhibitor in 5yjf and excluded residues that interact with SAM or SAH. We picked 12 amino acid residues (Y20, Y24, L164, D167, D197, A198, S201, Y204, S213, Y242, A247, and N249) for the FuncLib[4] application (http://funclib.weizmann.ac.il/bin/steps).

The library was created using PDB code of wtNMT 2iip, chain A with SAH as ligand (4001A) to keep. We used min ID 0.3, max targets 3000, coverage 0.6 and E value of 0.0001 resulting in 473.294 designs in step 1. For energy calculations and clustering, we set up the parameters: minimal number of mutations per design = 3, maximal number of mutations per

design = 5, minimal PSSM threshold = -1, ΔΔG = 5.5 and difference between clustered variants = 3.

(C) Expression for 24-deep well plate (DWP) screening:

Overnight cultures in 24 DWPs with 4 ml/well TB media and 30 mg/l kanamycin were inoculated with single colonies from LB agar plates with the same concentration of kanamycin and incubated at 37°C. Main cultures were inoculated with 400 µl of the overnight culture using 24 DWPs with 3.4 ml/well TB media (with 30 mg/l kanamycin) and incubated for 4 h at 37°C and 180 rpm shaking. Each plate had controls of 2x wtNMT, 1x empty vector and 1 well only with media. After cooling the plates in an ice bath for 15 min, the expression was started by induction with 1 mM IPTG for 20 h, 25°C and 180 rpm. The cells were harvested at 3220 x g, 4°C for 10 min and frozen and stored at -20°C for at least 1 night.

(D) Expression of wtNMT and FuncLib variants for enzyme purification:

Overnight cultures in 5 ml LB media with 30 mg/l kanamycin were inoculated either from single colonies on LB agar plates or from glycerol stocks that were stored at -80°C and incubated at 37°C. Main cultures in 400 ml autoinduction media (12 g/l tryptone, 24 g/l yeast extract, 11.1 g/l glycerol, 2.9 g/l D-(+)-glucose, 7.6 g/l α-lactose, 1% TB-salts), containing 30 mg/l kanamycin, were inoculated with the overnight cultures to a starting concentration of 1% (v/v) and the expression was started by autoinduction. The cultures were incubated for 24 h at 37°C and 180 rpm. The cells were harvested at 10.000 x g at 4°C for 10 min and used directly or were frozen and stored at -20°C.

(E) Expression of HMTs for screening and enzyme purification:

Overnight cultures in 5 ml LB media with 50 mg/l chloramphenicol were inoculated either from single colonies on LB agar plates or from glycerol stocks that were stored at -80°C and incubated at 37°C. Main cultures in 400 ml TB media, containing 50 mg/l chloramphenicol, were inoculated with the overnight cultures to a starting concentration of 1% (v/v) and incubated at 37°C, 180 rpm for 3 h. After cooling the cultures in an ice bath for 15 min, the expression was started by induction with 100 mM L-arabinose for 20 h at 25°C and 180 rpm. The cells were harvested at 10.000 x g at 4°C for 10 min and used directly or were frozen and stored at -20°C.

(F) Enzyme purification of wtNMT and FuncLib variants:

Frozen cells were resuspended and homogenized in 3 ml/g_{cww} Lysis buffer (50 mM KP_i, 10 mM imidazole, 500 mM NaCl, 5% glycerol, pH 7.6) and disrupted at 4°C and 800 bar with an Emulsiflex-C5 (Avestin Inc.). Soluble fraction was obtained after centrifugation (4°C, 55.000 x g, 1 h) and filtered through 0.2 µm. Enzyme purification was conducted at 4°C with and

Äkta™ Purifier (GE-healthcare) and a Histrap HP 5 ml column (GE-healthcare), loaded with Ni^{2+}. The purification was followed by UV/Vis-detector at 280 nm. The column was equilibrated with lysis buffer for 2 column volumes (cv) at 5 ml/min flow. Lysate was loaded and washed from the column for 20 cv. First washing step was 10% elution buffer (50 mM KP_i, 500 mM imidazole, 500 mM NaCl, 5% glycerol, pH 7.6) for 12 cv and elution was at 50% elution buffer for 7 cv followed by a wash out with 100% elution buffer for 5 cv and reequilibration with lysis buffer for 3 cv. Enzyme containing fractions were detected by SDS-PAGE and pooled and dialyzed with an dialysis bag (6-8 kDa cutoff) in dialysis buffer (50 mM KP_i, 5% glycerol, pH 7.6) over night and 2x 3 h the next day. Protein concentration was measured via Micro BCA™-Assay (Thermo Scientific, Prod.-# 23225) and aliquots of the enzyme were frozen and stored at -80°C.

(G) Enzyme purification of NSA-synthase (*acl*HMT):

Frozen cells were resuspended and homogenized in 3 ml/g_{cww} lysis buffer (50 mM KP_i, 10 mM imidazole, 500 mM NaCl, 5% glycerol, pH 7.6) and disrupted at 4°C and 800 bar with an Emulsiflex-C5 (Avestin Inc.). Soluble fraction was obtained after centrifugation (4°C, 55.000 x g, 1 h) and filtered through 0.2 µm. Enzyme purification was conducted at 4°C with an Äkta™ Purifier (GE-healthcare) and a Histrap HP 5 ml column loaded with Ni^{2+}. The purification was followed by UV/Vis-detector at 280 nm. The column was equilibrated with lysis buffer for 2 cv at 5 ml/min flow. Lysate was loaded and washed from the column for 20 cv. First washing step was 6% elution buffer (50 mM KP_i, 500 mM imidazole, 500 mM NaCl, 5% glycerol, pH 7.6) for 7 cv, second washing step at 13% elution buffer for 7 cv and elution was at 25% elution buffer for 7 cv followed by a wash out with 100% elution buffer for 5 cv and reequilibration with lysis buffer for 3 cv. Enzyme containing fractions were pooled and dialyzed with an dialysis bag (6-8 kDa cutoff) in dialysis buffer (50 mM KP_i, 5% glycerol, pH 7.6) over night and 2x 3 h the next day. Protein concentration was measured via Micro BCA™-Assay and aliquots of the enzyme were frozen and stored at -80°C.

(H) Biotransformations for 24 deep well plate screening:

Frozen cells were unfrozen and lysed with 500 µl/well lysis buffer containing 50 mM KP_i, pH 7.0, 1 mg/mL lysozyme and 0.2 mg/mL DNase1 for 1 h at 37°C and 180 rpm. The lysate was centrifuged at 4°C with 3320 x g for 15 min to receive the soluble supernatant. 500 µl biotransformations were performed in 1.5 ml reaction tubes (Sarstedt, Germany) with 400 µl lysate, 2 mM of corresponding pyrazole (**1**-**6**), 2 mM SAM disulfate tosylate and 1% (v/v) DMSO for 20 h at 37°C and 180 rpm in an Infors Multitron Pro. Extraction was performed with 500 µl DCM and 2-methylpyridine was added as internal standard for GC-MS measurement.

(I) Biotransformations for endpoint measurements:

Frozen enzymes were unfrozen rapidly and 500 µl biotransformations were performed in 1.5 ml reaction tubes (Sarstedt, Germany) with 50 µM of purified enzyme, 2 mM of corresponding pyrazole (**1**-**6**), 4 mM SAM disulfate tosylate and 1% (v/v) DMSO for 20 h at 37°C and 180 rpm. Extraction was performed with 500 µl DCM and 2-methylpyridine was added as internal standard for GC-FID measurement.

(J) Biotransformations for initial rates measurements:

Frozen enzymes were unfrozen rapidly at 37°C and 500 µl biotransformations were performed in 1.5 ml reaction tubes (Sarstedt, Germany) with 50 µM of purified enzyme, 2 mM of corresponding pyrazole (**1**-**6**), 2 mM SAM disulfate tosylate and 1% (v/v) DMSO at 37°C and 550 rpm in an Eppendorf Thermomixer. The reaction mixtures were prewarmed at 37°C and started by the addition of SAM. The reactions were stopped by the addition of 500 µl DCM and mixing for 40 s. p-xylene was added as internal standard for GC-FID measurement. Slopes of initial rates are calculated by Microsoft Excel.

(K) Biotransformations for the enzymatic generation of alkyl SAM analogs:

500 µl biotransformations were performed in 1.5 ml reaction tubes (Sarstedt, Germany) with 50 µM of purified NSA-synthase *acl*HMT, 10 mM of corresponding haloalkane, 1 mM SAH and 1% (v/v) DMSO at 25°C and 550 rpm for 20 h in an Eppendorf Thermomixer. The reaction was stopped by the addition of 100 µl 32% HCl and 200 µl ACN and mixing for 40 s. After centrifugation, the soluble fraction was transferred to HPLC and LC-MS measurements.

(L) Biotransformations for the enzymatic cascade for the alkylation of 3-cyclopropylpyrazole (3):

500 µl biotransformations were performed in 1.5 ml reaction tubes (Sarstedt, Germany) with 50 µM of purified variant v36 and NSA-synthase respectively, 2 mM of **3**, 20 mM haloalkane and 1% (v/v) DMSO at, as not otherwise described, 37°C and 550 rpm for 30 h to 72 h in an Eppendorf Thermomixer. The reaction was stopped by the addition of 500 µl DCM and mixing for 40 s. p-xylene was added as internal standard for GC-FID or GC-MS measurement.

(M) Scale-up biotransfromation for the enzymatic cascade for the methylation of 3-cyclopropylpyrazole (3):

500 ml biotransformation (1000x scale-up) was performed in 1 l Schott® bottle with 50 µM of purified variant v36 and NSA-synthase *acl*HMT respectively, 2 mM of **3**, 20 mM iodomethane at 37°C and 150 rpm for 30 h in an Infors Aquatron. 3 samples (500 µl) were taken at the end

of the reaction and extracted with 500 µl DCM to measure the GC-yield with p-xylene as internal standard. The remaining reaction was extracted 4 times with 250 ml DCM, dried with magnesium sulfate and evaporated carefully with a rotary evaporator under reduced pressure. The products were purified from the educt with a silica M60 column with DCM: ethyl acetate (4:1) and followed by TLC with vanilla staining and GC-FID. Fractions containing the product and fractions containing the educt were pooled and evaporated carefully with a rotary evaporator and analyzed by NMR.

(O) General protocol for chemical synthesis of alkylated pyrazoles:

The synthesis was performed based on the previous described protocol.[5] The pyrazole-educt **1-6** (1 equivalent) was dissolved in dry THF at 0°C under moderate stirring. 1 equivalent of NaH and 1.5 equivalents of haloalkane was added at room temperature and after 24 h, the reaction was quenched with ddH_2O and extracted 2-4 times with DCM. A sample of the crude product was taken for measurement of the chemical selectivity via GC-FID. The crude extract was dried using magnesium sulfate and evaporated using a rotary evaporator under reduced pressure. The resisting crude extract was purified by column chromatography using silica gel M60 and mixtures of ethyl acetate and cyclohexane as solvent. The purification was followed by thin layer chromatography and visualized by fluorescence at 254 nm, vanilla staining or by GC-FID. The purity of the collected fractions was always determined by GC-FID. The pure fractions were combined and dried at the rotary evaporator until all solvent was evaporated. The samples were characterized by NMR. Identified structures were further confirmed by high-resolution mass analysis.

III. Supporting figures

Figure S1: Chemical methylation and alkylation of pyrazoles generates product mixtures

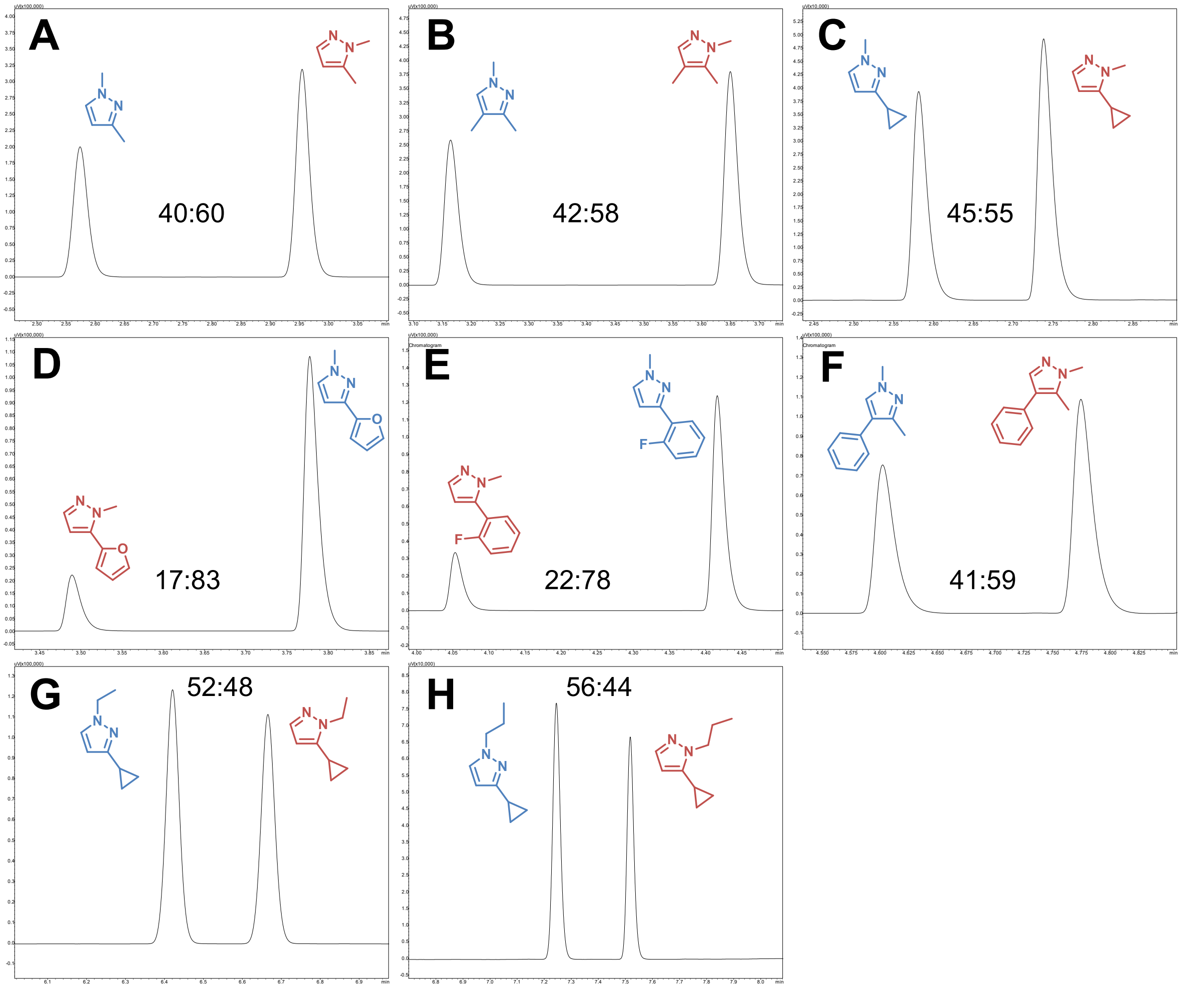

The chemical alkylation of pyrazoles **1-6** with the protocol described in **II.(O)** results in all cases in product mixtures that are often challenging to separate.

Figure S2: Examples of important bioactive pyrazoles

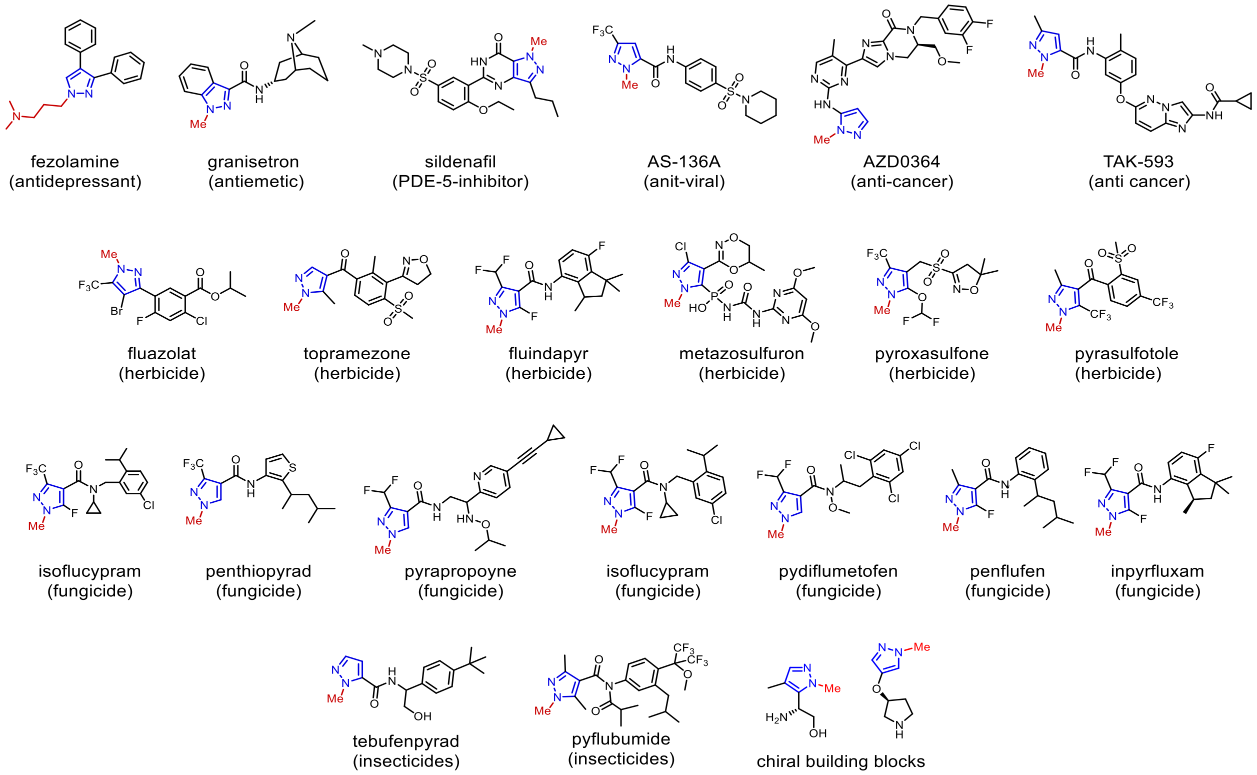

Methylated and alkylated pyrazoles are important active agents in pharmacy and agrochemistry. Besides pharmaceutical activities (antiemetic, anti viral, anti-cancer,…), these compounds also function as herbicides, fungicides and insecticides. The alkylation pattern often implements enormous effects on potency as well half-life and even change the bioactivity of these compounds.[6] Here, mostly *N*-methylated pyrazoles are displayed, but similar figures can as well be generated using *N*-alkylated pyrazoles.

Figure S3: Typical strategies to form *C*-heteroatom bonds in nature depend on complex leaving groups

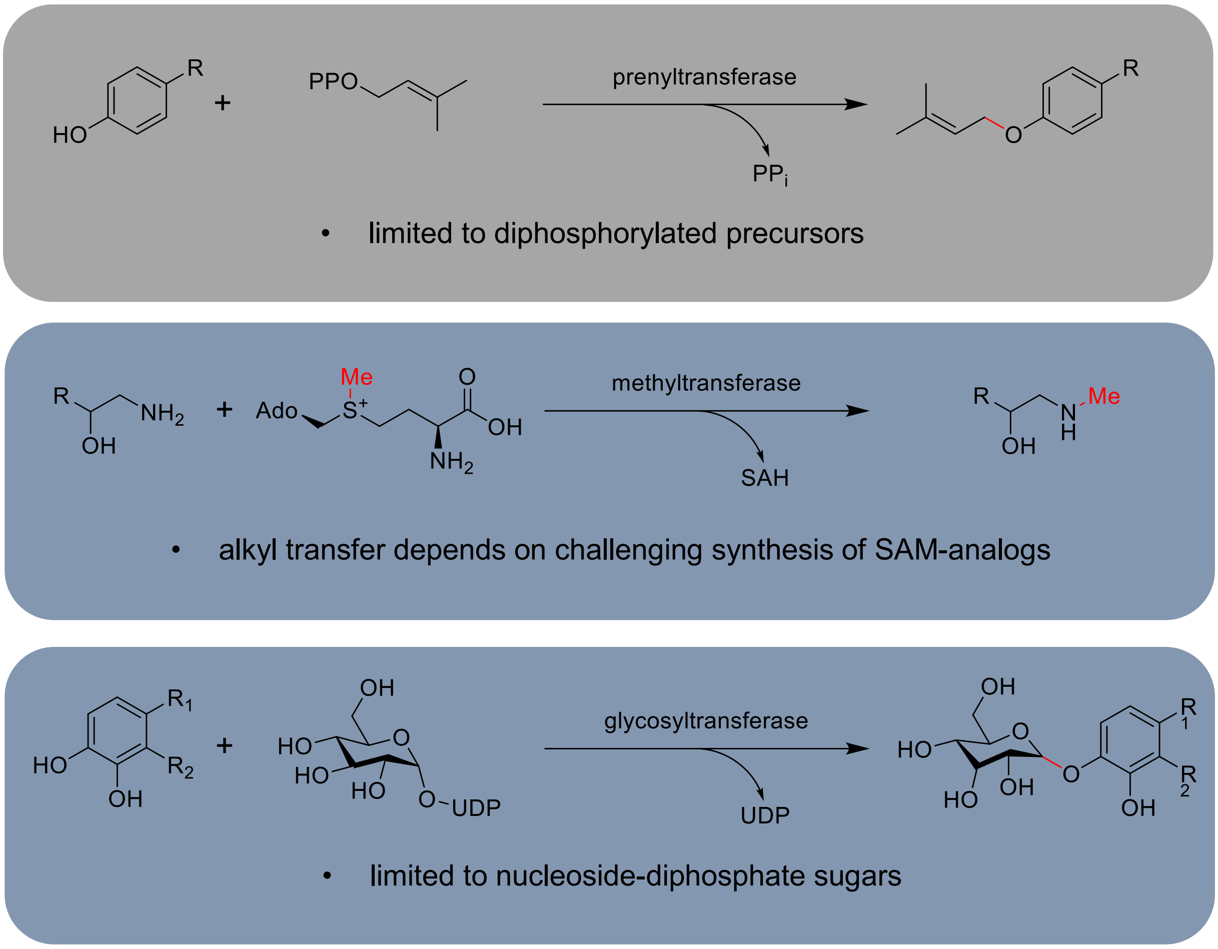

Natural alkylating enzymes to form *C*-heteroatom bonds can be divided in 3 major classes: prenyltransferases, methyltransferases and glycosyltransferases.[7–13] These enzymes are limited to specific alkyl precursors that are often unstable and challenging to access via synthesis. Further, the corresponding leaving groups (diphosphate, SAH or nucleoside-derivatives) lead to low atom economy.

Figure S4: Methods for enzymatic and chemoenzymatic NSA synthesis

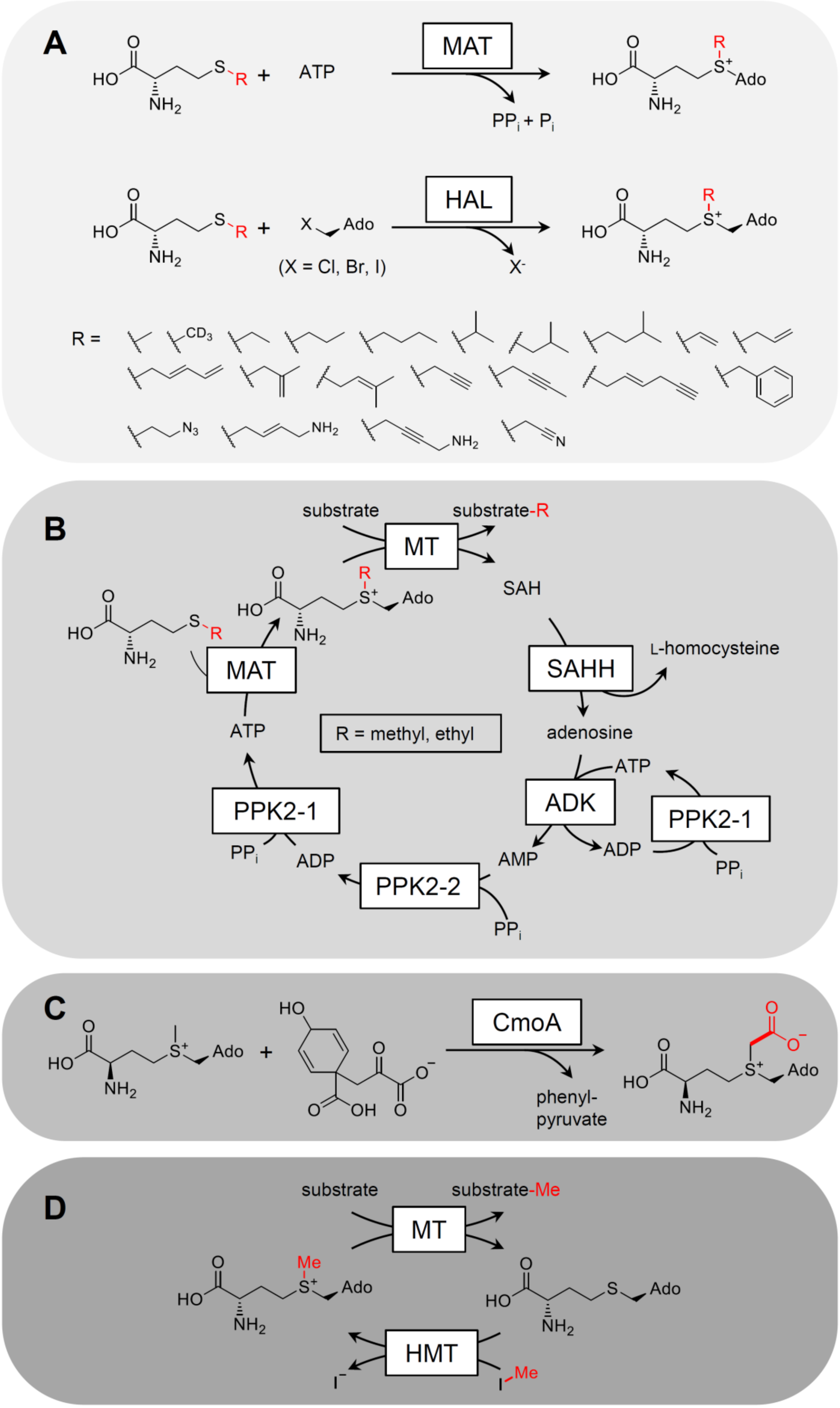

Systems for chemoenzymatic and enzymatic production of non-natural *S*-adenosyl-L-methionine (SAM) analogs (NSAs). **A)** Engineered methionine-adenosyl-transferases (MAT) and halogenases (HAL) are capable of synthesizing various functionalized alkyl- and aryl-analogs of SAM.[14,15] Both approaches depend on chemical synthesis of L-methionine analogs. **B)** Mordhorst & Andexer et al.[16] published in 2017 the first system for the recycling of SAM, where SAM is recycled under the stoichiometric use of L-methionine and L-ethionine but catalytic amounts of ATP. After transfer of the methyl group derived from SAM by a methyl transferase (MT) onto a substrate, the resulting *S*-adenosyl-L-homocysteine (SAH) is depleted by *S*-adenosyl-L-homocysteine-hydrolase (SAHH) into L-homocysteine and adenosine. Adenosine is further recycled by the kinases PPK2-1 and PKK2-2 to ATP that can be reloaded with L-methionine to achieve SAM by MAT. In this case the SAM-analogs are produced by MAT (as described in **A**), therefore, this approach also depends on the chemical synthesis of L-methionine analogs. **C)** Herbert & Micklefield et al.[17] published recently an approach of using carboxyl-SAM-synthase (CmoA) for loading the carboxyl group of prephenate onto SAM that could further be applied to *O*- and *N*-methyltransferases. **D)** Liao & Seebeck et al.[18] published a system for the recycling of SAM with the benefit of using methyl iodide as a simple methyl source.

Figure S5: Active site of wtNMT and choosen amino acid residues for computational library design

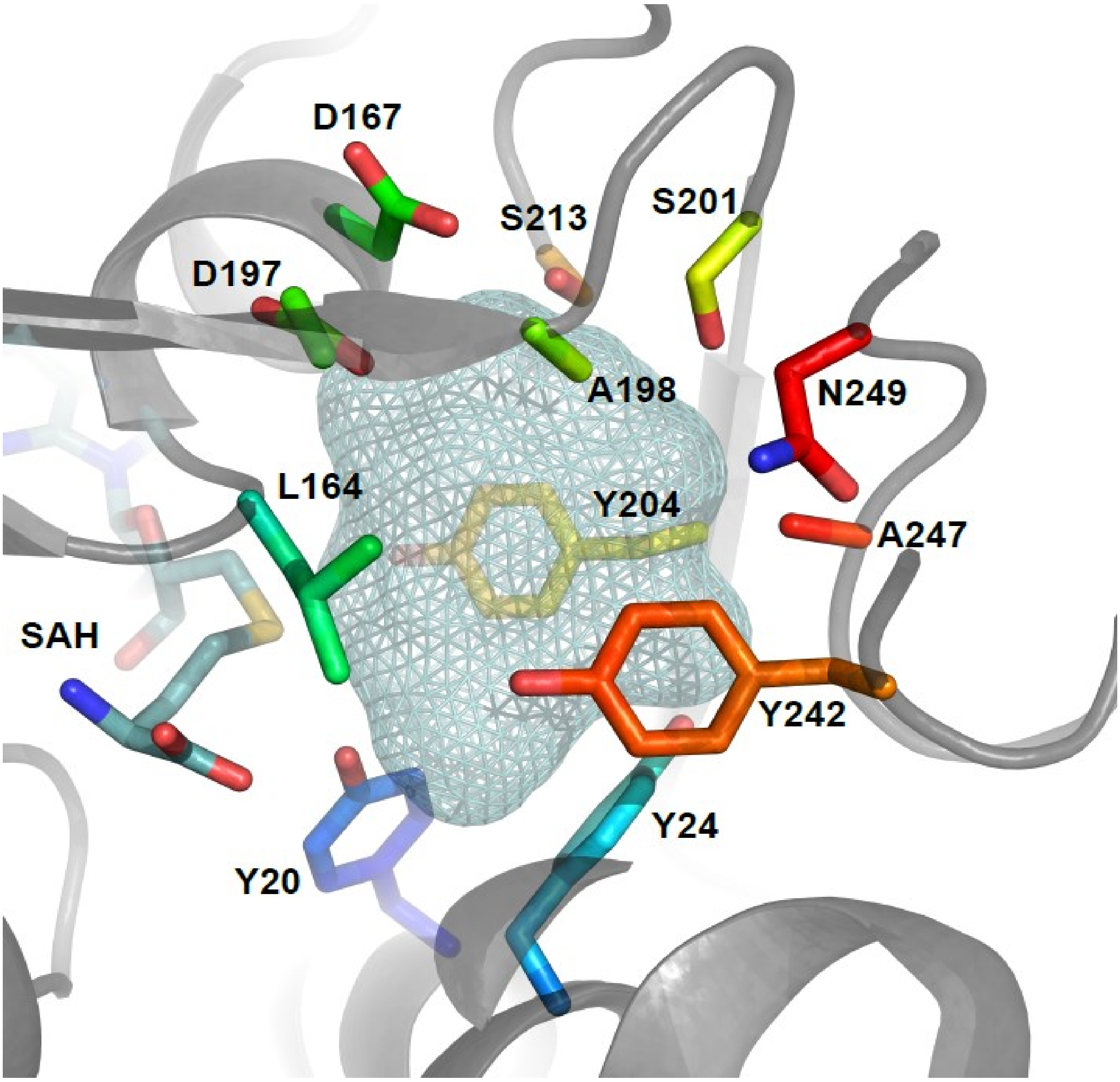

Active site of wtNMT (pdb-file 2iip) with SAH and chosen amino acid residues with substrate interactions for multiple point mutations. We identified the residues of the substrate-binding pocket 5 Å around the bound inhibitor in 5yjf and excluded residues that interact with SAM or SAH. We picked 12 amino acid residues (Y20, Y24, L164, D167, D197, A198, S201, Y204, S213, Y242, A247, and N249) for the FuncLib[4] application. Please see general protocols **B** for details.

Figure S6: Top 50 variants of FuncLib library

	active site amino acid positions											
#	20	24	164	167	197	198	201	204	213	242	247	249
wt	Y	Y	L	D	D	A	S	Y	S	Y	A	N
top 50 FuncLib variants 1	H	Y	L	C	D	M	A	Y	A	Y	A	N
2	Y	Y	L	H	D	M	C	Y	A	Y	A	C
3	Y	Y	L	H	D	S	R	Y	S	W	A	S
4	F	Y	L	H	D	M	C	Y	C	Y	A	N
5	Y	Y	L	H	D	M	A	Y	A	F	A	N
6	Y	Y	L	H	D	T	C	Y	A	Y	M	N
7	Y	Y	L	H	D	A	Q	Y	A	W	A	A
8	Y	Y	L	D	G	M	C	Y	A	Y	A	N
9	Y	Y	L	C	D	T	N	Y	A	F	A	N
10	Y	Y	L	H	D	M	A	Y	H	Y	A	A
11	Y	Y	L	C	D	L	C	Y	A	Y	A	A
12	F	Y	L	D	G	T	R	Y	S	Y	A	N
13	F	Y	L	H	D	A	Q	Y	S	Y	A	S
14	F	Y	L	H	D	L	A	Y	A	Y	A	N
15	Y	Y	L	H	D	A	T	Y	H	F	A	S
16	Y	Y	L	H	D	A	N	Y	A	Y	M	A
17	Y	Y	L	C	D	T	Q	Y	A	Y	S	N
18	Y	F	L	C	D	A	R	Y	S	W	A	A
19	H	Y	L	H	D	A	Q	Y	S	W	A	C
20	Y	Y	L	D	G	M	A	Y	S	F	A	A
21	Y	F	L	H	D	A	E	Y	A	Y	A	A
22	Y	Y	L	H	D	A	A	Y	H	Y	M	C
23	Y	Y	L	D	G	T	S	Y	A	W	A	A
24	Y	F	L	H	D	A	Q	Y	A	F	A	N
25	Y	F	L	H	D	M	S	Y	A	Y	A	N
26	Y	Y	L	H	D	T	Q	Y	S	Y	A	N
27	Y	Y	L	C	D	S	S	Y	A	W	A	A
28	Y	Y	L	H	D	A	C	Y	M	Y	A	A
29	Y	Y	L	C	D	A	T	Y	A	F	A	A
30	Y	Y	L	C	D	A	Q	Y	S	W	A	S
31	Y	Y	L	H	D	M	C	Y	S	F	A	S
32	Y	F	L	H	D	A	Q	Y	S	Y	A	C
33	Y	Y	L	C	D	M	C	Y	C	F	A	N
34	Y	Y	L	H	D	A	E	Y	A	F	A	C
35	Y	Y	L	D	G	L	A	Y	A	F	A	N
36	Y	F	L	C	D	T	C	Y	A	Y	A	N
37	Y	Y	L	H	D	T	E	Y	A	W	A	N
38	F	Y	L	C	D	A	M	Y	S	F	A	A
39	F	Y	L	H	D	M	S	Y	A	Y	A	A
40	Y	Y	L	H	D	L	A	Y	C	Y	A	C
41	F	Y	L	H	D	A	R	Y	S	W	A	A
42	H	Y	L	H	D	M	A	Y	S	Y	A	C
43	Y	Y	L	C	D	M	T	Y	T	Y	A	N
44	Y	Y	L	H	D	M	C	F	H	Y	A	N
45	F	Y	L	H	D	M	S	Y	S	F	A	C
46	Y	Y	L	D	G	M	S	Y	A	F	C	N
47	Y	Y	L	E	G	A	N	Y	A	F	A	N
48	F	Y	L	C	D	M	C	Y	S	Y	A	C
49	Y	Y	L	C	D	A	C	Y	H	W	A	A
50	H	F	L	H	D	A	R	Y	S	Y	A	N

The top 50 FuncLib variants (v1-v50) and their active site amino acids (AAs) in comparison to the wtNMT. Introduced mutations for each design are marked in red. All variants bearing 3-5 active site mutations and differ in at least 3 residues. Variants are ranked by calculated ΔΔG.

Figure S7: Mean standard deviation (CV) of the 24 deep well plate screening

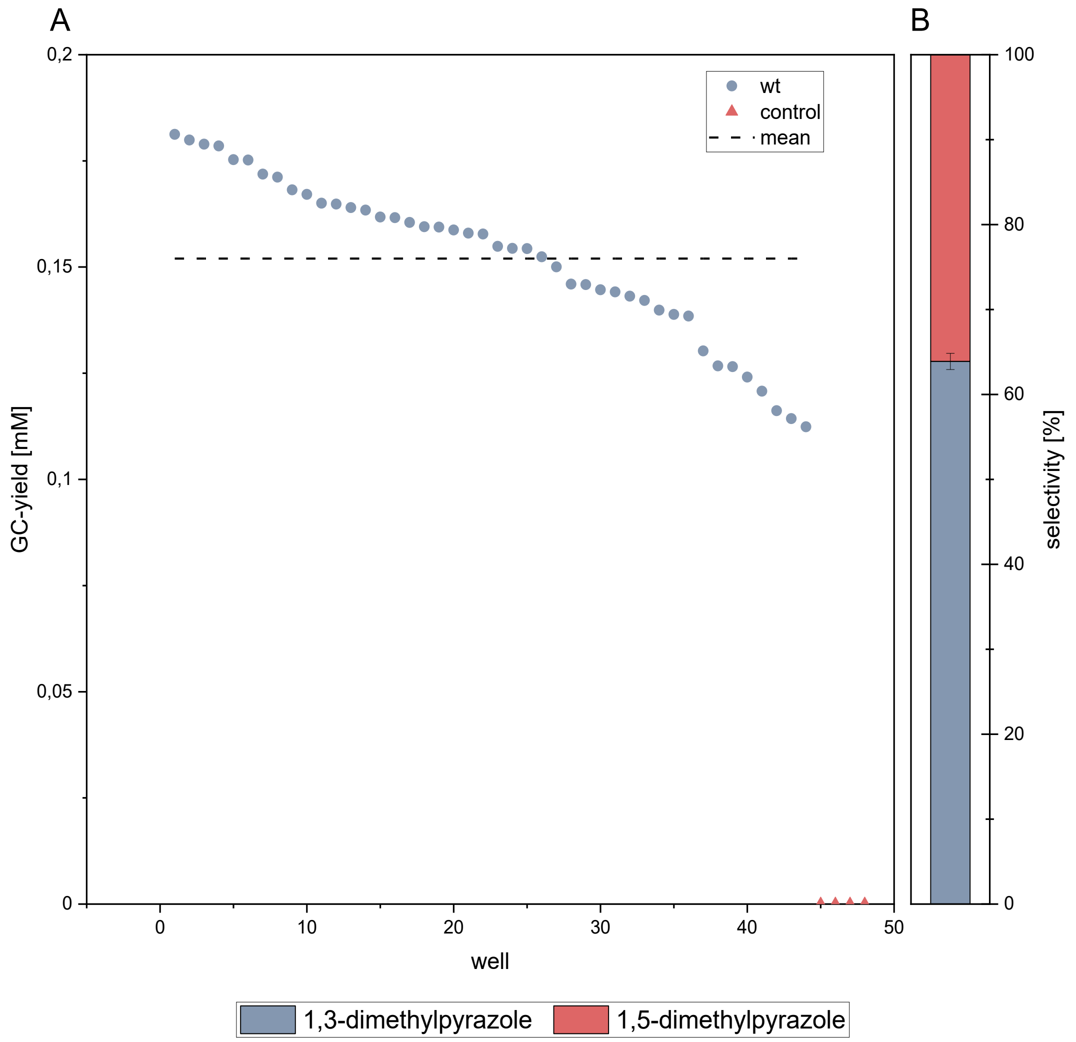

The mean standard deviation (CV) and the selectivity of the 24 deep well plate (DWP) screening was measured using 400 µl wtNMT lysate, 2 mM 3-methylpyrazole and 2 mM SAM at 37°C and 20 h according to paragraph **II.(H)**. **A)** The CV of 2x 24-DWPs was 12.2% and the empty vector control showed no activity. **B)** The wt selectivity under screening conditions was 64% for 1,3-dimethylpyrazole with a sdev of 1%.

Figure S8: Screening of enzymatic methylation of 3-methylpyrazole

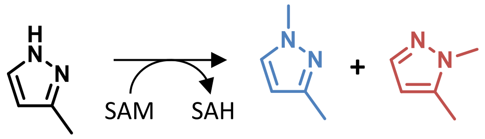

1 1a 1b

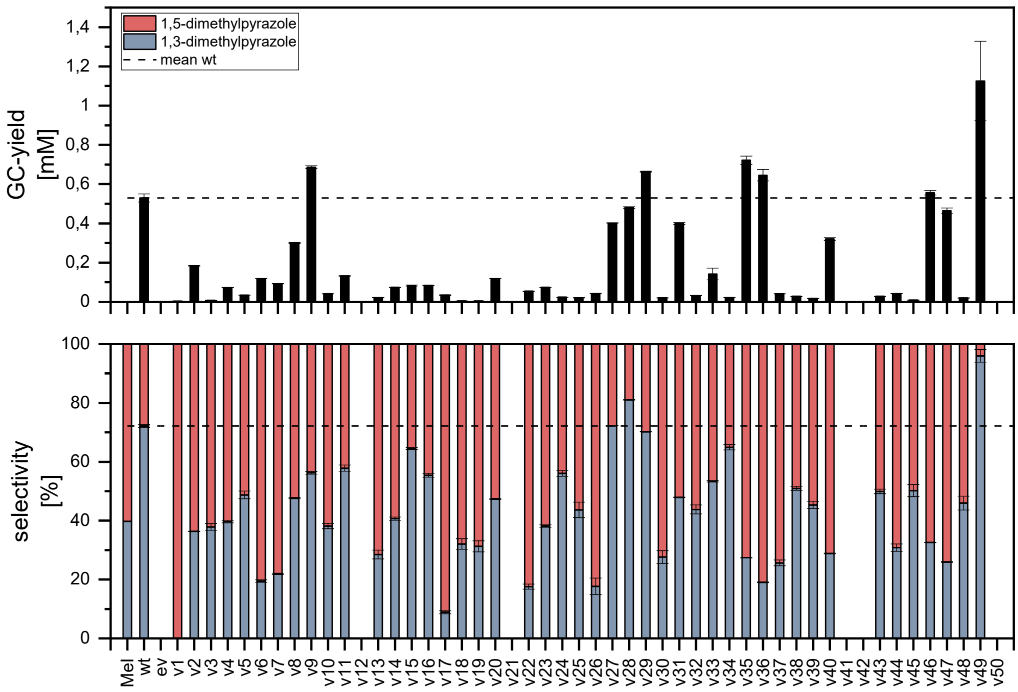

The screening of the substrate 3-methylpyrazole (**1**) was performed using 400 µl lysate, and 2 mM substrate and SAM respectively at 37°C and 20 h according to paragraph **II (H)**. The upper graph shows the total GC-yield of the reactions (black bars) and the mean wtNMT-yield (dashed line). The underneath graph shows the selectivity of the enzymes, with the selectivity of chemical methylation with methyl iodide (MeI) as reference and the empty vector (ev) as a control. The mean wt selectivity is shown in dashed lines. Error bars show standard deviation of triplicates.

Figure S9: Screening of enzymatic methylation of 3,4-dimethylpyrazole

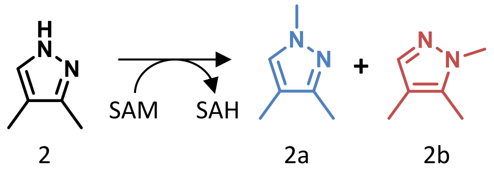

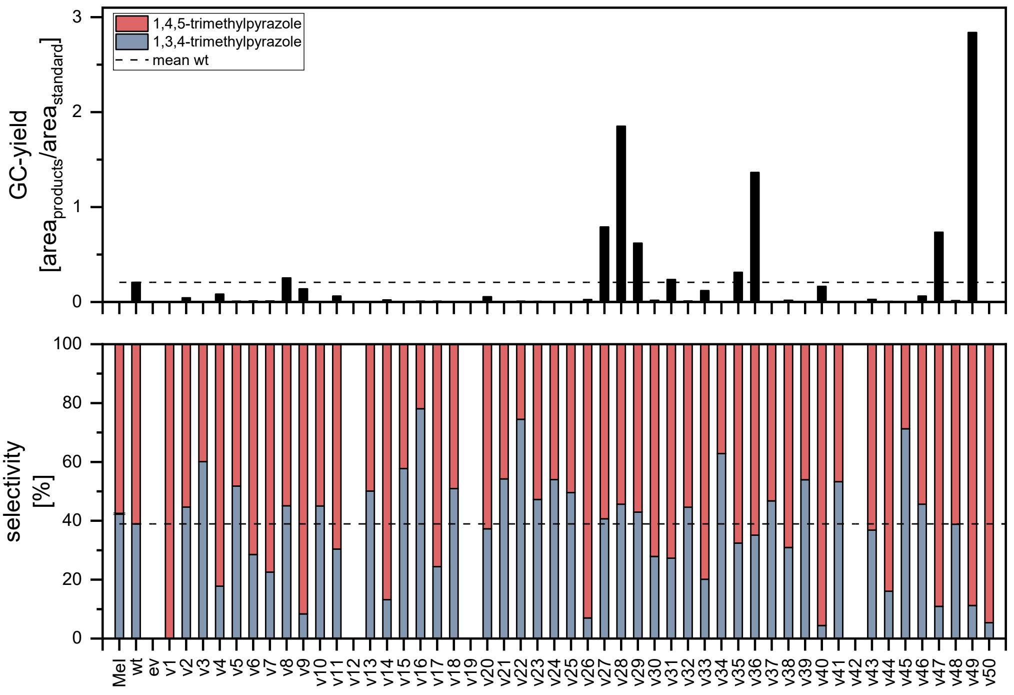

The screening of the substrate 3,4-dimethylpyrazole (**2**) was performed using 400 µl lysate, and 2 mM substrate and SAM respectively at 37°C and 20 h according to paragraph **II (H)**. The upper graph shows the total GC-yield of the reactions (black bars) and the mean wtNMT-yield (dashed line). The underneath graph shows the selectivity of the enzymes, with the selectivity of chemical methylation with methyl iodide (MeI) as reference and the empty vector (ev) as a control. The mean wt selectivity is shown in dashed lines. Error bars for wt show standard deviation of triplicates.

Figure S10: Screening of enzymatic methylation of 3-cyclopropylpyrazole

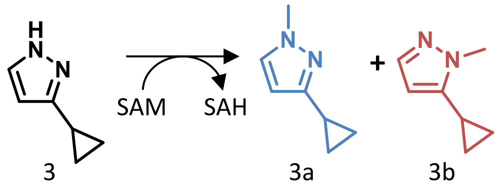

3 3a 3b

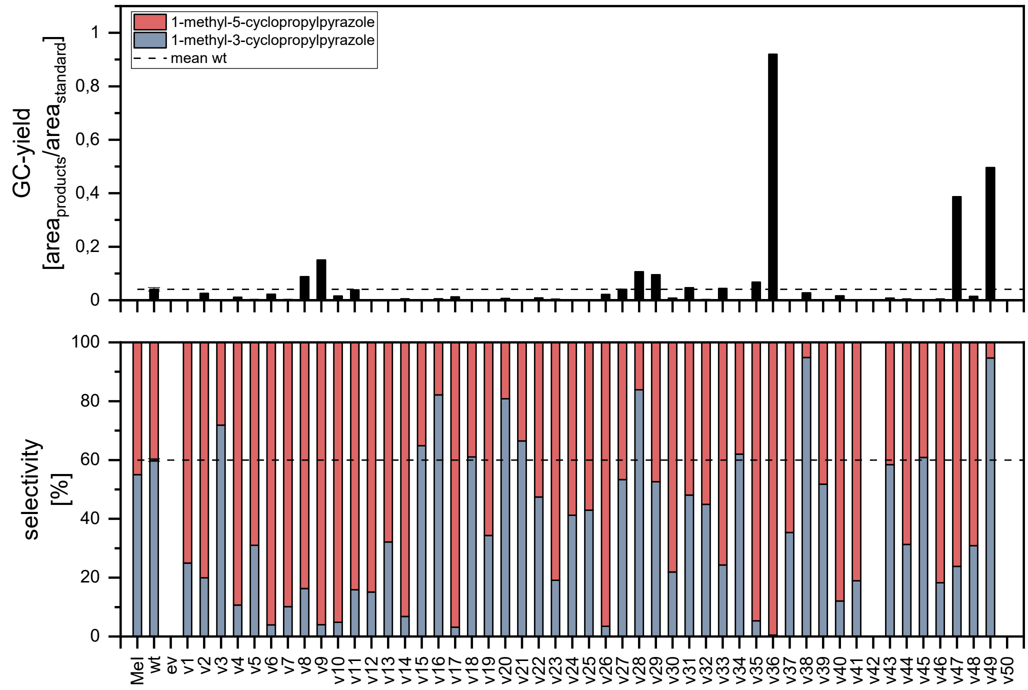

The screening of the substrate 3-cyclopropylpyrazole (**3**) was performed using 400 µl lysate, and 2 mM substrate and SAM respectively at 37°C and 20 h according to paragraph **II (H)**. The upper graph shows the total GC-yield of the reactions (black bars) and the mean wtNMT-yield (dashed line). The underneath graph shows the selectivity of the enzymes, with the selectivity of chemical methylation with methyl iodide (MeI) as reference and the empty vector (ev) as a control. The mean wt selectivity is shown in dashed lines. Error bars for wt show standard deviation of triplicates.

Figure S11: Screening of enzymatic methylation of 3(2-furyl)pyrazole

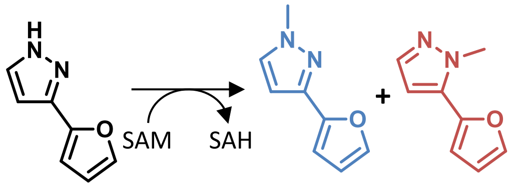

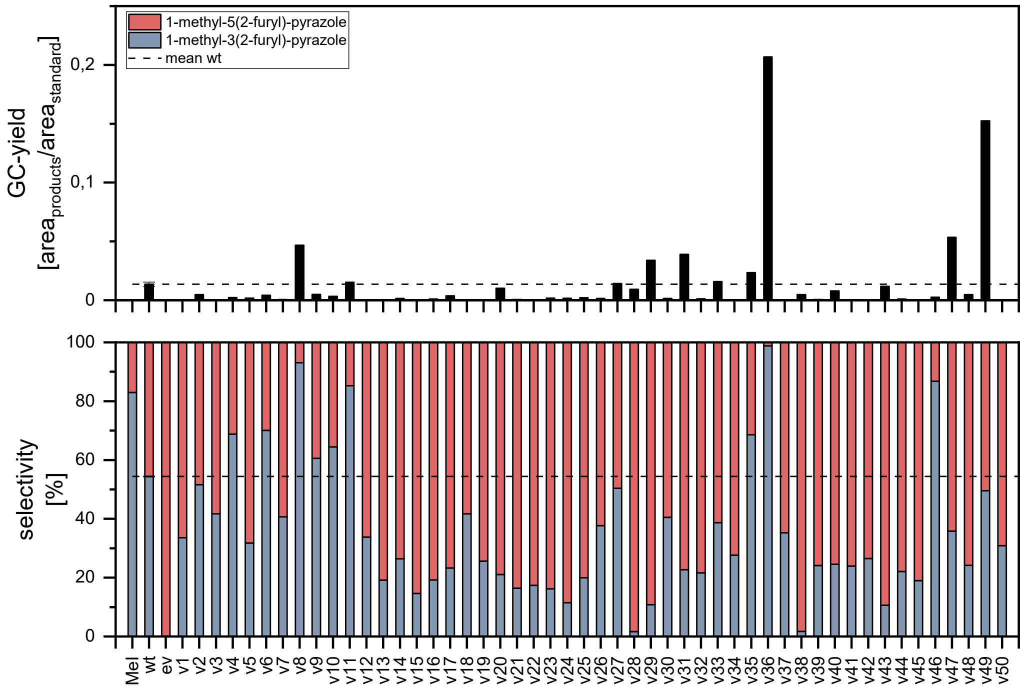

The screening of the substrate 3(2-furyl)pyrazole (**4**) was performed using 400 µl lysate, and 2 mM substrate and SAM respectively at 37°C and 20 h according to paragraph **II (H)**. The upper graph shows the total GC-yield of the reactions (black bars) and the mean wtNMT-yield (dashed line). The underneath graph shows the selectivity of the enzymes, with the selectivity of chemical methylation with methyl iodide (MeI) as reference and the empty vector (ev) as a control. The mean wt selectivity is shown in dashed lines. Error bars for wt show standard deviation of triplicates.

Figure S12: Screening of enzymatic methylation of 3(2-fluorophenyl)pyrazole

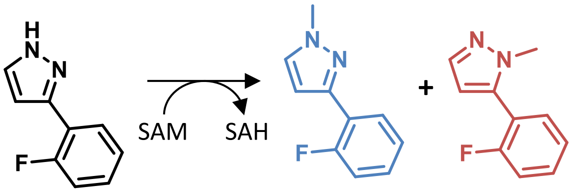

5 5a 5b

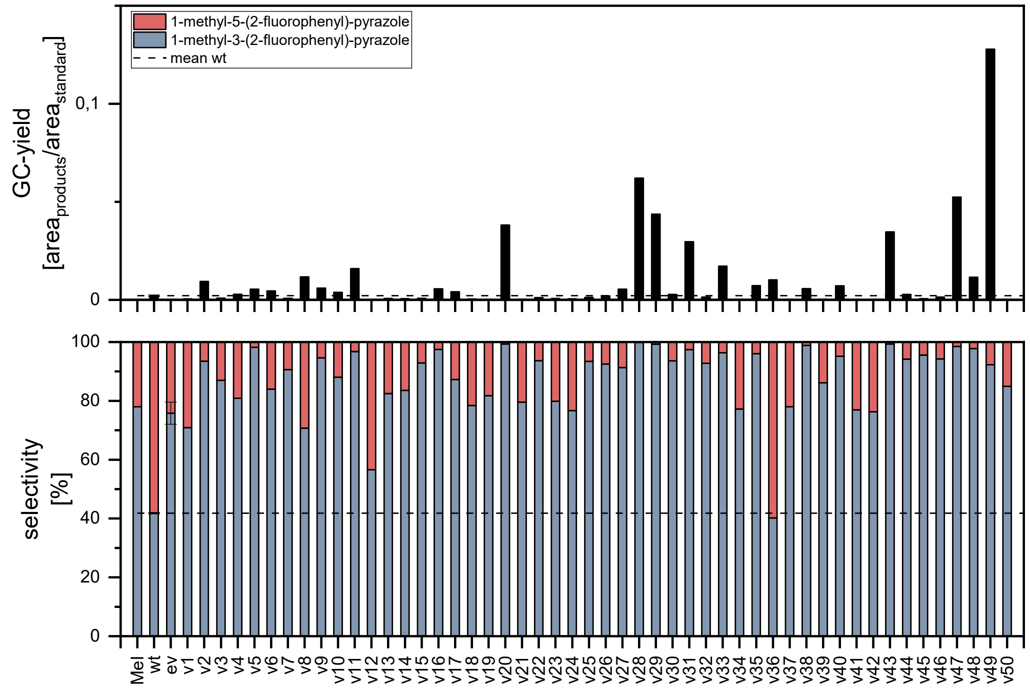

The screening of the substrate 3(2-fluorophenyl)pyrazole (**5**) was performed using 400 µl lysate, and 2 mM substrate and SAM respectively at 37°C and 20 h according to paragraph **II (H)**. The upper graph shows the total GC-yield of the reactions (black bars) and the mean wtNMT-yield (dashed line). The underneath graph shows the selectivity of the enzymes, with the selectivity of chemical methylation with methyl iodide (MeI) as reference and the empty vector (ev) as a control. The mean wt selectivity is shown in dashed lines. Error bars for wt show standard deviation of triplicates.

Figure S13: Screening of enzymatic methylation of 3-methyl-4-phenylpyrazole

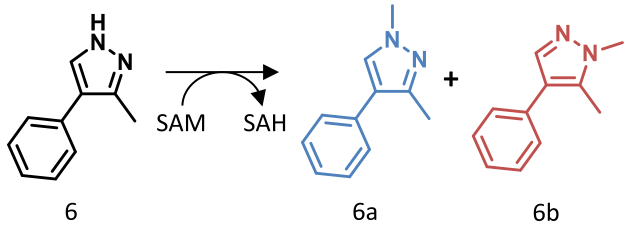

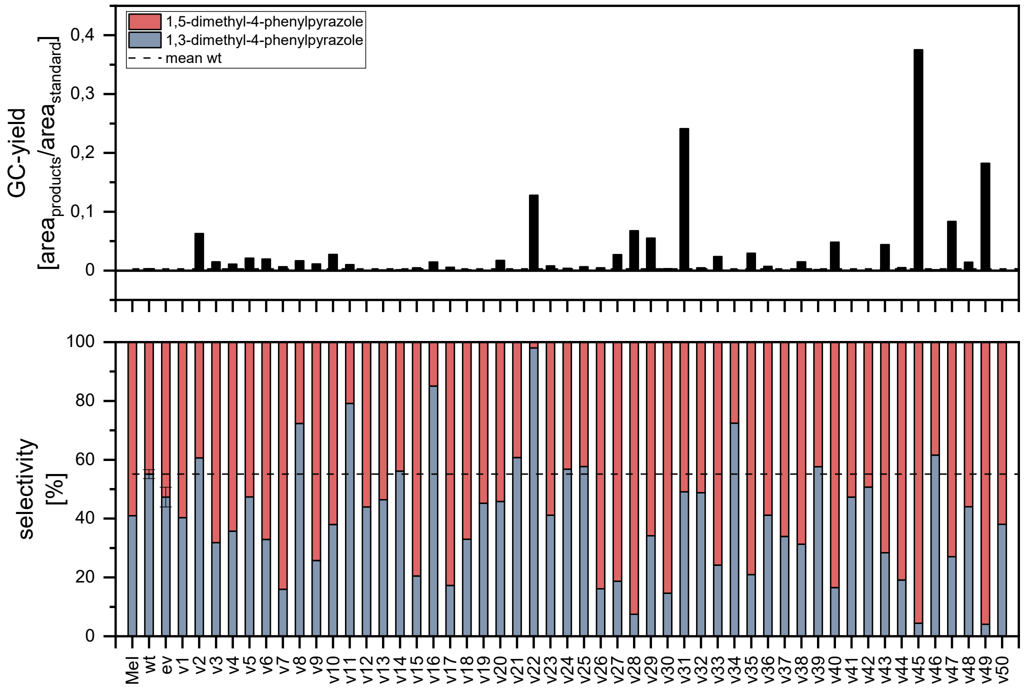

The screening of the substrate 3-methyl-4-phenylpyrazole (**6**) was performed using 400 µl lysate, and 2 mM substrate and SAM respectively at 37°C and 20 h according to paragraph **II (H)**. The upper graph shows the total GC-yield of the reactions (black bars) and the mean wtNMT-yield (dashed line). The underneath graph shows the selectivity of the enzymes, with the selectivity of chemical methylation with methyl iodide (MeI) as reference and the empty vector (ev) as a control. The mean wt selectivity is shown in dashed lines. Error bars for wt show standard deviation of triplicates.

Figure S14: SDS-PAGEs of purified wtNMT, selected FuncLib variants and NSA-synthase

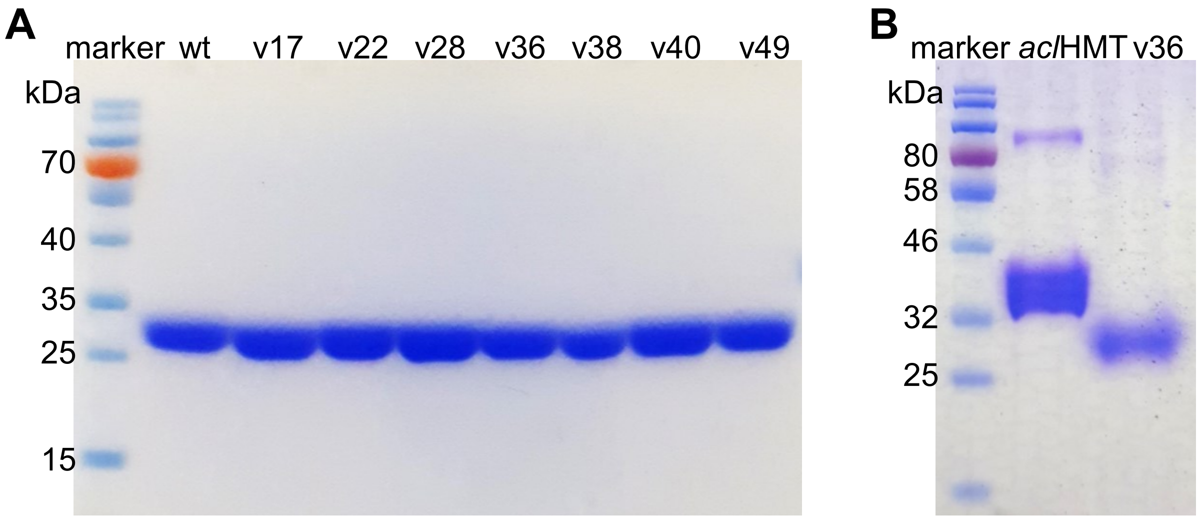

The SDS-PAGEs show the purified wtNMT, selected FuncLib variants and NSA-synthase (*acl*HMT). **A)** SDS-PAGE of wtNMT and selected FuncLib variants. 20 µg of purified enzyme with 6x SDS loading buffer was loaded to an 12% SDS-PAGE and run with 140 V for 1 h. **B)** SDS-PAGE of NSA-synthase and v36 used in the cyclic cascade. 20 µg of purified enzyme with 4x SDS loading buffer was loaded to an 12% SDS-PAGE and run with 120 V for 1 h.

Figure S15: Selectivity analysis of the enzymatic methylation of 3-methylpyrazole

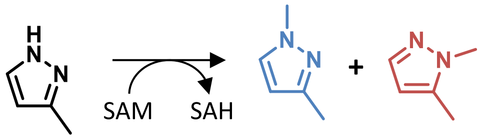

1 1a 1b

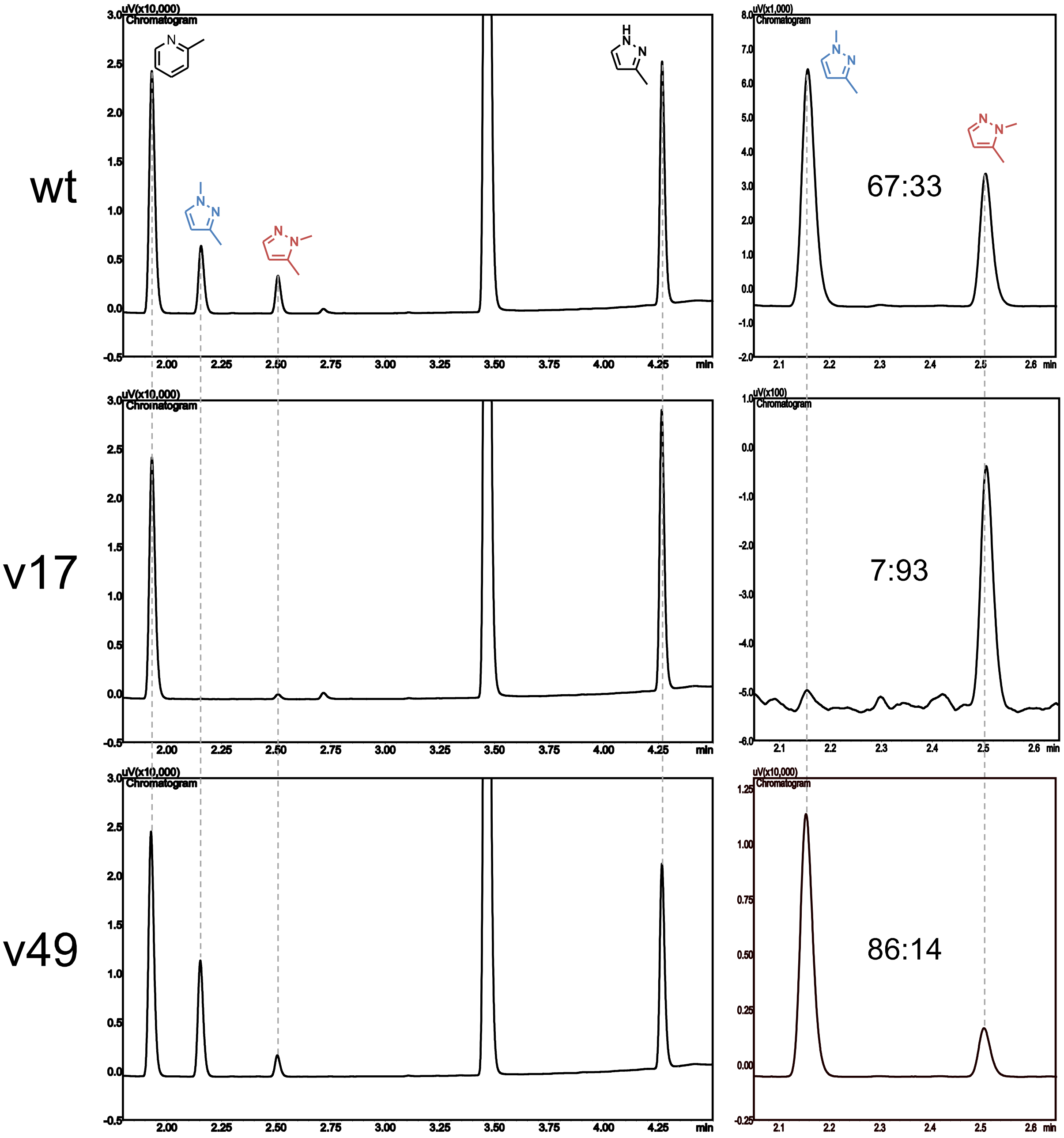

The GC chromatograms show the enzymatic methylation of 3-methylpyrazole **(1)** with wtNMT (top) and FuncLib variants v17 (middle) and v49 (bottom). The reactions were performed using 2 mM substrate, 4 mM SAM and 50 µM enzyme at 37°C and 20 h as described in detail in paragraph **II.(I)**. Chromatograms in the left row show the internal standard 2-methylpyridine, methylated products, DMSO (ret. time 3.455 min) and the substrate. Chromatograms in the right row are zoomed into the product peaks for better visualization of product selectivities.

Figure S16: Selectivity analysis of the enzymatic methylation of 3,4-dimethylpyrazole

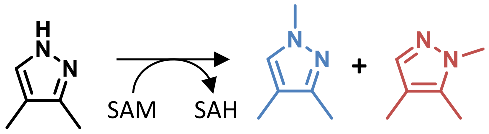

2 2a 2b

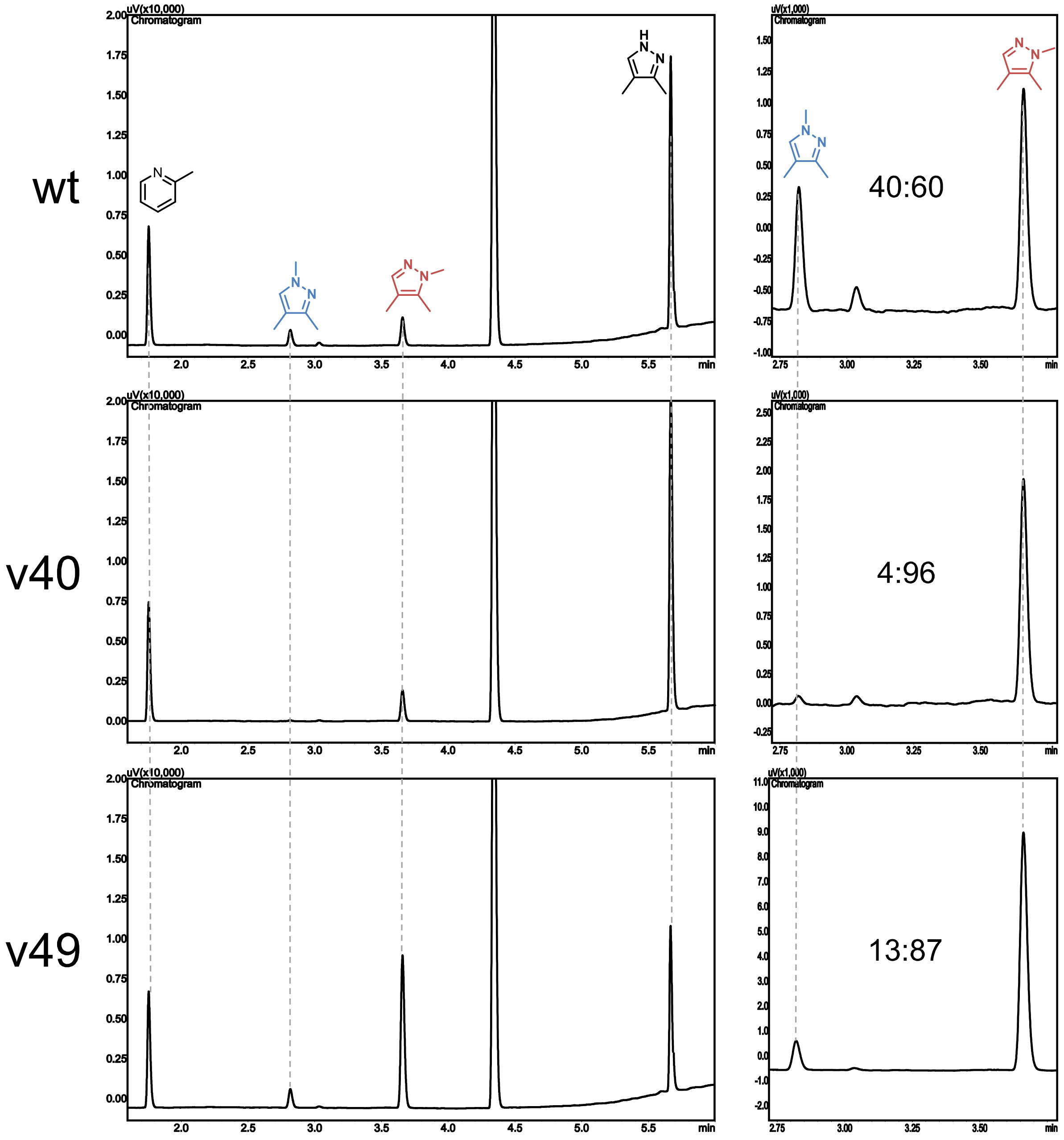

The GC chromatograms show the enzymatic methylation of 3,4-dimethylpyrazole **(2)** with wtNMT (top) and FuncLib variants v40 (middle) and v49 (bottom). The reactions were performed using 2 mM substrate, 4 mM SAM and 50 µM enzyme at 37°C and 20 h as described in detail in paragraph **II.(I)**. Chromatograms in the left row show the internal standard 2-methylpyridine, methylated products, DMSO (ret time 4.35 min) and the substrate. Chromatograms in the right row are zoomed into the product peaks for better visualization of product selectivities.

Figure S17: Selectivity analysis of the enzymatic methylation of 3-cyclopropylpyrazole

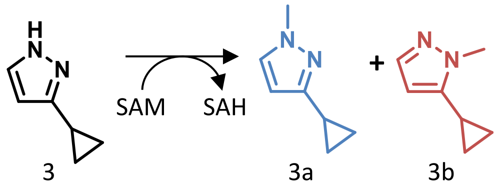

3 3a 3b

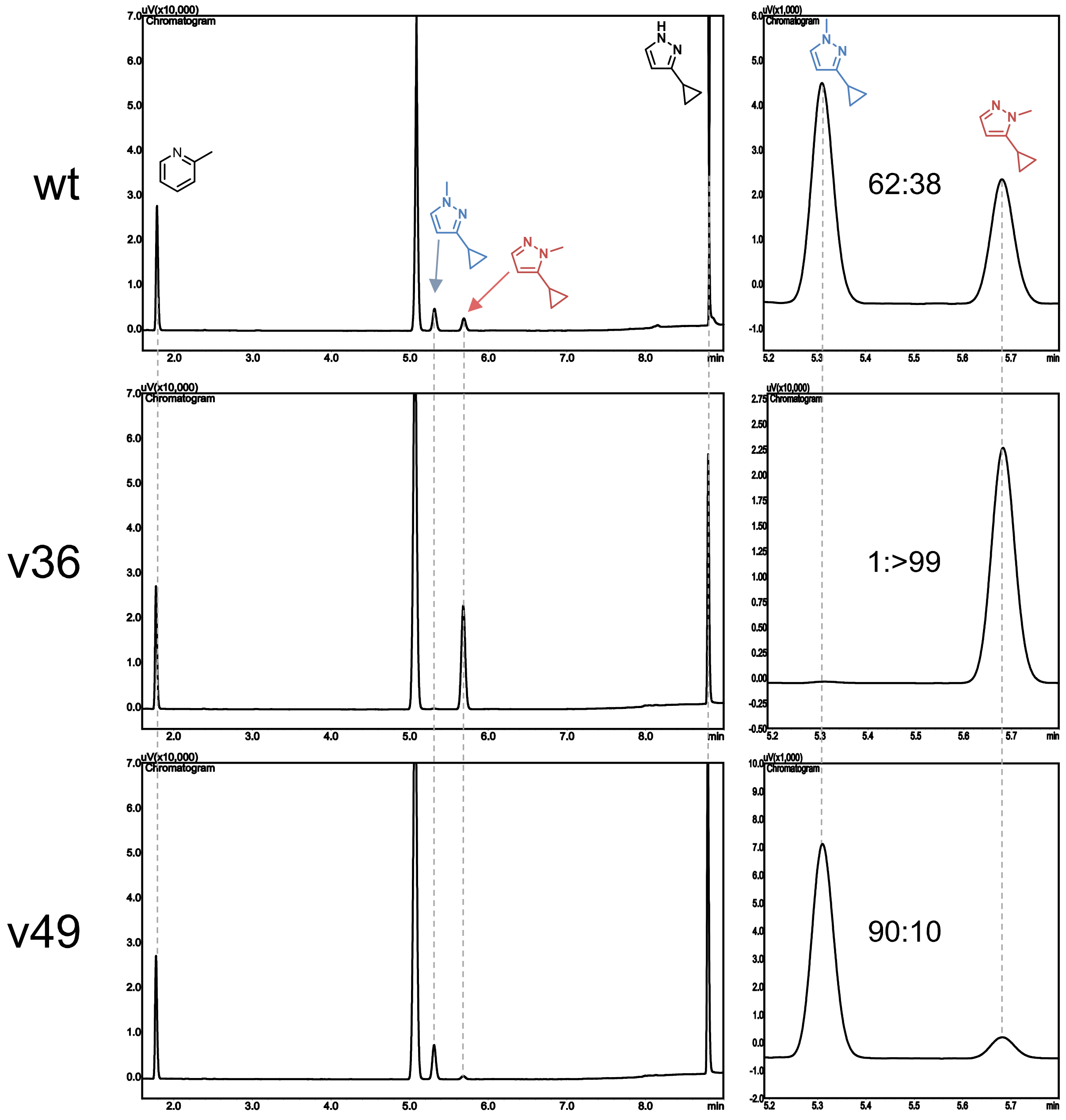

The GC chromatograms show the enzymatic methylation of 3-cyclopropylpyrazole **(3)** with wtNMT (top) and FuncLib variants v36 (middle) and v49 (bottom). The reactions were performed using 2 mM substrate, 4 mM SAM and 50 µM enzyme at 37°C and 20 h as described in detail in paragraph **II.(I)**. Chromatograms in the left row show the internal standard 2-methylpyridine, methylated products, DMSO (ret. time 5.1 min) and the substrate. Chromatograms in the right row are zoomed into the product peaks for better visualization of product selectivities.

Figure S18: Selectivity analysis of the enzymatic methylation of 3(2-furyl)pyrazole

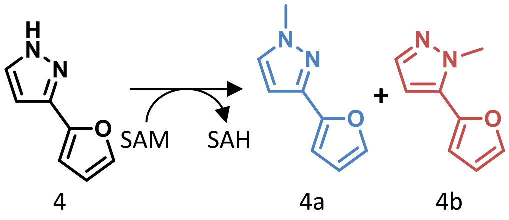

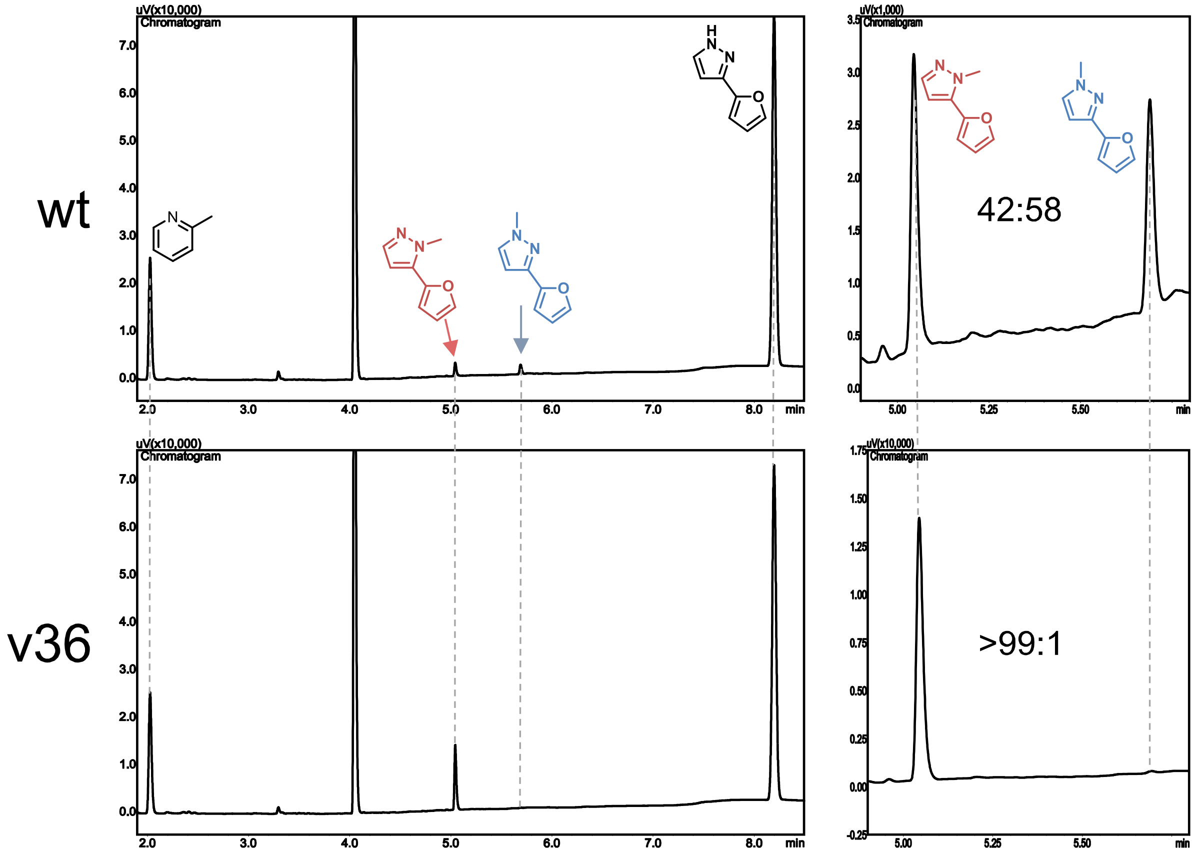

The GC chromatograms show the enzymatic methylation of 3(2-furyl)pyrazole **(4)** with wtNMT (top) and FuncLib variant v36 (bottom). The reactions were performed using 2 mM substrate, 4 mM SAM and 50 µM enzyme at 37°C and 20 h as described in detail in paragraph **II.(I)**. Chromatograms in the left row show the internal standard 2-methylpyridine, methylated products, DMSO (ret. time 4.05 min) and the substrate. Chromatograms in the right row are zoomed into the product peaks for better visualization of product selectivities.

Figure S19: Selectivity analysis of the enzymatic methylation of 3(2-fluorophenyl)pyrazole

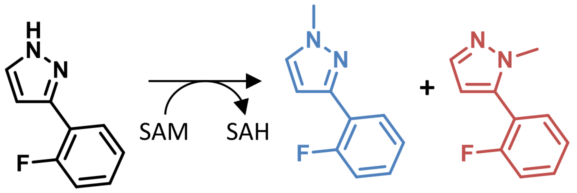

5 5a 5b

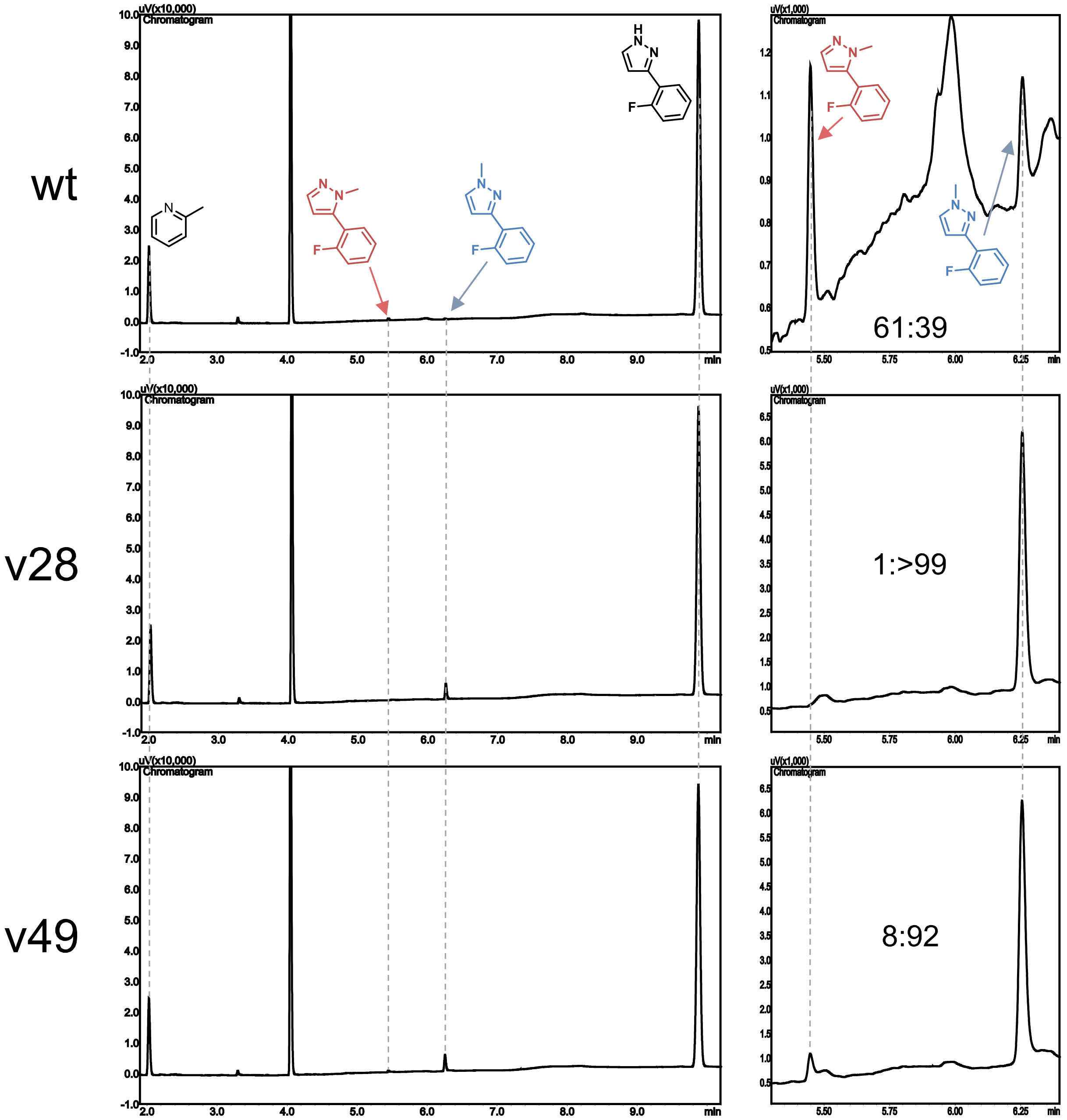

The GC chromatograms show the enzymatic methylation of 3(2-fluorophenyl)pyrazole **(5)** with wtNMT (top) and FuncLib variants v28 (middle) and v49 (bottom). The reactions were performed using 2 mM substrate, 4 mM SAM and 50 µM enzyme at 37°C and 20 h as described in detail in paragraph **II.(I)**. Chromatograms in the left row show the internal standard 2-methylpyridine, methylated products, DMSO (ret. time 4.05 min) and the substrate. Chromatograms in the right row are zoomed into the product peaks for better visualization of product selectivities.

Figure S20: Selectivity analysis of the enzymatic methylation of 3-methyl-4-phenylpyrazole

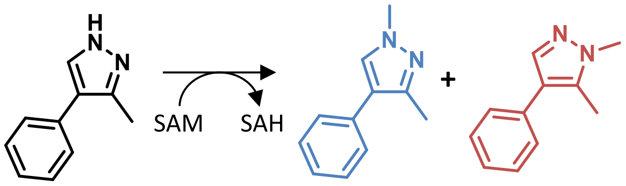

6 6a 6b

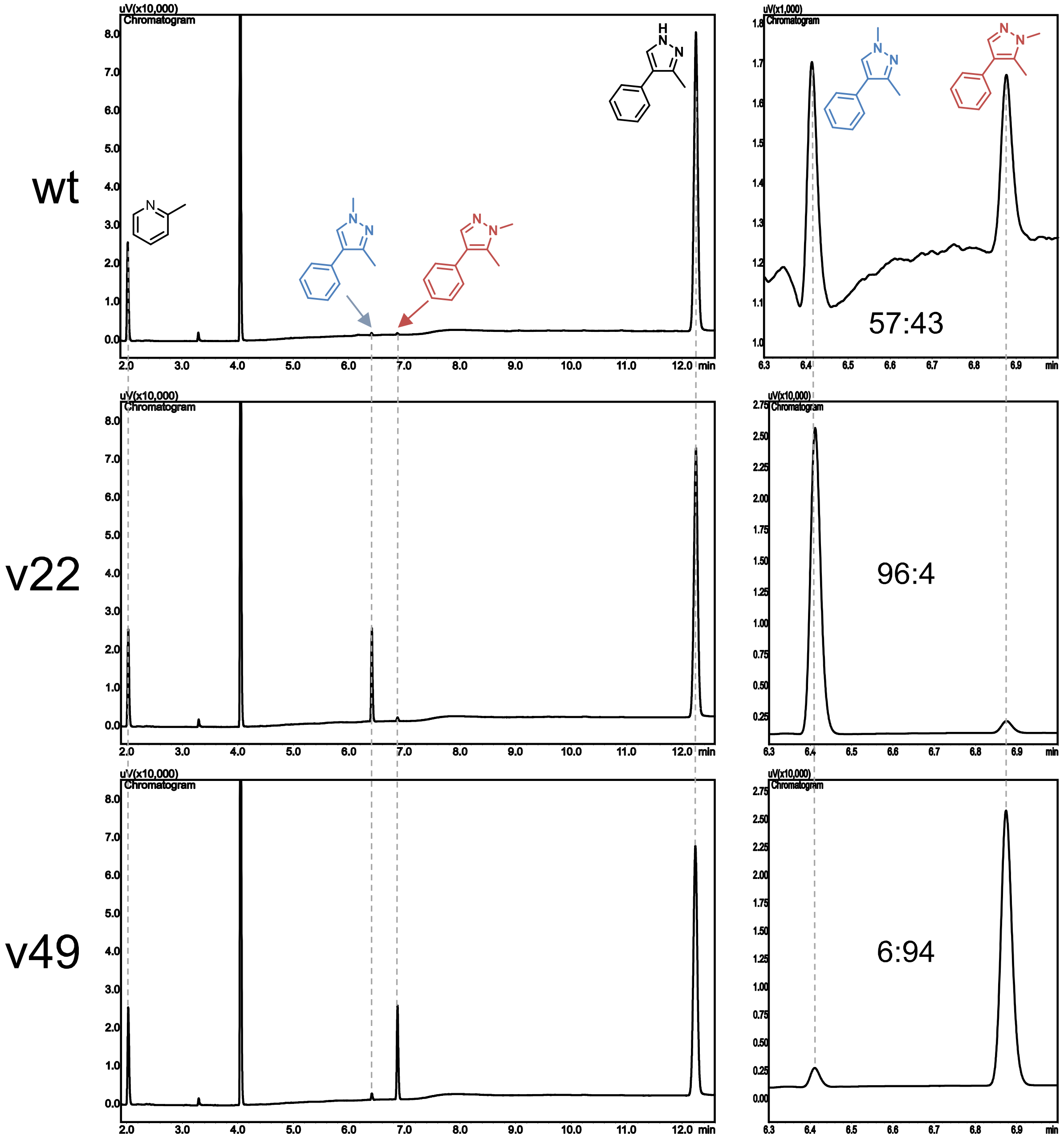

The GC chromatograms show the enzymatic methylation of 3-methyl-4-phenylpyrazole **(6)** with wtNMT (top) and FuncLib variants v22 (middle) and v49 (bottom). The reactions were performed using 2 mM substrate, 4 mM SAM and 50 µM enzyme at 37°C and 20 h as described in detail in paragraph **II.(I)**. Chromatograms in the left row show the internal standard 2-methylpyridine, methylated products, DMSO (ret. time 4.05 min) and the substrate. Chromatograms in the right row are zoomed into the product peaks for better visualization of product selectivities.

Figure S21: Initial rates for the enzymatic methylation of 3-methylpyrazole (**1**)

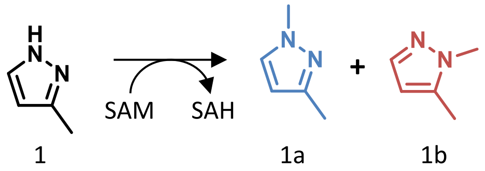

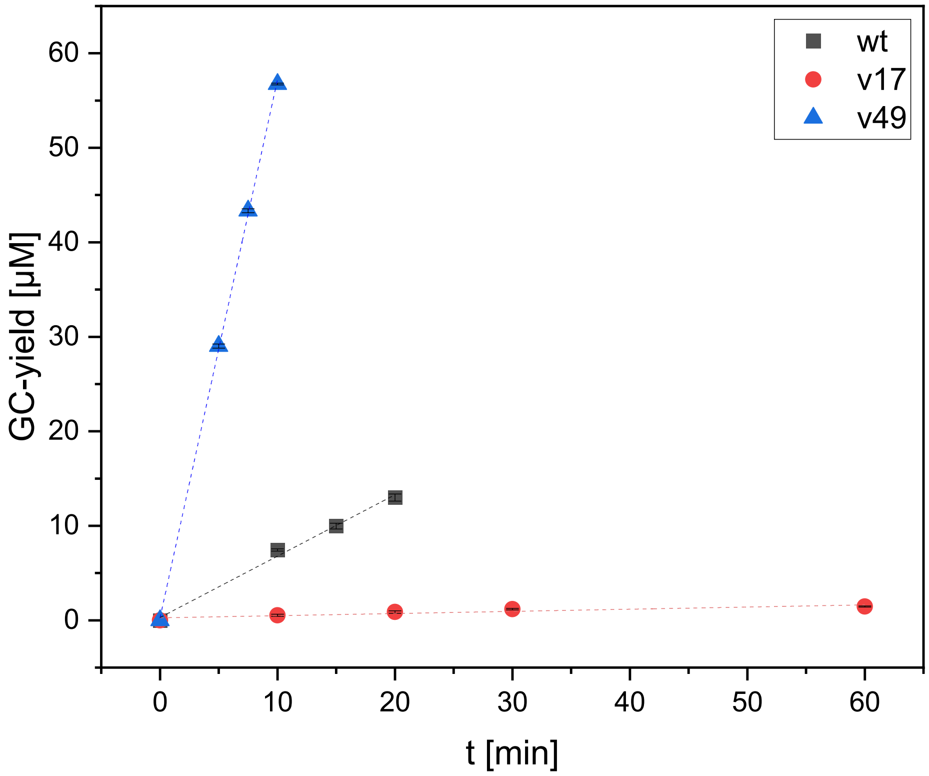

Measurements of initial rates were conducted using 2 mM of each, the pyrazole and SAM and 50 µM enzyme at 37°C as described in detail in paragraph **II.(J)**. The initial rate of wtNMT (grey) was calculated as a slope of 0.65 µM/min with an R^2 of 0.994. The slope of the initial rate of v17 (red) was calculated as 0.039 µM/min with an R^2 of 0.98 that reveals an increase factor of 0.06 to the wt. The slope of the initial rate of v49 (blue) was calculated as 5.69 with an R^2 of 0.999 µM/min that reveals an increase factor of 8.78 to the wt.

Figure S22: Initial rates for the enzymatic methylation of 3,4-methylpyrazole (**2**)

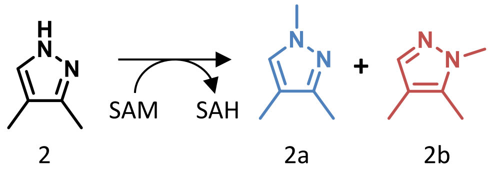

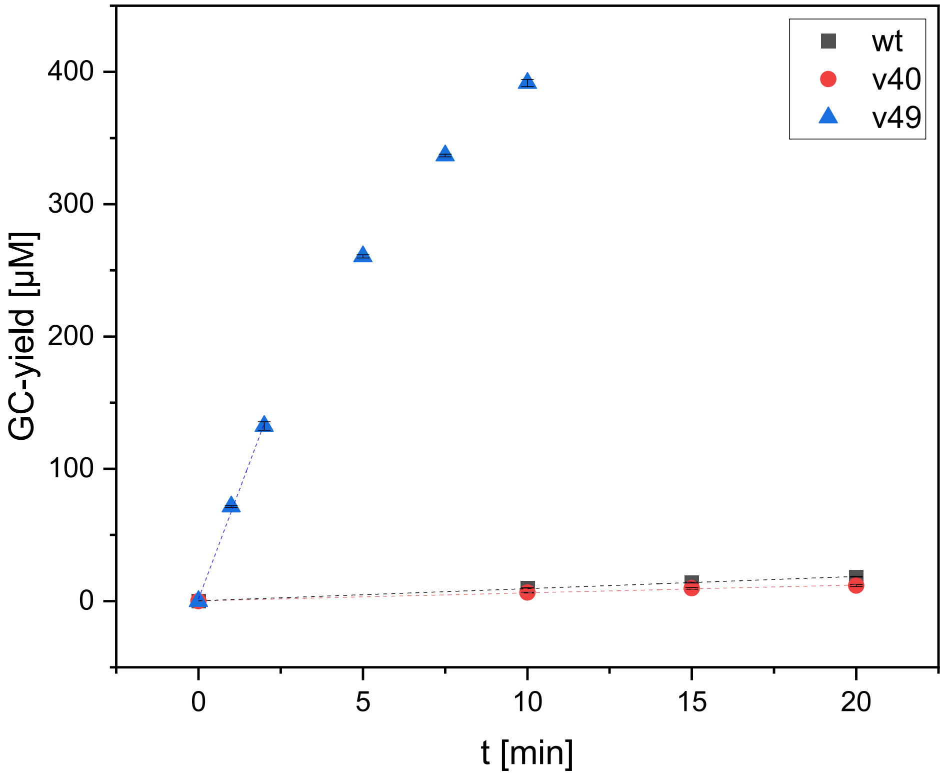

Measurements of initial rates were conducted using 2 mM of each, the pyrazole and SAM and 50 μM enzyme at 37°C as described in detail in paragraph **II.(J)**. The initial rate of wtNMT (grey) was calculated as a slope of 0.92 μM/min with an R^2 of 0.998. The slope of the initial rate of v40 (red) was calculated as 0.599 μM/min with an R^2 of 0.993 that reveals an increase factor of 0.65 to the wt. The slope of the initial rate of v49 (blue) was calculated as 66.2 μM/min with an R^2 of 0.998 that reveals an increase factor of 71.9 to the wt.

Figure S23: Initial rates for the enzymatic methylation of 3-cyclopropylpyrazole (**3**)

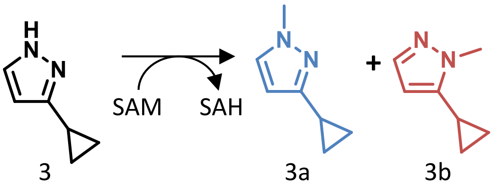

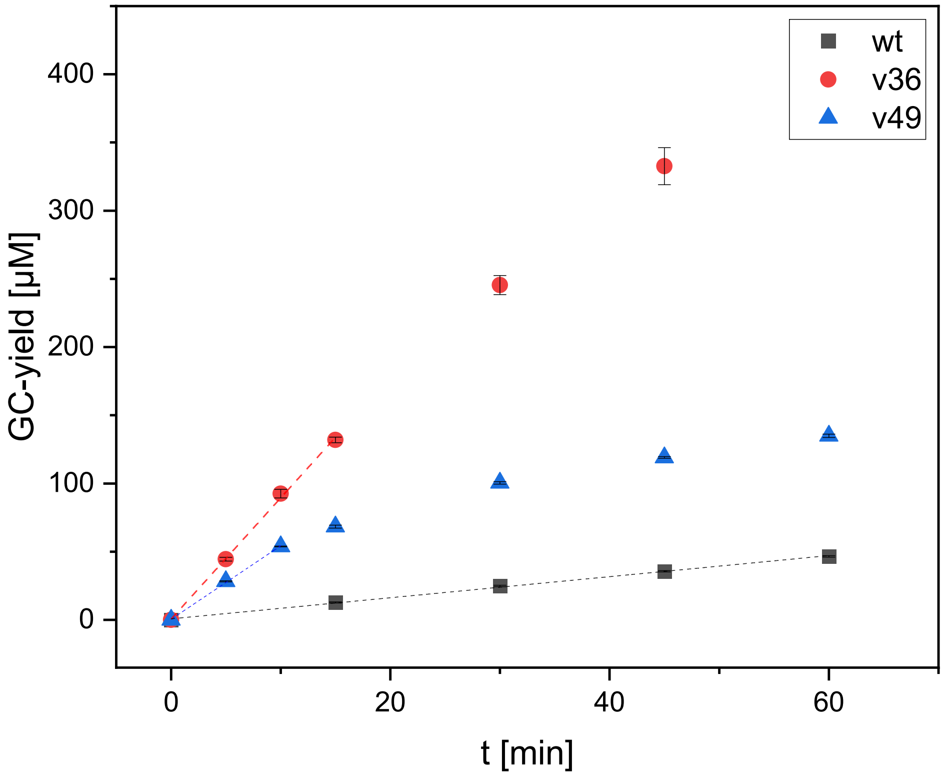

Measurements of initial rates were conducted using 2 mM of each, the pyrazole and SAM and 50 µM enzyme at 37°C as described in detail in paragraph **II.(J)**. The initial rate of wtNMT (grey) was calculated as a slope of 0.77 µM/min with an R^2 of 0.999. The slope of the initial rate of v36 (red) was calculated as 8.87 µM/min with an R^2 of 0.999 that reveals an increase factor of 11.5 to the wt. The slope of the initial rate of v49 (blue) was calculated as 5.39 µM/min with an R^2 of 0.999 that reveals an increase factor of 7.0 to the wt.

Figure S24: Initial rates for the enzymatic methylation of 3(2-furyl)pyrazole (**4**)

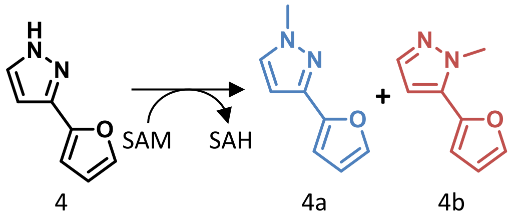

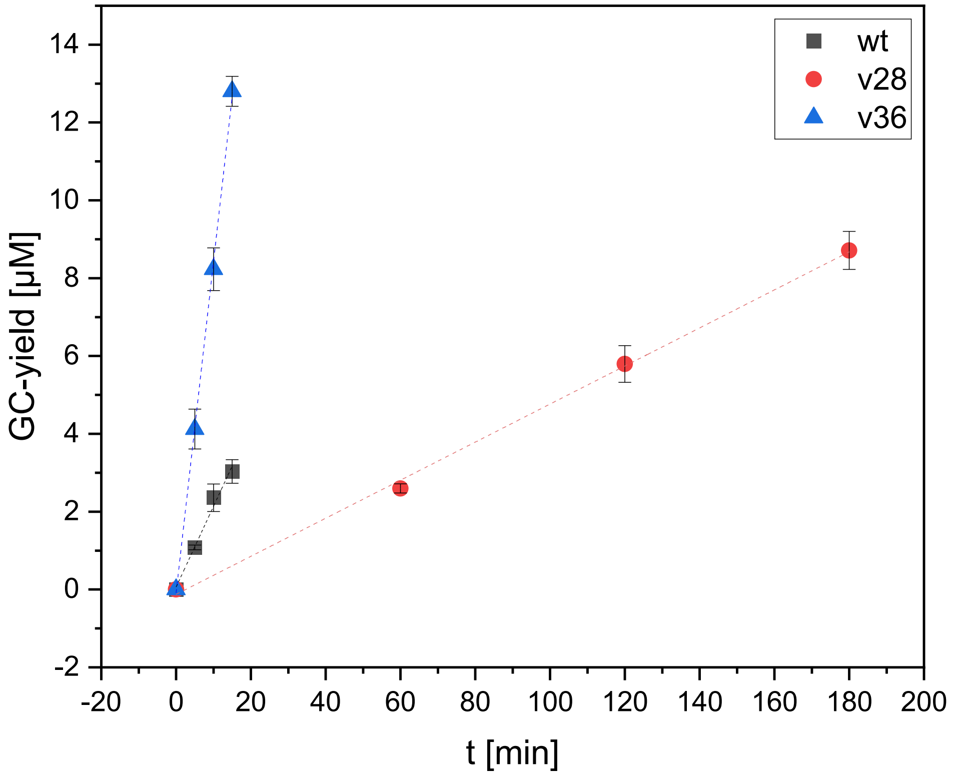

Measurements of initial rates were conducted using 2 mM of each, the pyrazole and SAM and 50 µM enzyme at 37°C as described in detail in paragraph **II.(J)**. The initial rate of wtNMT (grey) was calculated as a slope of 0.21 µM/min with an R^2 of 0.987. The slope of the initial rate of v28 (red) was calculated as 0.049 µM/min with an R^2 of 0.999 that reveals an increase factor of 0.24 to the wt. The slope of the initial rate of v36 (blue) was calculated as 0.85 µM/min with an R^2 of 0.999 that reveals an increase factor of 4.1 to the wt.

Figure S25: Initial rates for the enzymatic methylation of 3(2-fluorophenyl)pyrazole (**5**)

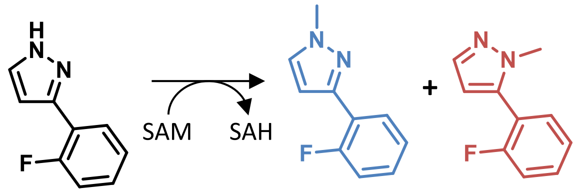

5 5a 5b

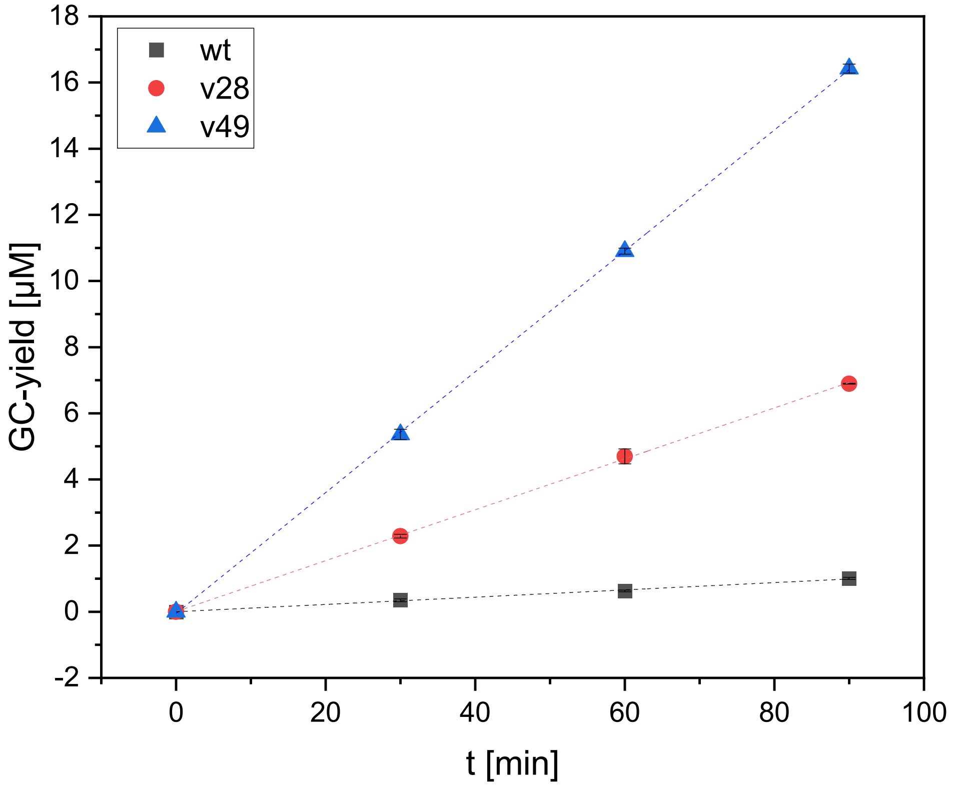

Measurements of initial rates were conducted using 2 mM of each, the pyrazole and SAM and 50 µM enzyme at 37°C as described in detail in paragraph **II.(J)**. The initial rate of wtNMT (grey) was calculated as a slope of 0.011 µM/min with an R^2 of 0.996. The slope of the initial rate of v28 (red) was calculated as 0.077 µM/min with an R^2 of 0.999 that reveals an increase factor of 6.89 to the wt. The slope of the initial rate of v49 (blue) was calculated as 0.18 µM/min with an R^2 of 0.999 that reveals an increase factor of 16.3 to the wt.

Figure S26: Initial rates for the enzymatic methylation of 3-methyl-4-phenylpyrazole (**6**)

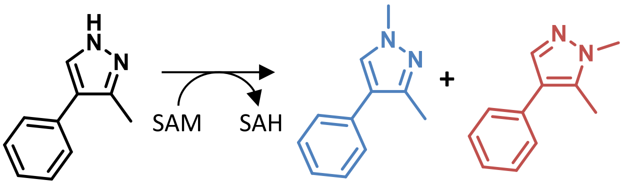

6 6a 6b

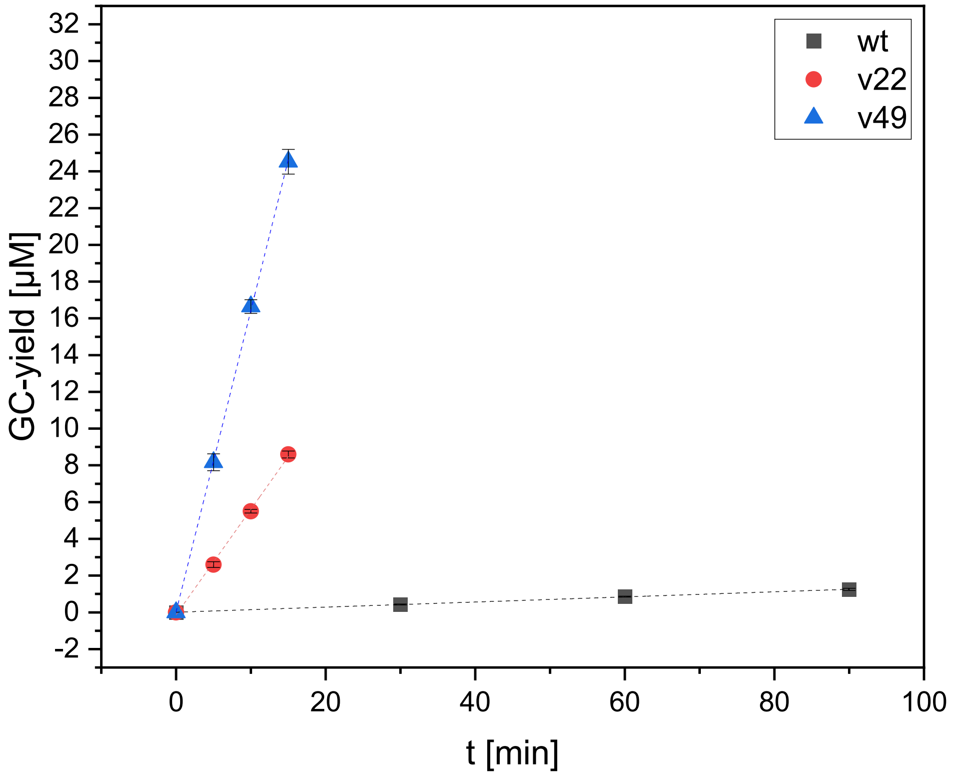

Measurements of initial rates were conducted using 2 mM of each, the pyrazole and SAM and 50 µM enzyme at 37°C as described in detail in paragraph **II.(J)**. The initial rate of wtNMT (grey) was calculated as a slope of 0.014 µM/min with an R^2 of 0.999. The slope of the initial rate of v22 (red) was calculated as 0.57 µM/min with an R^2 of 0.999 that reveals an increase factor of 41.3 to the wt. The slope of the initial rate of v49 (blue) was calculated as 1.64 µM/min with an R^2 of 0.999 that reveals an increase factor of 118 to the wt.

Figure S27: Enzymatic methylation of pyrazoles: GC-yields and initial rates

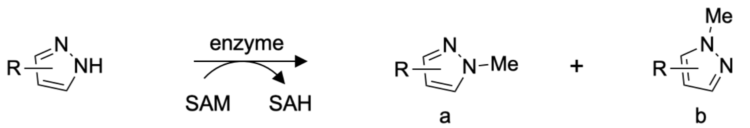

Substrate	Variant	Conversion in %	Activity in µmol/min	Activity increase[a]	Selectivity (a:b)
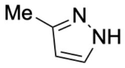 1	NMT wild-type	14	0.64		67 : 33
	v17	0.65	0.03	0.05	7 : 93
	v49	19	5.7	8.9	86 : 13
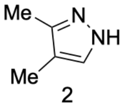 2	NMT wild-type	13	0.92		40 : 60
	v40	9.1	0.59	0.65	4 : 96
	v49	49	66	72	13 : 87
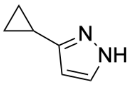 3	NMT wild-type	10	0.78		62 : 38
	v36	34	8.9	11	1 : >99
	v49	11	5.4	7.0	90 : 10
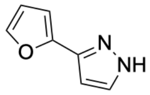 4	NMT wild-type	6.6	0.22		42 : 58
	v36	18	0.85	3.8	1 : >99
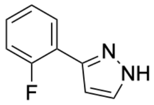 5	NMT wild-type	0.39	0.01		39 : 61
	v28	2.4	0.08	7.0	>99 : 1
	v49	2.7	0.18	17	92 : 8
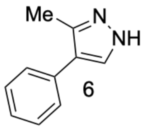 6	NMT wild-type	0.49	0.014		57 : 43
	v22	11	0.57	41	96 : 4
	v49	11	1.6	118	6 : 94

This figure supports Fig. 3A in the main paper with the addition of percentage conversion. Please note that in our opinion percentage conversion is not a good indicator to validate the performance of the variants. In this experimental setup SAM has been used as stoichiometric reagent and SAH is generated as stoichiometric byproduct. It is, however, known that SAH inhibits methyltransferases in general and NMT in particular.[19] For this reason, initial rate determination at very low SAH byproduct concentration is more insightful than percentage conversion to compare the described variants. Initial rates were analyzed using 2 mM pyrazole, 2 mM SAM and 50 µM enzyme at 37°C. Percentage conversion for each reaction was analyzed using 2 mM pyrazole, 4 mM SAM and 50 µM enzyme at 37 °C for 20 h. All enzymatic reactions were performed in triplicates. Please note that all chemical methylations as well as the reactions with the wild-type NMT generate product mixtures (see Fig. S1 and S15-S20). [a] Activity increase is calculated by dividing the initial rate of the variant by the initial rate of NMT wild-type.

Figure S28: Substrate scope screening of the HMTs

	MeI (80 equiv.)	EtI (60 equiv.)	EtBr (70 equiv.)	PropI (50 equiv.)	ButI (40 equiv.)	ButBr (50 equiv.)	ButCl (50 equiv.)
				conversion [%]			
buffer	1	0	0	0	0	0	0
empty vector	10	0	0	0	0	0	0
*cth*HMT	97	1	0	0	1	0	0
*ath*HMT	100	44	1	0	0	0	0
*acl*HMT	100	78	99	31	3	2	1
*bma*HMT	99	81	100	1	0	0	0
*bxe*HMT	99	2	2	0	0	0	0
*sel*HMT	100	61	21	3	1	0	0

The 500 µl biotransformations of the screening of HMTs were performed with 245 µl lysate, 1 mM SAH and 40-80 equivalents of haloalkanes at 25°C for 20 h as described in paragraph **II.(K)**. The product formation was analyzed by HPLC (**I.(E)**). The tested HMTs are NSA-synthase *Aspergillus clavatus* halide methyl transferase *(acl*HMT, UniProtKB: A1CIS5), *Chloracidobacterium thermophilum* HMT (*cth*HMT, UniProtKB: G2LF24), *Arabidopsis thaliana* HMT (*ath*HMT, UniProtKB: Q6AWU6), *Batis maritima* HMT (*bma*HMT, UniProtKB: Q7ZSZ7), *Burkholderia xenovorans* (*bxe*HMT, NCBI: WP_011486779) and *Synechococcus elongatus* (*sel*HMT, UniProtKB: Q31S13). Please note that each haloalkane substrate was tested in independent experiments to examine the promiscuity of the selected enzymes, therefore conversion of different haloalkanes is not directly comparable. All screened enzymes showed full conversion with iodomethane (MeI) and only little non-enzymatic reaction was observed. For iodoethane (EtI) and bromoethane (EtBr), only 4 of the tested enzymes reached medium to good conversions. Interestingly, the previous described *cth*HMT showed lowest or no conversions for all haloalkanes with longer alkyl-chains than methyl. The only enzyme that was active for all examined haloalkanes was *acl*HMT, that we further named NSA-synthase. No non-enzymatic reactions occurred for haloalkanes with longer alkyl-chains than methyl.

Figure S29: NSA-synthase catalyzed SAH alkylation

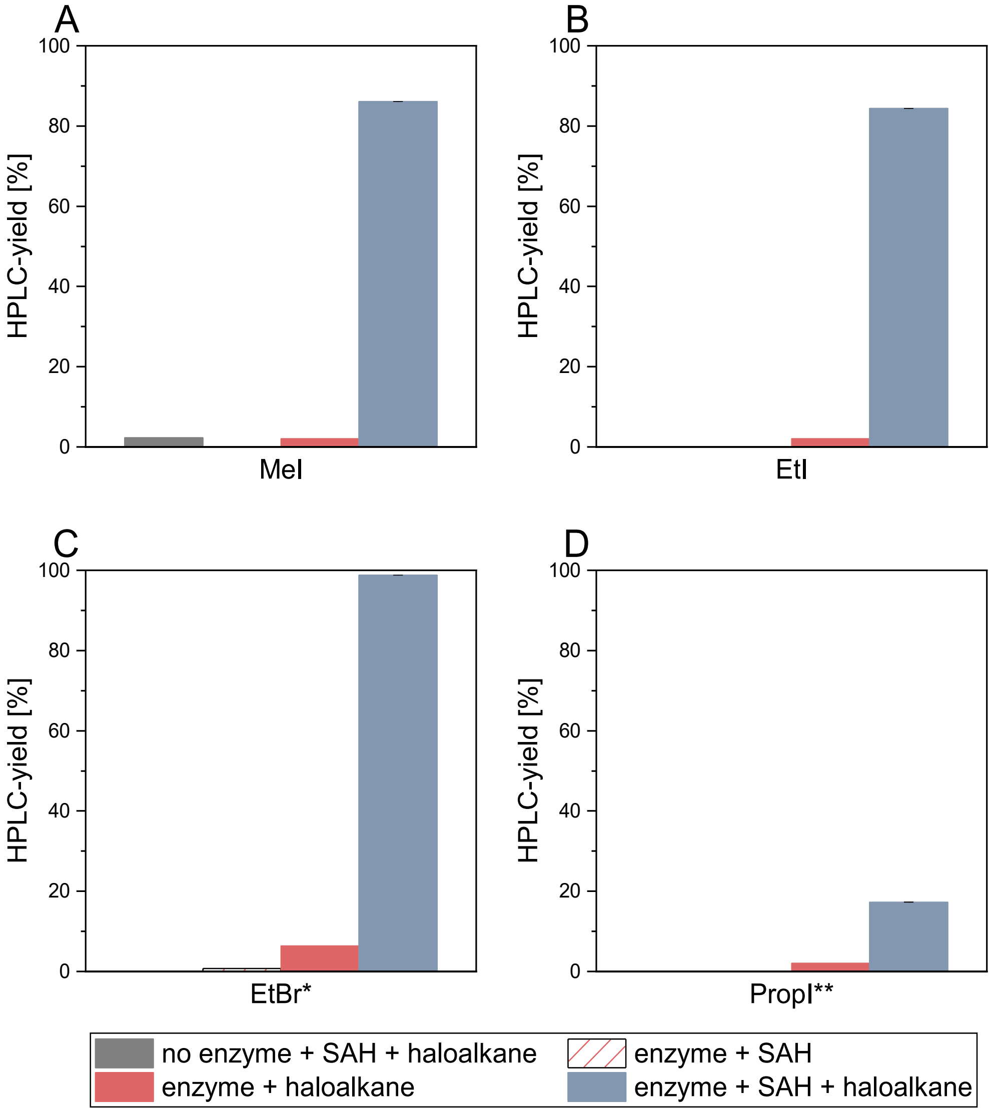

The NSA-synthase catalyzed SAH alkylation and control reactions with **A)** iodomethane (MeI) and **B)** iodoethane (EtI) were proceeded with 1 mM SAH, 10 equivalents of the corresponding haloalkane and 50 µM purified enzyme at 25 °C for 20 h as described in paragraph **II.(K)**. **C)** The reaction with bromoethane (EtBr*) was performed under the same conditions as with MeI and EtI but with 67 equivalents of EtBr in a lysate reaction (245 µl lysate instead of purified enzyme). **D)** The reaction with iodopropane (PropI**) was performed similar to the reactions with MeI and EtI as described in paragraph **II.(K)** but with 50 equivalents of PropI. Error bars show standard deviation of at least duplicates. These individual experiments should support that these reactions are enzyme catalyzed and not a non-enzymatic background. To confirm this, we did control experiments for the reactions with each haloalkane substrate without NSA-synthase, but with the addition of SAH and haloalkane. In addition, reactions with NSA-synthase and SAH showed residual SAM that is only observed when lysate was used in the reactions. Further, we investigated the NSA-synthase only treated with haloalkanes. This control experiment reveals the alkylation potency of the co-purified SAH.

Figure S30: Time course of NSA formation

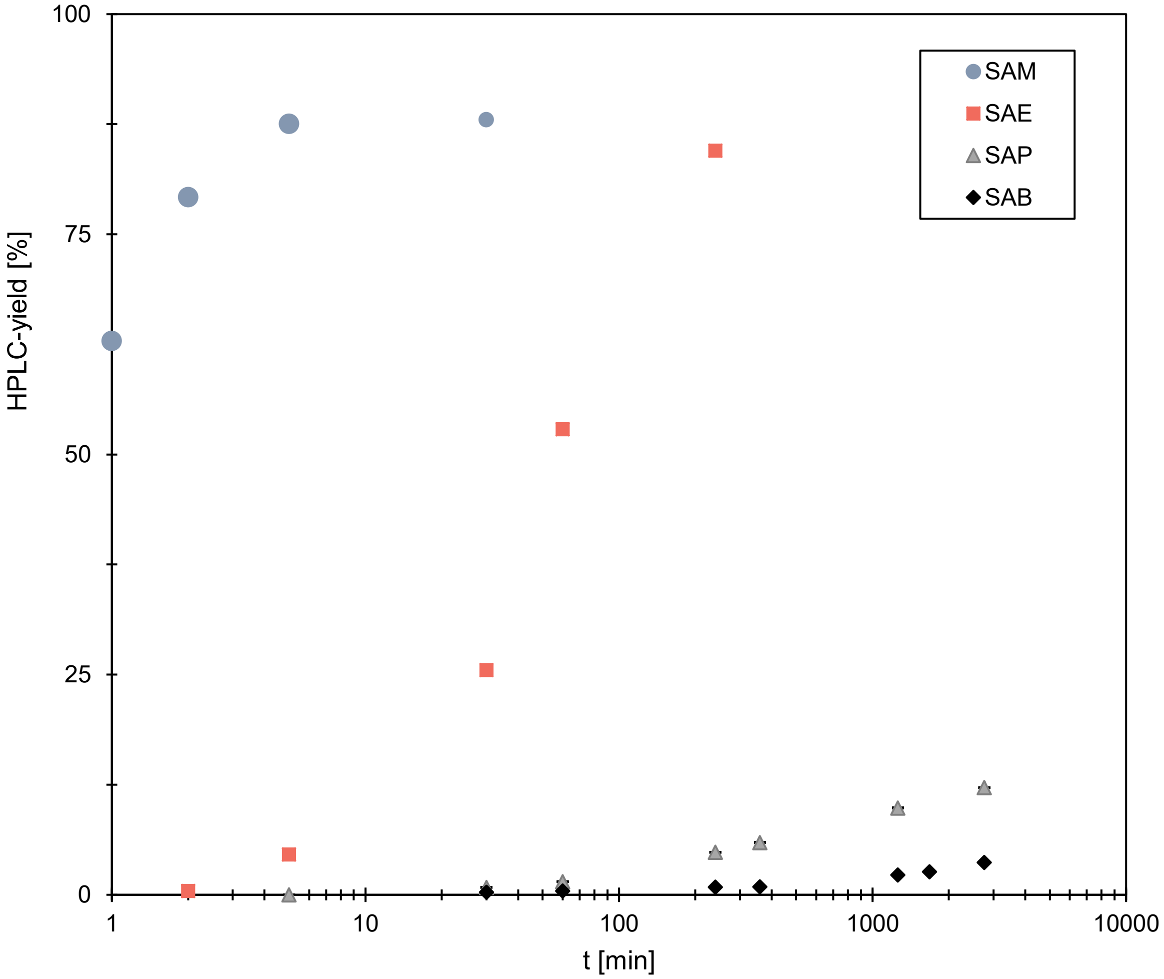

Time course of NSA formation by NSA-synthase. SAM was produced with highest activity with the use of iodomethane, followed by *S*-adenosyl-L-ethionine (SAE, red) with the use of iodoethane and slowest formation of *S*-adenosyl-L-propionine (SAP, grey) and *S*-adenosyl-L-butionine (SAB, black) with the use of iodopropane and iodobutane respectively. 50 µM of NSA-synthase, 1 mM SAH and 10 equivalents of each haloalkane were incubated in 500 µl reactions at 25°C as described in detail in paragraph **II.(K)**. Error bars present standard deviation of triplicates.

Figure S31: Conformation of enzymatically sythesized NSAs by mass spectrometry

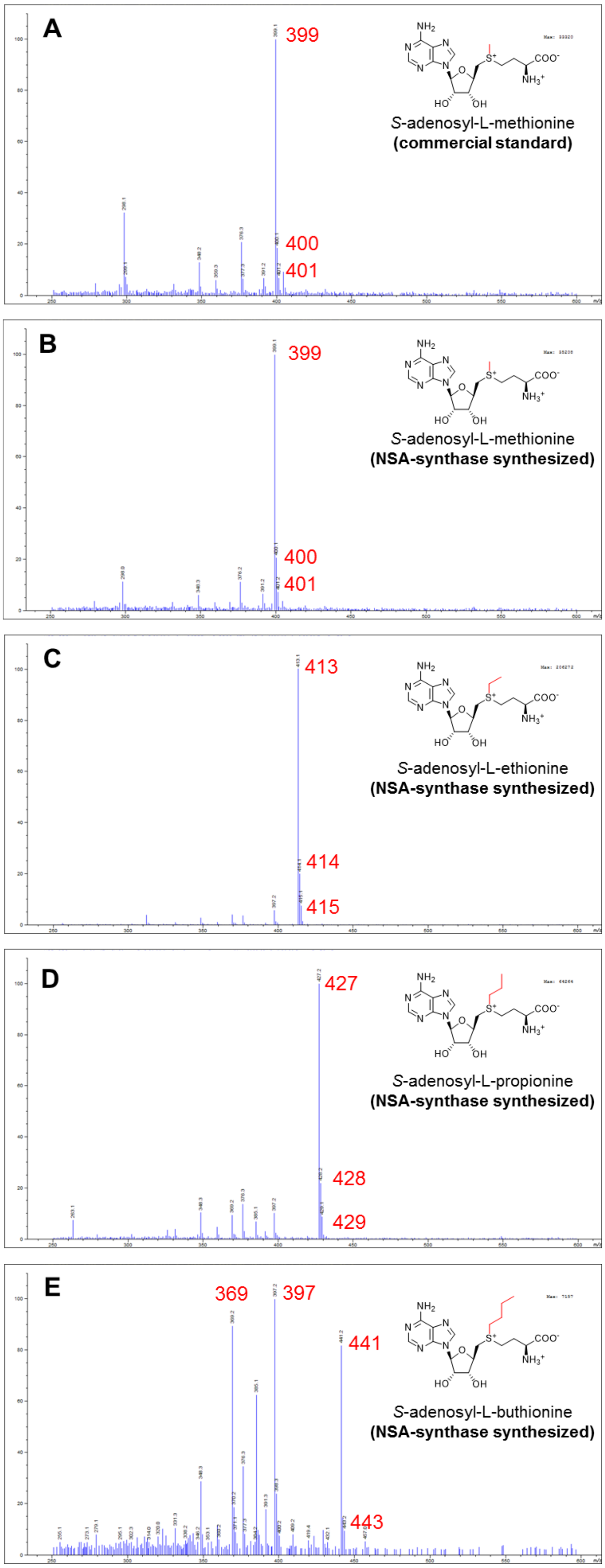

Synthesis of NSAs by NSA-synthase (50 µM NSA-synthase, 1 mM SAH, 10 equivalents of haloalkane, 25°C, 20 h) was confirmed by LC-MS measurements using ESI ionization in positive mode (see paragraph **II.(E/K)**). **A)** Mass spectra of a commercially available *S*-adenosyl-L-methionine standard. The measured main mass is identical to the theoretical mass of 399.14. **B)** Mass spectra of NSA-synthase synthesized SAM. The mass spectra is similar to the one of the authentic standard. **C)** Mass spectra of NSA-synthase synthesized *S*-adenosyl-L-ethionine. The main mass is identical to the theoretical mass of 413.16. **D)** Mass spectra of NSA-synthase synthesized *S*-adenosyl-L-propionine. The main mass is identical to the theoretical mass of 427.18. **E)** Mass spectra of NSA-synthase synthesized *S*-adenosyl-L-buthionine. We can find a mass hit for m/z 441 that is identical to the theoretical mass of 441.19.

Figure S32: Optimization of the cyclic enzmye cascade: SAH-addition

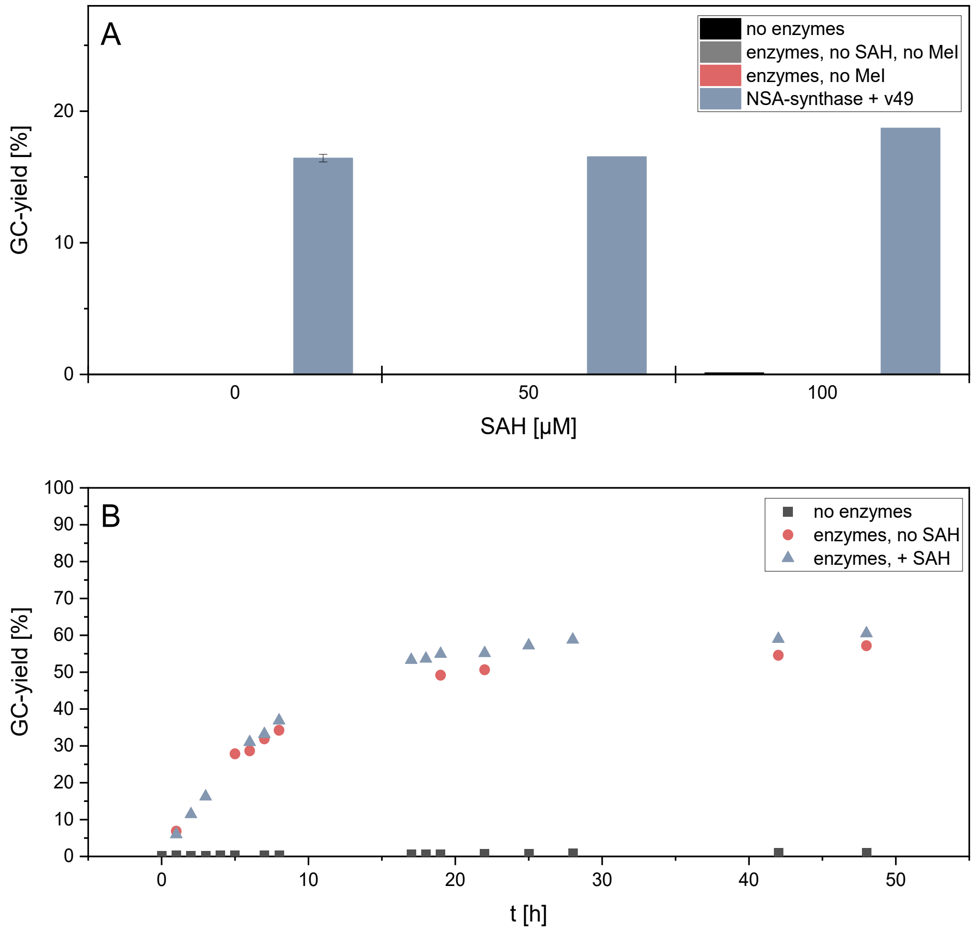

Addition of SAH to the cyclic enzyme cascade reaction of NSA-synthase with FuncLib variants. **A)** Reactions of NSA-synthase and v49 (50 µM each) was performed according to paragraph **II.(L)** at 37°C for 20 h with 2 mM 3-methylpyrazole (**1**) and 10 equivalents of iodmethane as substrates and variation of the SAH concentration. HPLC measurements (data not shown) showed that the purified enzymes contain residual amounts of co-purified SAH. These amounts of SAH correspond equally to the NSA-synthase concentration in the reactions. Error bar shows standard deviation of duplicates. **B)** Time course of NSA-synthase and v36 (50 µM each) with 2 mM 3-cyclopropyl (**3**) and 10 equivalents of iodomethane as substrates performed at 37°C according to paragraph **II.(L)**. As the deviation of the reactions in presence or absence of SAH is less than 2-5%, we performed the cascade reactions without the addition of SAH.

Figure S33: Excess of iodomethane influences the methylation yield

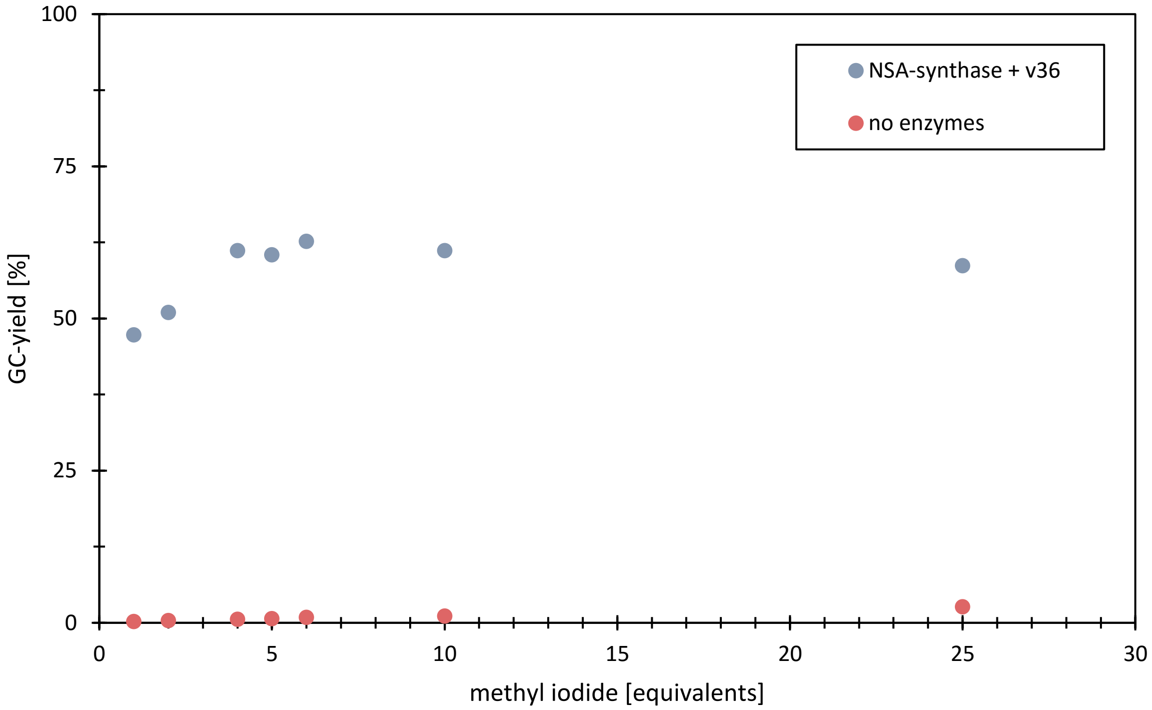

We examined the excess of iodomethane that is necessary to drive the enzyme cascade, with 50 µM of each, NSA-synthase and v36 and 2 mM 3-cyclopropylpyrazole at 37°C for 20 h according to the protocol in paragraph **II.(L)**. Analysis revealed that high amounts of iodomethane (25 equiv. and higher) increases the non-enzymatic background reaction and are not suitable for the enzyme cascade. In between 4 and 10 equivalents, the GC-yield remains constant, leading to around 60% product formation. Equimolar concentrations of iodomethane reduced the product formation only slightly to 47%.

Figure S34: Scale-up of enzymatic methylation of 3-cyclopropylpyrazole (**3**) with iodomethane

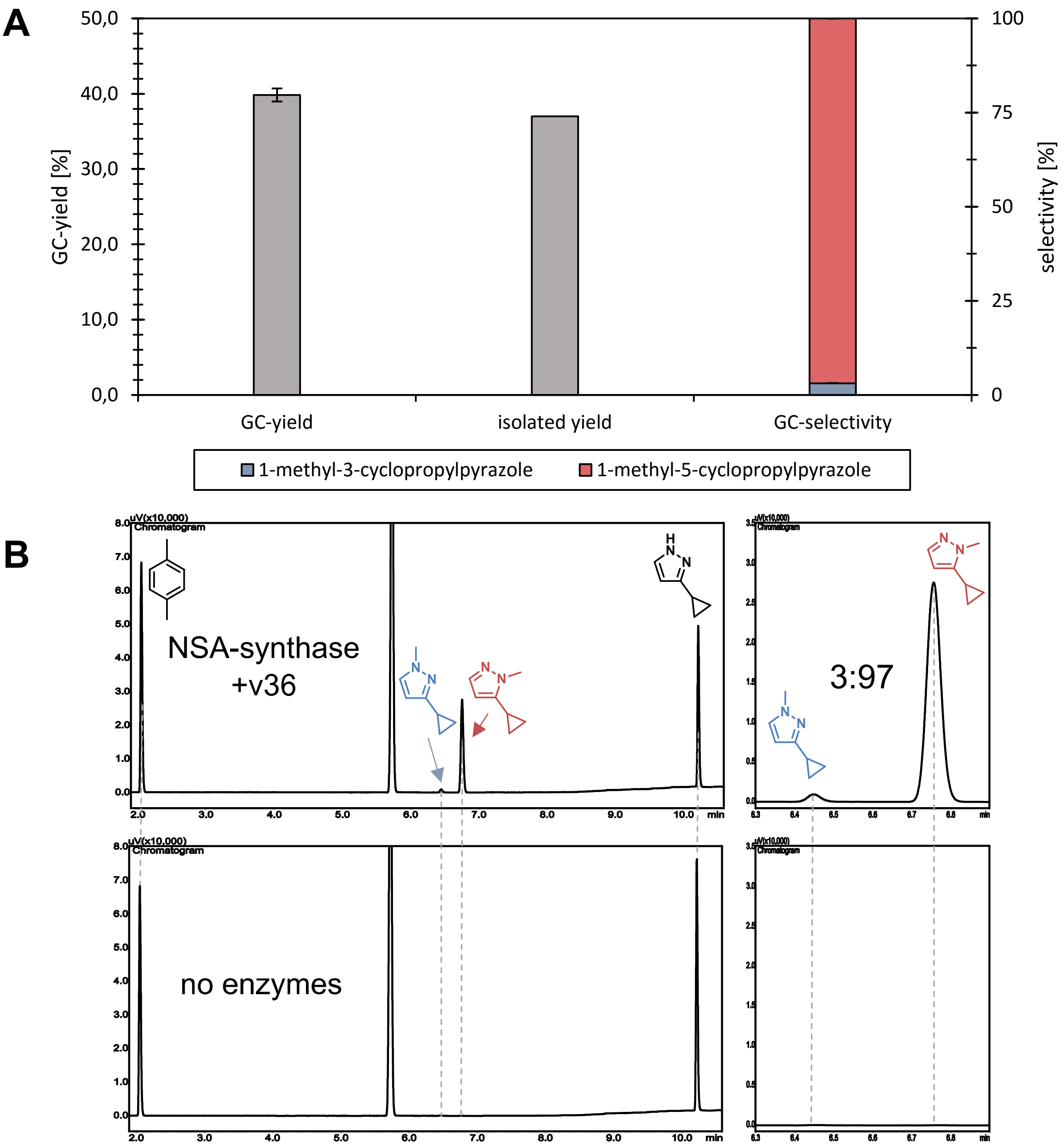

The scale-up of the reaction of NSA-synthase and v36 with 3-cyclopropylpyrazole (**3**) and methyl iodide was proceeded in a 500 ml reaction (x1000 scale-up) with 50 µM of NSA-synthase and v36 respectively, 2 mM 3-cyclopropylpyrazole (**3**) and 10 equivalents of iodomethane at 37°C for 30 h as described in paragraph **II.(M)**. **A)** GC- and isolated yield as well the GC-selectivity. 3% of the product yield was lost during purification of the product. Error bars show standard deviation of triplicates. **B)** GC-FID chromatograms of the up-scaled methylation of 3-cyclopropylpyrazole (**3**). Left row shows the full chromatograms of the enzymatic reaction (top) with internal standard p-xylene, DMSO (ret time 5.7), methylated products and substrate in comparison to the control reaction without enzymes (bottom). Chromatograms in the right row are zoomed into the methylated product peaks for better visualization of the GC-selectivities. Control reaction reveals that no non-enzymatic reaction occurred.

Figure S35: Enzymatic ethylation of 3-cyclopropylpyrazole (**3**) using iodoethane

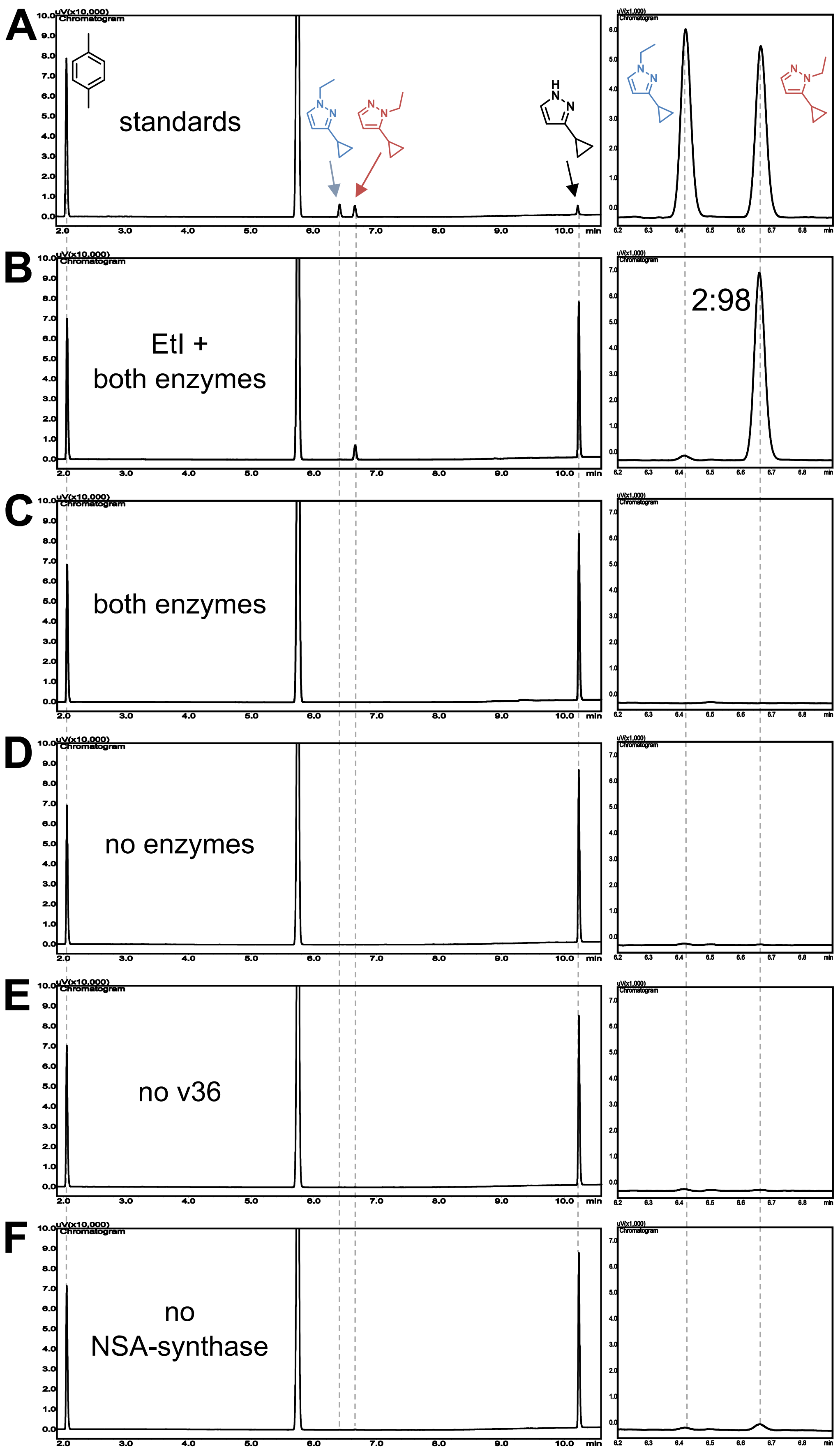

Comparison of GC-FID chromatograms (full chromatograms in the left row, zoom into product peaks in the right row) of (**A**) standard compounds (internal standard p-xylene, ethylated products, 3-cyclopropylpyrazole (**3**), DMSO at ret. time 5.75 min), (**B**) the product of selective enzymatic ethylation, using iodoethane (EtI). (**C**) The control reaction with both enzymes, but without EtI shows no product formation that proves that no methylation occurs. (**D**) The controls with EtI but without both enzymes, (**E**) without v36 and (**F**) without NSA-synthase reveal that the reaction is enzyme catalyzed. The reaction was performed using 50 µM of NSA-synthase and v36 respectively, 2 mM 3-cyclopropylpyrazole and 10 equivalents of iodoethane at 30°C for 72 h.

Figure S36: Enzymatic propylation of 3-cyclopropylpyrazole (**3**) using 1-iodopropane

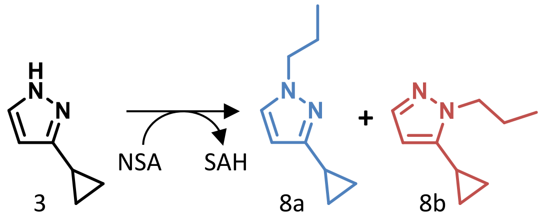

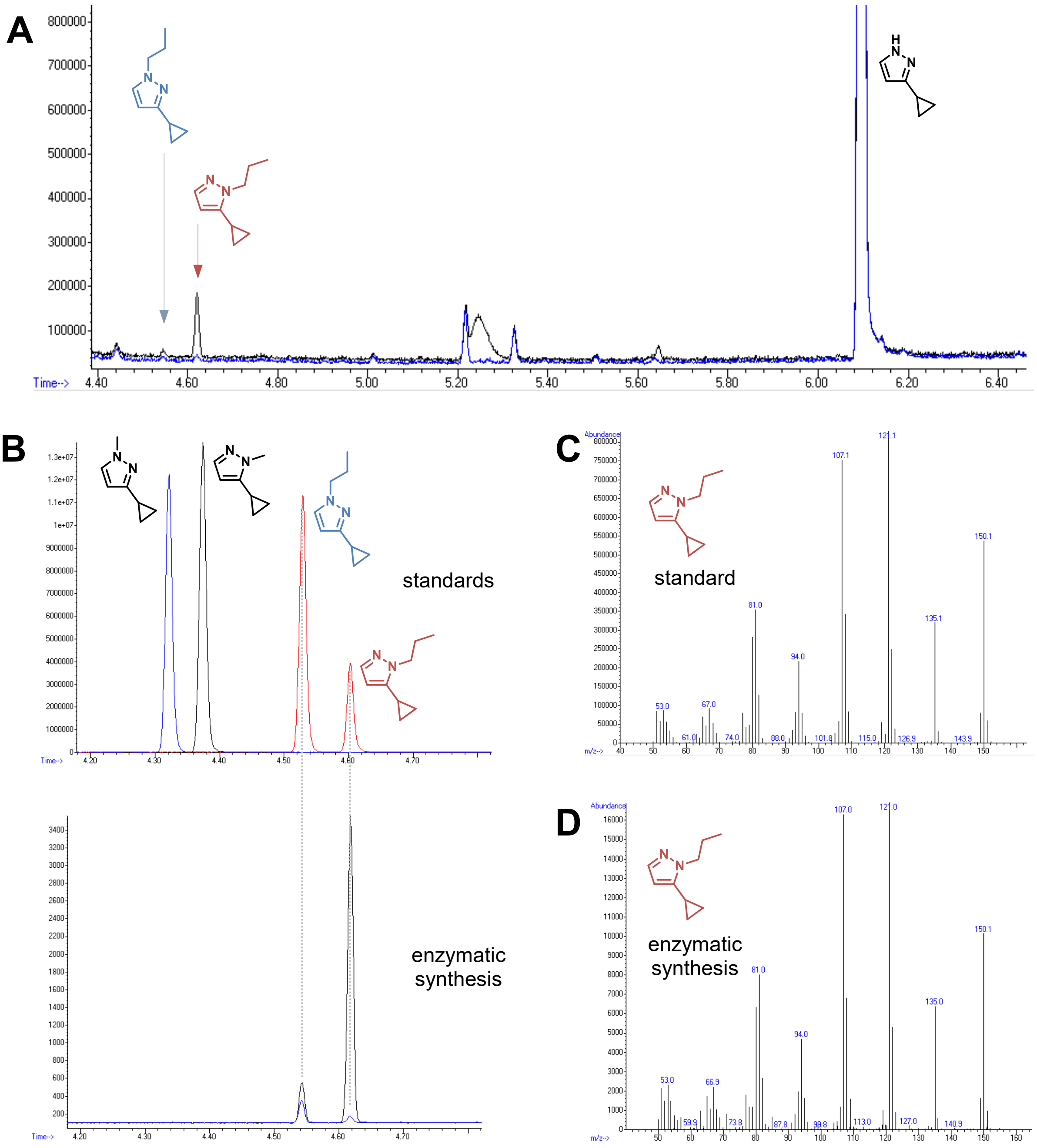

A) GC-MS Scan chromatogram of enzymatic propylation of 3-cyclopropylpyrazole (**3**) using iodopropane (black) compared to the control reaction without enzymes (blue) and **B)** comparison of GC-MS SIM chromatograms of standard compounds (top) with the product of selective enzymatic propylation using propyl iodide (bottom). The reaction was with 75 µM NSA-synthase and 75 µM v36, 1 mM SAH, 2 mM 3-cyclopropylpyrazole (**3**) and 10 equivalents iodopropane at 30°C for 50 h. The chromatogram of the standards (top) also

show the retention of the methylated compounds to proof that methylation is not observed in the enzymatic reaction. The chromatogram of the enzymatic reaction (bottom) shows the enzyme-catalyzed reaction (black) in comparison with the control experiment (without enzyme in blue). This reveals that there is a non-selective, non-enzyme-catalyzed background reaction. **C**) and **D**) show the corresponding MS spectra for the enzyme-catalyzed selective synthesis of 5-cyclopropyl-1-propy-pyrazole (**D**) in comparison to the authentic standard (**C**). Scan measurements were performed with the same chromatography as it is described in paragraph **I.(D)5**.

IV. Calibration curves for initial rate measurements, enzymatic ethylation and propylation

All calibration curves were measured from 3 single weighted standards and measured via GC-FID. The calibration curves of 1-propyl-3-cyclopropylpyrazole (**8a**) and 1-propyl-5-cyclopropylpyrazole (**8b**) were measured via GC-MS.

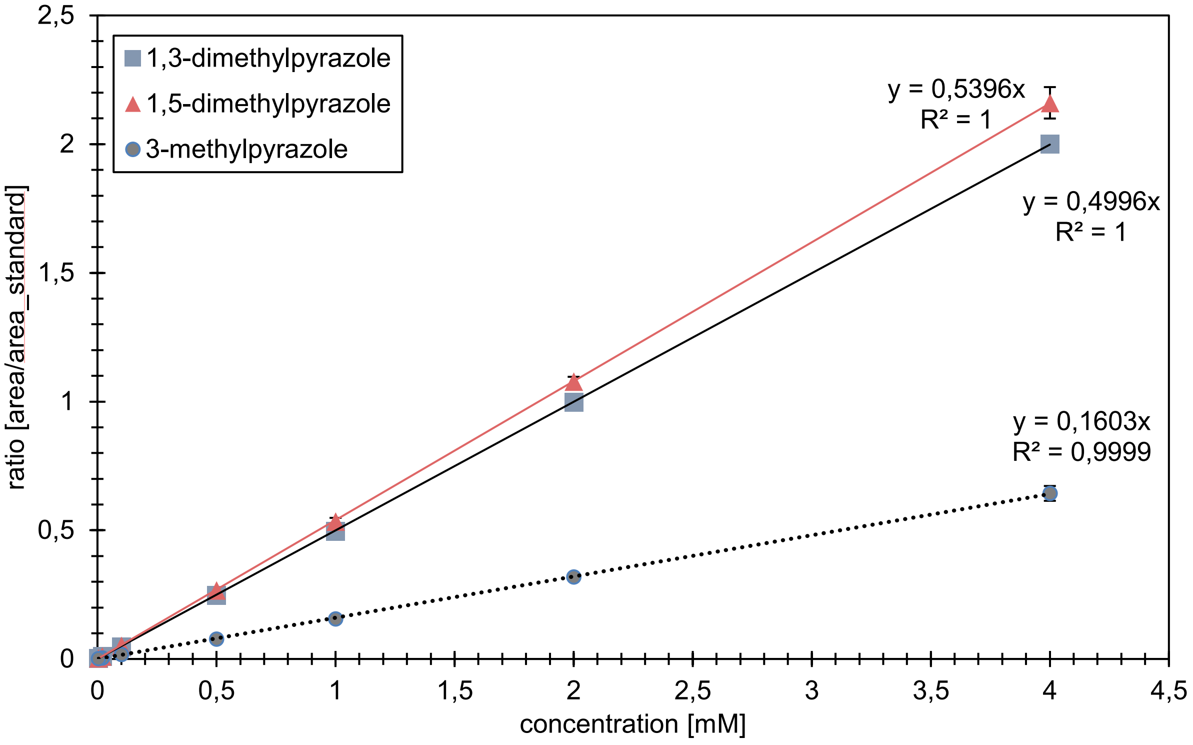

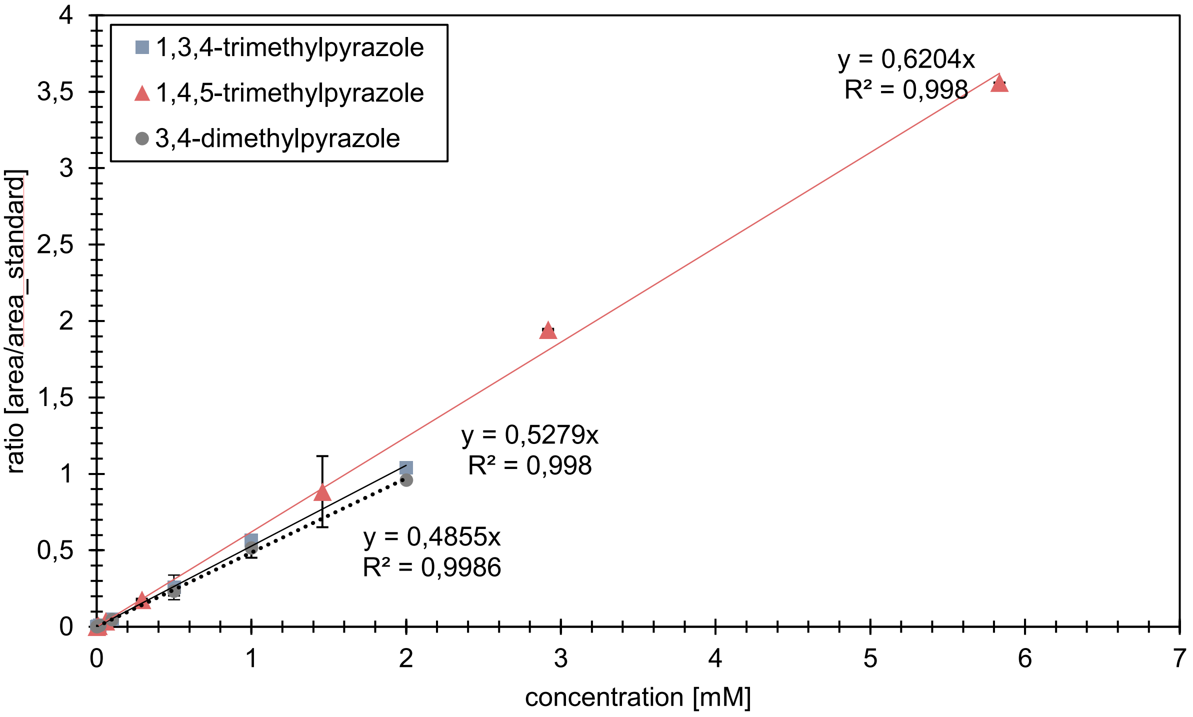

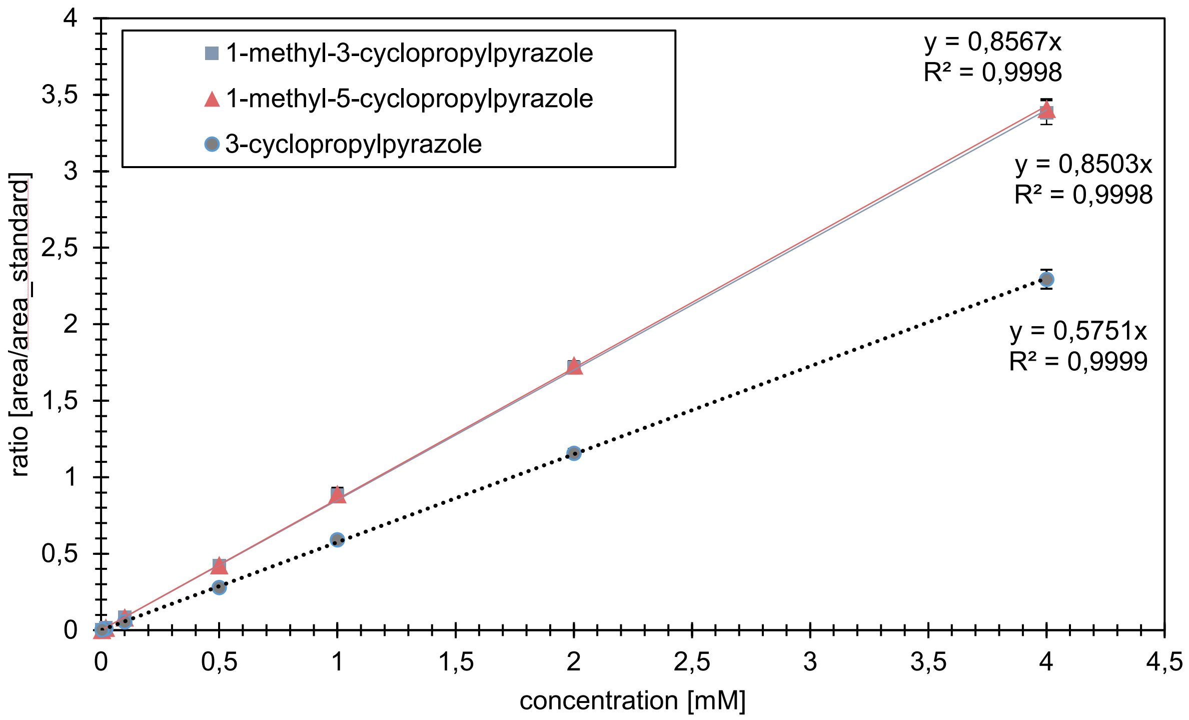
1-methyl-3-cyclopropylpyrazole
1-methyl-5-cyclopropylpyrazole
3-cyclopropylpyrazole
y = 0,8567x
R² = 0,9998
y = 0,8503x
R² = 0,9998
y = 0,5751x
R² = 0,9999
ratio [area/area_standard]
concentration [mM]
0
0,5
1
1,5
2
2,5
3
3,5
4
4,5

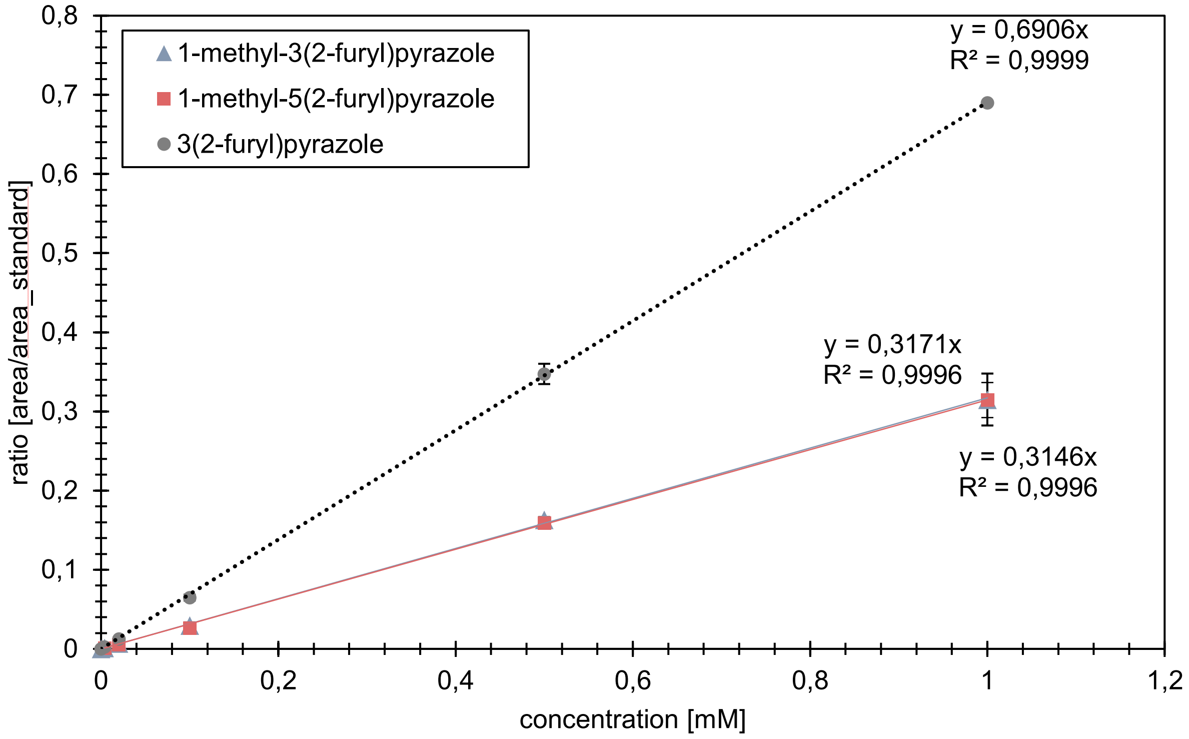
1-methyl-3(2-furyl)pyrazole
1-methyl-5(2-furyl)pyrazole
3(2-furyl)pyrazole
y = 0,6906x
R² = 0,9999
y = 0,3171x
R² = 0,9996
y = 0,3146x
R² = 0,9996
ratio [area/area_standard]
concentration [mM]
0
0,1
0,2
0,3
0,4
0,5
0,6
0,7
0,8
1
1,2

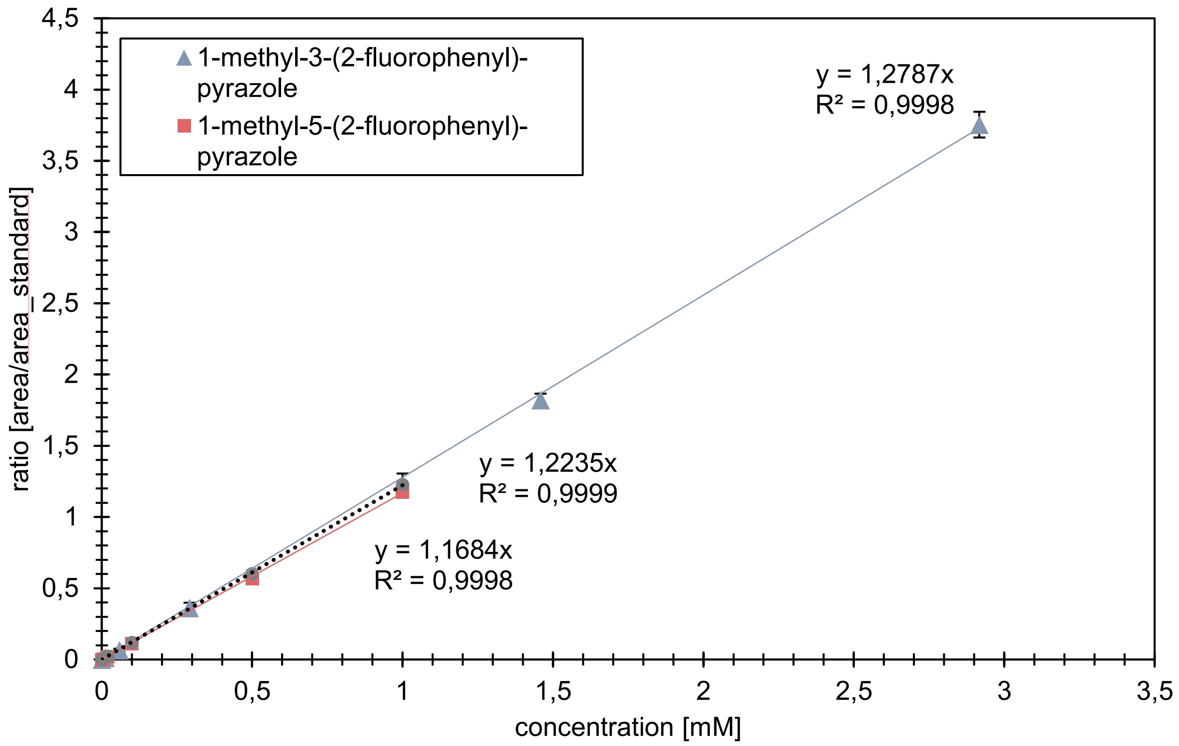
1-methyl-3-(2-fluorophenyl)-pyrazole
1-methyl-5-(2-fluorophenyl)-pyrazole
y = 1,2787x
R² = 0,9998
y = 1,2235x
R² = 0,9999
y = 1,1684x
R² = 0,9998
ratio [area/area_standard]
concentration [mM]

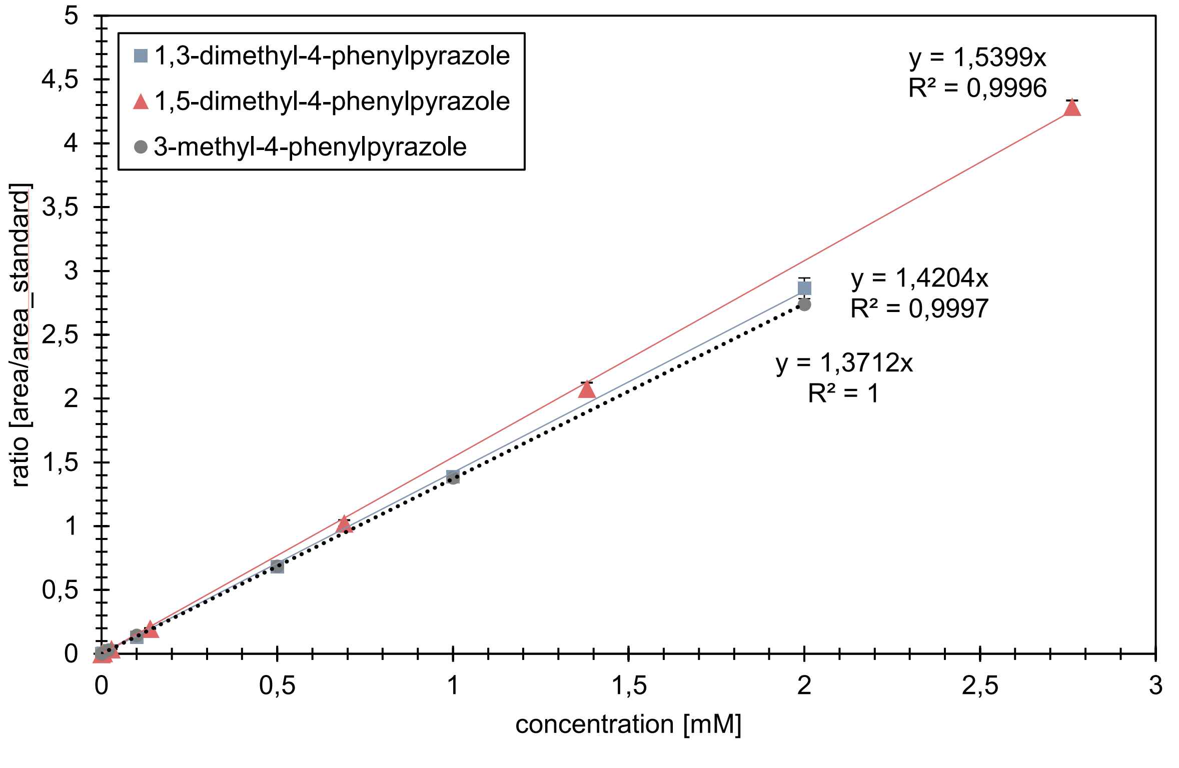
1,3-dimethyl-4-phenylpyrazole
1,5-dimethyl-4-phenylpyrazole
3-methyl-4-phenylpyrazole
y = 1,5399x
R² = 0,9996
y = 1,4204x
R² = 0,9997
y = 1,3712x
R² = 1
ratio [area/area_standard]
concentration [mM]

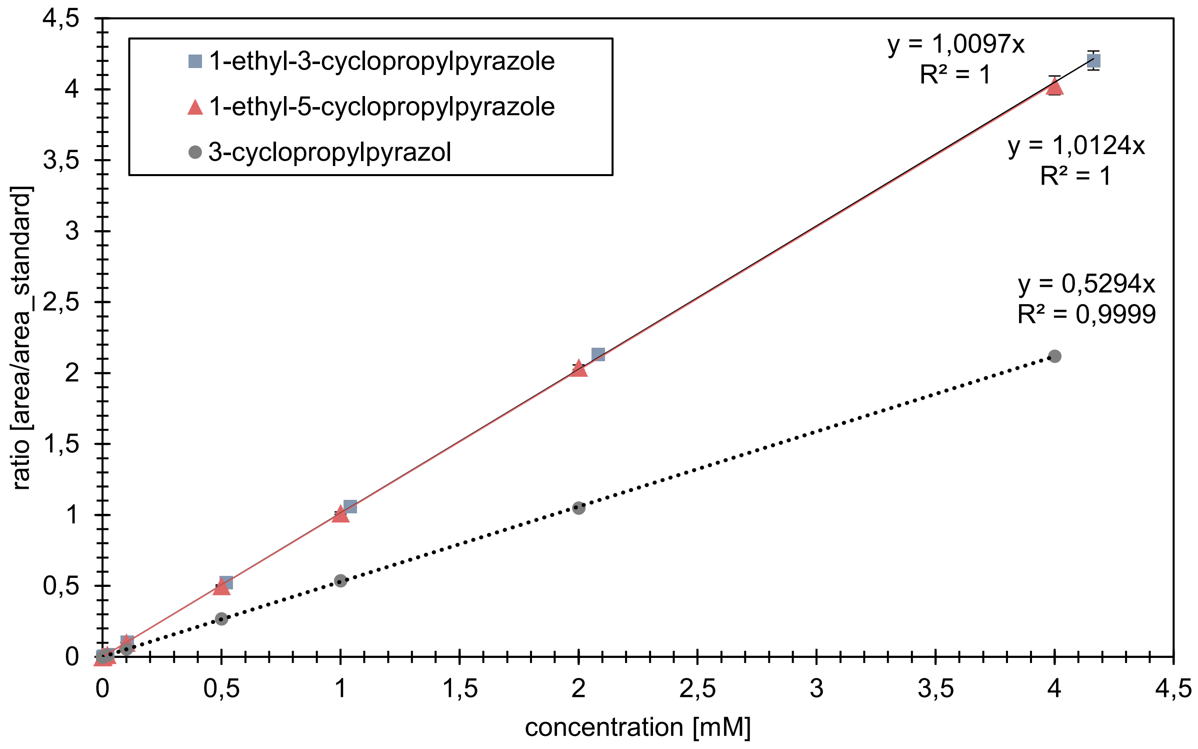

1-ethyl-3-cyclopropylpyrazole
1-ethyl-5-cyclopropylpyrazole
3-cyclopropylpyrazol
y = 1,0097x
R² = 1
y = 1,0124x
R² = 1
y = 0,5294x
R² = 0,9999
ratio [area/area_standard]
concentration [mM]
0
0,5
1
1,5
2
2,5
3
3,5
4
4,5

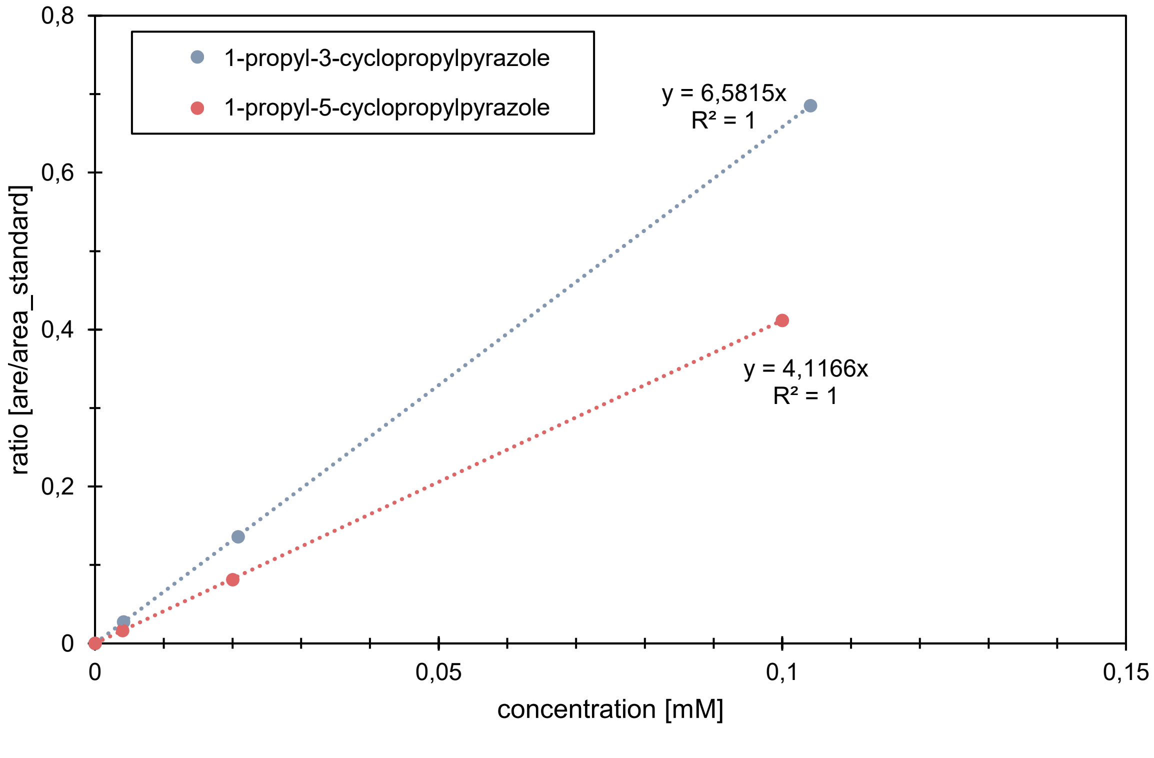

1-propyl-3-cyclopropylpyrazole
1-propyl-5-cyclopropylpyrazole
y = 6,5815x
R² = 1
y = 4,1166x
R² = 1
ratio [are/area_standard]
concentration [mM]
0
0,2
0,4
0,6
0,8
0,05
0,1
0,15

V. Chemical synthesis of product standards

All product standards were synthesized following the protocol from paragraph **II.(O)**.

1,3-dimethylpyrazole (1a):

The reaction was performed on 0.12 mmol scale and the crude product was compared to commercially available standards and used for selectivity measurements. NMR Data is derived from the commercial standard. **NMR Data:** ^{1}H NMR (500 MHz, $CDCl_3$) δ 7.21 (d, *J* = 2.3 Hz, 1H), 5.99 (d, *J* = 2.3 Hz, 1H), 3.82 (s, 3H), 2.26 (s, 3H). ^{13}C NMR (126 MHz, $CDCl_3$) δ 148.66, 130.71, 105.12, 38.63, 13.59. HRESIMS m/z 97.0765 (calculated for $C_5H_8N_2$ 97.0760).

1,5-dimethylpyrazole (1b):

The reaction was performed on 0.12 mmol scale and the crude product was compared to commercially available standards and used for selectivity measurements. NMR Data is derived from the commercial standard. **NMR Data**: ^{1}H NMR (500 MHz, CDCl3) δ 7.35 (d, J = 1.8 Hz, 1H), 6.00 (d, J = 1.8 Hz, 1H), 3.78 (s, 3H), 2.27 (s, 3H). ^{13}C NMR (126 MHz, CDCl3) δ 138.28, 138.12, 105.24, 36.13, 11.23. HRESIMS m/z 97.0768 (calculated for $C_5H_8N_2$ 97.0760).

1,3,4-trimethylpyrazole (2a), 1,4,5-trimethylpyrazole (2b):

The reaction was performed on 1.6 mmol scale. The product mixture was purified by column chromatography (3:1 ethyl acetate/cyclohexane). Isolated 39,99 mg. Product standard is mixture of 1,3,4- and 1,4,5-trimethyl-pyrazole (molar ratio 25.5 : 74.5). **NMR Data**: ^{1}H NMR (500 MHz, $CDCl_3$) δ 7.20 (s, 1H), 7.02 (s, 1H), 3.75 (s, 3H), 3.73 (s, 3H), 2.16 (s, 3H), 2.14 (s, 3H), 1.96 (s, 3H + 3H). ^{13}C NMR (126 MHz, $CDCl_3$) δ 147.23, 138.30, 135.53, 129.36, 113.96, 113.36, 38.41, 36.36, 11.56, 9.28, 8.82, 8.37. NMR spectrum of 1,3,4-trimethyl-pyrazole is in accordance with literature.[20] HRESIMS m/z 111.0936 (calculated for $C_6H_{10}N_2$ 111.0917).

1-methyl-3-cyclopropylpyrazole (3a):

The reaction was performed on 0.1 mmol scale and the crude product was compared to commercially available standards and used for selectivity measurements. NMR Data is derived from the commercial standard. **NMR Data**: ^{1}H NMR (500 MHz, $CDCl_3$) δ 7.19 (d, *J* = 2.4 Hz, 1H), 5.86 (d, *J* = 2.5 Hz, 1H), 3.81 (s, 3H), 1.96 – 1.87 (m, 1H), 0.92 – 0.85 (m, 2H), 0.72 – 0.66 (m, 2H). ^{13}C NMR (126 MHz, $CDCl_3$) δ 155.34, 130.64, 101.77, 38.71, 9.22, 7.96 (2C). HRESIMS m/z 123.0920 (calculated for $C_7H_{10}N_2$ 123.0917).

1-methyl-5-cyclopropylpyrazole (3b):

The reaction was performed on 0.1 mmol scale and the crude product was compared to commercial available standards and used for selectivity measurements. NMR Data is derived from the commercially standard. **NMR Data**: ^{1}H NMR (500 MHz, $CDCl_3$) δ 7.33 (d, *J* = 1.8 Hz, 1H), 5.82 (d, *J* = 1.8 Hz, 1H), 3.89 (s, 3H), 1.75 – 1.66 (m, 1H), 1.00 – 0.92 (m, 2H), 0.68 – 0.61 (m, 2H). ^{13}C NMR (126 MHz, $CDCl_3$) δ 145.08, 137.97, 102.54, 36.33, 6.84 (2C), 6.23. HRESIMS m/z 123.0920 (calculated for $C_7H_{10}N_2$ 123.0917).

1-ethyl-3-cyclopropylpyrazole (7a):

The reaction was performed on 4.5 mmol scale. The product was purified by column chromatography (1:1 ethyl acetate/cyclohexane). Isolated 7.53 mg. **NMR Data**: ^{1}H NMR (500 MHz, $CDCl_3$) δ 7.24 (d, *J* = 2.2 Hz, 1H), 5.85 (d, *J* = 2.5 Hz, 1H), 4.09 (q, *J* = 7.4 Hz, 2H), 1.99 – 1.89 (m, 1H), 1.46 (t, *J* = 7.5 Hz, 3H), 0.92 – 0.88 (m, 2H), 0.71 – 0.68 (m, 2H). ^{13}C NMR (126 MHz, $CDCl_3$) δ 155.04, 128.86, 101.23, 46.77, 15.74, 9.30, 8.04 (2C). HRESIMS m/z 137.1079 (calculated for $C_8H_{12}N_2$ 137.1073).

1-ethyl-5-cyclopropylpyrazole (7b):

The reaction was performed on 4.5 mmol scale. The product was purified by column chromatography (1:1 ethyl acetate/cyclohexane). Isolated 20.05 mg. **NMR Data**: ^{1}H NMR (500 MHz, $CDCl_3$) δ 7.34 (d, *J* = 2.1 Hz, 1H), 5.80 (d, *J* = 1.8 Hz, 1H), 4.23 (q, *J* = 7.0 Hz, 2H), 1.76 – 1.67 (m, 1H), 1.44 (t, *J* = 7.3 Hz, 3H), 0.98 – 0.92 (m, 2H), 0.67 – 0.63 (m, 2H). ^{13}C NMR (126 MHz, $CDCl_3$) δ 144.28, 138.02, 102.32, 44.08, 15.52, 6.96 (2C), 6.14. HRESIMS m/z 137.1073 (calculated for $C_8H_{12}N_2$ 137.1073). Purity 56%:36%:9% 1-ethyl-5-cyclopropylpyrazole:ethyl acetate:cyclohexane.

1-propyl-3-cyclopropylpyrazole (8a):

The reaction was performed on 4.5 mmol scale. The product was purified by column chromatography (1:3 ethyl acetate/cyclohexane). Isolated 270.1 mg. **NMR Data**: ^{1}H NMR (500 MHz, $CDCl_3$) δ 7.22 (d, *J* = 2.4 Hz, 1H), 5.84 (d, *J* = 1.9, 1H), 3.98 (t, *J* = 7.3 Hz, 2H), 1.92 – 1.88 (m, 1H), 1.87 – 1.81 (m, 2H), 0.95 – 0.86 (m, 2H and t, 3H), 0.70 – 0.68 (m, 2H). ^{13}C NMR (126 MHz, $CDCl_3$) δ 155.05, 129.62, 101.01, 53.74, 23.92, 11.31, 9.33, 8.03 (2C). HRESIMS m/z 151.1232 (calculated for $C_9H_{14}N_2$ 151.1230). 1-propyl-3-cyclopropyl-pyrazole is contaminated with the regioisomer 5-cyclopropyl-1-propyl pyrazole with the molar ratio 73:27.

1-propyl-5-cyclopropylpyrazole:

The reaction was performed on 4.5 mmol scale. The product was purified by column chromatography (1:3 ethyl acetate/cyclohexane). Isolated 28.2 mg. **NMR Data**: ^{1}H NMR (500 MHz, $CDCl_3$) δ 7.35 (d, *J* = 1.9 Hz, 1H), 5.80 (d, *J* = 1.7, 1H), 4.17 – 4.11 (t, *J* = 7.1, 2H), 1.93 – 1.86 (m, 2H), 1.72 (m, 1H), 0.98 – 0.85 (m, 2H and t, 3H), 0.67 – 0.64 (m, 2H). ^{13}C NMR (126 MHz, $CDCl_3$) δ 144.78, 138.01, 102.04, 50.79, 23.70, 11.41, 7.17 (2C), 6.27. HRESIMS m/z 151.1231 (calculated for $C_9H_{14}N_2$ 151.1230)

1-methyl-3(2-furyl)pyrazole (5a):

The reaction was performed on 0.72 mmol scale. The product was purified by column chromatography (1:9 ethyl acetate/cyclohexane). Isolated 13.63 mg. **NMR Data**: ^{1}H NMR (500 MHz, $CDCl_3$) δ 7.43 (d, *J* = 1.9 Hz, 1H), 7.34 (d, *J* = 2.4 Hz, 1H), 6.62 (d, *J* = 3.3 Hz, 1H), 6.45 (d, *J* = 2.5 Hz, 1H), 6.44 (dd, *J* = 3.3, 1.9 Hz, 1H), 3.93 (s, 3H). ^{13}C NMR (126 MHz, $CDCl_3$) δ 149.08, 144.13, 141.73, 131.18, 111.32, 105.39, 102.86, 39.16. HRESIMS m/z 149.0719 (calculated for $C_8H_8N_2O$ 149.0709).

1-methyl-5(2-furyl)pyrazole (5b):

The reaction was performed on 0.72 mmol scale. The product was purified by column chromatography (1:9 ethyl acetate/cyclohexane). Isolated 85.5 mg. **NMR Data**: ^{1}H NMR (500 MHz, $CDCl_3$) δ 7.51 (d, *J* = 1.9 Hz, 1H), 7.45 (d, *J* = 1.9 Hz, 1H), 6.55 (d, *J* = 3.4 Hz, 1H), 6.50 (dd, *J* = 3.1, 1.8 Hz, 1H), 6.47 (d, *J* = 1.9 Hz, 1H), 4.05 (s, 3H). ^{13}C NMR (126 MHz, $CDCl_3$) δ 145.13, 142.70, 138.55, 134.15, 111.53, 108.44, 105.12, 38.72. NMR data are in accordance with literature.[21] HRESIMS m/z 149.0715 (calculated for $C_8H_8N_2O$ 149.0709).

1-methyl-3(2-fluorophenyl)pyrazole (5a):

The reaction was performed on 0.6 mmol scale. The product mixture was purified by column chromatography (1:9 ethyl acetate/cyclohexane). Isolated 19.95 mg. **NMR Data**: ^{1}H NMR (500 MHz, $CDCl_3$) δ 7.97 (td, *J* = 7.7, 1.9 Hz, 1H), 7.40 (d, *J* = 2.5 Hz, 1H), 7.30 – 7.21 (m, 1H), 7.17 (t, *J* = 7.5 Hz, 1H), 7.11 (dd, *J* = 11.5, 8.2 Hz, 1H), 6.69 (dd, *J* = 4.0, 2.2 Hz, 1H), 3.96 (s, 3H). ^{13}C NMR (126 MHz, CDCl3) δ 161.14, 159.15, 146.20, 131.20, 128.95, 128.42, 124.31, 121.55, 121.45, 116.05, 106.58, 39.21. HRESIMS m/z 177.0823 (calculated for $C_{10}H_9F_1N_2$ 177.0823).

1-methyl-5(2-fluorophenyl)pyrazole (5b):

The reaction was performed on 0.6 mmol scale. The product mixture was purified by column chromatography (1:9 ethyl acetate/cyclohexane). Isolated 70.92 mg. Product standard is mixture of 1-methyl-3- and 1-methyl-5(fluorophenyl)-pyrazole (molar ratio 76 : 24). HRESIMS m/z 177.0826 (calculated for $C_{10}H_9F_1N_2$ 177.0823).

1,5-dimethyl-4-phenylpyrazole (6b):

The reaction was performed on 0.63 mmol scale. The product was purified by column chromatography (1:9 ethyl acetate/cyclohexane). Isolated 6.34 mg. **NMR Data**: ^{1}H NMR (500 MHz, $CDCl_3$) δ 7.56 (s, 1H), 7.44 – 7.33 (m, 4H), 7.30 – 7.22 (m, 1H), 3.85 (s, 3H), 2.38 (s, 3H). ^{13}C NMR (126 MHz, $CDCl_3$) δ 137.37, 135.19, 134.13, 128.77, 127.85, 126.23, 120.95, 36.65, 10.51. HRESIMS m/z 173.1073 (calculated for $C_{11}H_{12}N_2$ 173.1073). Purity: The sample contains 90.8% 1,5-dimethyl-4-phenyl-pyrazole and 9.2% 1,3-dimethyl-4-phenyl-pyrazole.

1,3-dimethyl-4-phenylpyrazole (6a):

The reaction was performed on 0.63 mmol scale. The product mixture was purified by column chromatography (1:9 ethyl acetate/cyclohexane). Isolated 65.37 mg. Product standard is mixture of 1,3-dimethyl- and 1,5-dimethyl-4-phenyl-pyrazole (molar ratio 42 : 58). HRESIMS m/z 173.1077 (calculated for $C_{11}H_{12}N_2$ 173.1073).

VI. NMR spectra of product standards

1,3-dimethylpyrazole (1a, commercial standard):

^{1}H-NMR

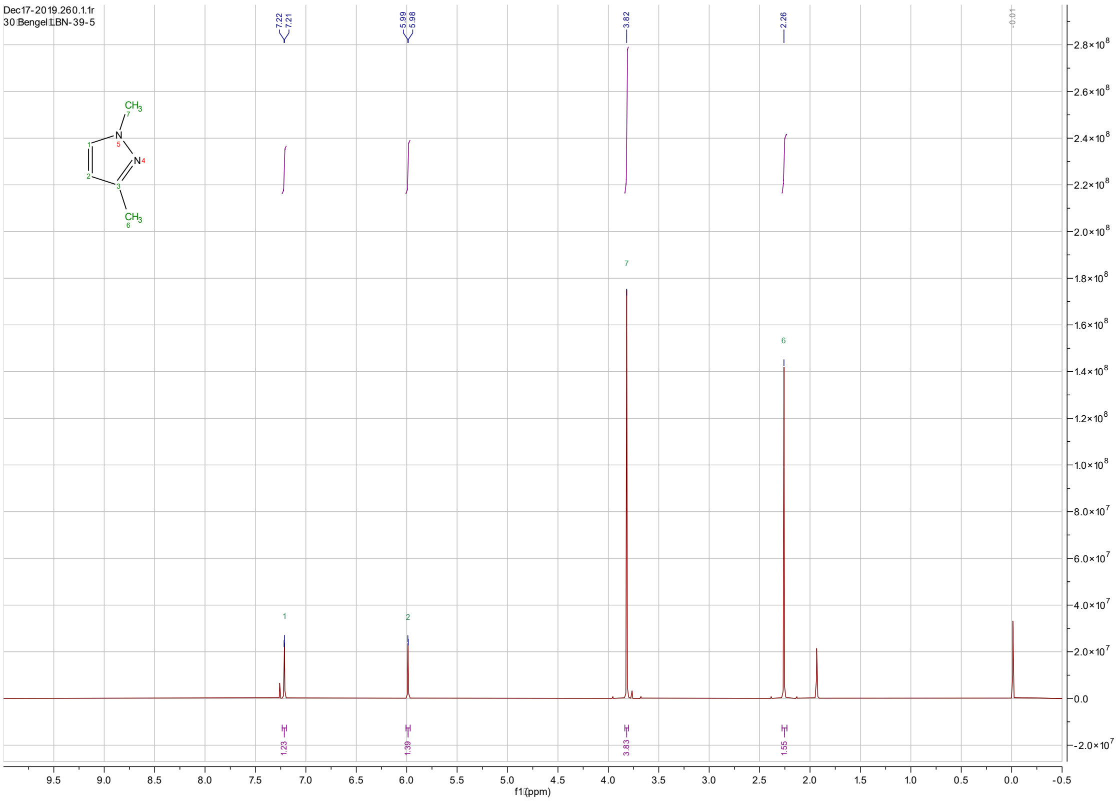

^{13}C-NMR

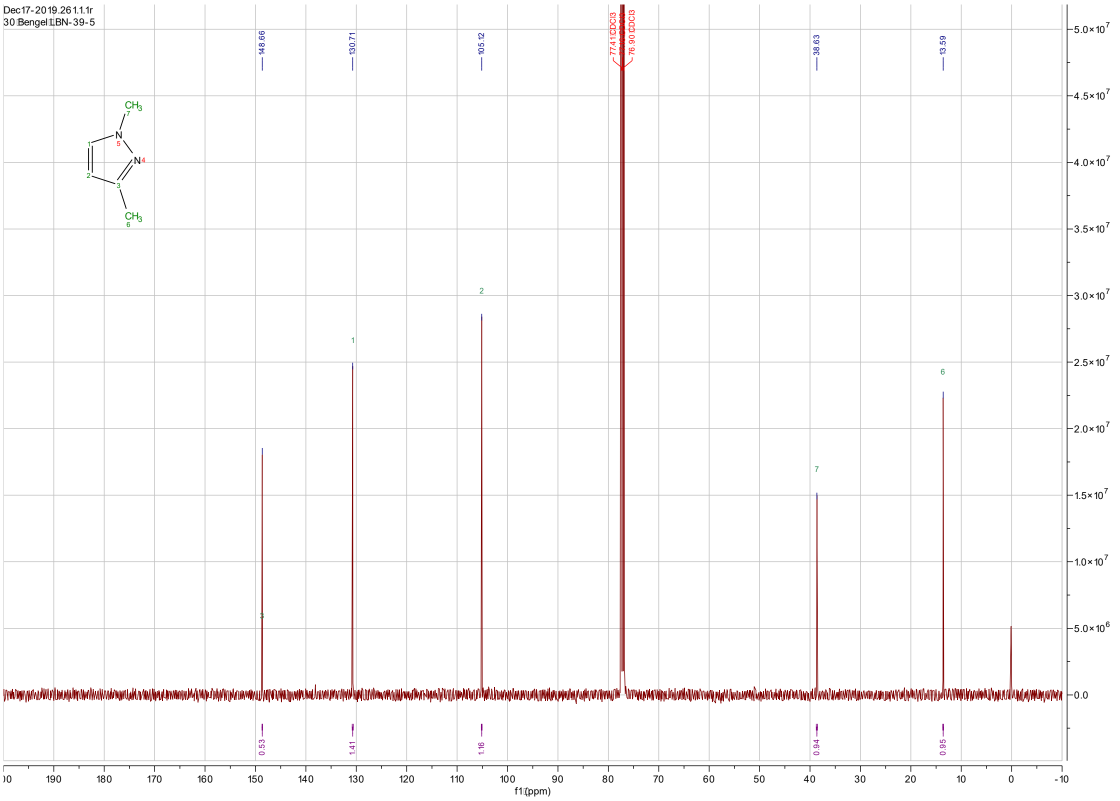

1,5-dimethylpyrazole (1b, commercial standard):

1H-NMR

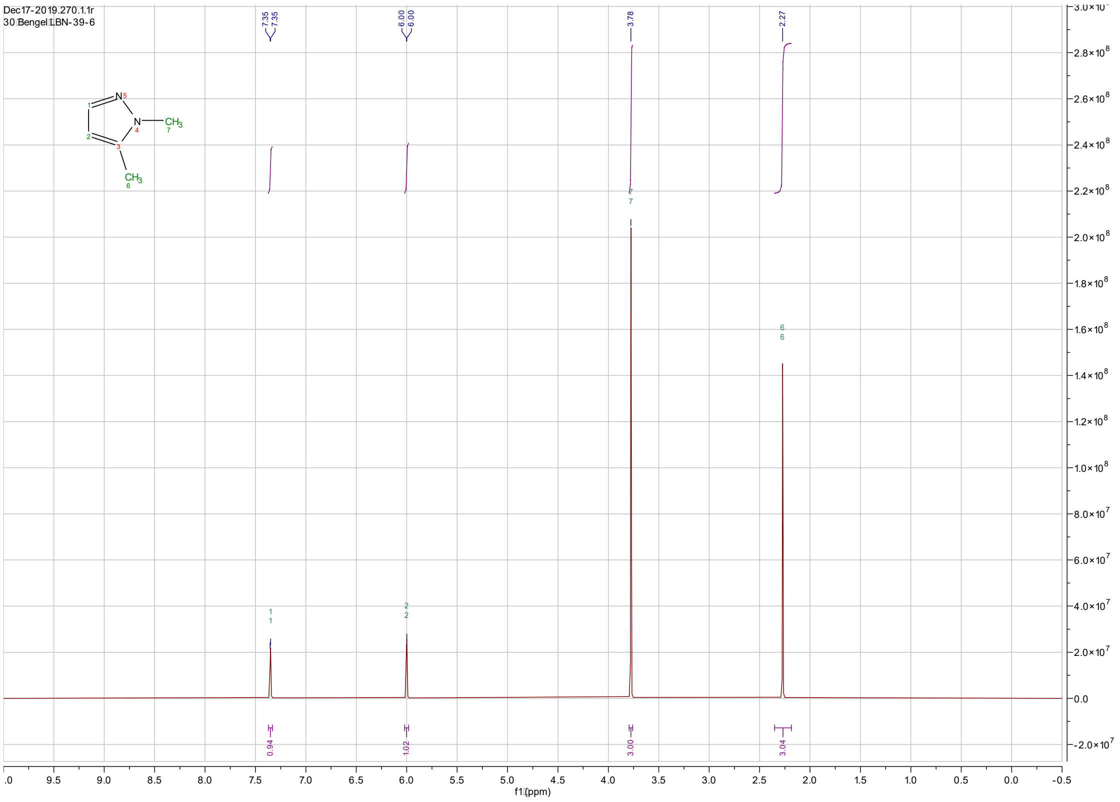

13C-NMR

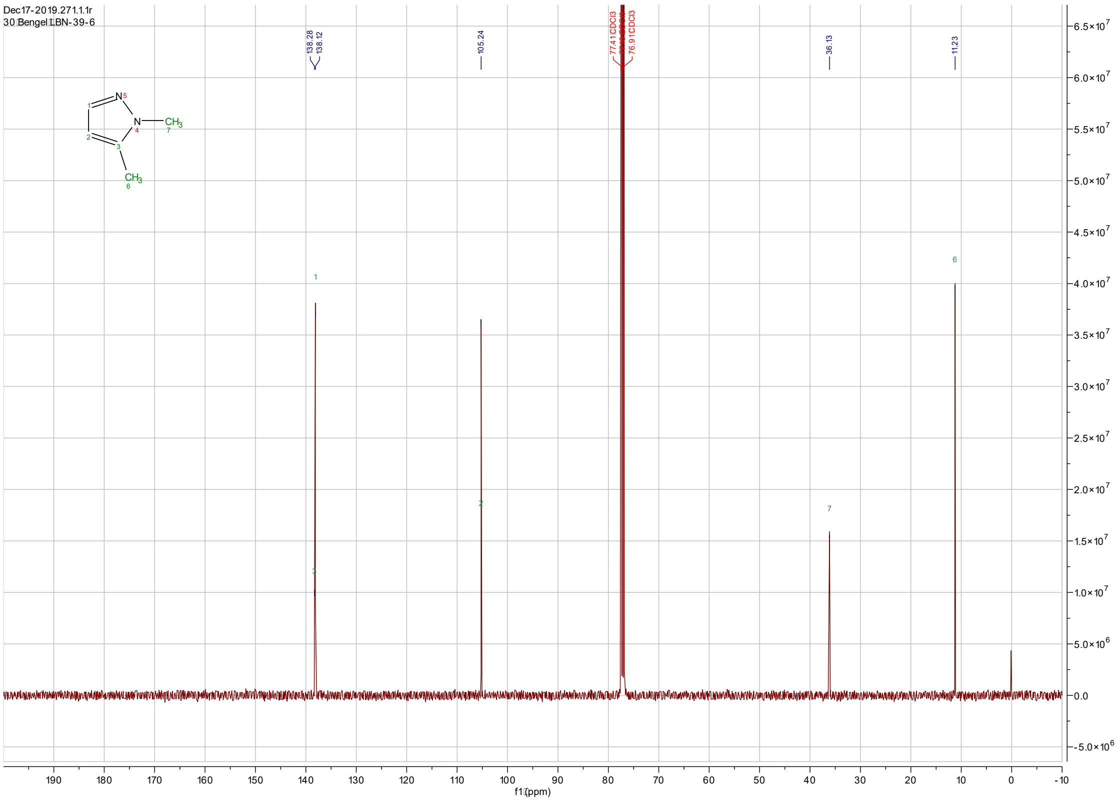

Mixture of 1,3,4-trimethylpyrazole (2a, 25.5%) and 1,4,5-trimethylpyrazole (2b, 74.5%):

^{1}H-NMR

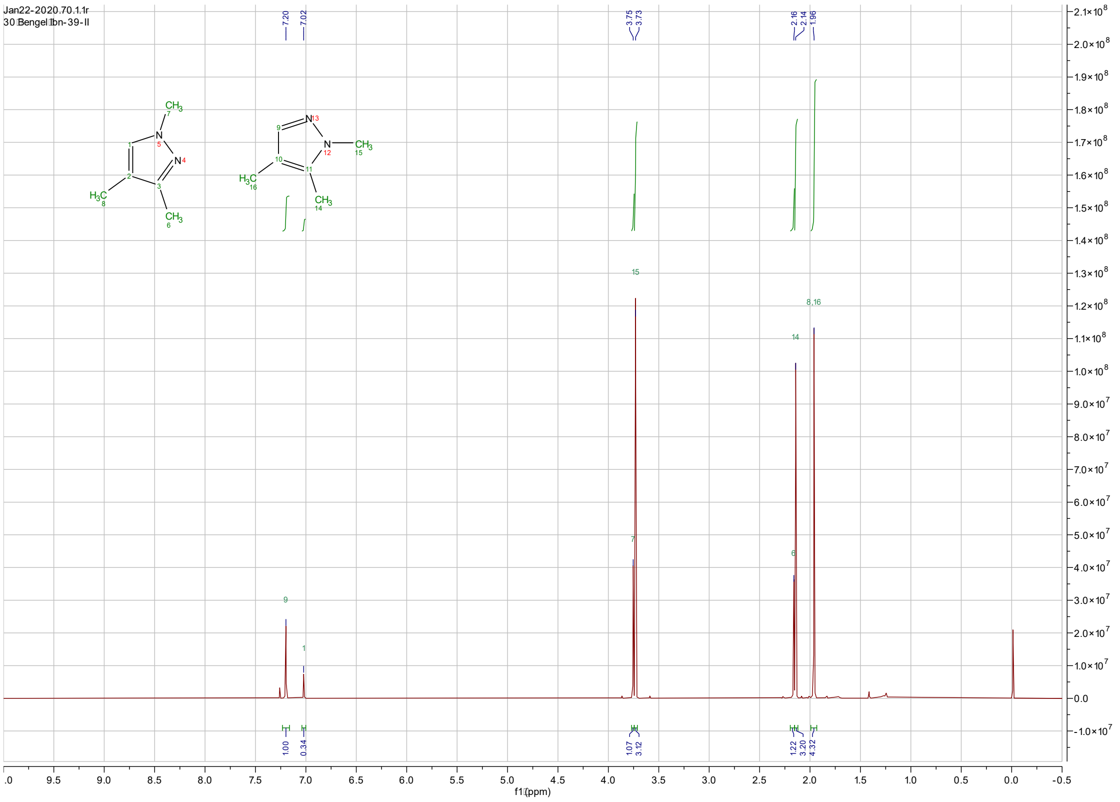

^{13}C-NMR

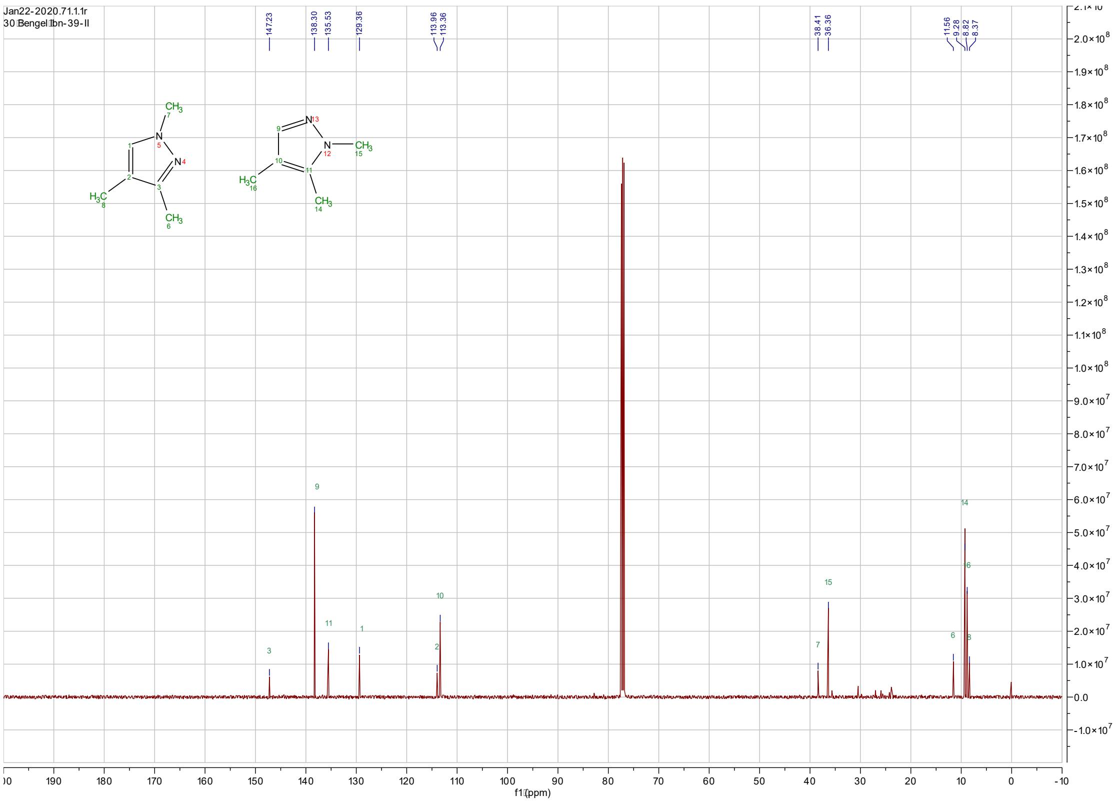

$^{1}H,^{13}C$-HSQC-NMR

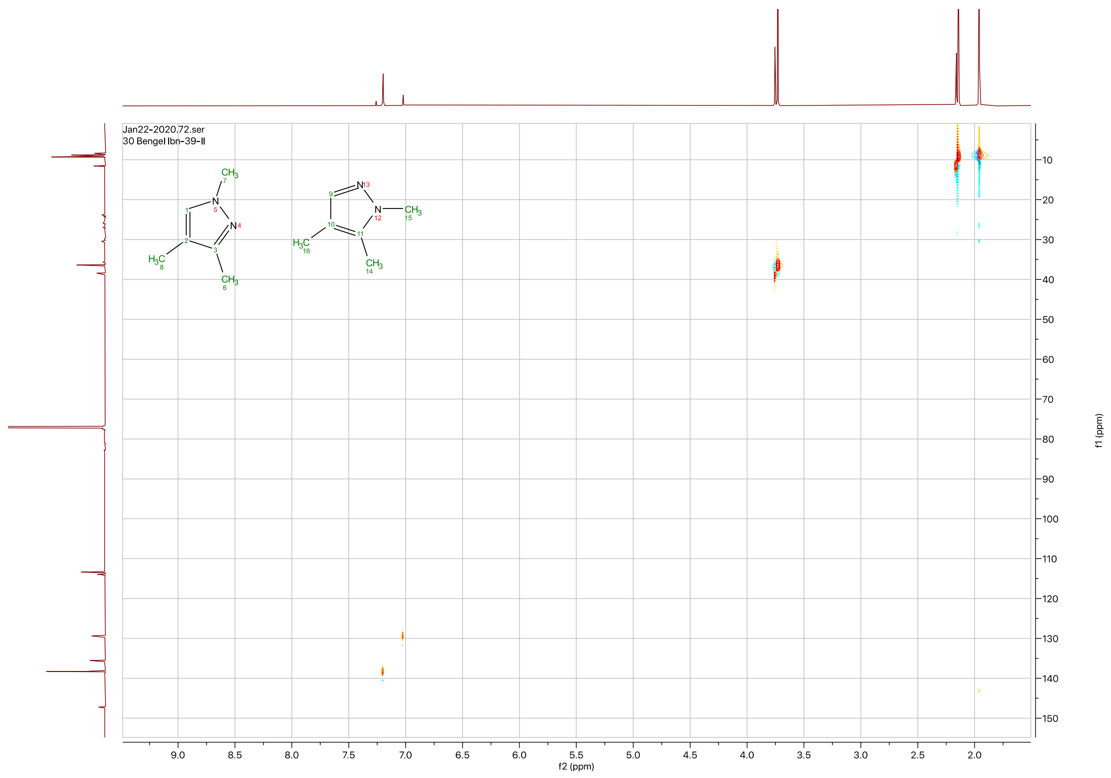

$^{1}H,^{13}C$-HMBC-NMR

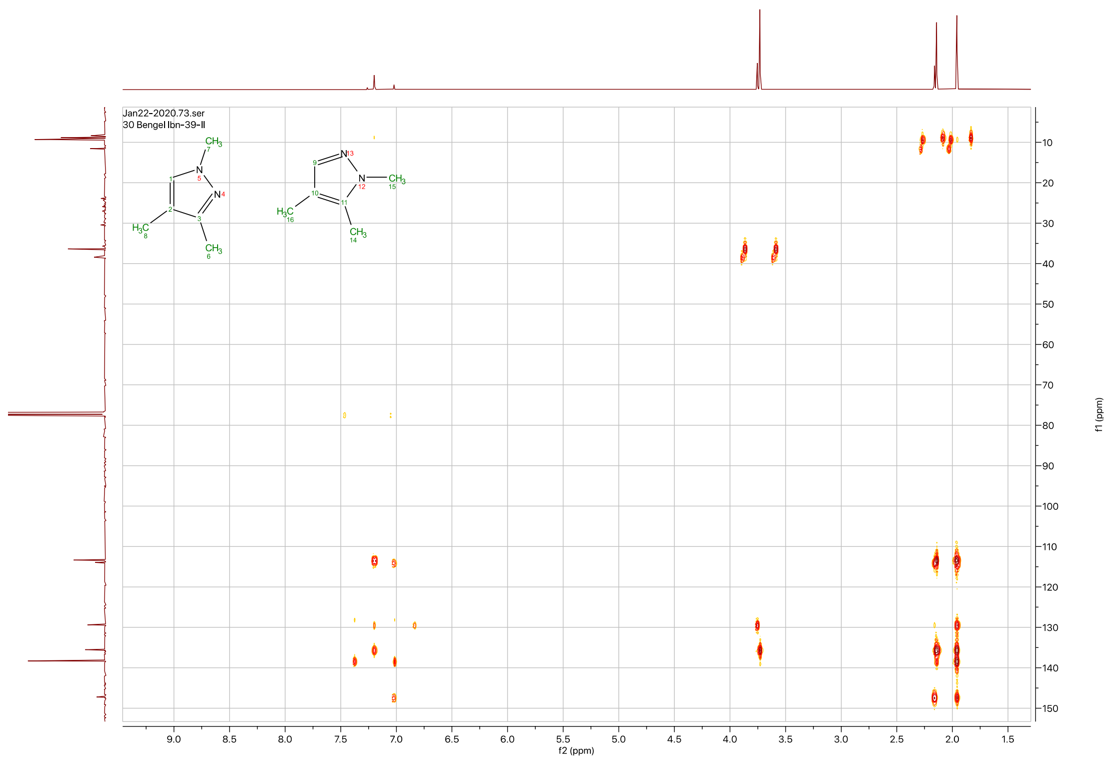

1-methyl-3-cyclopropylpyrazole (3a, commercial standard):

^1H-NMR

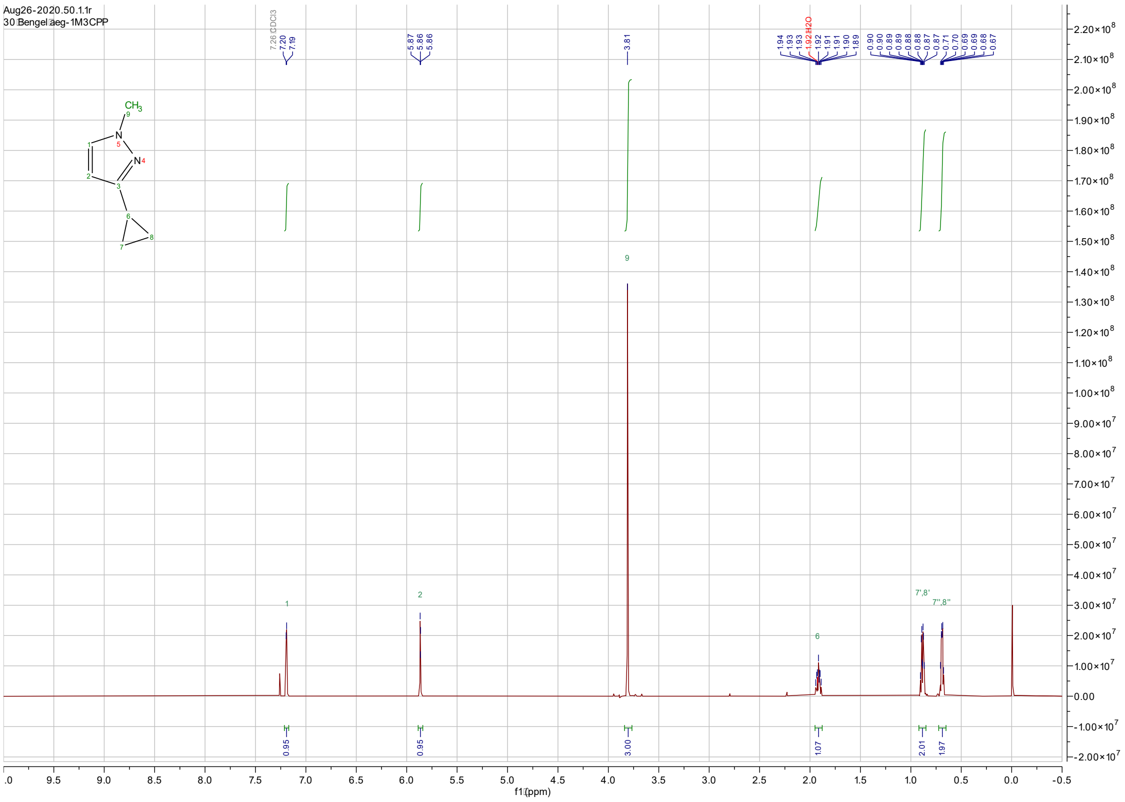

^13C-NMR

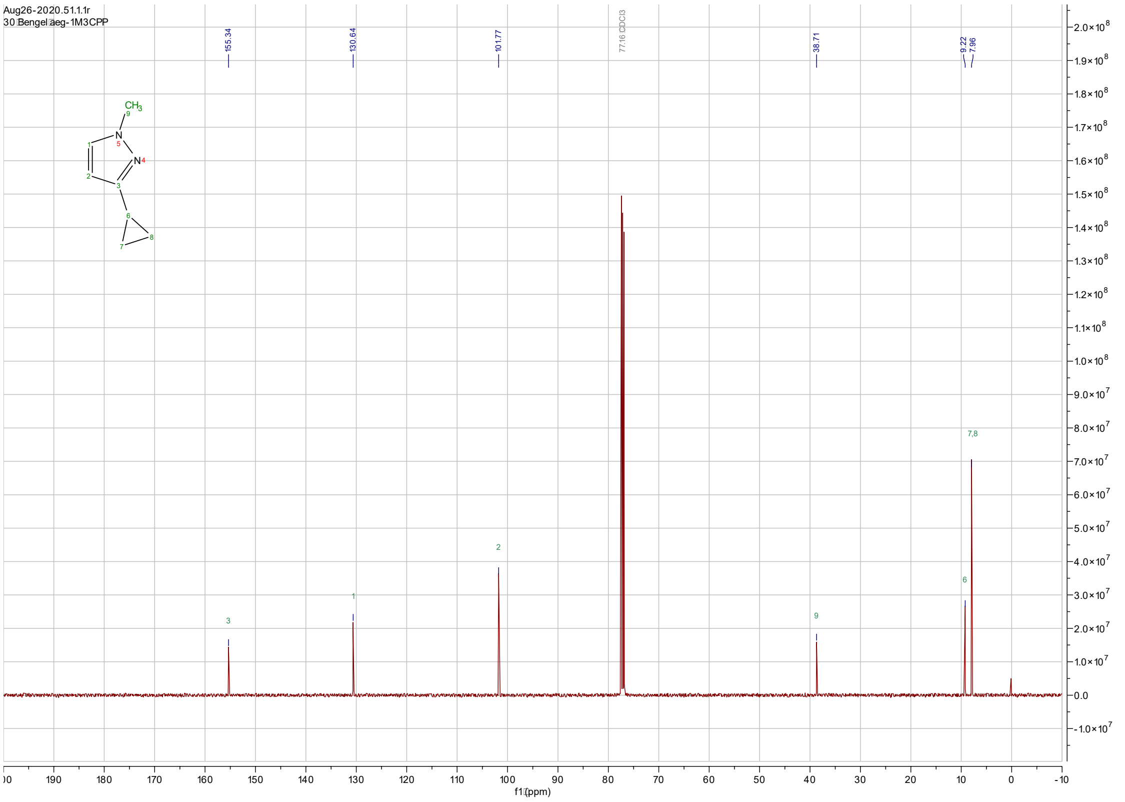

1-methyl-5-cyclopropylpyrazole (3b, commercial standard):

^{1}H-NMR

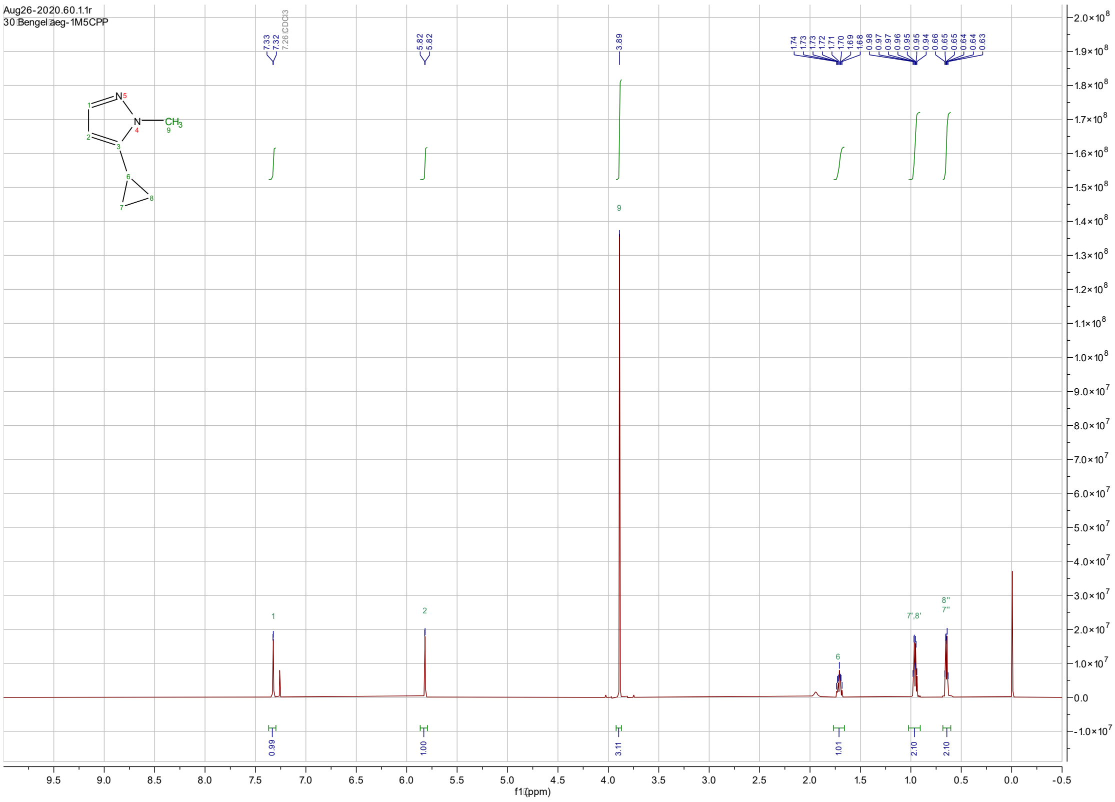

^{13}C-NMR

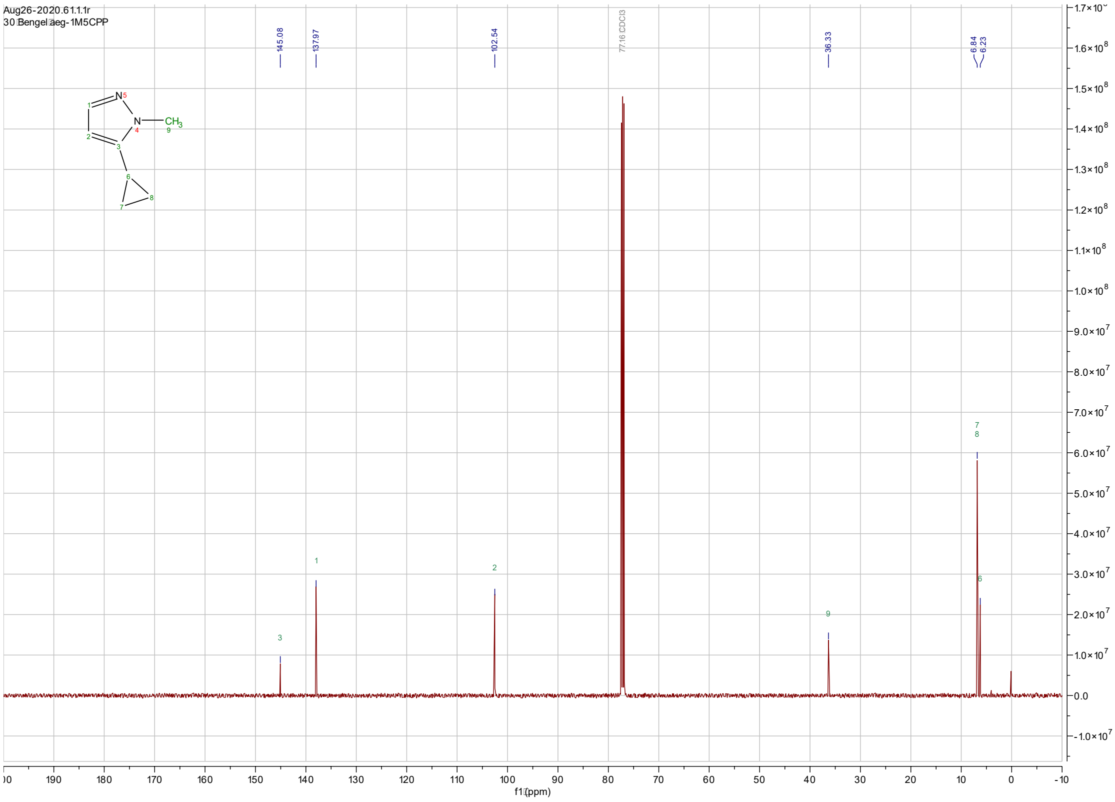

1-ethyl-3-cyclopropylpyrazole (7a):

^{1}H-NMR

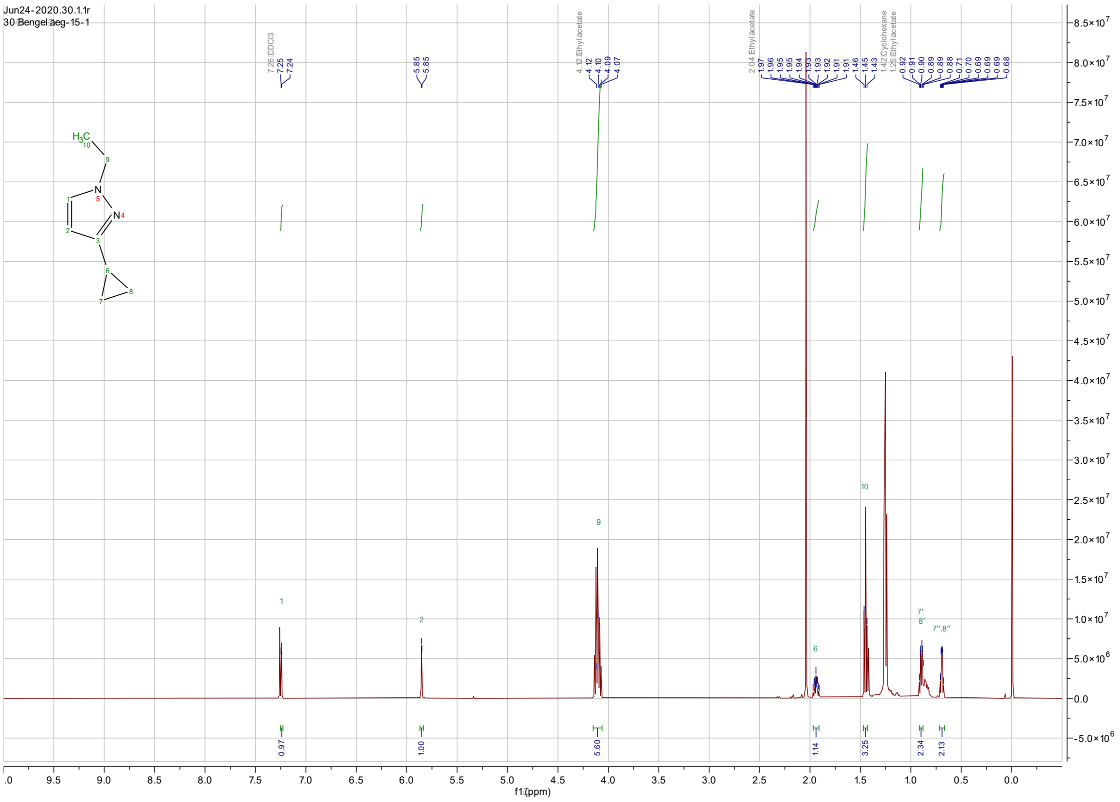

^{13}C-NMR

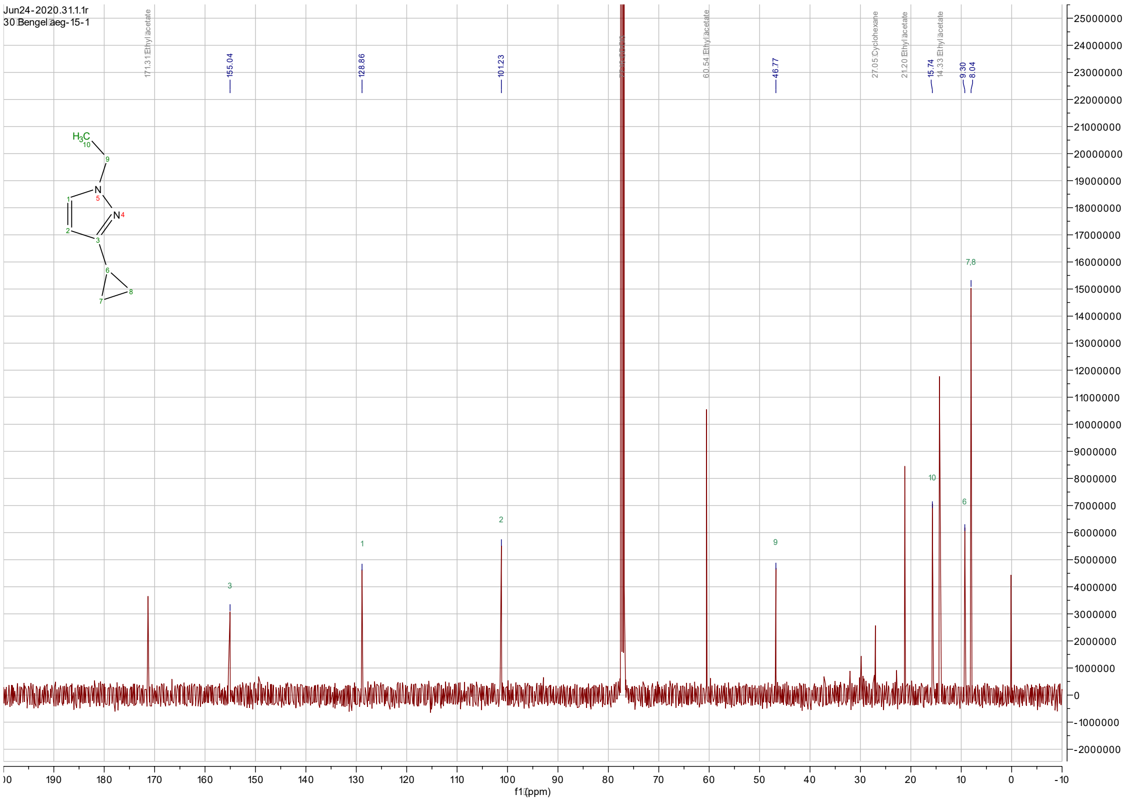

1-ethyl-5-cyclopropylpyrazole (7b):

^{1}H-NMR

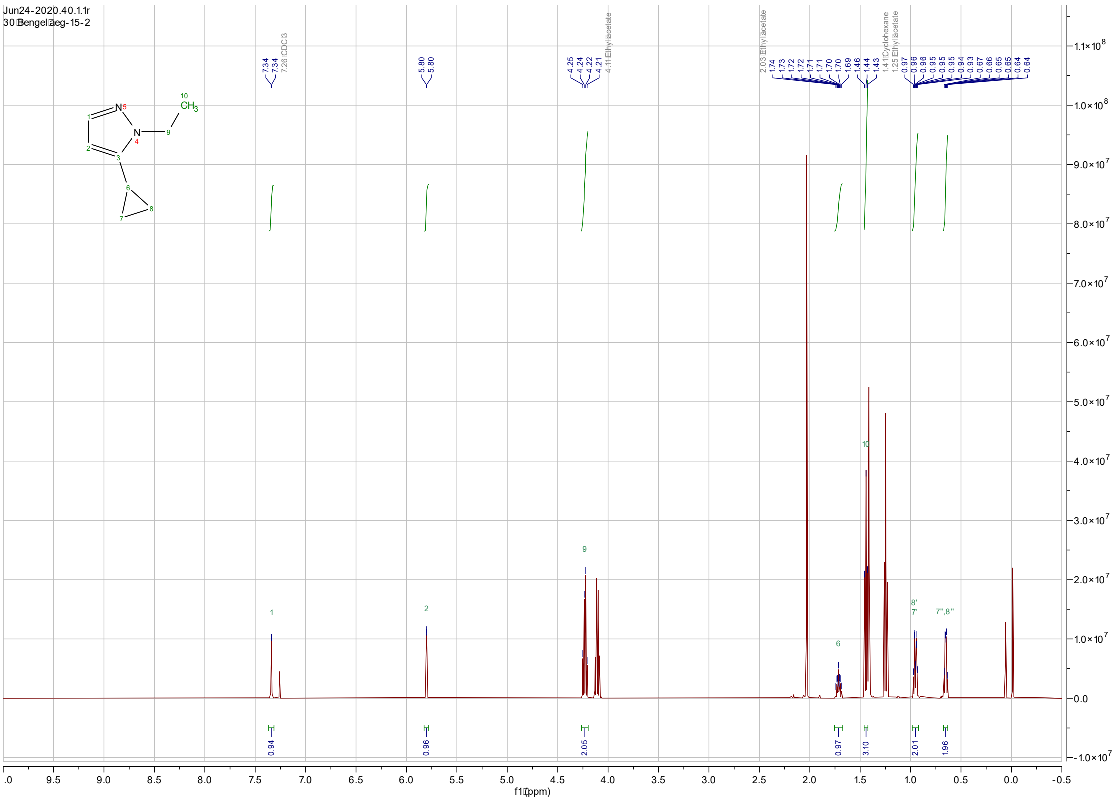

^{13}C-NMR

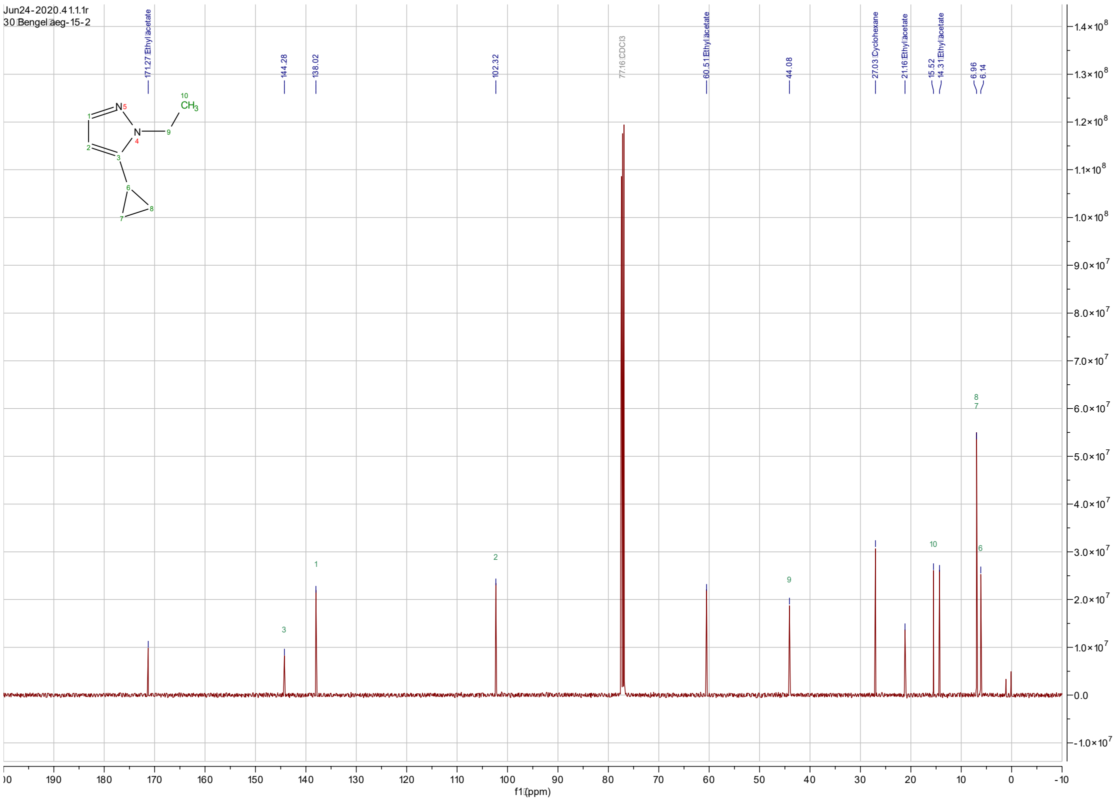

1-propyl-3-cyclopropylpyrazole (8a):

^{1}H-NMR

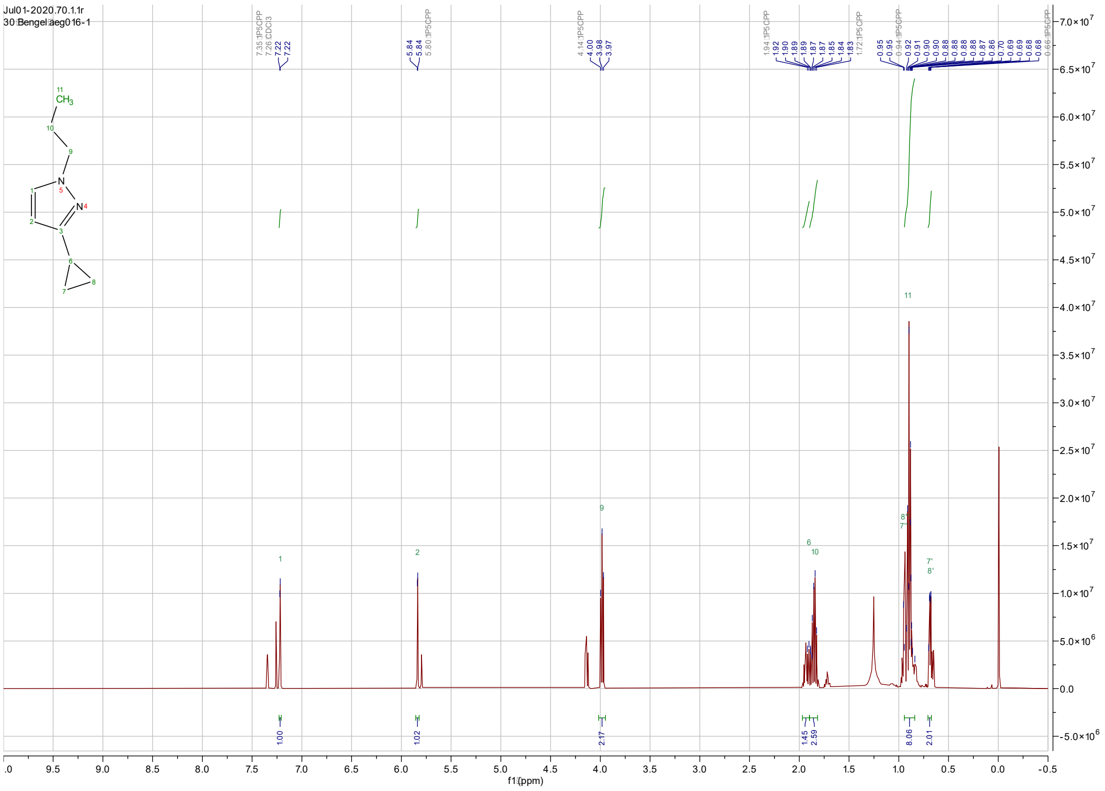

^{13}C-NMR

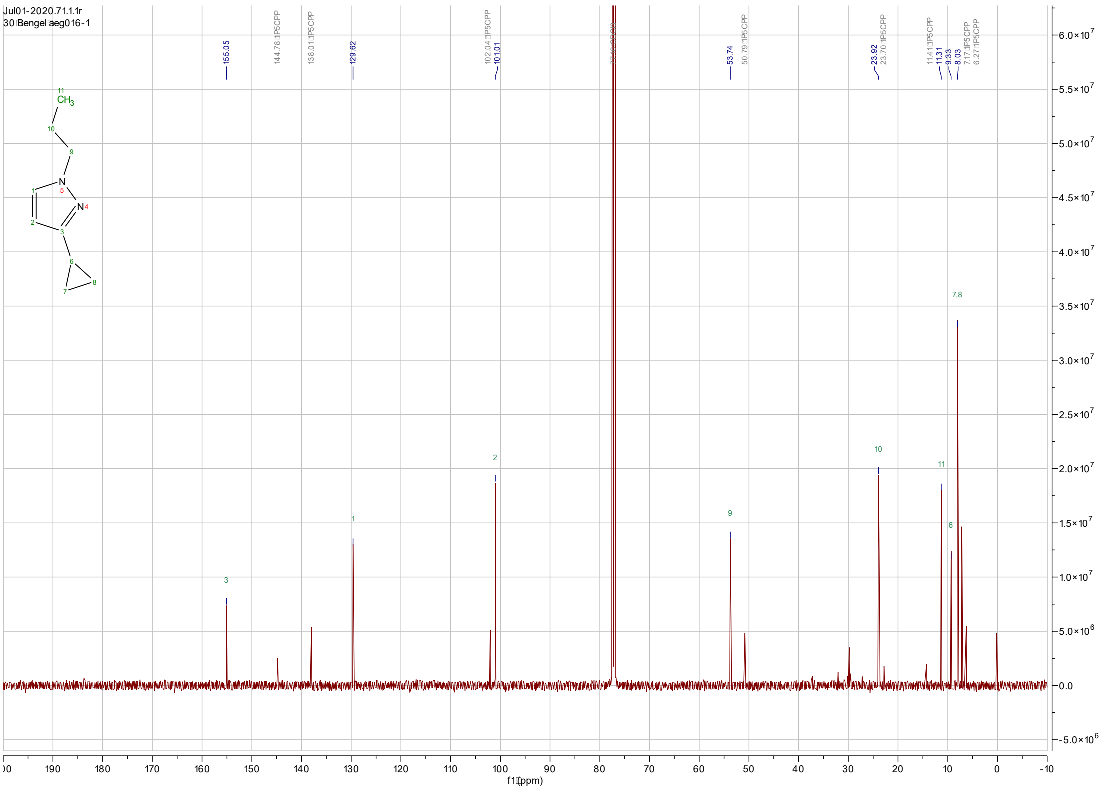

1-propyl-5-cyclopropylpyrazole (8b):

^{1}H-NMR

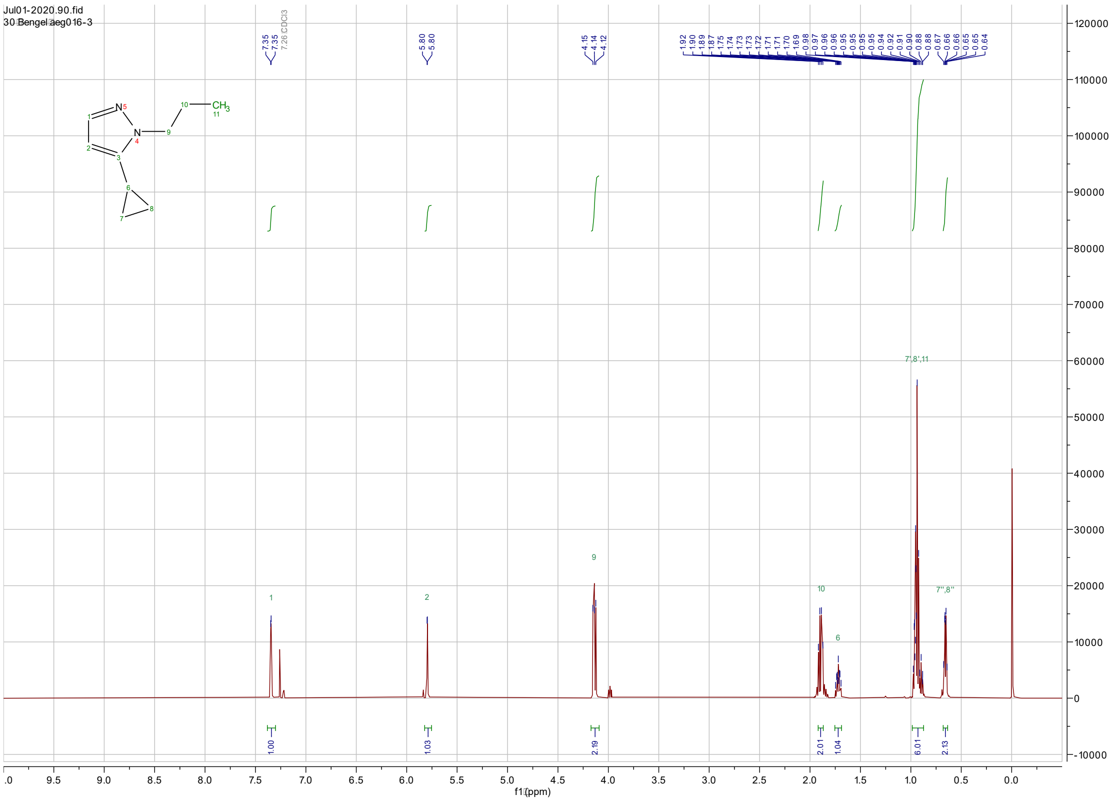

^{13}C-NMR

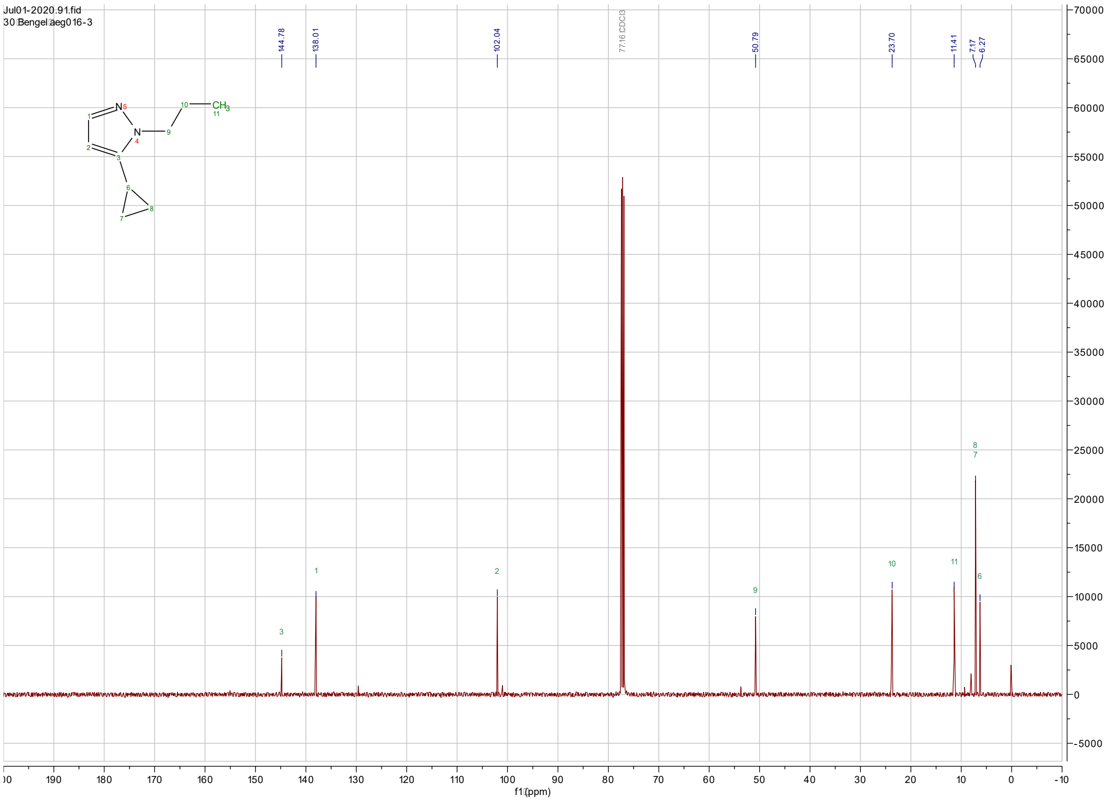

1-methyl-3(2-furyl)pyrazole (4a):

^{1}H-NMR

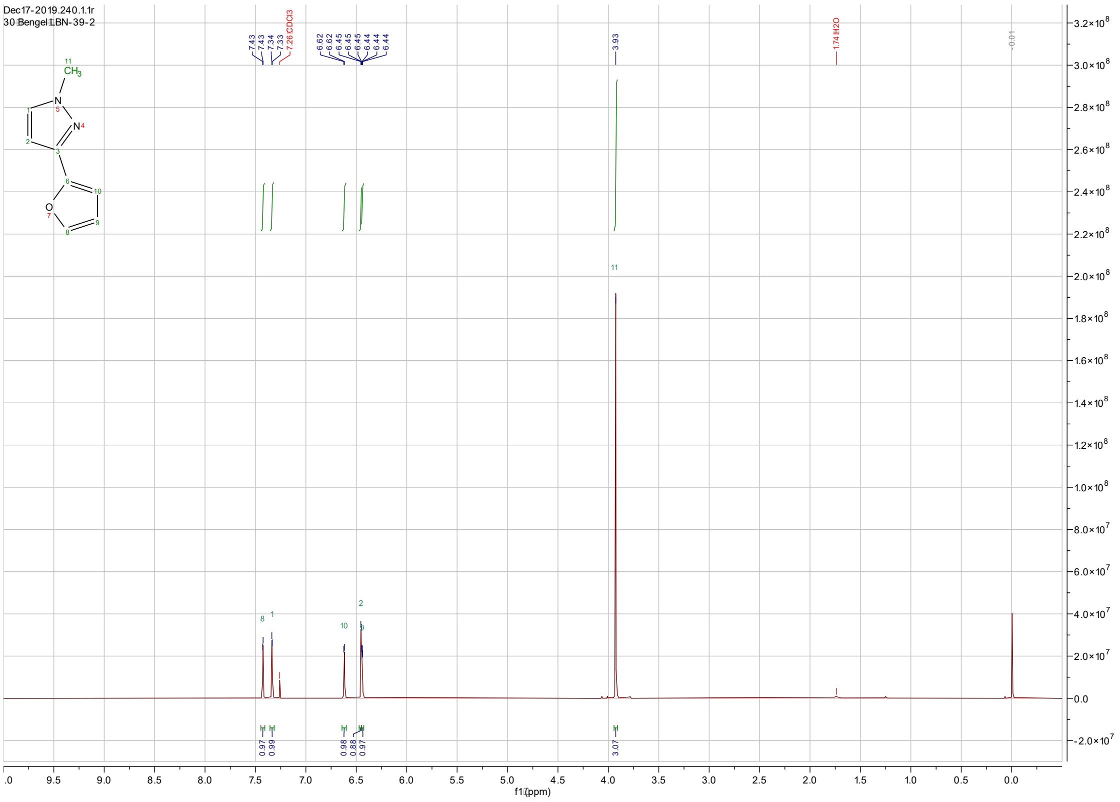

^{13}C-NMR

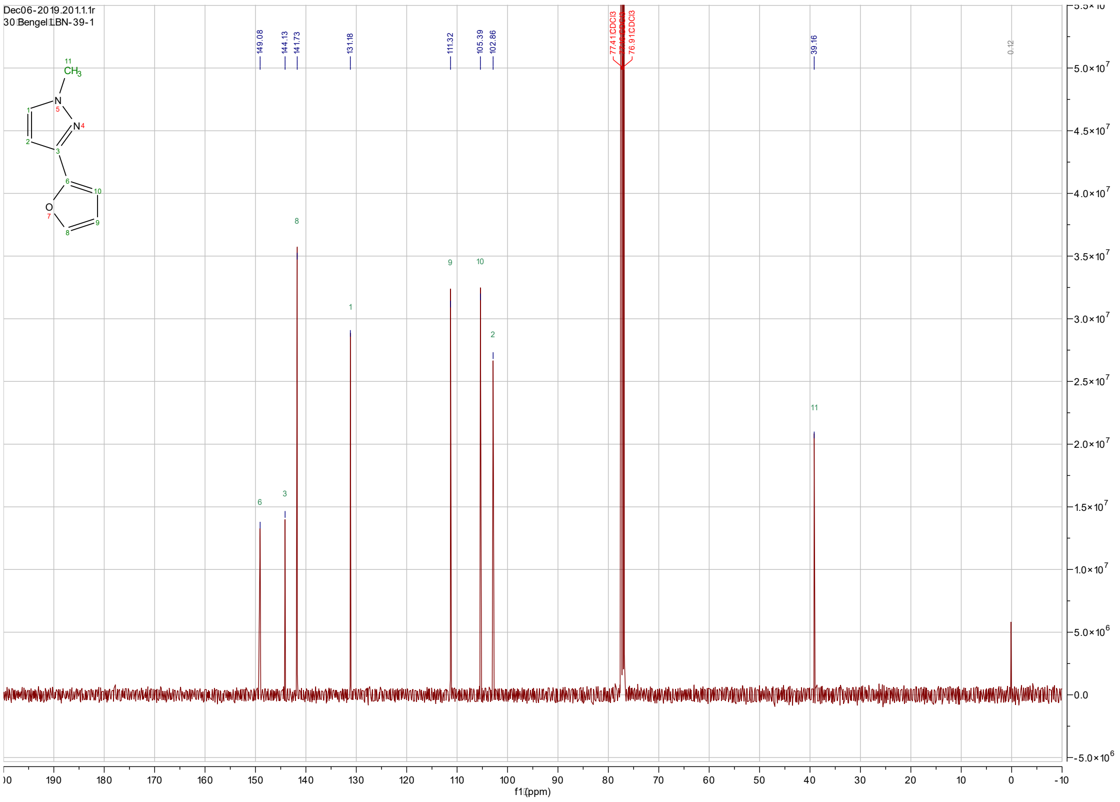

$^{1}H,^{13}C$-HSQC-NMR

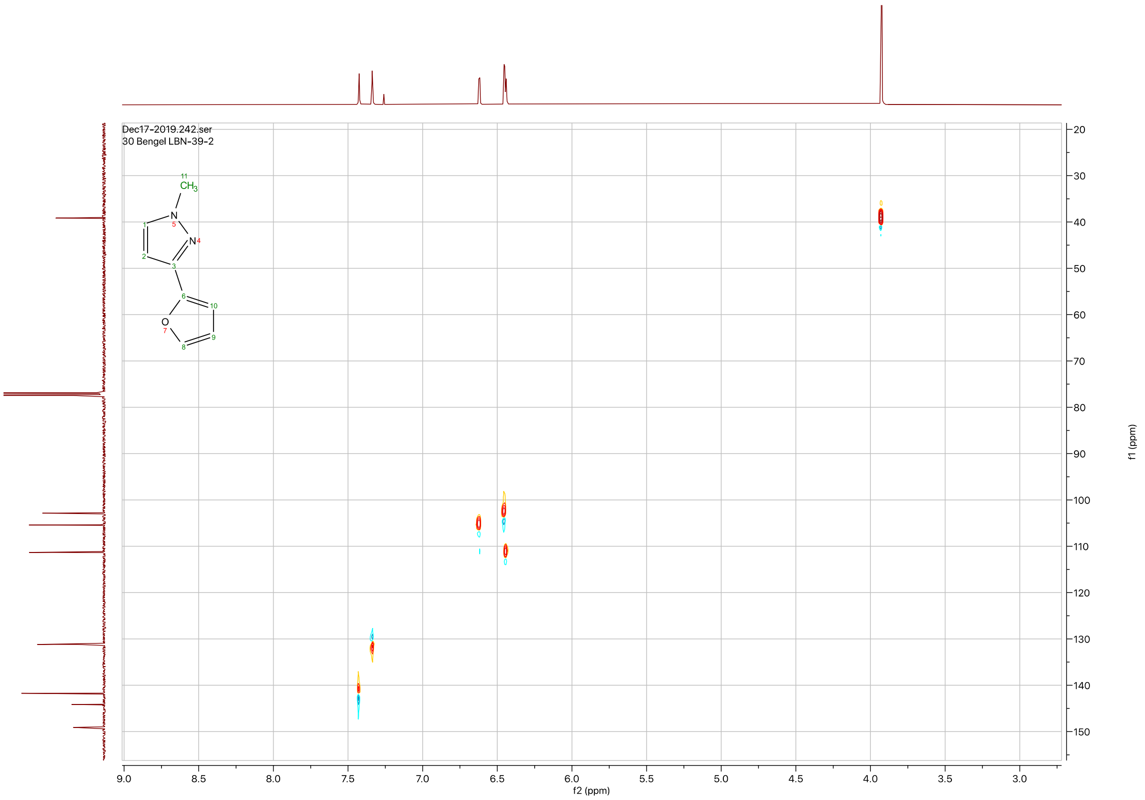

$^{1}H,^{13}C$-HMBC-NMR

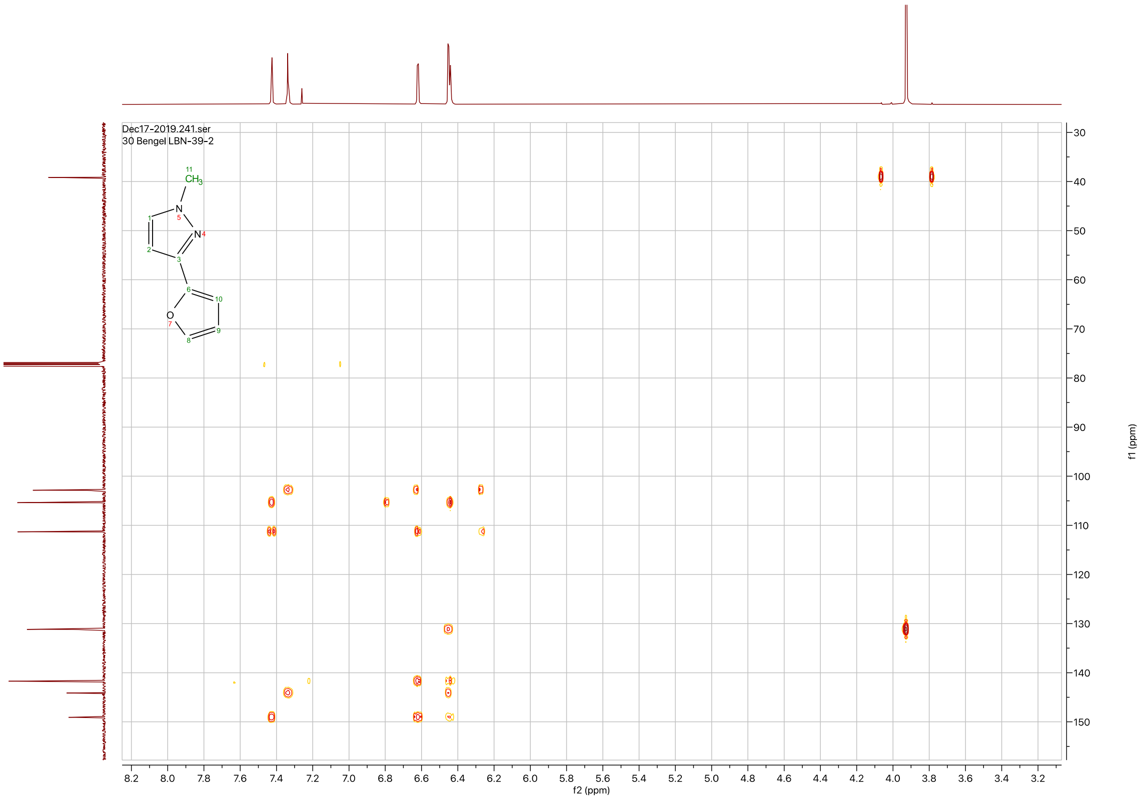

1-methyl-5(2-furyl)pyrazole (4b):

^{1}H-NMR

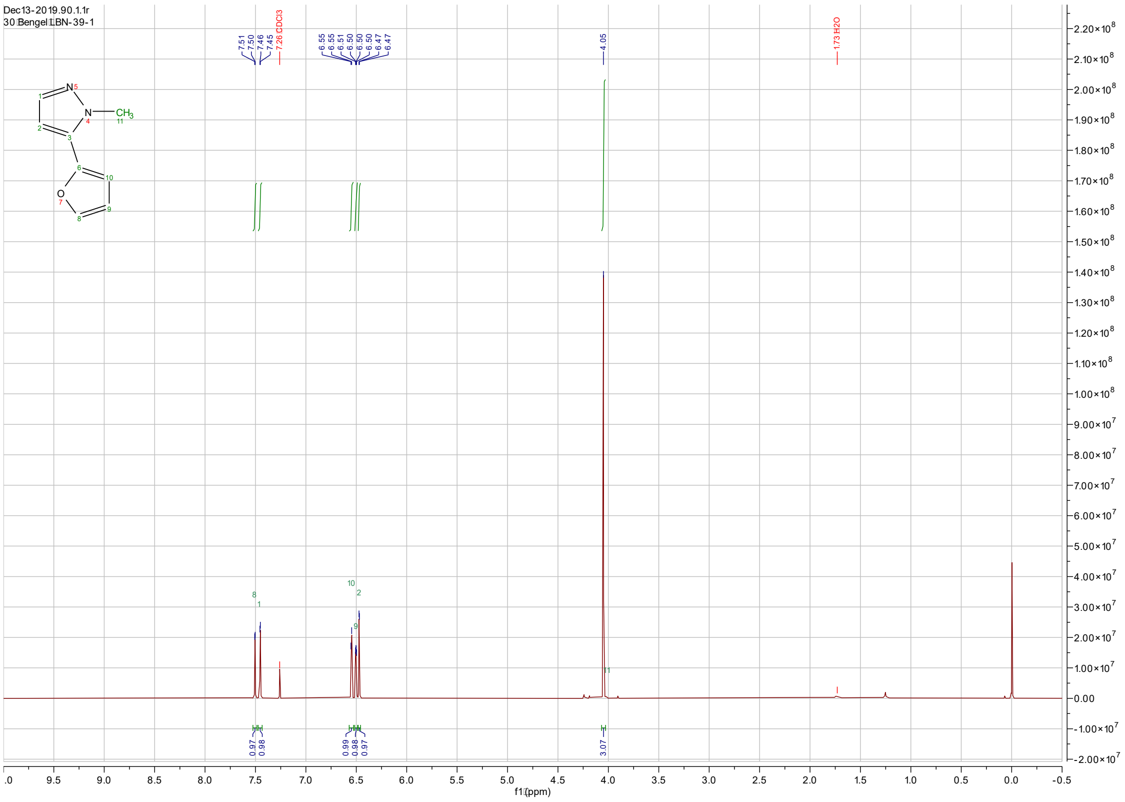

^{13}C-NMR

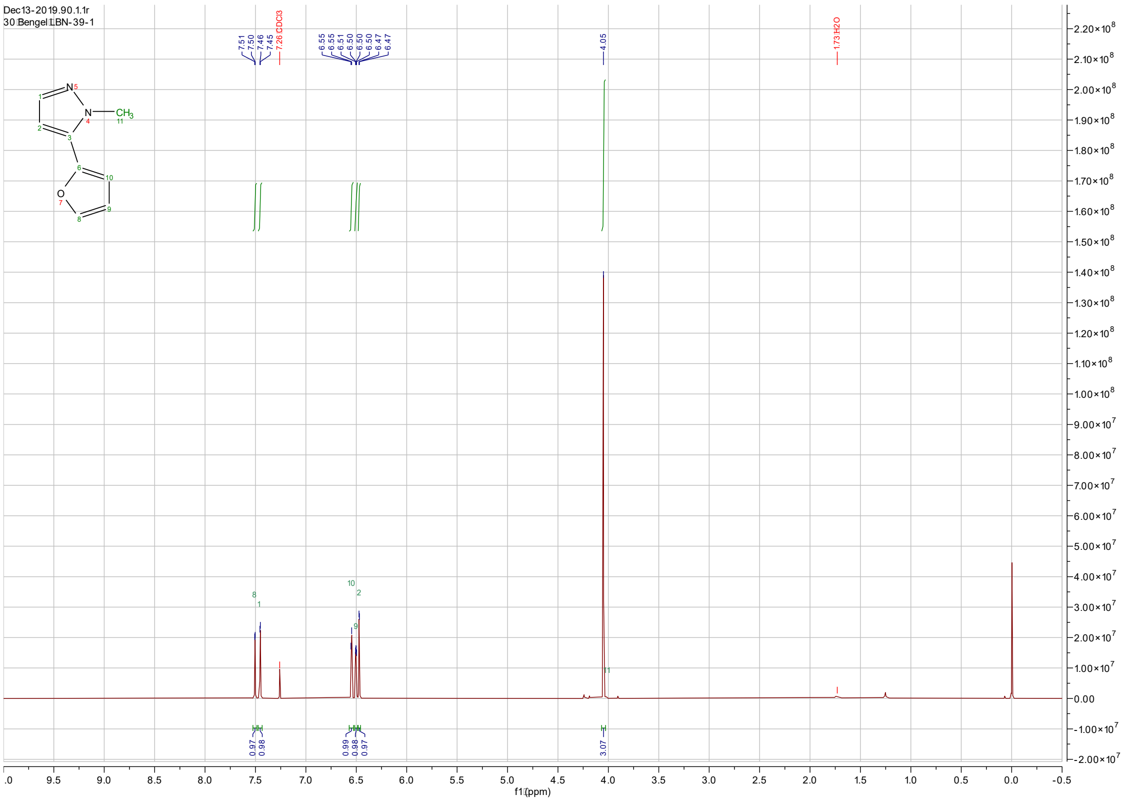

$^1H,^{13}C$-HSQC-NMR

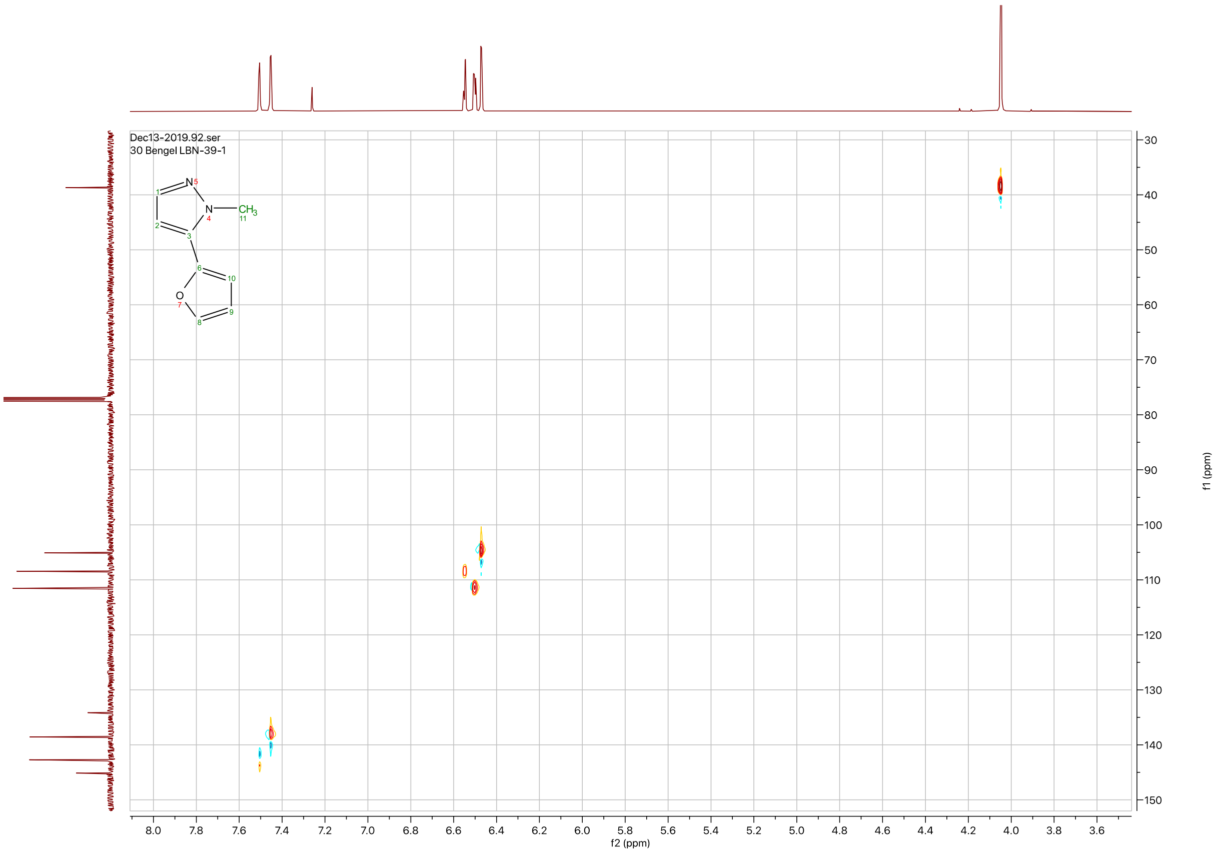

$^1H,^{13}C$-HMBC-NMR

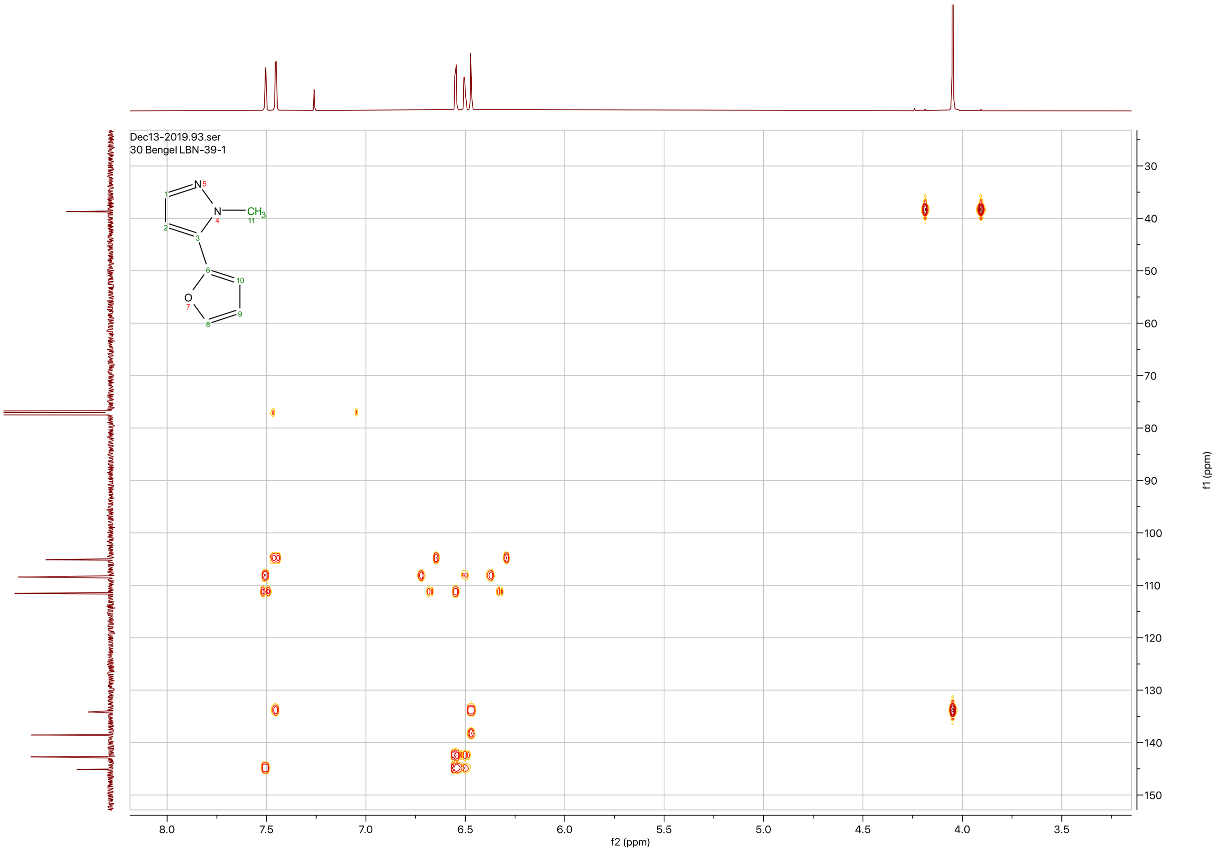

1-methyl-3(2-fluorophenyl)pyrazole (5a):

^{1}H-NMR

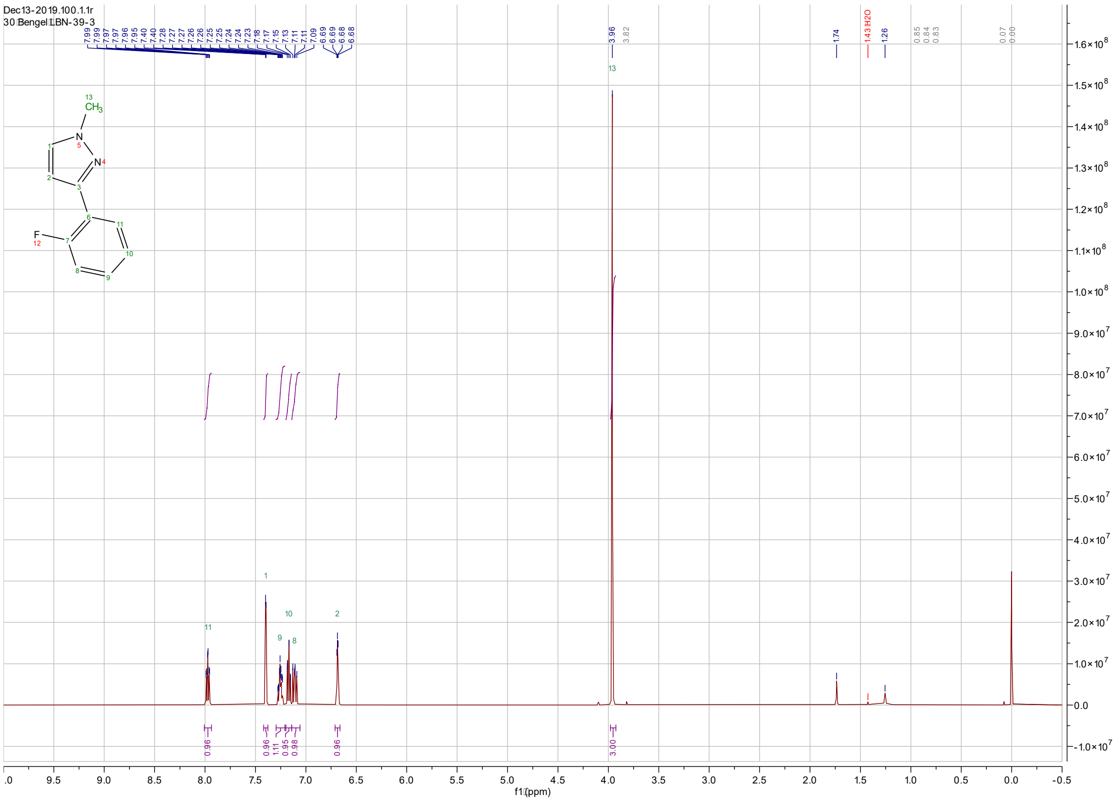

^{13}C-NMR

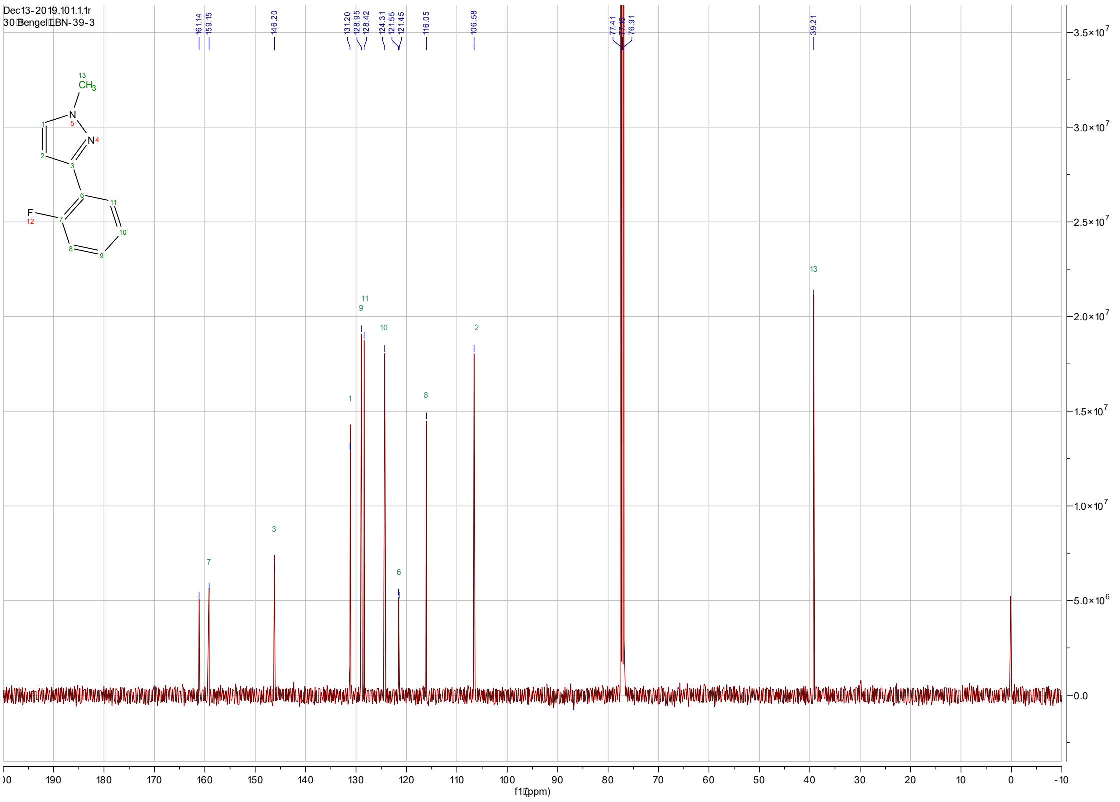

$^{1}H,^{13}C$-HSQC-NMR

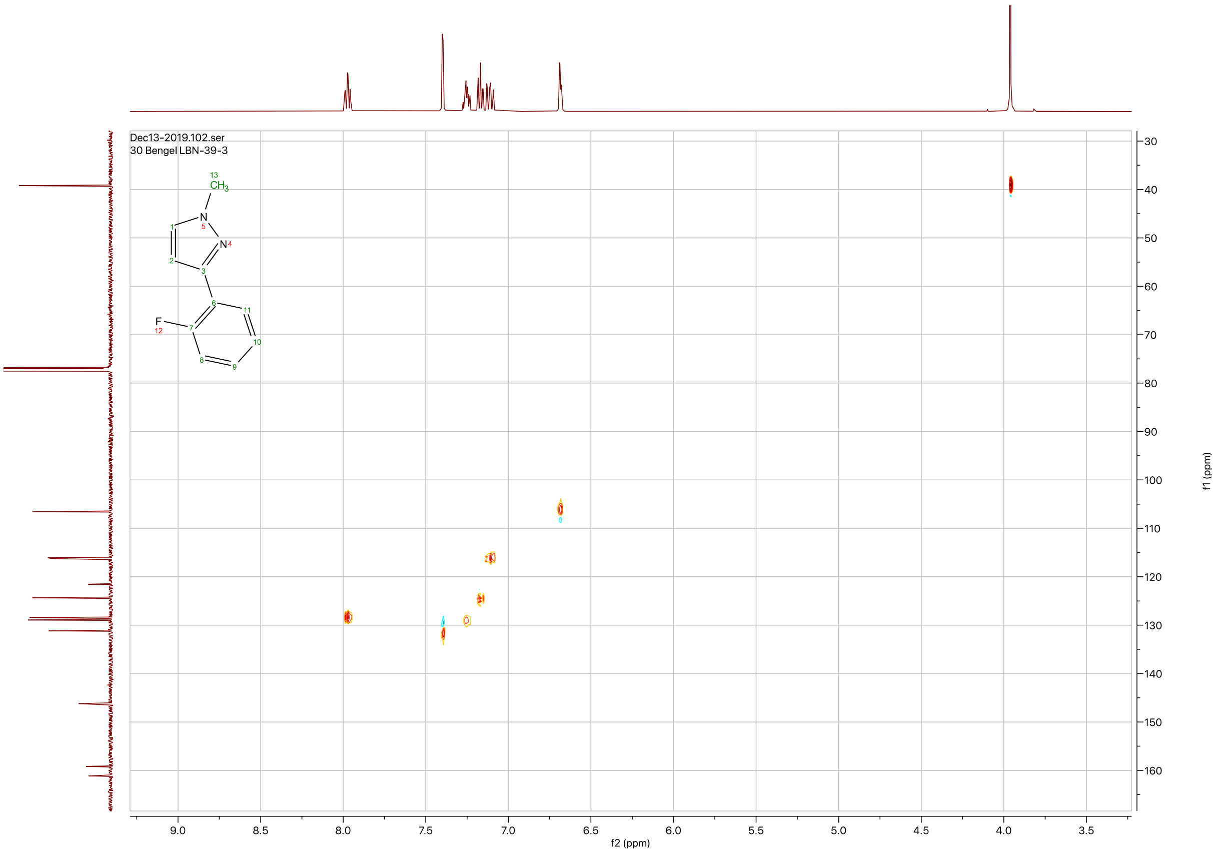

$^{1}H,^{13}C$-HMBC-NMR

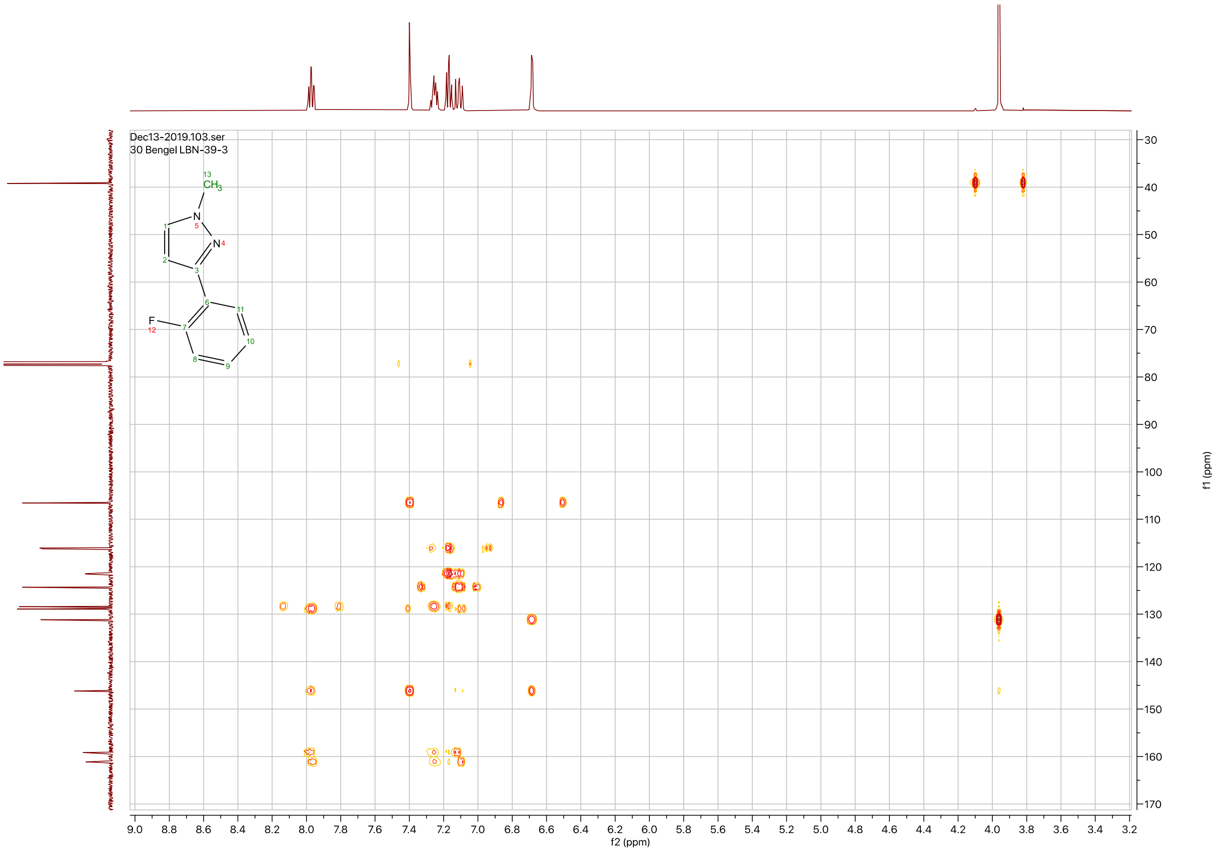

^{1}H, ^{1}H-COSY-NMR

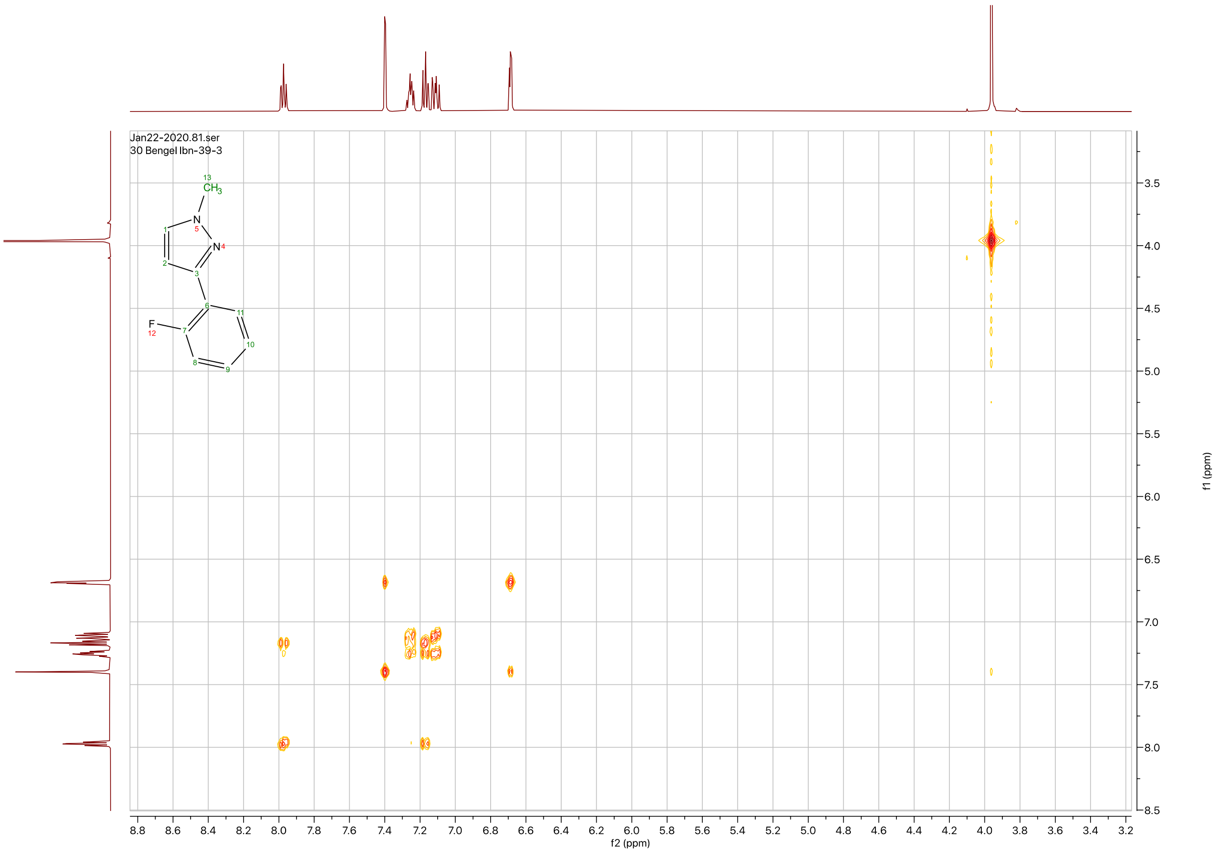

Mixture of 1-methyl-3(2-fluorophenyl)pyrazole (5a, 76%) and 1-methyl-5(2-fluorophenyl)pyrazole (5b, 24%):

^{1}H-NMR

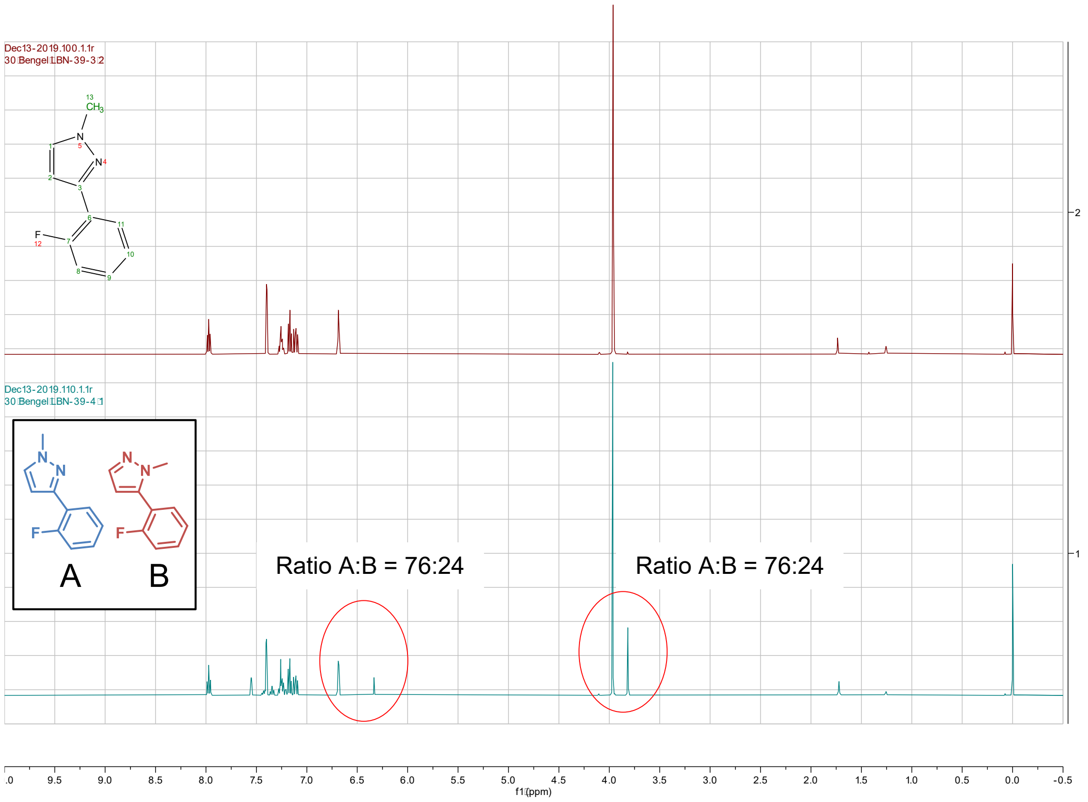

1,5-dimethyl-4-phenylpyrazole (6b):

^{1}H-NMR

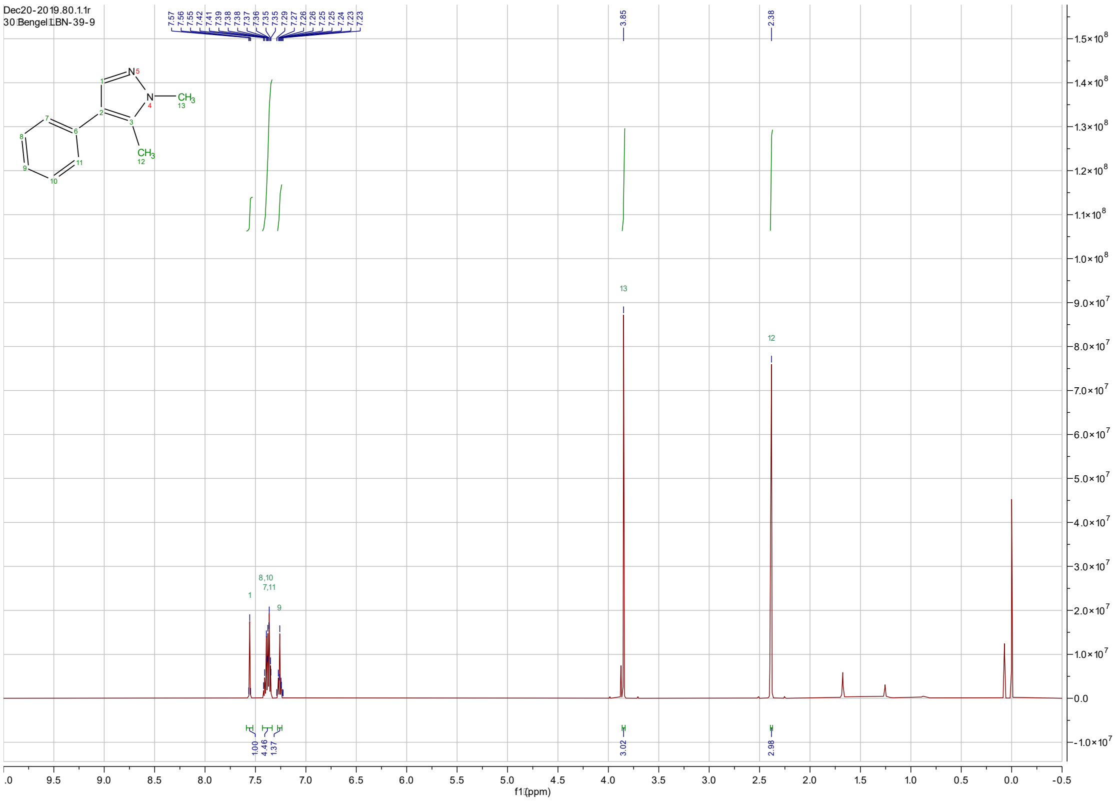

^{13}C-NMR

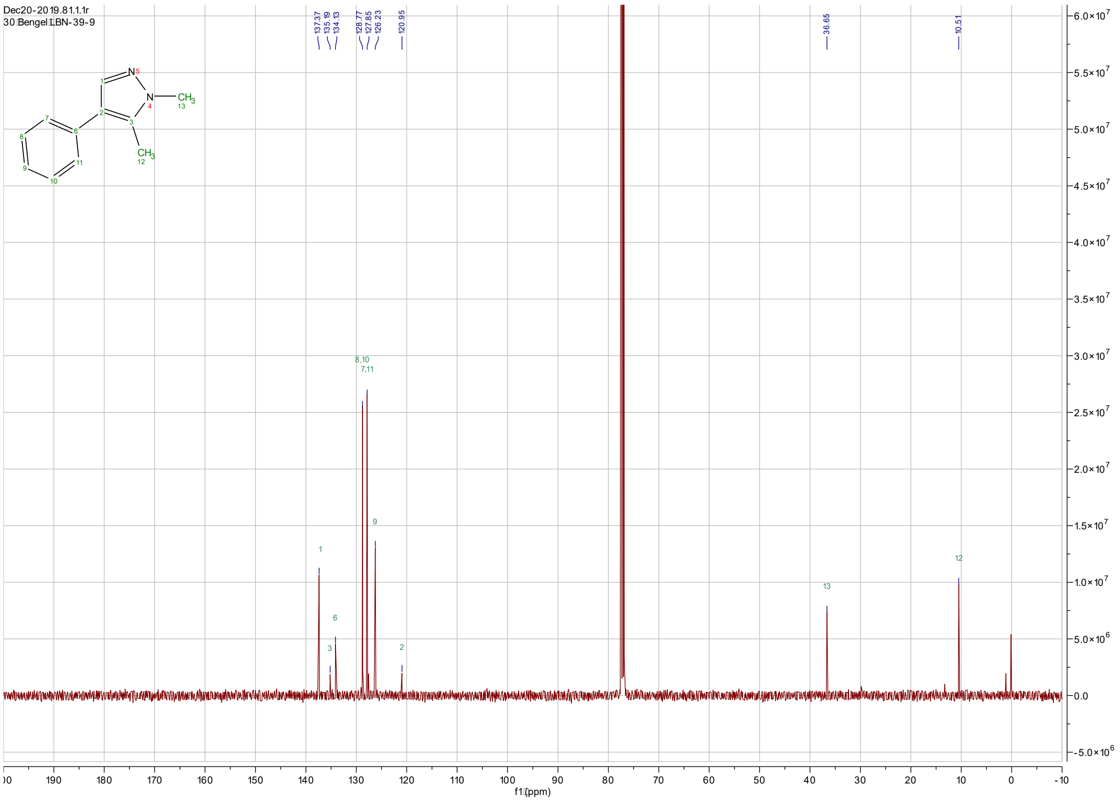

$^{1}H,^{13}C$-HSQC-NMR

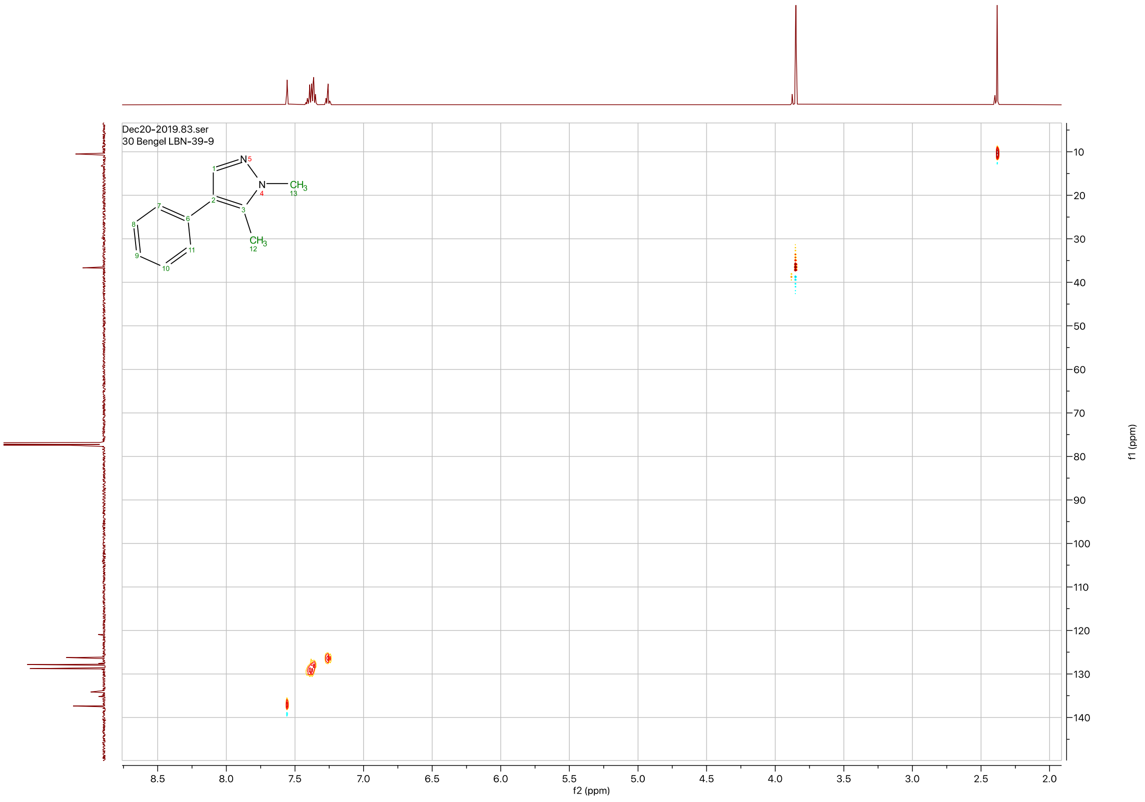

$^{1}H,^{13}C$-HMBC-NMR

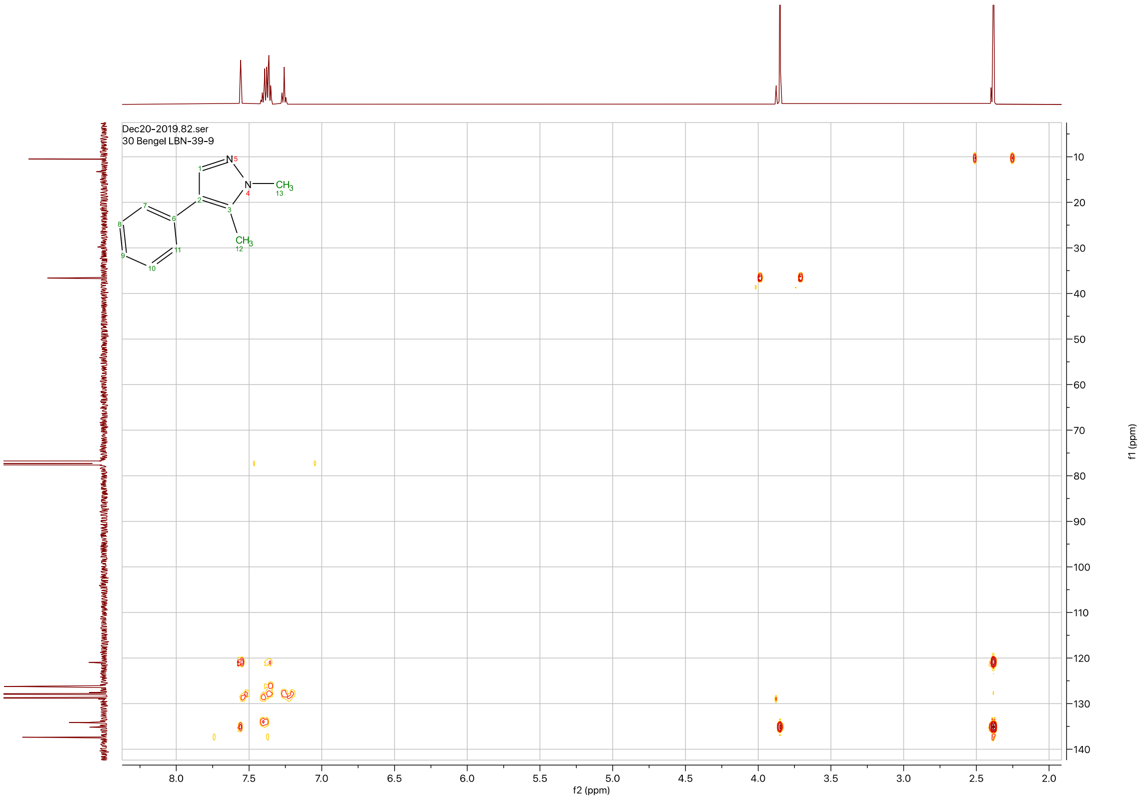

Mixture of 1,5-dimethyl-4-phenyl-pyrazole (6b, 58%) and 1,3-dimethyl-4-phenyl-pyrazole (6a, 24%):

^{1}H-NMR

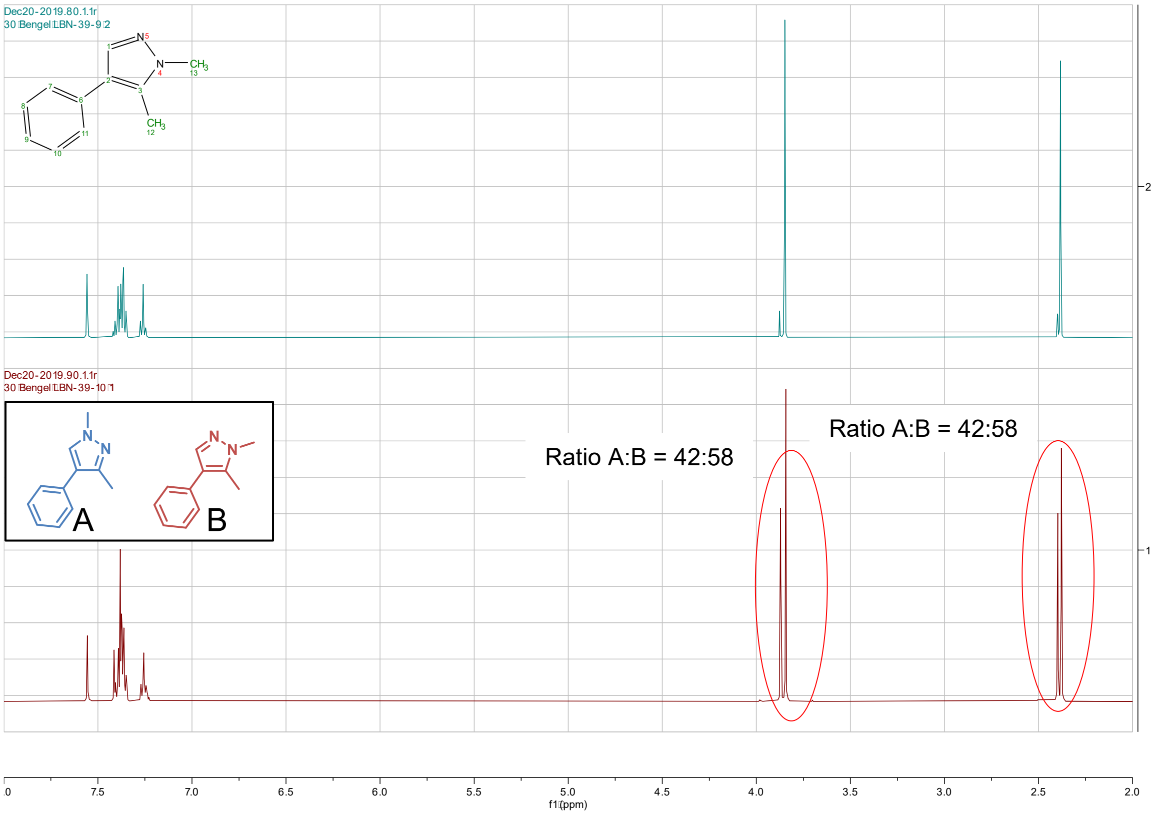

VII. NMR analysis of enzymatic scale-up methylation of 3-cycloproyplpyrazole (3)

1-methyl-5-cyclopropylpyrazole (3b):

NMR Data: ^{1}H NMR (500 MHz, $CDCl_3$) δ 7.33 (d, *J* = 1.8 Hz, 1H), 5.82 (d, *J* = 1.7 Hz, 1H), 3.89 (s, 3H), 1.71 (m, 1H), 0.96 (m, 2H), 0.65 (m, 2H). ^{13}C NMR (126 MHz, $CDCl_3$) δ 144.96, 137.84, 102.41, 36.18, 6.71 (2C), 6.10. Yield: 44.0 mg (37.3%).

^{1}H-NMR

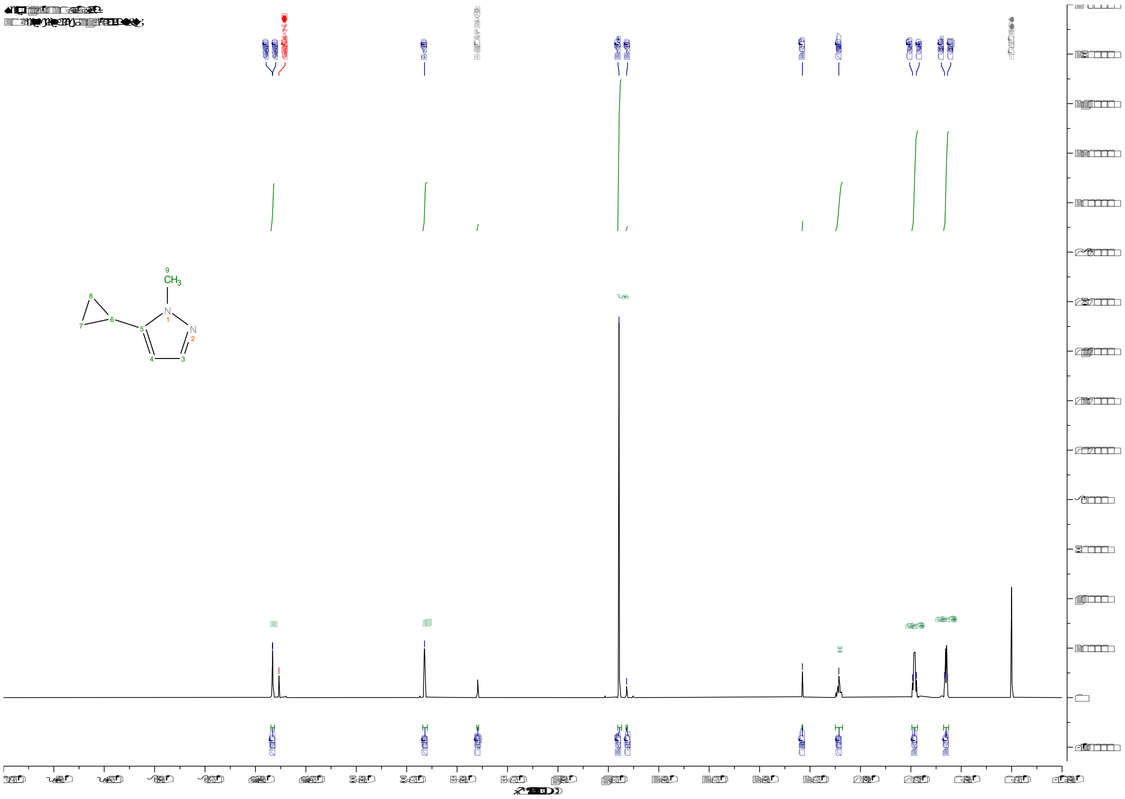

^{13}C-NMR

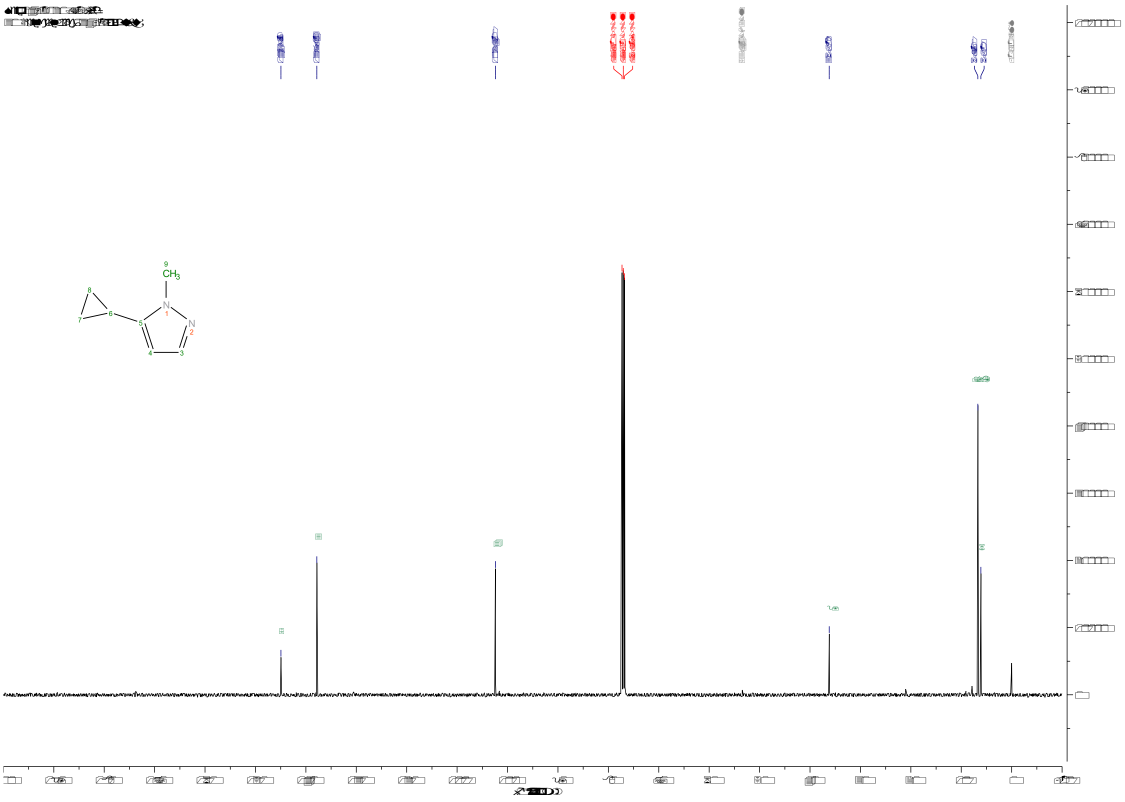

VIII. HR-ESI-MS analysis of product standards.

1,3-dimethylpyrazole (1a):

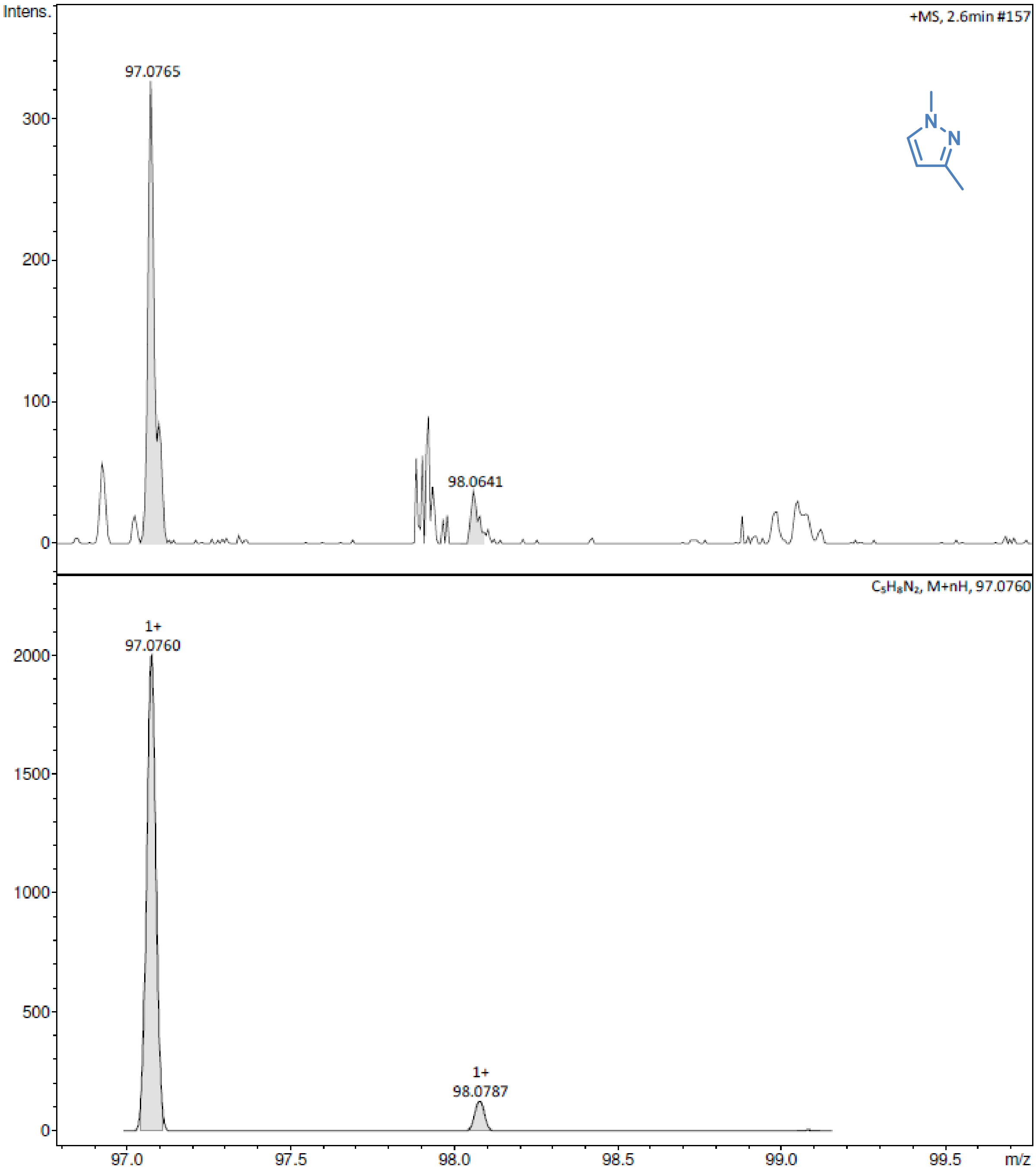

1,5-dimethylpyrazole (1b):

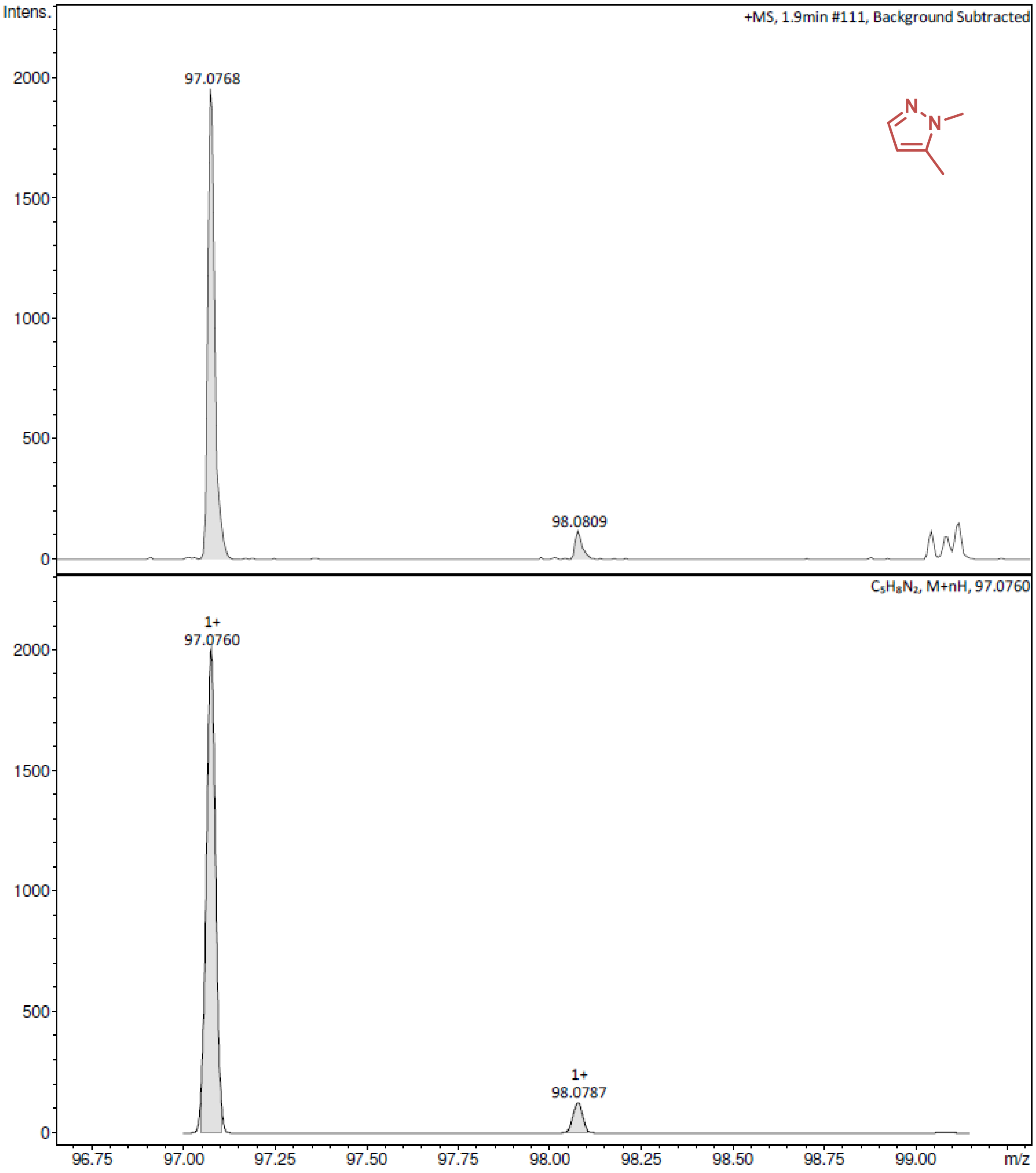

Mixture of 1,3,4-trimethylpyrazole (25.5%) and 1,4,5-trimethylpyrazole (74.5%):

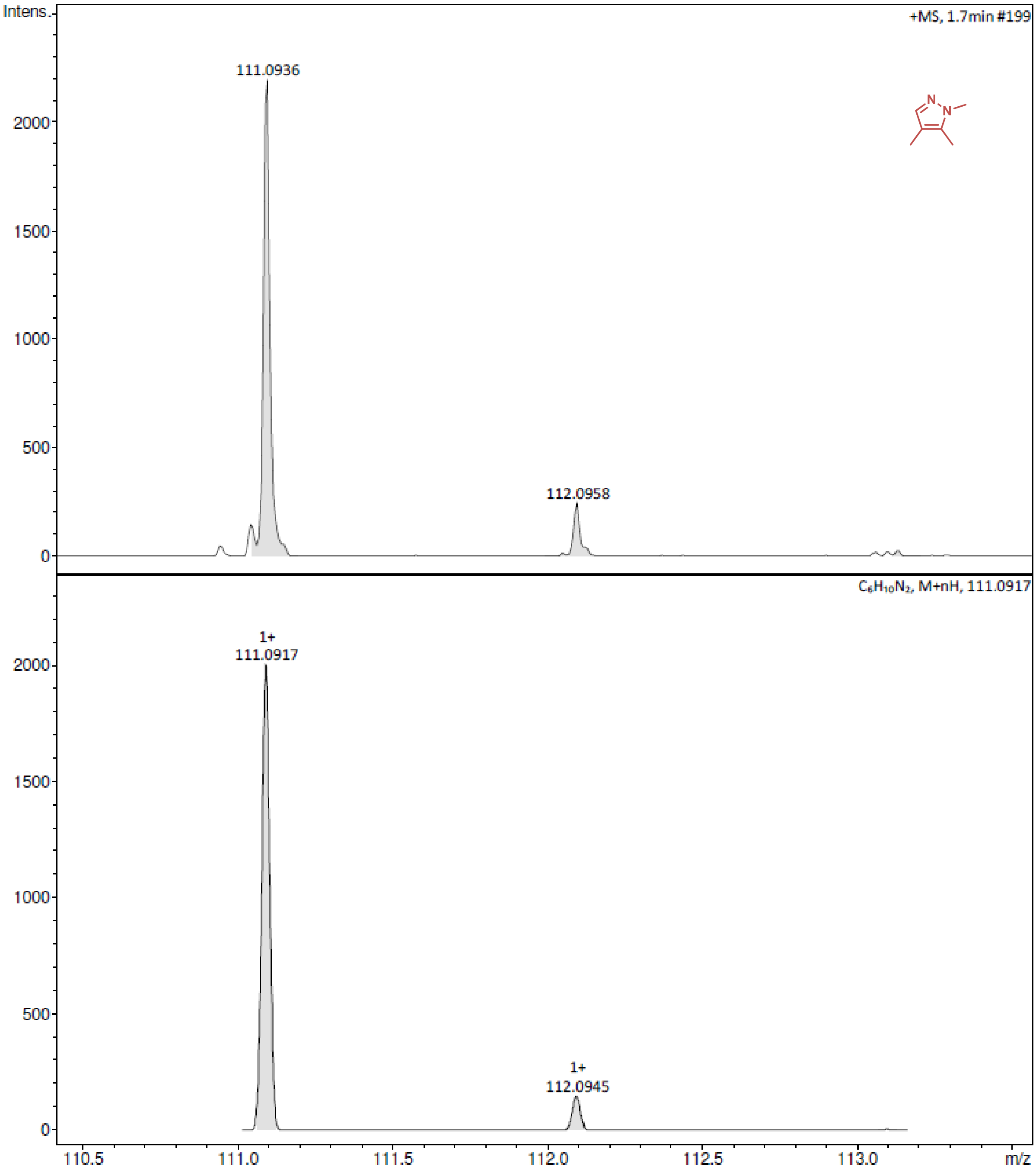

1-methyl-3-cyclopropylpyrazole (3a):

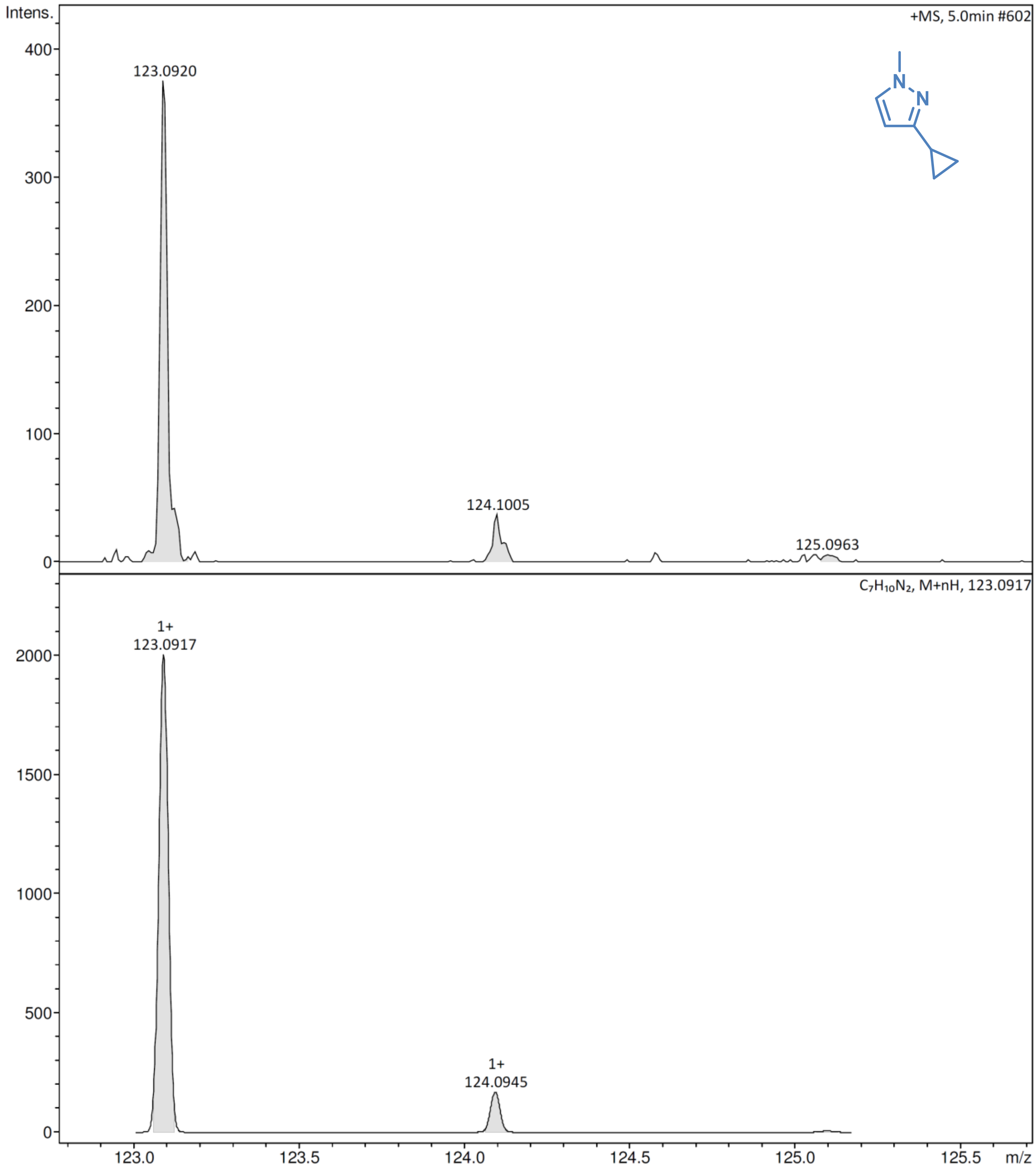

1-methyl-5-cyclopropylpyrazole (3b):

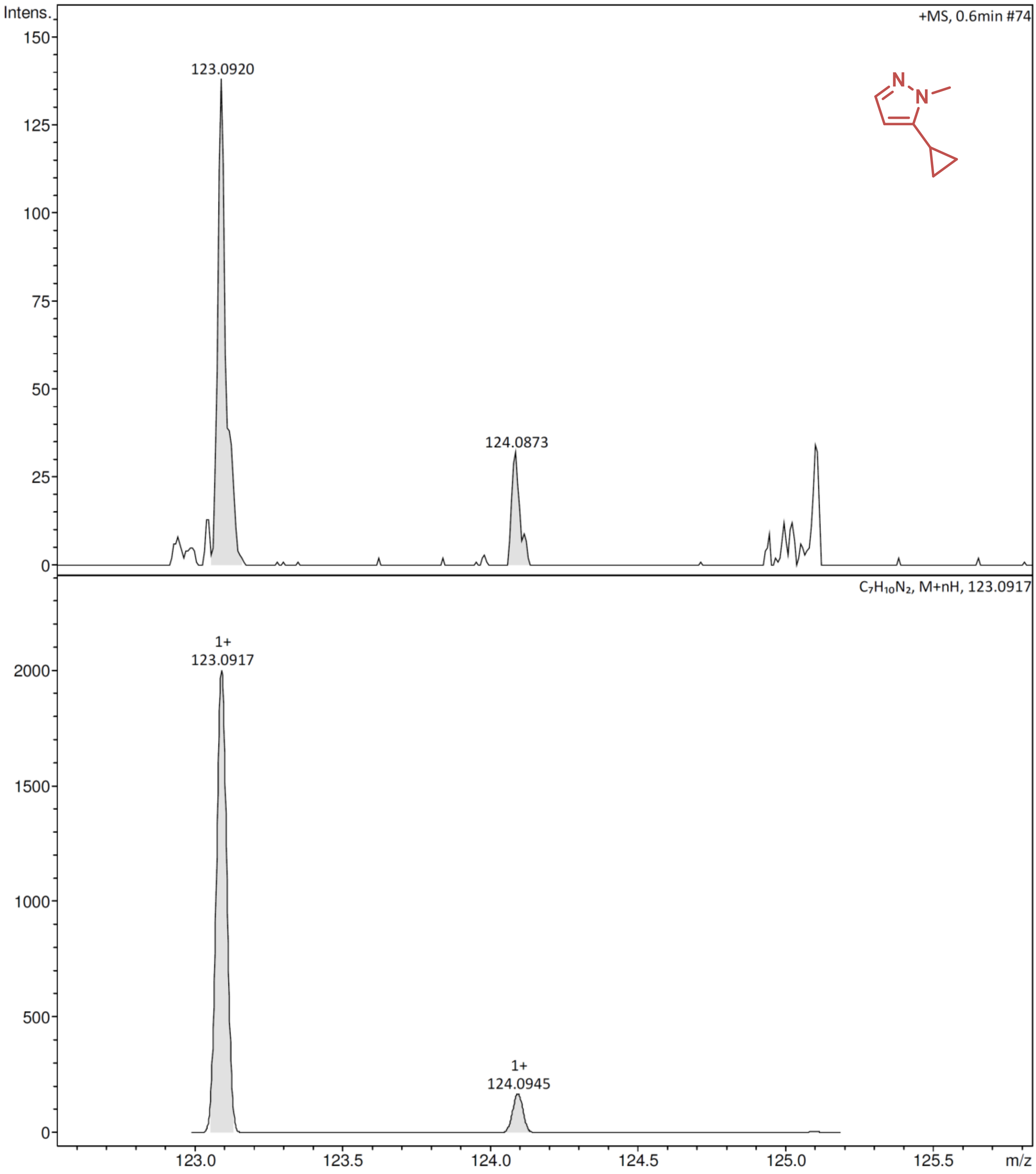

1-ethyl-3-cyclopropylpyrazole (7a):

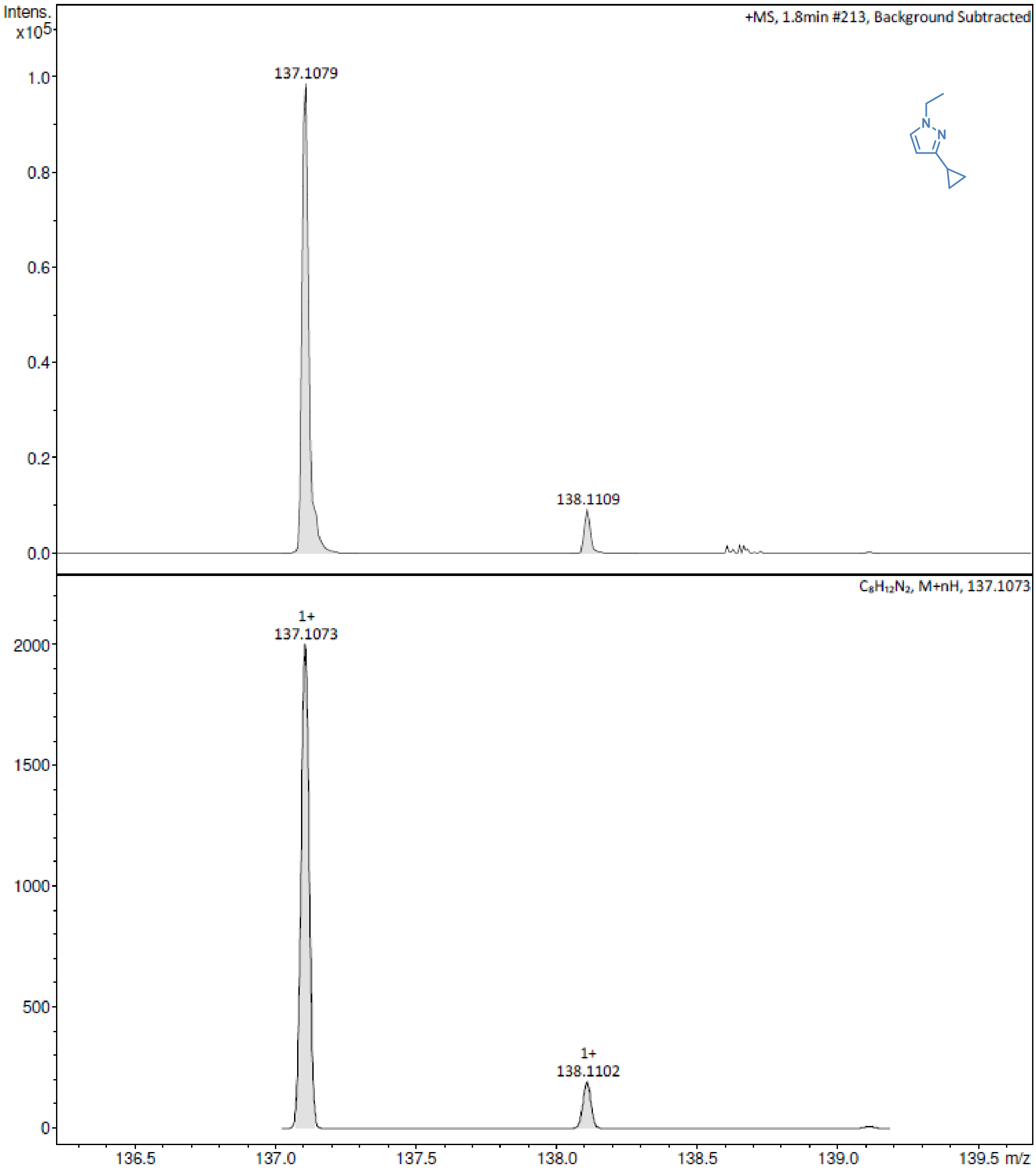

1-ethyl-5-cyclopropylpyrazole (7b):

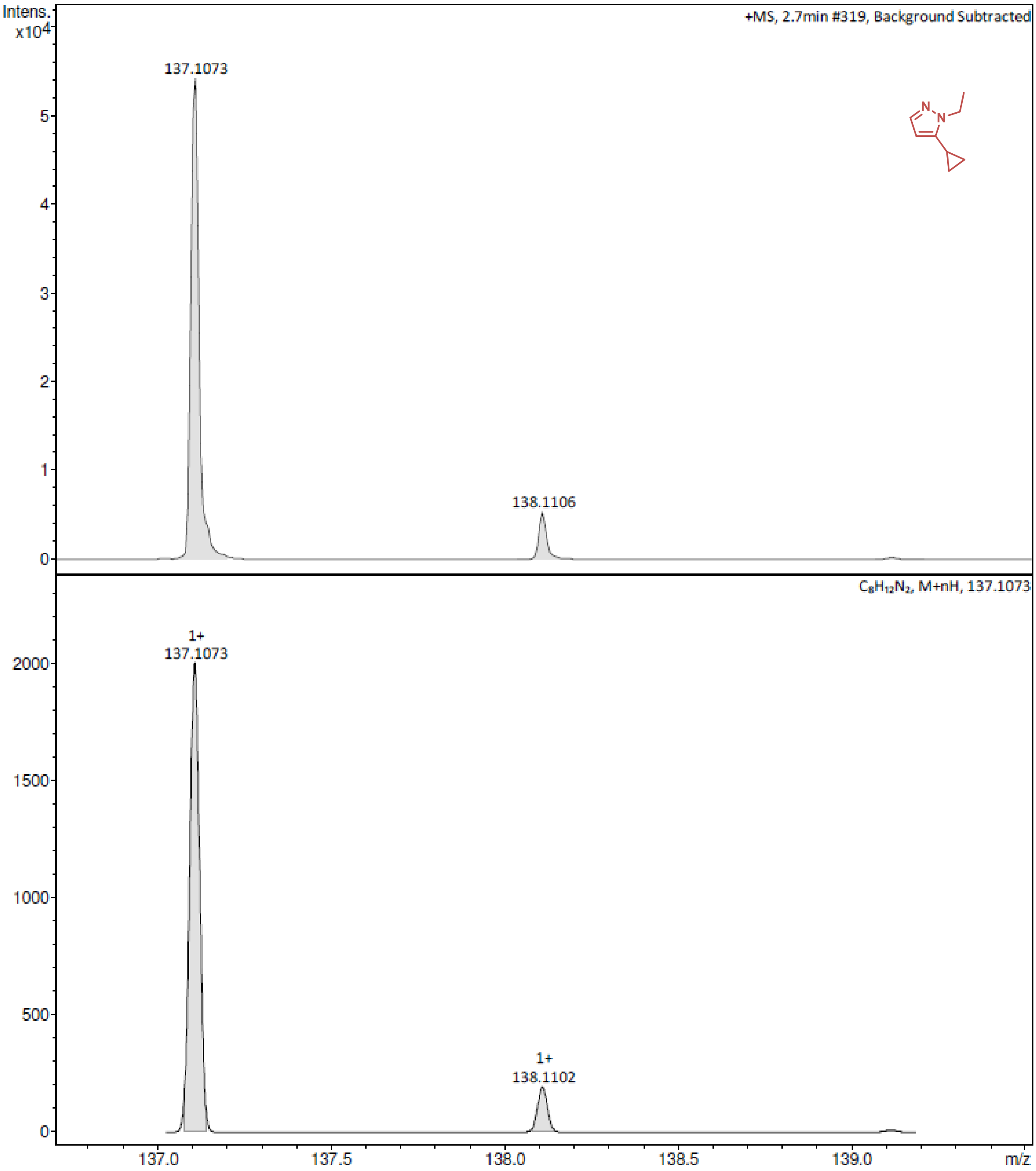

1-propyl-3-cyclopropylpyrazole (8a):

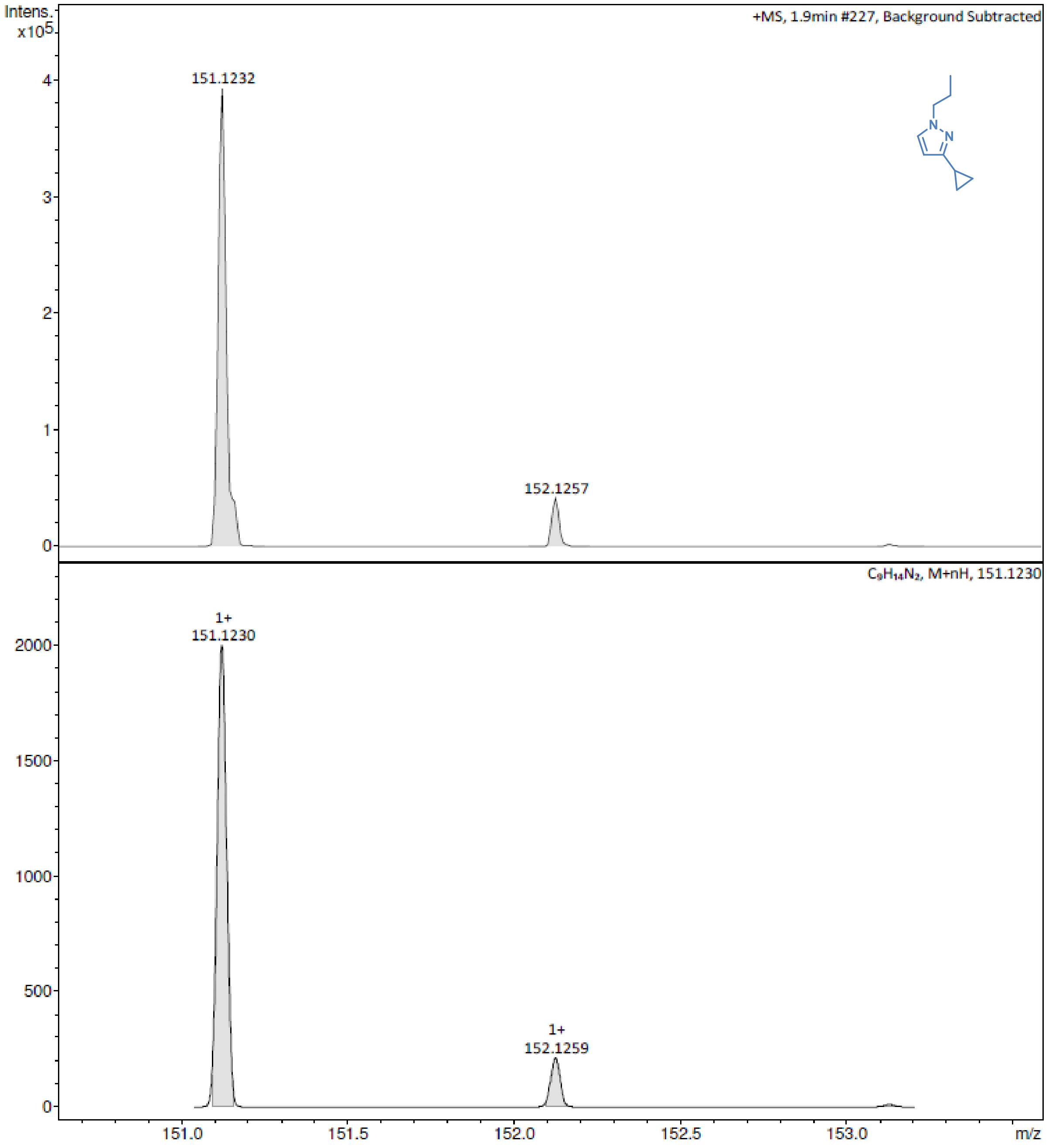

1-propyl-5-cyclopropylpyrazole (8b):

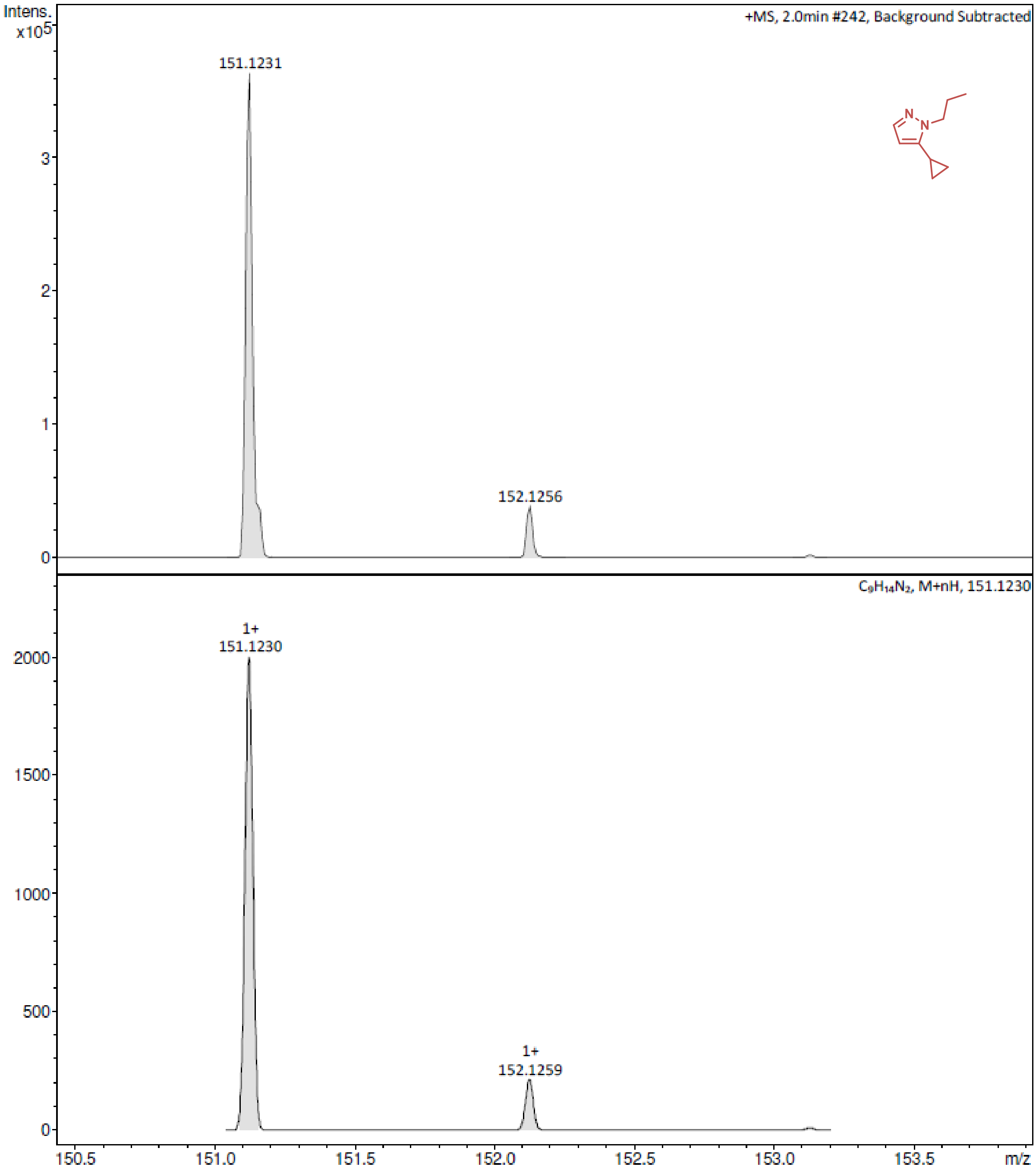

1-methyl-3(2-furyl)pyrazole (4a):

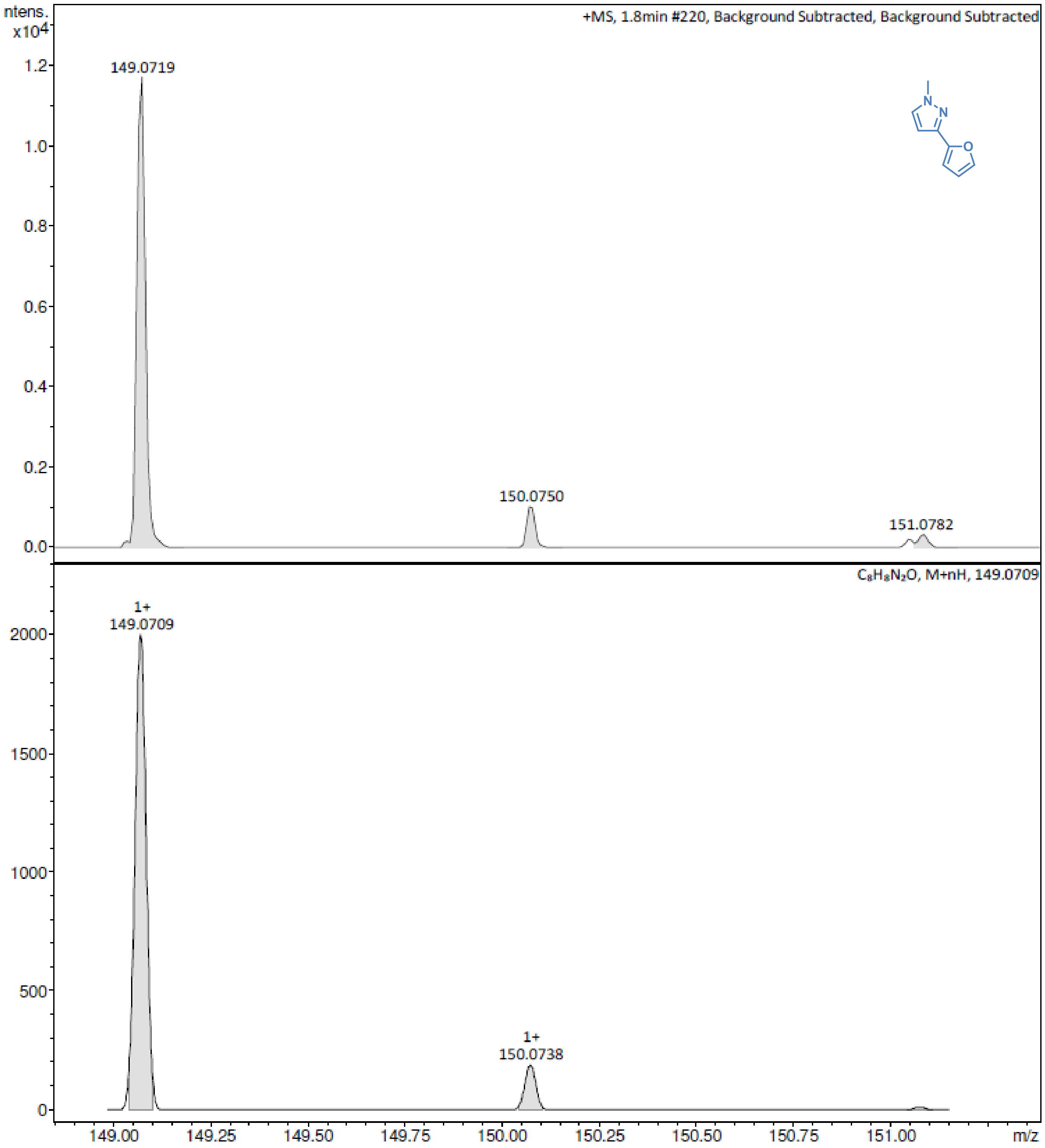

1-methyl-5(2-furyl)pyrazole (4b):

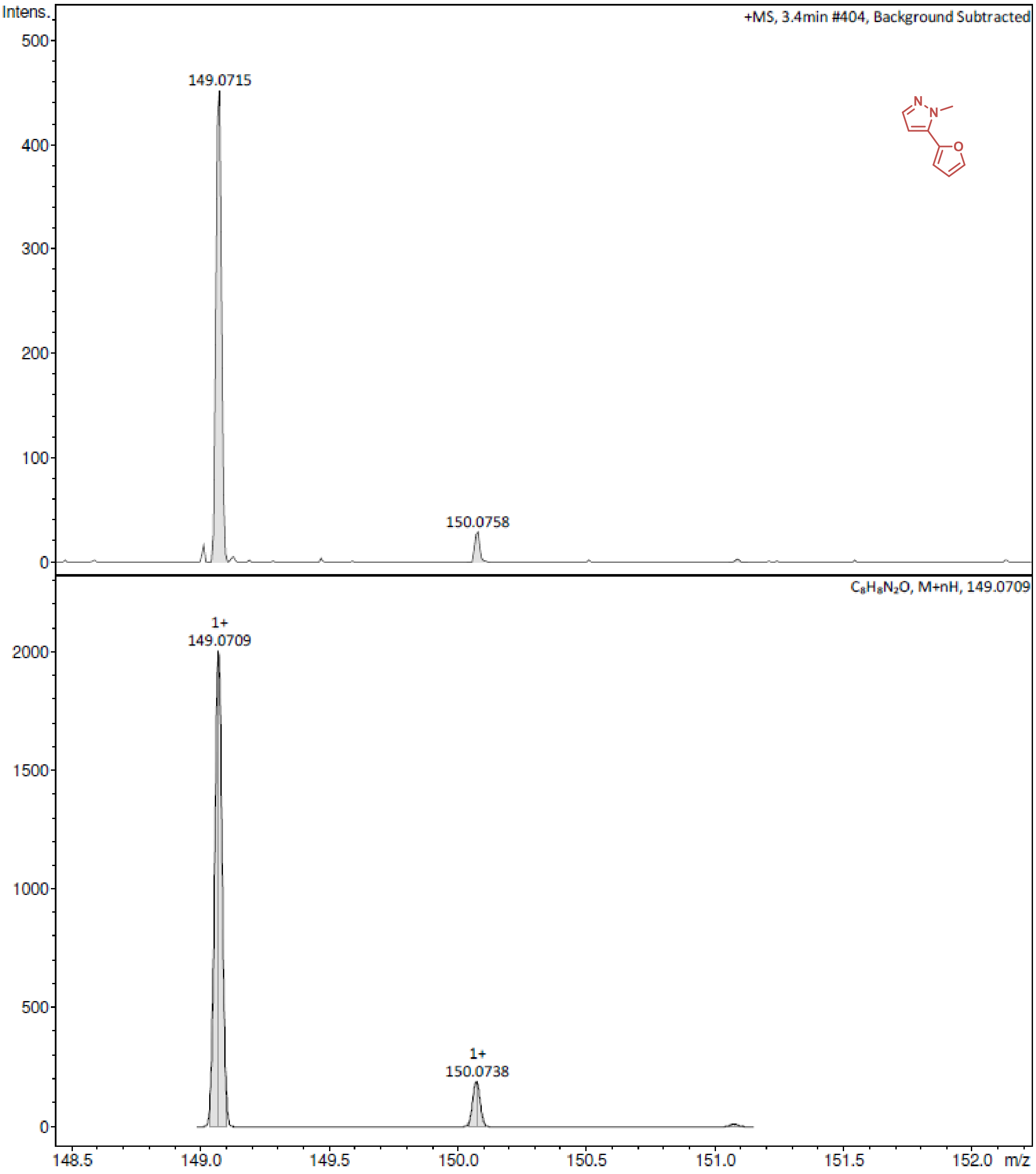

1-methyl-3(2-fluorophenyl)pyrazole (5a):

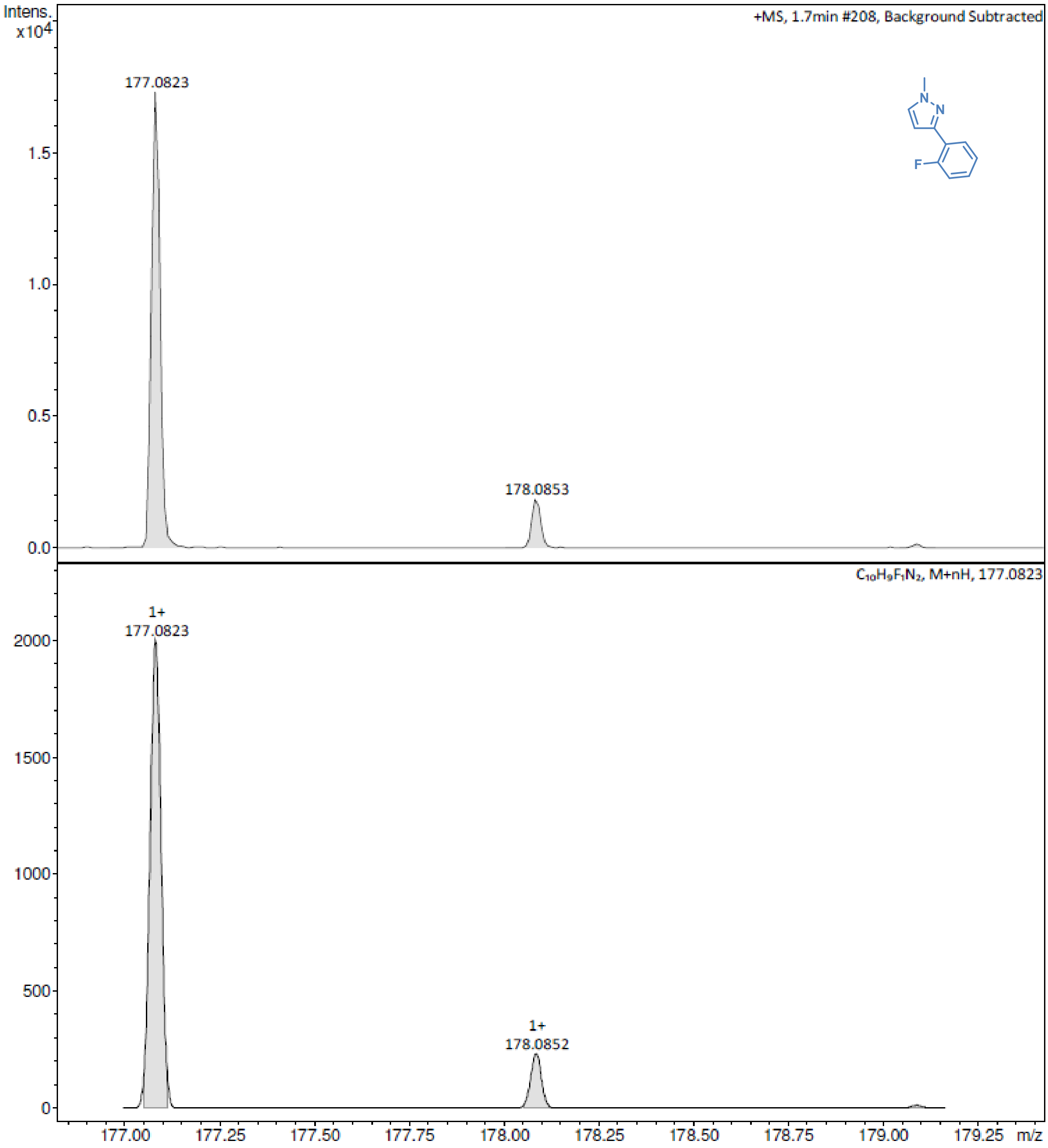

Mixture of 1-methyl-3(2-fluorophenyl)pyrazole (5a, 76%) and 1-methyl-5(2-fluorophenyl)pyrazole (5b, 24%):

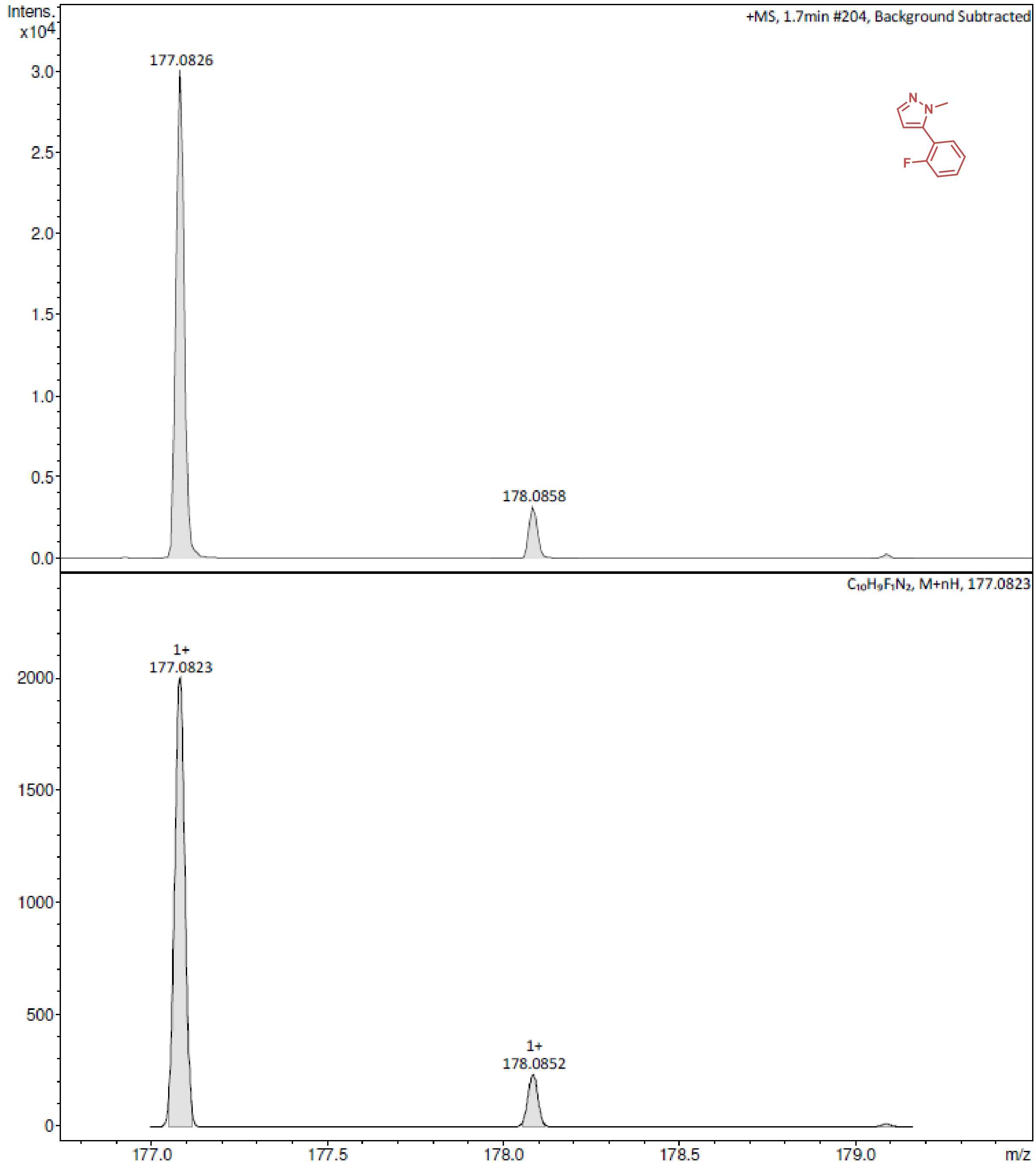

1,5-dimethyl-4-phenylpyrazole (6b):

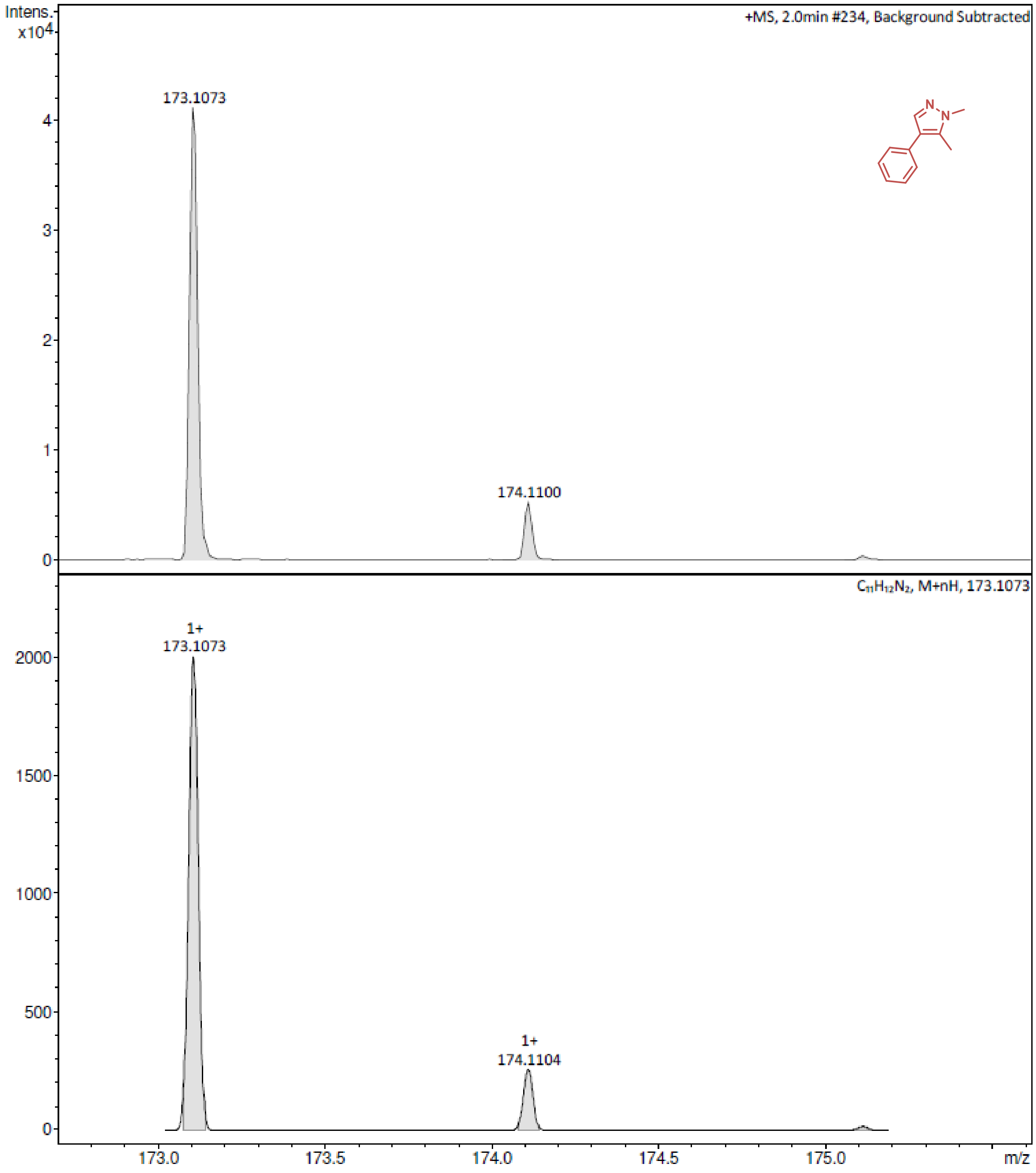

Mixture of 1,5-dimethyl-4-phenyl-pyrazole (6b, 58%) and 1,3-dimethyl-4-phenyl-pyrazole (6a, 24%):

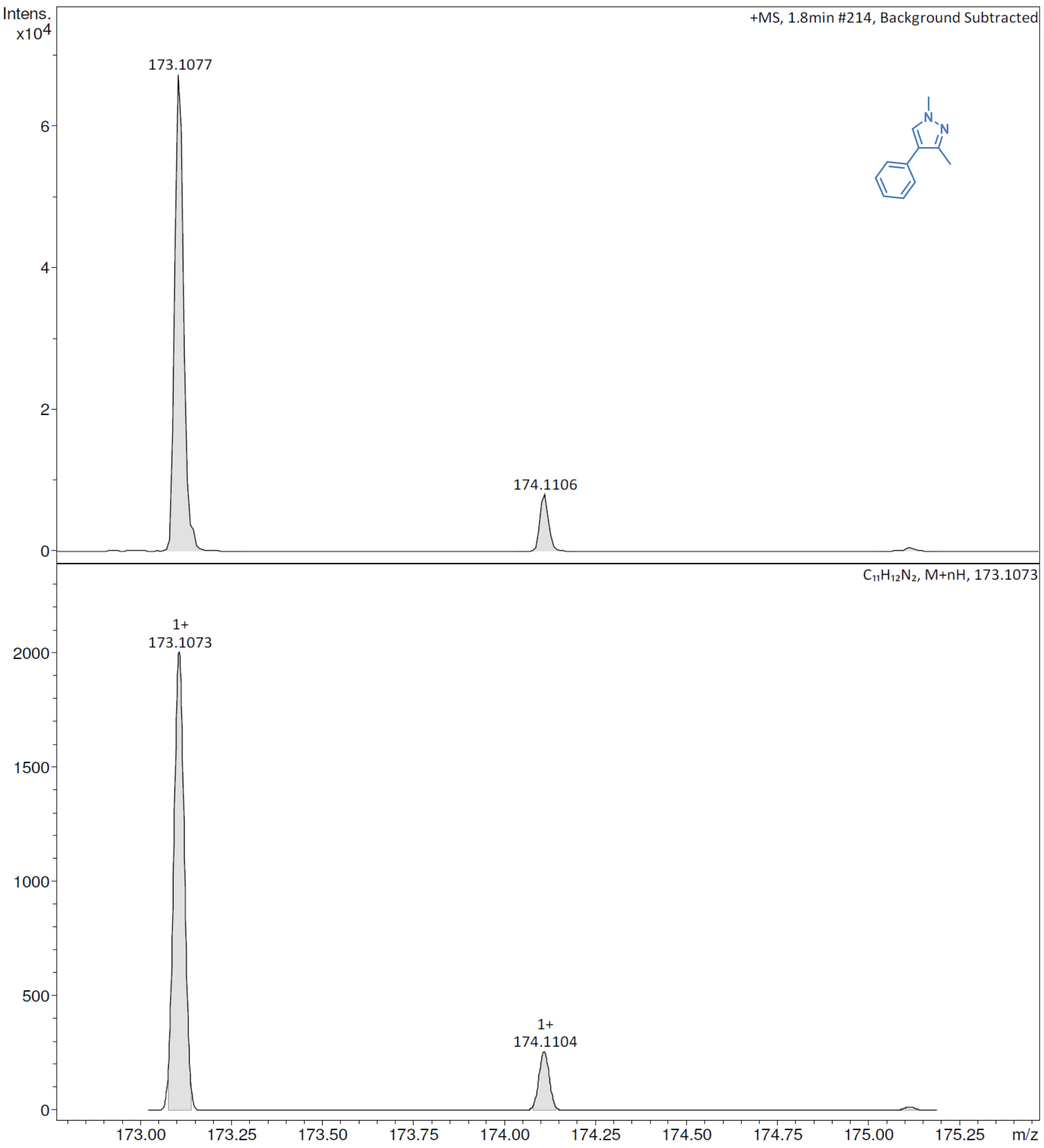

IX. Enzyme sequences

>(wtNMT)
ATGGAGAGTGGATTTACGAGTAAGGACACTTATTTGAGTCATTTTAACCCGCGCGACTACTTAGAAAAATACTACAAGTTTGGAAGCCGCCATT
CAGCAGAATCACAAATTCTGAAACACCTTTTAAAGAACCTGTTTAAAATCTTCTGTTTAGATGGGGTCAAAGGAGATTTGTTGATTGACATCGG
ATCAGGTCCCACAATTTACCAACTTCTGTCCGCTTGTGAGAGTTTTAAGGAGATCGTCGTGACGGACTATTCAGATCAAAATCTGCAAGAACTG
GAGAAATGGTTAAAGAAAGAACCAGAGGCGTTCGATTGGAGCCCAGTTGTGACCTATGTCTGTGATCTTGAAGGAAACCGCGTAAAGGGTCCTG
AAAAGGAGGAAAAGCTGCGCCAAGCCGTAAAGCAAGTATTAAAATGTGATGTTACTCAAAGCCAACCATTAGGAGCCGTCCCACTGCCCCCGGC
GGATTGTGTATTGTCAACCCTTTGCTTGGACGCAGCTTGTCCTGACTTGCCTACATATTGTCGTGCATTACGCAACTTAGGAAGTTTGCTTAAA
CCAGGAGGGTTTCTGGTCATCATGGATGCCTTGAAGTCATCCTACTACATGATTGGGGAACAGAAGTTCTCATCTTTACCTTTGGGCCGTGAAG
CCGTAGAAGCGGCCGTAAAGGAGGCGGGGTACACCATTGAATGGTTCGAGGTTATTTCACAGTCCTACAGCTCCACAATGGCAAATAACGAGGG
CTTATTTTCGCTGGTTGCTCGCAAACTGAGTCGTCCTCTTCTCGAGCACCACCACCACCACCACTGA

MESGFTSKDTYLSHFNPRDYLEKYYKFGSRHSAESQILKHLLKNLFKIFCLDGVKGDLLIDIGSGPTIYQLLSACESFKEIVVTDYSDQNLQEL
EKWLKKEPEAFDWSPVVTYVCDLEGNRVKGPEKEEKLRQAVKQVLKCDVTQSQPLGAVPLPPADCVLSTLCLDAACPDLPTYCRALRNLGSLLK
PGGFLVIMDALKSSYYMIGEQKFSSLPLGREAVEAAVKEAGYTIEWFEVISQSYSSTMANNEGLFSLVARKLSRPLLEHHHHHH

>v1
ATGGAGAGTGGATTTACGAGTAAGGACACTTATTTGAGTCATTTTAACCCGCGCGACcACTTAGAAAAATACTACAAGTTTGGAAGCCGCCATT
CAGCAGAATCACAAATTCTGAAACACCTTTTAAAGAACCTGTTTAAAATCTTCTGTTTAGATGGGGTCAAAGGAGATTTGTTGATTGACATCGG
ATCAGGTCCCACAATTTACCAACTTCTGTCCGCTTGTGAGAGTTTTAAGGAGATCGTCGTGACGGACTATTCAGATCAAAATCTGCAAGAACTG
GAGAAATGGTTAAAGAAAGAACCAGAGGCGTTCGATTGGAGCCCAGTTGTGACCTATGTCTGTGATCTTGAAGGAAACCGCGTAAAGGGTCCTG
AAAAGGAGGAAAAGCTGCGCCAAGCCGTAAAGCAAGTATTAAAATGTGATGTTACTCAAAGCCAACCATTAGGAGCCGTCCCACTGCCCCCGGC
GGATTGTGTATTGTCAACCCTTTGCTTGtgCGCAGCTTGTCCTGACTTGCCTACATATTGTCGTGCATTACGCAACTTAGGAAGTTTGCTTAAA
CCAGGAGGGTTTCTGGTCATCATGGATatgTTGAAGgCgTCCTACTACATGATTGGGGAACAGAAGTTCTCAgCgTTACCTTTGGGCCGTGAAG
CCGTAGAAGCGGCCGTAAAGGAGGCGGGGTACACCATTGAATGGTTCGAGGTTATTTCACAGTCCTACAGCTCCACAATGGCAAATAACGAGGG
CTTATTTTCGCTGGTTGCTCGCAAACTGAGTCGTCCTCTTCTCGAGCACCACCACCACCACCACTGA

MESGFTSKDTYLSHFNPRDHLEKYYKFGSRHSAESQILKHLLKNLFKIFCLDGVKGDLLIDIGSGPTIYQLLSACESFKEIVVTDYSDQNLQEL
EKWLKKEPEAFDWSPVVTYVCDLEGNRVKGPEKEEKLRQAVKQVLKCDVTQSQPLGAVPLPPADCVLSTLCLCAACPDLPTYCRALRNLGSLLK
PGGFLVIMDMLKASYYMIGEQKFSALPLGREAVEAAVKEAGYTIEWFEVISQSYSSTMANNEGLFSLVARKLSRPLLEHHHHHH

>v2
ATGGAGAGTGGATTTACGAGTAAGGACACTTATTTGAGTCATTTTAACCCGCGCGACTACTTAGAAAAATACTACAAGTTTGGAAGCCGCCATT
CAGCAGAATCACAAATTCTGAAACACCTTTTAAAGAACCTGTTTAAAATCTTCTGTTTAGATGGGGTCAAAGGAGATTTGTTGATTGACATCGG
ATCAGGTCCCACAATTTACCAACTTCTGTCCGCTTGTGAGAGTTTTAAGGAGATCGTCGTGACGGACTATTCAGATCAAAATCTGCAAGAACTG
GAGAAATGGTTAAAGAAAGAACCAGAGGCGTTCGATTGGAGCCCAGTTGTGACCTATGTCTGTGATCTTGAAGGAAACCGCGTAAAGGGTCCTG
AAAAGGAGGAAAAGCTGCGCCAAGCCGTAAAGCAAGTATTAAAATGTGATGTTACTCAAAGCCAACCATTAGGAGCCGTCCCACTGCCCCCGGC
GGATTGTGTATTGTCAACCCTTTGCTTGcACGCAGCTTGTCCTGACTTGCCTACATATTGTCGTGCATTACGCAACTTAGGAAGTTTGCTTAAA
CCAGGAGGGTTTCTGGTCATCATGGATatgTTGAAGTgcTCCTACTACATGATTGGGGAACAGAAGTTCTCAgCgTTACCTTTGGGCCGTGAAG
CCGTAGAAGCGGCCGTAAAGGAGGCGGGGTACACCATTGAATGGTTCGAGGTTATTTCACAGTCCTACAGCTCCACAATGGCAAATtgCGAGGG
CTTATTTTCGCTGGTTGCTCGCAAACTGAGTCGTCCTCTTCTCGAGCACCACCACCACCACCACTGA

MESGFTSKDTYLSHFNPRDYLEKYYKFGSRHSAESQILKHLLKNLFKIFCLDGVKGDLLIDIGSGPTIYQLLSACESFKEIVVTDYSDQNLQEL
EKWLKKEPEAFDWSPVVTYVCDLEGNRVKGPEKEEKLRQAVKQVLKCDVTQSQPLGAVPLPPADCVLSTLCLHAACPDLPTYCRALRNLGSLLK
PGGFLVIMDMLKCSYYMIGEQKFSALPLGREAVEAAVKEAGYTIEWFEVISQSYSSTMANCEGLFSLVARKLSRPLLEHHHHHH

>v3
ATGGAGAGTGGATTTACGAGTAAGGACACTTATTTGAGTCATTTTAACCCGCGCGACTACTTAGAAAAATACTACAAGTTTGGAAGCCGCCATT
CAGCAGAATCACAAATTCTGAAACACCTTTTAAAGAACCTGTTTAAAATCTTCTGTTTAGATGGGGTCAAAGGAGATTTGTTGATTGACATCGG
ATCAGGTCCCACAATTTACCAACTTCTGTCCGCTTGTGAGAGTTTTAAGGAGATCGTCGTGACGGACTATTCAGATCAAAATCTGCAAGAACTG
GAGAAATGGTTAAAGAAAGAACCAGAGGCGTTCGATTGGAGCCCAGTTGTGACCTATGTCTGTGATCTTGAAGGAAACCGCGTAAAGGGTCCTG
AAAAGGAGGAAAAGCTGCGCCAAGCCGTAAAGCAAGTATTAAAATGTGATGTTACTCAAAGCCAACCATTAGGAGCCGTCCCACTGCCCCCGGC
GGATTGTGTATTGTCAACCCTTTGCTTGcACGCAGCTTGTCCTGACTTGCCTACATATTGTCGTGCATTACGCAACTTAGGAAGTTTGCTTAAA
CCAGGAGGGTTTCTGGTCATCATGGATtCCTTGAAGcgtTCCTACTACATGATTGGGGAACAGAAGTTCTCATCTTTACCTTTGGGCCGTGAAG
CCGTAGAAGCGGCCGTAAAGGAGGCGGGGTACACCATTGAATGGTTCGAGGTTATTTCACAGTCCTggAGCTCCACAATGGCAAATtcCGAGGG
CTTATTTTCGCTGGTTGCTCGCAAACTGAGTCGTCCTCTTCTCGAGCACCACCACCACCACCACTGA

MESGFTSKDTYLSHFNPRDYLEKYYKFGSRHSAESQILKHLLKNLFKIFCLDGVKGDLLIDIGSGPTIYQLLSACESFKEIVVTDYSDQNLQEL
EKWLKKEPEAFDWSPVVTYVCDLEGNRVKGPEKEEKLRQAVKQVLKCDVTQSQPLGAVPLPPADCVLSTLCLHAACPDLPTYCRALRNLGSLLK
PGGFLVIMDSLKRSYYMIGEQKFSSLPLGREAVEAAVKEAGYTIEWFEVISQSWSSTMANSEGLFSLVARKLSRPLLEHHHHHH

>v4
ATGGAGAGTGGATTTACGAGTAAGGACACTTATTTGAGTCATTTTAACCCGCGCGACTtCTTAGAAAAATACTACAAGTTTGGAAGCCGCCATT
CAGCAGAATCACAAATTCTGAAACACCTTTTAAAGAACCTGTTTAAAATCTTCTGTTTAGATGGGGTCAAAGGAGATTTGTTGATTGACATCGG
ATCAGGTCCCACAATTTACCAACTTCTGTCCGCTTGTGAGAGTTTTAAGGAGATCGTCGTGACGGACTATTCAGATCAAAATCTGCAAGAACTG
GAGAAATGGTTAAAGAAAGAACCAGAGGCGTTCGATTGGAGCCCAGTTGTGACCTATGTCTGTGATCTTGAAGGAAACCGCGTAAAGGGTCCTG
AAAAGGAGGAAAAGCTGCGCCAAGCCGTAAAGCAAGTATTAAAATGTGATGTTACTCAAAGCCAACCATTAGGAGCCGTCCCACTGCCCCCGGC
GGATTGTGTATTGTCAACCCTTTGCTTGcACGCAGCTTGTCCTGACTTGCCTACATATTGTCGTGCATTACGCAACTTAGGAAGTTTGCTTAAA
CCAGGAGGGTTTCTGGTCATCATGGATatgTTGAAGTgcTCCTACTACATGATTGGGGAACAGAAGTTCTCATgTTTACCTTTGGGCCGTGAAG
CCGTAGAAGCGGCCGTAAAGGAGGCGGGGTACACCATTGAATGGTTCGAGGTTATTTCACAGTCCTACAGCTCCACAATGGCAAATAACGAGGG
CTTATTTTCGCTGGTTGCTCGCAAACTGAGTCGTCCTCTTCTCGAGCACCACCACCACCACCACTGA

MESGFTSKDTYLSHFNPRDFLEKYYKFGSRHSAESQILKHLLKNLFKIFCLDGVKGDLLIDIGSGPTIYQLLSACESFKEIVVTDYSDQNLQEL
EKWLKKEPEAFDWSPVVTYVCDLEGNRVKGPEKEEKLRQAVKQVLKCDVTQSQPLGAVPLPPADCVLSTLCLHAACPDLPTYCRALRNLGSLLK
PGGFLVIMDMLKCSYYMIGEQKFSCLPLGREAVEAAVKEAGYTIEWFEVISQSYSSTMANNEGLFSLVARKLSRPLLEHHHHHH

>v5
ATGGAGAGTGGATTTACGAGTAAGGACACTTATTTGAGTCATTTTAACCCGCGCGACTACTTAGAAAAATACTACAAGTTTGGAAGCCGCCATT
CAGCAGAATCACAAATTCTGAAACACCTTTTAAAGAACCTGTTTAAAATCTTCTGTTTAGATGGGGTCAAAGGAGATTTGTTGATTGACATCGG
ATCAGGTCCCACAATTTACCAACTTCTGTCCGCTTGTGAGAGTTTTAAGGAGATCGTCGTGACGGACTATTCAGATCAAAATCTGCAAGAACTG
GAGAAATGGTTAAAGAAAGAACCAGAGGCGTTCGATTGGAGCCCAGTTGTGACCTATGTCTGTGATCTTGAAGGAAACCGCGTAAAGGGTCCTG
AAAAGGAGGAAAAGCTGCGCCAAGCCGTAAAGCAAGTATTAAAATGTGATGTTACTCAAAGCCAACCATTAGGAGCCGTCCCACTGCCCCCGGC
GGATTGTGTATTGTCAACCCTTTGCTTGcACGCAGCTTGTCCTGACTTGCCTACATATTGTCGTGCATTACGCAACTTAGGAAGTTTGCTTAAA
CCAGGAGGGTTTCTGGTCATCATGGATatgTTGAAGgCgTCCTACTACATGATTGGGGAACAGAAGTTCTCAgCgTTACCTTTGGGCCGTGAAG
CCGTAGAAGCGGCCGTAAAGGAGGCGGGGTACACCATTGAATGGTTCGAGGTTATTTCACAGTCCTtCAGCTCCACAATGGCAAATAACGAGGG
CTTATTTTCGCTGGTTGCTCGCAAACTGAGTCGTCCTCTTCTCGAGCACCACCACCACCACCACTGA

MESGFTSKDTYLSHFNPRDYLEKYYKFGSRHSAESQILKHLLKNLFKIFCLDGVKGDLLIDIGSGPTIYQLLSACESFKEIVVTDYSDQNLQEL
EKWLKKEPEAFDWSPVVTYVCDLEGNRVKGPEKEEKLRQAVKQVLKCDVTQSQPLGAVPLPPADCVLSTLCLHAACPDLPTYCRALRNLGSLLK
PGGFLVIMDMLKASYYMIGEQKFSALPLGREAVEAAVKEAGYTIEWFEVISQSFSSTMANNEGLFSLVARKLSRPLLEHHHHHH

>v6
ATGGAGAGTGGATTTACGAGTAAGGACACTTATTTGAGTCATTTTAACCCGCGCGACTACTTAGAAAAATACTACAAGTTTGGAAGCCGCCATT
CAGCAGAATCACAAATTCTGAAACACCTTTTAAAGAACCTGTTTAAAATCTTCTGTTTAGATGGGGTCAAAGGAGATTTGTTGATTGACATCGG
ATCAGGTCCCACAATTTACCAACTTCTGTCCGCTTGTGAGAGTTTTAAGGAGATCGTCGTGACGGACTATTCAGATCAAAATCTGCAAGAACTG
GAGAAATGGTTAAAGAAAGAACCAGAGGCGTTCGATTGGAGCCCAGTTGTGACCTATGTCTGTGATCTTGAAGGAAACCGCGTAAAGGGTCCTG
AAAAGGAGGAAAAGCTGCGCCAAGCCGTAAAGCAAGTATTAAAATGTGATGTTACTCAAAGCCAACCATTAGGAGCCGTCCCACTGCCCCCGGC
GGATTGTGTATTGTCAACCCTTTGCTTGcACGCAGCTTGTCCTGACTTGCCTACATATTGTCGTGCATTACGCAACTTAGGAAGTTTGCTTAAA
CCAGGAGGGTTTCTGGTCATCATGGATaCCTTGAAGTgcTCCTACTACATGATTGGGGAACAGAAGTTCTCAgCgTTACCTTTGGGCCGTGAAG
CCGTAGAAGCGGCCGTAAAGGAGGCGGGGTACACCATTGAATGGTTCGAGGTTATTTCACAGTCCTACAGCTCCACAATGatgAATAACGAGGG
CTTATTTTCGCTGGTTGCTCGCAAACTGAGTCGTCCTCTTCTCGAGCACCACCACCACCACCACTGA

MESGFTSKDTYLSHFNPRDYLEKYYKFGSRHSAESQILKHLLKNLFKIFCLDGVKGDLLIDIGSGPTIYQLLSACESFKEIVVTDYSDQNLQEL
EKWLKKEPEAFDWSPVVTYVCDLEGNRVKGPEKEEKLRQAVKQVLKCDVTQSQPLGAVPLPPADCVLSTLCLHAACPDLPTYCRALRNLGSLLK
PGGFLVIMDTLKCSYYMIGEQKFSALPLGREAVEAAVKEAGYTIEWFEVISQSYSSTMMNNEGLFSLVARKLSRPLLEHHHHHH

>v7
ATGGAGAGTGGATTTACGAGTAAGGACACTTATTTGAGTCATTTTAACCCGCGCGACTACTTAGAAAAATACTACAAGTTTGGAAGCCGCCATT
CAGCAGAATCACAAATTCTGAAACACCTTTTAAAGAACCTGTTTAAAATCTTCTGTTTAGATGGGGTCAAAGGAGATTTGTTGATTGACATCGG
ATCAGGTCCCACAATTTACCAACTTCTGTCCGCTTGTGAGAGTTTTAAGGAGATCGTCGTGACGGACTATTCAGATCAAAATCTGCAAGAACTG
GAGAAATGGTTAAAGAAAGAACCAGAGGCGTTCGATTGGAGCCCAGTTGTGACCTATGTCTGTGATCTTGAAGGAAACCGCGTAAAGGGTCCTG
AAAAGGAGGAAAAGCTGCGCCAAGCCGTAAAGCAAGTATTAAAATGTGATGTTACTCAAAGCCAACCATTAGGAGCCGTCCCACTGCCCCCGGC
GGATTGTGTATTGTCAACCCTTTGCTTGcACGCAGCTTGTCCTGACTTGCCTACATATTGTCGTGCATTACGCAACTTAGGAAGTTTGCTTAAA
CCAGGAGGGTTTCTGGTCATCATGGATGCCTTGAAGcagTCCTACTACATGATTGGGGAACAGAAGTTCTCAgCgTTACCTTTGGGCCGTGAAG
CCGTAGAAGCGGCCGTAAAGGAGGCGGGGTACACCATTGAATGGTTCGAGGTTATTTCACAGTCCTggAGCTCCACAATGGCAAATgcCGAGGG
CTTATTTTCGCTGGTTGCTCGCAAACTGAGTCGTCCTCTTCTCGAGCACCACCACCACCACCACTGA

MESGFTSKDTYLSHFNPRDYLEKYYKFGSRHSAESQILKHLLKNLFKIFCLDGVKGDLLIDIGSGPTIYQLLSACESFKEIVVTDYSDQNLQEL
EKWLKKEPEAFDWSPVVTYVCDLEGNRVKGPEKEEKLRQAVKQVLKCDVTQSQPLGAVPLPPADCVLSTLCLHAACPDLPTYCRALRNLGSLLK
PGGFLVIMDALKQSYYMIGEQKFSALPLGREAVEAAVKEAGYTIEWFEVISQSWSSTMANAEGLFSLVARKLSRPLLEHHHHHH

>v8
ATGGAGAGTGGATTTACGAGTAAGGACACTTATTTGAGTCATTTTAACCCGCGCGACTACTTAGAAAAATACTACAAGTTTGGAAGCCGCCATT
CAGCAGAATCACAAATTCTGAAACACCTTTTAAAGAACCTGTTTAAAATCTTCTGTTTAGATGGGGTCAAAGGAGATTTGTTGATTGACATCGG
ATCAGGTCCCACAATTTACCAACTTCTGTCCGCTTGTGAGAGTTTTAAGGAGATCGTCGTGACGGACTATTCAGATCAAAATCTGCAAGAACTG
GAGAAATGGTTAAAGAAAGAACCAGAGGCGTTCGATTGGAGCCCAGTTGTGACCTATGTCTGTGATCTTGAAGGAAACCGCGTAAAGGGTCCTG
AAAAGGAGGAAAAGCTGCGCCAAGCCGTAAAGCAAGTATTAAAATGTGATGTTACTCAAAGCCAACCATTAGGAGCCGTCCCACTGCCCCCGGC
GGATTGTGTATTGTCAACCCTTTGCTTGGACGCAGCTTGTCCTGACTTGCCTACATATTGTCGTGCATTACGCAACTTAGGAAGTTTGCTTAAA
CCAGGAGGGTTTCTGGTCATCATGGgTatgTTGAAGTgcTCCTACTACATGATTGGGGAACAGAAGTTCTCAgCgTTACCTTTGGGCCGTGAAG
CCGTAGAAGCGGCCGTAAAGGAGGCGGGGTACACCATTGAATGGTTCGAGGTTATTTCACAGTCCTACAGCTCCACAATGGCAAATAACGAGGG
CTTATTTTCGCTGGTTGCTCGCAAACTGAGTCGTCCTCTTCTCGAGCACCACCACCACCACCACTGA

MESGFTSKDTYLSHFNPRDYLEKYYKFGSRHSAESQILKHLLKNLFKIFCLDGVKGDLLIDIGSGPTIYQLLSACESFKEIVVTDYSDQNLQEL
EKWLKKEPEAFDWSPVVTYVCDLEGNRVKGPEKEEKLRQAVKQVLKCDVTQSQPLGAVPLPPADCVLSTLCLDAACPDLPTYCRALRNLGSLLK
PGGFLVIMGMLKCSYYMIGEQKFSALPLGREAVEAAVKEAGYTIEWFEVISQSYSSTMANNEGLFSLVARKLSRPLLEHHHHHH

>v9
ATGGAGAGTGGATTTACGAGTAAGGACACTTATTTGAGTCATTTTAACCCGCGCGACTACTTAGAAAAATACTACAAGTTTGGAAGCCGCCATT
CAGCAGAATCACAAATTCTGAAACACCTTTTAAAGAACCTGTTTAAAATCTTCTGTTTAGATGGGGTCAAAGGAGATTTGTTGATTGACATCGG
ATCAGGTCCCACAATTTACCAACTTCTGTCCGCTTGTGAGAGTTTTAAGGAGATCGTCGTGACGGACTATTCAGATCAAAATCTGCAAGAACTG
GAGAAATGGTTAAAGAAAGAACCAGAGGCGTTCGATTGGAGCCCAGTTGTGACCTATGTCTGTGATCTTGAAGGAAACCGCGTAAAGGGTCCTG
AAAAGGAGGAAAAGCTGCGCCAAGCCGTAAAGCAAGTATTAAAATGTGATGTTACTCAAAGCCAACCATTAGGAGCCGTCCCACTGCCCCCGGC
GGATTGTGTATTGTCAACCCTTTGCTTGtgCGCAGCTTGTCCTGACTTGCCTACATATTGTCGTGCATTACGCAACTTAGGAAGTTTGCTTAAA
CCAGGAGGGTTTCTGGTCATCATGGATaCCTTGAAGaacTCCTACTACATGATTGGGGAACAGAAGTTCTCAgCgTTACCTTTGGGCCGTGAAG
CCGTAGAAGCGGCCGTAAAGGAGGCGGGGTACACCATTGAATGGTTCGAGGTTATTTCACAGTCCTtCAGCTCCACAATGGCAAATAACGAGGG
CTTATTTTCGCTGGTTGCTCGCAAACTGAGTCGTCCTCTTCTCGAGCACCACCACCACCACCACTGA

MESGFTSKDTYLSHFNPRDYLEKYYKFGSRHSAESQILKHLLKNLFKIFCLDGVKGDLLIDIGSGPTIYQLLSACESFKEIVVTDYSDQNLQEL
EKWLKKEPEAFDWSPVVTYVCDLEGNRVKGPEKEEKLRQAVKQVLKCDVTQSQPLGAVPLPPADCVLSTLCLCAACPDLPTYCRALRNLGSLLK
PGGFLVIMDTLKNSYYMIGEQKFSALPLGREAVEAAVKEAGYTIEWFEVISQSFSSTMANNEGLFSLVARKLSRPLLEHHHHHH

>v10
ATGGAGAGTGGATTTACGAGTAAGGACACTTATTTGAGTCATTTTAACCCGCGCGACTACTTAGAAAAATACTACAAGTTTGGAAGCCGCCATT
CAGCAGAATCACAAATTCTGAAACACCTTTTAAAGAACCTGTTTAAAATCTTCTGTTTAGATGGGGTCAAAGGAGATTTGTTGATTGACATCGG
ATCAGGTCCCACAATTTACCAACTTCTGTCCGCTTGTGAGAGTTTTAAGGAGATCGTCGTGACGGACTATTCAGATCAAAATCTGCAAGAACTG
GAGAAATGGTTAAAGAAAGAACCAGAGGCGTTCGATTGGAGCCCAGTTGTGACCTATGTCTGTGATCTTGAAGGAAACCGCGTAAAGGGTCCTG
AAAAGGAGGAAAAGCTGCGCCAAGCCGTAAAGCAAGTATTAAAATGTGATGTTACTCAAAGCCAACCATTAGGAGCCGTCCCACTGCCCCCGGC
GGATTGTGTATTGTCAACCCTTTGCTTGcACGCAGCTTGTCCTGACTTGCCTACATATTGTCGTGCATTACGCAACTTAGGAAGTTTGCTTAAA
CCAGGAGGGTTTCTGGTCATCATGGATatgTTGAAGgCgTCCTACTACATGATTGGGGAACAGAAGTTCTCAcaTTTACCTTTGGGCCGTGAAG
CCGTAGAAGCGGCCGTAAAGGAGGCGGGGTACACCATTGAATGGTTCGAGGTTATTTCACAGTCCTACAGCTCCACAATGGCAAATgcCGAGGG
CTTATTTTCGCTGGTTGCTCGCAAACTGAGTCGTCCTCTTCTCGAGCACCACCACCACCACCACTGA

MESGFTSKDTYLSHFNPRDYLEKYYKFGSRHSAESQILKHLLKNLFKIFCLDGVKGDLLIDIGSGPTIYQLLSACESFKEIVVTDYSDQNLQEL
EKWLKKEPEAFDWSPVVTYVCDLEGNRVKGPEKEEKLRQAVKQVLKCDVTQSQPLGAVPLPPADCVLSTLCLHAACPDLPTYCRALRNLGSLLK
PGGFLVIMDMLKASYYMIGEQKFSHLPLGREAVEAAVKEAGYTIEWFEVISQSYSSTMANAEGLFSLVARKLSRPLLEHHHHHH

>v11
ATGGAGAGTGGATTTACGAGTAAGGACACTTATTTGAGTCATTTTAACCCGCGCGACTACTTAGAAAAATACTACAAGTTTGGAAGCCGCCATT
CAGCAGAATCACAAATTCTGAAACACCTTTTAAAGAACCTGTTTAAAATCTTCTGTTTAGATGGGGTCAAAGGAGATTTGTTGATTGACATCGG
ATCAGGTCCCACAATTTACCAACTTCTGTCCGCTTGTGAGAGTTTTAAGGAGATCGTCGTGACGGACTATTCAGATCAAAATCTGCAAGAACTG
GAGAAATGGTTAAAGAAAGAACCAGAGGCGTTCGATTGGAGCCCAGTTGTGACCTATGTCTGTGATCTTGAAGGAAACCGCGTAAAGGGTCCTG
AAAAGGAGGAAAAGCTGCGCCAAGCCGTAAAGCAAGTATTAAAATGTGATGTTACTCAAAGCCAACCATTAGGAGCCGTCCCACTGCCCCCGGC
GGATTGTGTATTGTCAACCCTTTGCTTGtgCGCAGCTTGTCCTGACTTGCCTACATATTGTCGTGCATTACGCAACTTAGGAAGTTTGCTTAAA
CCAGGAGGGTTTCTGGTCATCATGGATctgTTGAAGTgcTCCTACTACATGATTGGGGAACAGAAGTTCTCAgCgTTACCTTTGGGCCGTGAAG
CCGTAGAAGCGGCCGTAAAGGAGGCGGGGTACACCATTGAATGGTTCGAGGTTATTTCACAGTCCTACAGCTCCACAATGGCAAATgcCGAGGG
CTTATTTTCGCTGGTTGCTCGCAAACTGAGTCGTCCTCTTCTCGAGCACCACCACCACCACCACTGA

MESGFTSKDTYLSHFNPRDYLEKYYKFGSRHSAESQILKHLLKNLFKIFCLDGVKGDLLIDIGSGPTIYQLLSACESFKEIVVTDYSDQNLQEL
EKWLKKEPEAFDWSPVVTYVCDLEGNRVKGPEKEEKLRQAVKQVLKCDVTQSQPLGAVPLPPADCVLSTLCLCAACPDLPTYCRALRNLGSLLK
PGGFLVIMDLLKCSYYMIGEQKFSALPLGREAVEAAVKEAGYTIEWFEVISQSYSSTMANAEGLFSLVARKLSRPLLEHHHHHH

>Vv2
ATGGAGAGTGGATTTACGAGTAAGGACACTTATTTGAGTCATTTTAACCCGCGCGACTtCTTAGAAAAATACTACAAGTTTGGAAGCCGCCATT
CAGCAGAATCACAAATTCTGAAACACCTTTTAAAGAACCTGTTTAAAATCTTCTGTTTAGATGGGGTCAAAGGAGATTTGTTGATTGACATCGG
ATCAGGTCCCACAATTTACCAACTTCTGTCCGCTTGTGAGAGTTTTAAGGAGATCGTCGTGACGGACTATTCAGATCAAAATCTGCAAGAACTG
GAGAAATGGTTAAAGAAAGAACCAGAGGCGTTCGATTGGAGCCCAGTTGTGACCTATGTCTGTGATCTTGAAGGAAACCGCGTAAAGGGTCCTG
AAAAGGAGGAAAAGCTGCGCCAAGCCGTAAAGCAAGTATTAAAATGTGATGTTACTCAAAGCCAACCATTAGGAGCCGTCCCACTGCCCCCGGC
GGATTGTGTATTGTCAACCCTTTGCTTGGACGCAGCTTGTCCTGACTTGCCTACATATTGTCGTGCATTACGCAACTTAGGAAGTTTGCTTAAA
CCAGGAGGGTTTCTGGTCATCATGGgTaCCTTGAAGcgtTCCTACTACATGATTGGGGAACAGAAGTTCTCATCTTTACCTTTGGGCCGTGAAG
CCGTAGAAGCGGCCGTAAAGGAGGCGGGGTACACCATTGAATGGTTCGAGGTTATTTCACAGTCCTACAGCTCCACAATGGCAAATAACGAGGG
CTTATTTTCGCTGGTTGCTCGCAAACTGAGTCGTCCTCTTCTCGAGCACCACCACCACCACCACTGA

MESGFTSKDTYLSHFNPRDFLEKYYKFGSRHSAESQILKHLLKNLFKIFCLDGVKGDLLIDIGSGPTIYQLLSACESFKEIVVTDYSDQNLQEL
EKWLKKEPEAFDWSPVVTYVCDLEGNRVKGPEKEEKLRQAVKQVLKCDVTQSQPLGAVPLPPADCVLSTLCLDAACPDLPTYCRALRNLGSLLK
PGGFLVIMGTLKRSYYMIGEQKFSSLPLGREAVEAAVKEAGYTIEWFEVISQSYSSTMANNEGLFSLVARKLSRPLLEHHHHHH

>v13
ATGGAGAGTGGATTTACGAGTAAGGACACTTATTTGAGTCATTTTAACCCGCGCGACTtCTTAGAAAAATACTACAAGTTTGGAAGCCGCCATT
CAGCAGAATCACAAATTCTGAAACACCTTTTAAAGAACCTGTTTAAAATCTTCTGTTTAGATGGGGTCAAAGGAGATTTGTTGATTGACATCGG
ATCAGGTCCCACAATTTACCAACTTCTGTCCGCTTGTGAGAGTTTTAAGGAGATCGTCGTGACGGACTATTCAGATCAAAATCTGCAAGAACTG
GAGAAATGGTTAAAGAAAGAACCAGAGGCGTTCGATTGGAGCCCAGTTGTGACCTATGTCTGTGATCTTGAAGGAAACCGCGTAAAGGGTCCTG
AAAAGGAGGAAAAGCTGCGCCAAGCCGTAAAGCAAGTATTAAAATGTGATGTTACTCAAAGCCAACCATTAGGAGCCGTCCCACTGCCCCCGGC
GGATTGTGTATTGTCAACCCTTTGCTTGcACGCAGCTTGTCCTGACTTGCCTACATATTGTCGTGCATTACGCAACTTAGGAAGTTTGCTTAAA
CCAGGAGGGTTTCTGGTCATCATGGATGCCTTGAAGcagTCCTACTACATGATTGGGGAACAGAAGTTCTCATCTTTACCTTTGGGCCGTGAAG
CCGTAGAAGCGGCCGTAAAGGAGGCGGGGTACACCATTGAATGGTTCGAGGTTATTTCACAGTCCTACAGCTCCACAATGGCAAATtcCGAGGG
CTTATTTTCGCTGGTTGCTCGCAAACTGAGTCGTCCTCTTCTCGAGCACCACCACCACCACCACTGA

MESGFTSKDTYLSHFNPRDFLEKYYKFGSRHSAESQILKHLLKNLFKIFCLDGVKGDLLIDIGSGPTIYQLLSACESFKEIVVTDYSDQNLQEL
EKWLKKEPEAFDWSPVVTYVCDLEGNRVKGPEKEEKLRQAVKQVLKCDVTQSQPLGAVPLPPADCVLSTLCLHAACPDLPTYCRALRNLGSLLK
PGGFLVIMDALKQSYYMIGEQKFSSLPLGREAVEAAVKEAGYTIEWFEVISQSYSSTMANSEGLFSLVARKLSRPLLEHHHHHH

>v14
ATGGAGAGTGGATTTACGAGTAAGGACACTTATTTGAGTCATTTTAACCCGCGCGACTtCTTAGAAAAATACTACAAGTTTGGAAGCCGCCATT
CAGCAGAATCACAAATTCTGAAACACCTTTTAAAGAACCTGTTTAAAATCTTCTGTTTAGATGGGGTCAAAGGAGATTTGTTGATTGACATCGG
ATCAGGTCCCACAATTTACCAACTTCTGTCCGCTTGTGAGAGTTTTAAGGAGATCGTCGTGACGGACTATTCAGATCAAAATCTGCAAGAACTG
GAGAAATGGTTAAAGAAAGAACCAGAGGCGTTCGATTGGAGCCCAGTTGTGACCTATGTCTGTGATCTTGAAGGAAACCGCGTAAAGGGTCCTG
AAAAGGAGGAAAAGCTGCGCCAAGCCGTAAAGCAAGTATTAAAATGTGATGTTACTCAAAGCCAACCATTAGGAGCCGTCCCACTGCCCCCGGC
GGATTGTGTATTGTCAACCCTTTGCTTGcACGCAGCTTGTCCTGACTTGCCTACATATTGTCGTGCATTACGCAACTTAGGAAGTTTGCTTAAA
CCAGGAGGGTTTCTGGTCATCATGGATctgTTGAAGgCgTCCTACTACATGATTGGGGAACAGAAGTTCTCAgCgTTACCTTTGGGCCGTGAAG
CCGTAGAAGCGGCCGTAAAGGAGGCGGGGTACACCATTGAATGGTTCGAGGTTATTTCACAGTCCTACAGCTCCACAATGGCAAATAACGAGGG
CTTATTTTCGCTGGTTGCTCGCAAACTGAGTCGTCCTCTTCTCGAGCACCACCACCACCACCACTGA

MESGFTSKDTYLSHFNPRDFLEKYYKFGSRHSAESQILKHLLKNLFKIFCLDGVKGDLLIDIGSGPTIYQLLSACESFKEIVVTDYSDQNLQEL
EKWLKKEPEAFDWSPVVTYVCDLEGNRVKGPEKEEKLRQAVKQVLKCDVTQSQPLGAVPLPPADCVLSTLCLHAACPDLPTYCRALRNLGSLLK
PGGFLVIMDLLKASYYMIGEQKFSALPLGREAVEAAVKEAGYTIEWFEVISQSYSSTMANNEGLFSLVARKLSRPLLEHHHHHH

>v15
ATGGAGAGTGGATTTACGAGTAAGGACACTTATTTGAGTCATTTTAACCCGCGCGACTACTTAGAAAAATACTACAAGTTTGGAAGCCGCCATT
CAGCAGAATCACAAATTCTGAAACACCTTTTAAAGAACCTGTTTAAAATCTTCTGTTTAGATGGGGTCAAAGGAGATTTGTTGATTGACATCGG
ATCAGGTCCCACAATTTACCAACTTCTGTCCGCTTGTGAGAGTTTTAAGGAGATCGTCGTGACGGACTATTCAGATCAAAATCTGCAAGAACTG
GAGAAATGGTTAAAGAAAGAACCAGAGGCGTTCGATTGGAGCCCAGTTGTGACCTATGTCTGTGATCTTGAAGGAAACCGCGTAAAGGGTCCTG
AAAAGGAGGAAAAGCTGCGCCAAGCCGTAAAGCAAGTATTAAAATGTGATGTTACTCAAAGCCAACCATTAGGAGCCGTCCCACTGCCCCCGGC
GGATTGTGTATTGTCAACCCTTTGCTTGcACGCAGCTTGTCCTGACTTGCCTACATATTGTCGTGCATTACGCAACTTAGGAAGTTTGCTTAAA
CCAGGAGGGTTTCTGGTCATCATGGATGCCTTGAAGaCcTCCTACTACATGATTGGGGAACAGAAGTTCTCAcaTTTACCTTTGGGCCGTGAAG
CCGTAGAAGCGGCCGTAAAGGAGGCGGGGTACACCATTGAATGGTTCGAGGTTATTTCACAGTCCTtCAGCTCCACAATGGCAAATtcCGAGGG
CTTATTTTCGCTGGTTGCTCGCAAACTGAGTCGTCCTCTTCTCGAGCACCACCACCACCACCACTGA

MESGFTSKDTYLSHFNPRDYLEKYYKFGSRHSAESQILKHLLKNLFKIFCLDGVKGDLLIDIGSGPTIYQLLSACESFKEIVVTDYSDQNLQEL
EKWLKKEPEAFDWSPVVTYVCDLEGNRVKGPEKEEKLRQAVKQVLKCDVTQSQPLGAVPLPPADCVLSTLCLHAACPDLPTYCRALRNLGSLLK
PGGFLVIMDALKTSYYMIGEQKFSHLPLGREAVEAAVKEAGYTIEWFEVISQSFSSTMANSEGLFSLVARKLSRPLLEHHHHHH

>v16
ATGGAGAGTGGATTTACGAGTAAGGACACTTATTTGAGTCATTTTAACCCGCGCGACTACTTAGAAAAATACTACAAGTTTGGAAGCCGCCATT
CAGCAGAATCACAAATTCTGAAACACCTTTTAAAGAACCTGTTTAAAATCTTCTGTTTAGATGGGGTCAAAGGAGATTTGTTGATTGACATCGG
ATCAGGTCCCACAATTTACCAACTTCTGTCCGCTTGTGAGAGTTTTAAGGAGATCGTCGTGACGGACTATTCAGATCAAAATCTGCAAGAACTG
GAGAAATGGTTAAAGAAAGAACCAGAGGCGTTCGATTGGAGCCCAGTTGTGACCTATGTCTGTGATCTTGAAGGAAACCGCGTAAAGGGTCCTG
AAAAGGAGGAAAAGCTGCGCCAAGCCGTAAAGCAAGTATTAAAATGTGATGTTACTCAAAGCCAACCATTAGGAGCCGTCCCACTGCCCCCGGC
GGATTGTGTATTGTCAACCCTTTGCTTGcACGCAGCTTGTCCTGACTTGCCTACATATTGTCGTGCATTACGCAACTTAGGAAGTTTGCTTAAA
CCAGGAGGGTTTCTGGTCATCATGGATGCCTTGAAGaacTCCTACTACATGATTGGGGAACAGAAGTTCTCAgCgTTACCTTTGGGCCGTGAAG
CCGTAGAAGCGGCCGTAAAGGAGGCGGGGTACACCATTGAATGGTTCGAGGTTATTTCACAGTCCTACAGCTCCACAATGatgAATgcCGAGGG
CTTATTTTCGCTGGTTGCTCGCAAACTGAGTCGTCCTCTTCTCGAGCACCACCACCACCACCACTGA

MESGFTSKDTYLSHFNPRDYLEKYYKFGSRHSAESQILKHLLKNLFKIFCLDGVKGDLLIDIGSGPTIYQLLSACESFKEIVVTDYSDQNLQEL
EKWLKKEPEAFDWSPVVTYVCDLEGNRVKGPEKEEKLRQAVKQVLKCDVTQSQPLGAVPLPPADCVLSTLCLHAACPDLPTYCRALRNLGSLLK
PGGFLVIMDALKNSYYMIGEQKFSALPLGREAVEAAVKEAGYTIEWFEVISQSYSSTMMNAEGLFSLVARKLSRPLLEHHHHHH

>v17
ATGGAGAGTGGATTTACGAGTAAGGACACTTATTTGAGTCATTTTAACCCGCGCGACTACTTAGAAAAATACTACAAGTTTGGAAGCCGCCATT
CAGCAGAATCACAAATTCTGAAACACCTTTTAAAGAACCTGTTTAAAATCTTCTGTTTAGATGGGGTCAAAGGAGATTTGTTGATTGACATCGG
ATCAGGTCCCACAATTTACCAACTTCTGTCCGCTTGTGAGAGTTTTAAGGAGATCGTCGTGACGGACTATTCAGATCAAAATCTGCAAGAACTG
GAGAAATGGTTAAAGAAAGAACCAGAGGCGTTCGATTGGAGCCCAGTTGTGACCTATGTCTGTGATCTTGAAGGAAACCGCGTAAAGGGTCCTG
AAAAGGAGGAAAAGCTGCGCCAAGCCGTAAAGCAAGTATTAAAATGTGATGTTACTCAAAGCCAACCATTAGGAGCCGTCCCACTGCCCCCGGC
GGATTGTGTATTGTCAACCCTTTGCTTGtgCGCAGCTTGTCCTGACTTGCCTACATATTGTCGTGCATTACGCAACTTAGGAAGTTTGCTTAAA
CCAGGAGGGTTTCTGGTCATCATGGATaCCTTGAAGcagTCCTACTACATGATTGGGGAACAGAAGTTCTCAgCgTTACCTTTGGGCCGTGAAG
CCGTAGAAGCGGCCGTAAAGGAGGCGGGGTACACCATTGAATGGTTCGAGGTTATTTCACAGTCCTACAGCTCCACAATGagcAATAACGAGGG
CTTATTTTCGCTGGTTGCTCGCAAACTGAGTCGTCCTCTTCTCGAGCACCACCACCACCACCACTGA

MESGFTSKDTYLSHFNPRDYLEKYYKFGSRHSAESQILKHLLKNLFKIFCLDGVKGDLLIDIGSGPTIYQLLSACESFKEIVVTDYSDQNLQEL
EKWLKKEPEAFDWSPVVTYVCDLEGNRVKGPEKEEKLRQAVKQVLKCDVTQSQPLGAVPLPPADCVLSTLCLCAACPDLPTYCRALRNLGSLLK
PGGFLVIMDTLKQSYYMIGEQKFSALPLGREAVEAAVKEAGYTIEWFEVISQSYSSTMSNNEGLFSLVARKLSRPLLEHHHHHH

>v18
ATGGAGAGTGGATTTACGAGTAAGGACACTTATTTGAGTCATTTTAACCCGCGCGACTACTTAGAAAAATtCTACAAGTTTGGAAGCCGCCATT
CAGCAGAATCACAAATTCTGAAACACCTTTTAAAGAACCTGTTTAAAATCTTCTGTTTAGATGGGGTCAAAGGAGATTTGTTGATTGACATCGG
ATCAGGTCCCACAATTTACCAACTTCTGTCCGCTTGTGAGAGTTTTAAGGAGATCGTCGTGACGGACTATTCAGATCAAAATCTGCAAGAACTG
GAGAAATGGTTAAAGAAAGAACCAGAGGCGTTCGATTGGAGCCCAGTTGTGACCTATGTCTGTGATCTTGAAGGAAACCGCGTAAAGGGTCCTG
AAAAGGAGGAAAAGCTGCGCCAAGCCGTAAAGCAAGTATTAAAATGTGATGTTACTCAAAGCCAACCATTAGGAGCCGTCCCACTGCCCCCGGC
GGATTGTGTATTGTCAACCCTTTGCTTGtgCGCAGCTTGTCCTGACTTGCCTACATATTGTCGTGCATTACGCAACTTAGGAAGTTTGCTTAAA
CCAGGAGGGTTTCTGGTCATCATGGATGCCTTGAAGcgtTCCTACTACATGATTGGGGAACAGAAGTTCTCATCTTTACCTTTGGGCCGTGAAG
CCGTAGAAGCGGCCGTAAAGGAGGCGGGGTACACCATTGAATGGTTCGAGGTTATTTCACAGTCCTggAGCTCCACAATGGCAAATgcCGAGGG
CTTATTTTCGCTGGTTGCTCGCAAACTGAGTCGTCCTCTTCTCGAGCACCACCACCACCACCACTGA

MESGFTSKDTYLSHFNPRDYLEKFYKFGSRHSAESQILKHLLKNLFKIFCLDGVKGDLLIDIGSGPTIYQLLSACESFKEIVVTDYSDQNLQEL
EKWLKKEPEAFDWSPVVTYVCDLEGNRVKGPEKEEKLRQAVKQVLKCDVTQSQPLGAVPLPPADCVLSTLCLCAACPDLPTYCRALRNLGSLLK
PGGFLVIMDALKRSYYMIGEQKFSSLPLGREAVEAAVKEAGYTIEWFEVISQSWSSTMANAEGLFSLVARKLSRPLLEHHHHHH

>v19
ATGGAGAGTGGATTTACGAGTAAGGACACTTATTTGAGTCATTTTAACCCGCGCGACcACTTAGAAAAATACTACAAGTTTGGAAGCCGCCATT
CAGCAGAATCACAAATTCTGAAACACCTTTTAAAGAACCTGTTTAAAATCTTCTGTTTAGATGGGGTCAAAGGAGATTTGTTGATTGACATCGG
ATCAGGTCCCACAATTTACCAACTTCTGTCCGCTTGTGAGAGTTTTAAGGAGATCGTCGTGACGGACTATTCAGATCAAAATCTGCAAGAACTG
GAGAAATGGTTAAAGAAAGAACCAGAGGCGTTCGATTGGAGCCCAGTTGTGACCTATGTCTGTGATCTTGAAGGAAACCGCGTAAAGGGTCCTG
AAAAGGAGGAAAAGCTGCGCCAAGCCGTAAAGCAAGTATTAAAATGTGATGTTACTCAAAGCCAACCATTAGGAGCCGTCCCACTGCCCCCGGC
GGATTGTGTATTGTCAACCCTTTGCTTGcACGCAGCTTGTCCTGACTTGCCTACATATTGTCGTGCATTACGCAACTTAGGAAGTTTGCTTAAA
CCAGGAGGGTTTCTGGTCATCATGGATGCCTTGAAGcagTCCTACTACATGATTGGGGAACAGAAGTTCTCATCTTTACCTTTGGGCCGTGAAG
CCGTAGAAGCGGCCGTAAAGGAGGCGGGGTACACCATTGAATGGTTCGAGGTTATTTCACAGTCCTggAGCTCCACAATGGCAAATtgCGAGGG
CTTATTTTCGCTGGTTGCTCGCAAACTGAGTCGTCCTCTTCTCGAGCACCACCACCACCACCACTGA

MESGFTSKDTYLSHFNPRDHLEKYYKFGSRHSAESQILKHLLKNLFKIFCLDGVKGDLLIDIGSGPTIYQLLSACESFKEIVVTDYSDQNLQEL
EKWLKKEPEAFDWSPVVTYVCDLEGNRVKGPEKEEKLRQAVKQVLKCDVTQSQPLGAVPLPPADCVLSTLCLHAACPDLPTYCRALRNLGSLLK
PGGFLVIMDALKQSYYMIGEQKFSSLPLGREAVEAAVKEAGYTIEWFEVISQSWSSTMANCEGLFSLVARKLSRPLLEHHHHHH

>v20
ATGGAGAGTGGATTTACGAGTAAGGACACTTATTTGAGTCATTTTAACCCGCGCGACTACTTAGAAAAATACTACAAGTTTGGAAGCCGCCATT
CAGCAGAATCACAAATTCTGAAACACCTTTTAAAGAACCTGTTTAAAATCTTCTGTTTAGATGGGGTCAAAGGAGATTTGTTGATTGACATCGG
ATCAGGTCCCACAATTTACCAACTTCTGTCCGCTTGTGAGAGTTTTAAGGAGATCGTCGTGACGGACTATTCAGATCAAAATCTGCAAGAACTG
GAGAAATGGTTAAAGAAAGAACCAGAGGCGTTCGATTGGAGCCCAGTTGTGACCTATGTCTGTGATCTTGAAGGAAACCGCGTAAAGGGTCCTG
AAAAGGAGGAAAAGCTGCGCCAAGCCGTAAAGCAAGTATTAAAATGTGATGTTACTCAAAGCCAACCATTAGGAGCCGTCCCACTGCCCCCGGC
GGATTGTGTATTGTCAACCCTTTGCTTGGACGCAGCTTGTCCTGACTTGCCTACATATTGTCGTGCATTACGCAACTTAGGAAGTTTGCTTAAA
CCAGGAGGGTTTCTGGTCATCATGGgTatgTTGAAGgCgTCCTACTACATGATTGGGGAACAGAAGTTCTCATCTTTACCTTTGGGCCGTGAAG
CCGTAGAAGCGGCCGTAAAGGAGGCGGGGTACACCATTGAATGGTTCGAGGTTATTTCACAGTCCTtCAGCTCCACAATGGCAAATgcCGAGGG
CTTATTTTCGCTGGTTGCTCGCAAACTGAGTCGTCCTCTTCTCGAGCACCACCACCACCACCACTGA

MESGFTSKDTYLSHFNPRDYLEKYYKFGSRHSAESQILKHLLKNLFKIFCLDGVKGDLLIDIGSGPTIYQLLSACESFKEIVVTDYSDQNLQEL
EKWLKKEPEAFDWSPVVTYVCDLEGNRVKGPEKEEKLRQAVKQVLKCDVTQSQPLGAVPLPPADCVLSTLCLDAACPDLPTYCRALRNLGSLLK
PGGFLVIMGMLKASYYMIGEQKFSSLPLGREAVEAAVKEAGYTIEWFEVISQSFSSTMANAEGLFSLVARKLSRPLLEHHHHHH

>v21
ATGGAGAGTGGATTTACGAGTAAGGACACTTATTTGAGTCATTTTAACCCGCGCGACTACTTAGAAAAATtCTACAAGTTTGGAAGCCGCCATT
CAGCAGAATCACAAATTCTGAAACACCTTTTAAAGAACCTGTTTAAAATCTTCTGTTTAGATGGGGTCAAAGGAGATTTGTTGATTGACATCGG
ATCAGGTCCCACAATTTACCAACTTCTGTCCGCTTGTGAGAGTTTTAAGGAGATCGTCGTGACGGACTATTCAGATCAAAATCTGCAAGAACTG
GAGAAATGGTTAAAGAAAGAACCAGAGGCGTTCGATTGGAGCCCAGTTGTGACCTATGTCTGTGATCTTGAAGGAAACCGCGTAAAGGGTCCTG
AAAAGGAGGAAAAGCTGCGCCAAGCCGTAAAGCAAGTATTAAAATGTGATGTTACTCAAAGCCAACCATTAGGAGCCGTCCCACTGCCCCCGGC
GGATTGTGTATTGTCAACCCTTTGCTTGcACGCAGCTTGTCCTGACTTGCCTACATATTGTCGTGCATTACGCAACTTAGGAAGTTTGCTTAAA
CCAGGAGGGTTTCTGGTCATCATGGATGCCTTGAAGgaATCCTACTACATGATTGGGGAACAGAAGTTCTCAgCgTTACCTTTGGGCCGTGAAG
CCGTAGAAGCGGCCGTAAAGGAGGCGGGGTACACCATTGAATGGTTCGAGGTTATTTCACAGTCCTACAGCTCCACAATGGCAAATgcCGAGGG
CTTATTTTCGCTGGTTGCTCGCAAACTGAGTCGTCCTCTTCTCGAGCACCACCACCACCACCACTGA

MESGFTSKDTYLSHFNPRDYLEKFYKFGSRHSAESQILKHLLKNLFKIFCLDGVKGDLLIDIGSGPTIYQLLSACESFKEIVVTDYSDQNLQEL
EKWLKKEPEAFDWSPVVTYVCDLEGNRVKGPEKEEKLRQAVKQVLKCDVTQSQPLGAVPLPPADCVLSTLCLHAACPDLPTYCRALRNLGSLLK
PGGFLVIMDALKESYYMIGEQKFSALPLGREAVEAAVKEAGYTIEWFEVISQSYSSTMANAEGLFSLVARKLSRPLLEHHHHHH

>v22
ATGGAGAGTGGATTTACGAGTAAGGACACTTATTTGAGTCATTTTAACCCGCGCGACTACTTAGAAAAATACTACAAGTTTGGAAGCCGCCATT
CAGCAGAATCACAAATTCTGAAACACCTTTTAAAGAACCTGTTTAAAATCTTCTGTTTAGATGGGGTCAAAGGAGATTTGTTGATTGACATCGG
ATCAGGTCCCACAATTTACCAACTTCTGTCCGCTTGTGAGAGTTTTAAGGAGATCGTCGTGACGGACTATTCAGATCAAAATCTGCAAGAACTG
GAGAAATGGTTAAAGAAAGAACCAGAGGCGTTCGATTGGAGCCCAGTTGTGACCTATGTCTGTGATCTTGAAGGAAACCGCGTAAAGGGTCCTG
AAAAGGAGGAAAAGCTGCGCCAAGCCGTAAAGCAAGTATTAAAATGTGATGTTACTCAAAGCCAACCATTAGGAGCCGTCCCACTGCCCCCGGC
GGATTGTGTATTGTCAACCCTTTGCTTGcACGCAGCTTGTCCTGACTTGCCTACATATTGTCGTGCATTACGCAACTTAGGAAGTTTGCTTAAA
CCAGGAGGGTTTCTGGTCATCATGGATGCCTTGAAGgCgTCCTACTACATGATTGGGGAACAGAAGTTCTCAcaTTTACCTTTGGGCCGTGAAG
CCGTAGAAGCGGCCGTAAAGGAGGCGGGGTACACCATTGAATGGTTCGAGGTTATTTCACAGTCCTACAGCTCCACAATGatgAATtgCGAGGG
CTTATTTTCGCTGGTTGCTCGCAAACTGAGTCGTCCTCTTCTCGAGCACCACCACCACCACCACTGA

MESGFTSKDTYLSHFNPRDYLEKYYKFGSRHSAESQILKHLLKNLFKIFCLDGVKGDLLIDIGSGPTIYQLLSACESFKEIVVTDYSDQNLQEL
EKWLKKEPEAFDWSPVVTYVCDLEGNRVKGPEKEEKLRQAVKQVLKCDVTQSQPLGAVPLPPADCVLSTLCLHAACPDLPTYCRALRNLGSLLK
PGGFLVIMDALKASYYMIGEQKFSHLPLGREAVEAAVKEAGYTIEWFEVISQSYSSTMMNCEGLFSLVARKLSRPLLEHHHHHH

>V23
ATGGAGAGTGGATTTACGAGTAAGGACACTTATTTGAGTCATTTTAACCCGCGCGACTACTTAGAAAAATACTACAAGTTTGGAAGCCGCCATT
CAGCAGAATCACAAATTCTGAAACACCTTTTAAAGAACCTGTTTAAAATCTTCTGTTTAGATGGGGTCAAAGGAGATTTGTTGATTGACATCGG
ATCAGGTCCCACAATTTACCAACTTCTGTCCGCTTGTGAGAGTTTTAAGGAGATCGTCGTGACGGACTATTCAGATCAAAATCTGCAAGAACTG
GAGAAATGGTTAAAGAAAGAACCAGAGGCGTTCGATTGGAGCCCAGTTGTGACCTATGTCTGTGATCTTGAAGGAAACCGCGTAAAGGGTCCTG
AAAAGGAGGAAAAGCTGCGCCAAGCCGTAAAGCAAGTATTAAAATGTGATGTTACTCAAAGCCAACCATTAGGAGCCGTCCCACTGCCCCCGGC
GGATTGTGTATTGTCAACCCTTTGCTTGGACGCAGCTTGTCCTGACTTGCCTACATATTGTCGTGCATTACGCAACTTAGGAAGTTTGCTTAAA
CCAGGAGGGTTTCTGGTCATCATGGgTaCCTTGAAGTCATCCTACTACATGATTGGGGAACAGAAGTTCTCAgCgTTACCTTTGGGCCGTGAAG
CCGTAGAAGCGGCCGTAAAGGAGGCGGGGTACACCATTGAATGGTTCGAGGTTATTTCACAGTCCTggAGCTCCACAATGGCAAATgcCGAGGG
CTTATTTTCGCTGGTTGCTCGCAAACTGAGTCGTCCTCTTCTCGAGCACCACCACCACCACCACTGA

MESGFTSKDTYLSHFNPRDYLEKYYKFGSRHSAESQILKHLLKNLFKIFCLDGVKGDLLIDIGSGPTIYQLLSACESFKEIVVTDYSDQNLQEL
EKWLKKEPEAFDWSPVVTYVCDLEGNRVKGPEKEEKLRQAVKQVLKCDVTQSQPLGAVPLPPADCVLSTLCLDAACPDLPTYCRALRNLGSLLK
PGGFLVIMGTLKSSYYMIGEQKFSALPLGREAVEAAVKEAGYTIEWFEVISQSWSSTMANAEGLFSLVARKLSRPLLEHHHHHH

>v24
ATGGAGAGTGGATTTACGAGTAAGGACACTTATTTGAGTCATTTTAACCCGCGCGACTACTTAGAAAAATtCTACAAGTTTGGAAGCCGCCATT
CAGCAGAATCACAAATTCTGAAACACCTTTTAAAGAACCTGTTTAAAATCTTCTGTTTAGATGGGGTCAAAGGAGATTTGTTGATTGACATCGG
ATCAGGTCCCACAATTTACCAACTTCTGTCCGCTTGTGAGAGTTTTAAGGAGATCGTCGTGACGGACTATTCAGATCAAAATCTGCAAGAACTG
GAGAAATGGTTAAAGAAAGAACCAGAGGCGTTCGATTGGAGCCCAGTTGTGACCTATGTCTGTGATCTTGAAGGAAACCGCGTAAAGGGTCCTG
AAAAGGAGGAAAAGCTGCGCCAAGCCGTAAAGCAAGTATTAAAATGTGATGTTACTCAAAGCCAACCATTAGGAGCCGTCCCACTGCCCCCGGC
GGATTGTGTATTGTCAACCCTTTGCTTGcACGCAGCTTGTCCTGACTTGCCTACATATTGTCGTGCATTACGCAACTTAGGAAGTTTGCTTAAA
CCAGGAGGGTTTCTGGTCATCATGGATGCCTTGAAGcagTCCTACTACATGATTGGGGAACAGAAGTTCTCAgCgTTACCTTTGGGCCGTGAAG
CCGTAGAAGCGGCCGTAAAGGAGGCGGGGTACACCATTGAATGGTTCGAGGTTATTTCACAGTCCTtCAGCTCCACAATGGCAAATAACGAGGG
CTTATTTTCGCTGGTTGCTCGCAAACTGAGTCGTCCTCTTCTCGAGCACCACCACCACCACCACTGA

MESGFTSKDTYLSHFNPRDYLEKFYKFGSRHSAESQILKHLLKNLFKIFCLDGVKGDLLIDIGSGPTIYQLLSACESFKEIVVTDYSDQNLQEL
EKWLKKEPEAFDWSPVVTYVCDLEGNRVKGPEKEEKLRQAVKQVLKCDVTQSQPLGAVPLPPADCVLSTLCLHAACPDLPTYCRALRNLGSLLK
PGGFLVIMDALKQSYYMIGEQKFSALPLGREAVEAAVKEAGYTIEWFEVISQSFSSTMANNEGLFSLVARKLSRPLLEHHHHHH

>v25
ATGGAGAGTGGATTTACGAGTAAGGACACTTATTTGAGTCATTTTAACCCGCGCGACTACTTAGAAAAATtCTACAAGTTTGGAAGCCGCCATT
CAGCAGAATCACAAATTCTGAAACACCTTTTAAAGAACCTGTTTAAAATCTTCTGTTTAGATGGGGTCAAAGGAGATTTGTTGATTGACATCGG
ATCAGGTCCCACAATTTACCAACTTCTGTCCGCTTGTGAGAGTTTTAAGGAGATCGTCGTGACGGACTATTCAGATCAAAATCTGCAAGAACTG
GAGAAATGGTTAAAGAAAGAACCAGAGGCGTTCGATTGGAGCCCAGTTGTGACCTATGTCTGTGATCTTGAAGGAAACCGCGTAAAGGGTCCTG
AAAAGGAGGAAAAGCTGCGCCAAGCCGTAAAGCAAGTATTAAAATGTGATGTTACTCAAAGCCAACCATTAGGAGCCGTCCCACTGCCCCCGGC
GGATTGTGTATTGTCAACCCTTTGCTTGcACGCAGCTTGTCCTGACTTGCCTACATATTGTCGTGCATTACGCAACTTAGGAAGTTTGCTTAAA
CCAGGAGGGTTTCTGGTCATCATGGATatgTTGAAGTCATCCTACTACATGATTGGGGAACAGAAGTTCTCAgCgTTACCTTTGGGCCGTGAAG
CCGTAGAAGCGGCCGTAAAGGAGGCGGGGTACACCATTGAATGGTTCGAGGTTATTTCACAGTCCTACAGCTCCACAATGGCAAATAACGAGGG
CTTATTTTCGCTGGTTGCTCGCAAACTGAGTCGTCCTCTTCTCGAGCACCACCACCACCACCACTGA

MESGFTSKDTYLSHFNPRDYLEKFYKFGSRHSAESQILKHLLKNLFKIFCLDGVKGDLLIDIGSGPTIYQLLSACESFKEIVVTDYSDQNLQEL
EKWLKKEPEAFDWSPVVTYVCDLEGNRVKGPEKEEKLRQAVKQVLKCDVTQSQPLGAVPLPPADCVLSTLCLHAACPDLPTYCRALRNLGSLLK
PGGFLVIMDMLKSSYYMIGEQKFSALPLGREAVEAAVKEAGYTIEWFEVISQSYSSTMANNEGLFSLVARKLSRPLLEHHHHHH

>v26
ATGGAGAGTGGATTTACGAGTAAGGACACTTATTTGAGTCATTTTAACCCGCGCGACTACTTAGAAAAATACTACAAGTTTGGAAGCCGCCATT
CAGCAGAATCACAAATTCTGAAACACCTTTTAAAGAACCTGTTTAAAATCTTCTGTTTAGATGGGGTCAAAGGAGATTTGTTGATTGACATCGG
ATCAGGTCCCACAATTTACCAACTTCTGTCCGCTTGTGAGAGTTTTAAGGAGATCGTCGTGACGGACTATTCAGATCAAAATCTGCAAGAACTG
GAGAAATGGTTAAAGAAAGAACCAGAGGCGTTCGATTGGAGCCCAGTTGTGACCTATGTCTGTGATCTTGAAGGAAACCGCGTAAAGGGTCCTG
AAAAGGAGGAAAAGCTGCGCCAAGCCGTAAAGCAAGTATTAAAATGTGATGTTACTCAAAGCCAACCATTAGGAGCCGTCCCACTGCCCCCGGC
GGATTGTGTATTGTCAACCCTTTGCTTGcACGCAGCTTGTCCTGACTTGCCTACATATTGTCGTGCATTACGCAACTTAGGAAGTTTGCTTAAA
CCAGGAGGGTTTCTGGTCATCATGGATaCCTTGAAGcagTCCTACTACATGATTGGGGAACAGAAGTTCTCATCTTTACCTTTGGGCCGTGAAG
CCGTAGAAGCGGCCGTAAAGGAGGCGGGGTACACCATTGAATGGTTCGAGGTTATTTCACAGTCCTACAGCTCCACAATGGCAAATAACGAGGG
CTTATTTTCGCTGGTTGCTCGCAAACTGAGTCGTCCTCTTCTCGAGCACCACCACCACCACCACTGA

MESGFTSKDTYLSHFNPRDYLEKYYKFGSRHSAESQILKHLLKNLFKIFCLDGVKGDLLIDIGSGPTIYQLLSACESFKEIVVTDYSDQNLQEL
EKWLKKEPEAFDWSPVVTYVCDLEGNRVKGPEKEEKLRQAVKQVLKCDVTQSQPLGAVPLPPADCVLSTLCLHAACPDLPTYCRALRNLGSLLK
PGGFLVIMDTLKQSYYMIGEQKFSSLPLGREAVEAAVKEAGYTIEWFEVISQSYSSTMANNEGLFSLVARKLSRPLLEHHHHHH

>v27
ATGGAGAGTGGATTTACGAGTAAGGACACTTATTTGAGTCATTTTAACCCGCGCGACTACTTAGAAAAATACTACAAGTTTGGAAGCCGCCATT
CAGCAGAATCACAAATTCTGAAACACCTTTTAAAGAACCTGTTTAAAATCTTCTGTTTAGATGGGGTCAAAGGAGATTTGTTGATTGACATCGG
ATCAGGTCCCACAATTTACCAACTTCTGTCCGCTTGTGAGAGTTTTAAGGAGATCGTCGTGACGGACTATTCAGATCAAAATCTGCAAGAACTG
GAGAAATGGTTAAAGAAAGAACCAGAGGCGTTCGATTGGAGCCCAGTTGTGACCTATGTCTGTGATCTTGAAGGAAACCGCGTAAAGGGTCCTG
AAAAGGAGGAAAAGCTGCGCCAAGCCGTAAAGCAAGTATTAAAATGTGATGTTACTCAAAGCCAACCATTAGGAGCCGTCCCACTGCCCCCGGC
GGATTGTGTATTGTCAACCCTTTGCTTGtgCGCAGCTTGTCCTGACTTGCCTACATATTGTCGTGCATTACGCAACTTAGGAAGTTTGCTTAAA
CCAGGAGGGTTTCTGGTCATCATGGATtCCTTGAAGTCATCCTACTACATGATTGGGGAACAGAAGTTCTCAgCgTTACCTTTGGGCCGTGAAG
CCGTAGAAGCGGCCGTAAAGGAGGCGGGGTACACCATTGAATGGTTCGAGGTTATTTCACAGTCCTggAGCTCCACAATGGCAAATgcCGAGGG
CTTATTTTCGCTGGTTGCTCGCAAACTGAGTCGTCCTCTTCTCGAGCACCACCACCACCACCACTGA

MESGFTSKDTYLSHFNPRDYLEKYYKFGSRHSAESQILKHLLKNLFKIFCLDGVKGDLLIDIGSGPTIYQLLSACESFKEIVVTDYSDQNLQEL
EKWLKKEPEAFDWSPVVTYVCDLEGNRVKGPEKEEKLRQAVKQVLKCDVTQSQPLGAVPLPPADCVLSTLCLCAACPDLPTYCRALRNLGSLLK
PGGFLVIMDSLKSSYYMIGEQKFSALPLGREAVEAAVKEAGYTIEWFEVISQSWSSTMANAEGLFSLVARKLSRPLLEHHHHHH

>v28
ATGGAGAGTGGATTTACGAGTAAGGACACTTATTTGAGTCATTTTAACCCGCGCGACTACTTAGAAAAATACTACAAGTTTGGAAGCCGCCATT
CAGCAGAATCACAAATTCTGAAACACCTTTTAAAGAACCTGTTTAAAATCTTCTGTTTAGATGGGGTCAAAGGAGATTTGTTGATTGACATCGG
ATCAGGTCCCACAATTTACCAACTTCTGTCCGCTTGTGAGAGTTTTAAGGAGATCGTCGTGACGGACTATTCAGATCAAAATCTGCAAGAACTG
GAGAAATGGTTAAAGAAAGAACCAGAGGCGTTCGATTGGAGCCCAGTTGTGACCTATGTCTGTGATCTTGAAGGAAACCGCGTAAAGGGTCCTG
AAAAGGAGGAAAAGCTGCGCCAAGCCGTAAAGCAAGTATTAAAATGTGATGTTACTCAAAGCCAACCATTAGGAGCCGTCCCACTGCCCCCGGC
GGATTGTGTATTGTCAACCCTTTGCTTGcACGCAGCTTGTCCTGACTTGCCTACATATTGTCGTGCATTACGCAACTTAGGAAGTTTGCTTAAA
CCAGGAGGGTTTCTGGTCATCATGGATGCCTTGAAGTgcTCCTACTACATGATTGGGGAACAGAAGTTCTCAatgTTACCTTTGGGCCGTGAAG
CCGTAGAAGCGGCCGTAAAGGAGGCGGGGTACACCATTGAATGGTTCGAGGTTATTTCACAGTCCTACAGCTCCACAATGGCAAATgcCGAGGG
CTTATTTTCGCTGGTTGCTCGCAAACTGAGTCGTCCTCTTCTCGAGCACCACCACCACCACCACTGA

MESGFTSKDTYLSHFNPRDYLEKYYKFGSRHSAESQILKHLLKNLFKIFCLDGVKGDLLIDIGSGPTIYQLLSACESFKEIVVTDYSDQNLQEL
EKWLKKEPEAFDWSPVVTYVCDLEGNRVKGPEKEEKLRQAVKQVLKCDVTQSQPLGAVPLPPADCVLSTLCLHAACPDLPTYCRALRNLGSLLK
PGGFLVIMDALKCSYYMIGEQKFSMLPLGREAVEAAVKEAGYTIEWFEVISQSYSSTMANAEGLFSLVARKLSRPLLEHHHHHH

>v29
ATGGAGAGTGGATTTACGAGTAAGGACACTTATTTGAGTCATTTTAACCCGCGCGACTACTTAGAAAAATACTACAAGTTTGGAAGCCGCCATT
CAGCAGAATCACAAATTCTGAAACACCTTTTAAAGAACCTGTTTAAAATCTTCTGTTTAGATGGGGTCAAAGGAGATTTGTTGATTGACATCGG
ATCAGGTCCCACAATTTACCAACTTCTGTCCGCTTGTGAGAGTTTTAAGGAGATCGTCGTGACGGACTATTCAGATCAAAATCTGCAAGAACTG
GAGAAATGGTTAAAGAAAGAACCAGAGGCGTTCGATTGGAGCCCAGTTGTGACCTATGTCTGTGATCTTGAAGGAAACCGCGTAAAGGGTCCTG
AAAAGGAGGAAAAGCTGCGCCAAGCCGTAAAGCAAGTATTAAAATGTGATGTTACTCAAAGCCAACCATTAGGAGCCGTCCCACTGCCCCCGGC
GGATTGTGTATTGTCAACCCTTTGCTTGtgCGCAGCTTGTCCTGACTTGCCTACATATTGTCGTGCATTACGCAACTTAGGAAGTTTGCTTAAA
CCAGGAGGGTTTCTGGTCATCATGGATGCCTTGAAGaCcTCCTACTACATGATTGGGGAACAGAAGTTCTCAgCgTTACCTTTGGGCCGTGAAG
CCGTAGAAGCGGCCGTAAAGGAGGCGGGGTACACCATTGAATGGTTCGAGGTTATTTCACAGTCCTtCAGCTCCACAATGGCAAATgcCGAGGG
CTTATTTTCGCTGGTTGCTCGCAAACTGAGTCGTCCTCTTCTCGAGCACCACCACCACCACCACTGA

MESGFTSKDTYLSHFNPRDYLEKYYKFGSRHSAESQILKHLLKNLFKIFCLDGVKGDLLIDIGSGPTIYQLLSACESFKEIVVTDYSDQNLQEL
EKWLKKEPEAFDWSPVVTYVCDLEGNRVKGPEKEEKLRQAVKQVLKCDVTQSQPLGAVPLPPADCVLSTLCLCAACPDLPTYCRALRNLGSLLK
PGGFLVIMDALKTSYYMIGEQKFSALPLGREAVEAAVKEAGYTIEWFEVISQSFSSTMANAEGLFSLVARKLSRPLLEHHHHHH

>v30
ATGGAGAGTGGATTTACGAGTAAGGACACTTATTTGAGTCATTTTAACCCGCGCGACTACTTAGAAAAATACTACAAGTTTGGAAGCCGCCATT
CAGCAGAATCACAAATTCTGAAACACCTTTTAAAGAACCTGTTTAAAATCTTCTGTTTAGATGGGGTCAAAGGAGATTTGTTGATTGACATCGG
ATCAGGTCCCACAATTTACCAACTTCTGTCCGCTTGTGAGAGTTTTAAGGAGATCGTCGTGACGGACTATTCAGATCAAAATCTGCAAGAACTG
GAGAAATGGTTAAAGAAAGAACCAGAGGCGTTCGATTGGAGCCCAGTTGTGACCTATGTCTGTGATCTTGAAGGAAACCGCGTAAAGGGTCCTG
AAAAGGAGGAAAAGCTGCGCCAAGCCGTAAAGCAAGTATTAAAATGTGATGTTACTCAAAGCCAACCATTAGGAGCCGTCCCACTGCCCCCGGC
GGATTGTGTATTGTCAACCCTTTGCTTGtgCGCAGCTTGTCCTGACTTGCCTACATATTGTCGTGCATTACGCAACTTAGGAAGTTTGCTTAAA
CCAGGAGGGTTTCTGGTCATCATGGATGCCTTGAAGcagTCCTACTACATGATTGGGGAACAGAAGTTCTCATCTTTACCTTTGGGCCGTGAAG
CCGTAGAAGCGGCCGTAAAGGAGGCGGGGTACACCATTGAATGGTTCGAGGTTATTTCACAGTCCTggAGCTCCACAATGGCAAATtcCGAGGG
CTTATTTTCGCTGGTTGCTCGCAAACTGAGTCGTCCTCTTCTCGAGCACCACCACCACCACCACTGA

MESGFTSKDTYLSHFNPRDYLEKYYKFGSRHSAESQILKHLLKNLFKIFCLDGVKGDLLIDIGSGPTIYQLLSACESFKEIVVTDYSDQNLQEL
EKWLKKEPEAFDWSPVVTYVCDLEGNRVKGPEKEEKLRQAVKQVLKCDVTQSQPLGAVPLPPADCVLSTLCLCAACPDLPTYCRALRNLGSLLK
PGGFLVIMDALKQSYYMIGEQKFSSLPLGREAVEAAVKEAGYTIEWFEVISQSWSSTMANSEGLFSLVARKLSRPLLEHHHHHH

>v31
ATGGAGAGTGGATTTACGAGTAAGGACACTTATTTGAGTCATTTTAACCCGCGCGACTACTTAGAAAAATACTACAAGTTTGGAAGCCGCCATT
CAGCAGAATCACAAATTCTGAAACACCTTTTAAAGAACCTGTTTAAAATCTTCTGTTTAGATGGGGTCAAAGGAGATTTGTTGATTGACATCGG
ATCAGGTCCCACAATTTACCAACTTCTGTCCGCTTGTGAGAGTTTTAAGGAGATCGTCGTGACGGACTATTCAGATCAAAATCTGCAAGAACTG
GAGAAATGGTTAAAGAAAGAACCAGAGGCGTTCGATTGGAGCCCAGTTGTGACCTATGTCTGTGATCTTGAAGGAAACCGCGTAAAGGGTCCTG
AAAAGGAGGAAAAGCTGCGCCAAGCCGTAAAGCAAGTATTAAAATGTGATGTTACTCAAAGCCAACCATTAGGAGCCGTCCCACTGCCCCCGGC
GGATTGTGTATTGTCAACCCTTTGCTTGcACGCAGCTTGTCCTGACTTGCCTACATATTGTCGTGCATTACGCAACTTAGGAAGTTTGCTTAAA
CCAGGAGGGTTTCTGGTCATCATGGATatgTTGAAGTgcTCCTACTACATGATTGGGGAACAGAAGTTCTCATCTTTACCTTTGGGCCGTGAAG
CCGTAGAAGCGGCCGTAAAGGAGGCGGGGTACACCATTGAATGGTTCGAGGTTATTTCACAGTCCTtCAGCTCCACAATGGCAAATtcCGAGGG
CTTATTTTCGCTGGTTGCTCGCAAACTGAGTCGTCCTCTTCTCGAGCACCACCACCACCACCACTGA

MESGFTSKDTYLSHFNPRDYLEKYYKFGSRHSAESQILKHLLKNLFKIFCLDGVKGDLLIDIGSGPTIYQLLSACESFKEIVVTDYSDQNLQEL
EKWLKKEPEAFDWSPVVTYVCDLEGNRVKGPEKEEKLRQAVKQVLKCDVTQSQPLGAVPLPPADCVLSTLCLHAACPDLPTYCRALRNLGSLLK
PGGFLVIMDMLKCSYYMIGEQKFSSLPLGREAVEAAVKEAGYTIEWFEVISQSFSSTMANSEGLFSLVARKLSRPLLEHHHHHH

>v32
ATGGAGAGTGGATTTACGAGTAAGGACACTTATTTGAGTCATTTTAACCCGCGCGACTACTTAGAAAAAttCTACAAGTTTGGAAGCCGCCATT
CAGCAGAATCACAAATTCTGAAACACCTTTTAAAGAACCTGTTTAAAATCTTCTGTTTAGATGGGGTCAAAGGAGATTTGTTGATTGACATCGG
ATCAGGTCCCACAATTTACCAACTTCTGTCCGCTTGTGAGAGTTTTAAGGAGATCGTCGTGACGGACTATTCAGATCAAAATCTGCAAGAACTG
GAGAAATGGTTAAAGAAAGAACCAGAGGCGTTCGATTGGAGCCCAGTTGTGACCTATGTCTGTGATCTTGAAGGAAACCGCGTAAAGGGTCCTG
AAAAGGAGGAAAAGCTGCGCCAAGCCGTAAAGCAAGTATTAAAATGTGATGTTACTCAAAGCCAACCATTAGGAGCCGTCCCACTGCCCCCGGC
GGATTGTGTATTGTCAACCCTTTGCTTGcACGCAGCTTGTCCTGACTTGCCTACATATTGTCGTGCATTACGCAACTTAGGAAGTTTGCTTAAA
CCAGGAGGGTTTCTGGTCATCATGGATGCCTTGAAGcagTCCTACTACATGATTGGGGAACAGAAGTTCTCATCTTTACCTTTGGGCCGTGAAG
CCGTAGAAGCGGCCGTAAAGGAGGCGGGGTACACCATTGAATGGTTCGAGGTTATTTCACAGTCCTACAGCTCCACAATGGCAAATtgCGAGGG
CTTATTTTCGCTGGTTGCTCGCAAACTGAGTCGTCCTCTTCTCGAGCACCACCACCACCACCACTGA

MESGFTSKDTYLSHFNPRDYLEKFYKFGSRHSAESQILKHLLKNLFKIFCLDGVKGDLLIDIGSGPTIYQLLSACESFKEIVVTDYSDQNLQEL
EKWLKKEPEAFDWSPVVTYVCDLEGNRVKGPEKEEKLRQAVKQVLKCDVTQSQPLGAVPLPPADCVLSTLCLHAACPDLPTYCRALRNLGSLLK
PGGFLVIMDALKQSYYMIGEQKFSSLPLGREAVEAAVKEAGYTIEWFEVISQSYSSTMANCEGLFSLVARKLSRPLLEHHHHHH

>v33
ATGGAGAGTGGATTTACGAGTAAGGACACTTATTTGAGTCATTTTAACCCGCGCGACTACTTAGAAAAATACTACAAGTTTGGAAGCCGCCATT
CAGCAGAATCACAAATTCTGAAACACCTTTTAAAGAACCTGTTTAAAATCTTCTGTTTAGATGGGGTCAAAGGAGATTTGTTGATTGACATCGG
ATCAGGTCCCACAATTTACCAACTTCTGTCCGCTTGTGAGAGTTTTAAGGAGATCGTCGTGACGGACTATTCAGATCAAAATCTGCAAGAACTG
GAGAAATGGTTAAAGAAAGAACCAGAGGCGTTCGATTGGAGCCCAGTTGTGACCTATGTCTGTGATCTTGAAGGAAACCGCGTAAAGGGTCCTG
AAAAGGAGGAAAAGCTGCGCCAAGCCGTAAAGCAAGTATTAAAATGTGATGTTACTCAAAGCCAACCATTAGGAGCCGTCCCACTGCCCCCGGC
GGATTGTGTATTGTCAACCCTTTGCTTGtgCGCAGCTTGTCCTGACTTGCCTACATATTGTCGTGCATTACGCAACTTAGGAAGTTTGCTTAAA
CCAGGAGGGTTTCTGGTCATCATGGATatgTTGAAGTgcTCCTACTACATGATTGGGGAACAGAAGTTCTCATgTTTACCTTTGGGCCGTGAAG
CCGTAGAAGCGGCCGTAAAGGAGGCGGGGTACACCATTGAATGGTTCGAGGTTATTTCACAGTCCTtCAGCTCCACAATGGCAAATAACGAGGG
CTTATTTTCGCTGGTTGCTCGCAAACTGAGTCGTCCTCTTCTCGAGCACCACCACCACCACCACTGA

MESGFTSKDTYLSHFNPRDYLEKYYKFGSRHSAESQILKHLLKNLFKIFCLDGVKGDLLIDIGSGPTIYQLLSACESFKEIVVTDYSDQNLQEL
EKWLKKEPEAFDWSPVVTYVCDLEGNRVKGPEKEEKLRQAVKQVLKCDVTQSQPLGAVPLPPADCVLSTLCLCAACPDLPTYCRALRNLGSLLK
PGGFLVIMDMLKCSYYMIGEQKFSCLPLGREAVEAAVKEAGYTIEWFEVISQSFSSTMANNEGLFSLVARKLSRPLLEHHHHHH

>v34
ATGGAGAGTGGATTTACGAGTAAGGACACTTATTTGAGTCATTTTAACCCGCGCGACTACTTAGAAAAATACTACAAGTTTGGAAGCCGCCATT
CAGCAGAATCACAAATTCTGAAACACCTTTTAAAGAACCTGTTTAAAATCTTCTGTTTAGATGGGGTCAAAGGAGATTTGTTGATTGACATCGG
ATCAGGTCCCACAATTTACCAACTTCTGTCCGCTTGTGAGAGTTTTAAGGAGATCGTCGTGACGGACTATTCAGATCAAAATCTGCAAGAACTG
GAGAAATGGTTAAAGAAAGAACCAGAGGCGTTCGATTGGAGCCCAGTTGTGACCTATGTCTGTGATCTTGAAGGAAACCGCGTAAAGGGTCCTG
AAAAGGAGGAAAAGCTGCGCCAAGCCGTAAAGCAAGTATTAAAATGTGATGTTACTCAAAGCCAACCATTAGGAGCCGTCCCACTGCCCCCGGC
GGATTGTGTATTGTCAACCCTTTGCTTGcACGCAGCTTGTCCTGACTTGCCTACATATTGTCGTGCATTACGCAACTTAGGAAGTTTGCTTAAA
CCAGGAGGGTTTCTGGTCATCATGGATGCCTTGAAGgaATCCTACTACATGATTGGGGAACAGAAGTTCTCAgCgTTACCTTTGGGCCGTGAAG
CCGTAGAAGCGGCCGTAAAGGAGGCGGGGTACACCATTGAATGGTTCGAGGTTATTTCACAGTCCTtCAGCTCCACAATGGCAAATtgCGAGGG
CTTATTTTCGCTGGTTGCTCGCAAACTGAGTCGTCCTCTTCTCGAGCACCACCACCACCACCACTGA

MESGFTSKDTYLSHFNPRDYLEKYYKFGSRHSAESQILKHLLKNLFKIFCLDGVKGDLLIDIGSGPTIYQLLSACESFKEIVVTDYSDQNLQEL
EKWLKKEPEAFDWSPVVTYVCDLEGNRVKGPEKEEKLRQAVKQVLKCDVTQSQPLGAVPLPPADCVLSTLCLHAACPDLPTYCRALRNLGSLLK
PGGFLVIMDALKESYYMIGEQKFSALPLGREAVEAAVKEAGYTIEWFEVISQSFSSTMANCEGLFSLVARKLSRPLLEHHHHHH

>v35
ATGGAGAGTGGATTTACGAGTAAGGACACTTATTTGAGTCATTTTAACCCGCGCGACTACTTAGAAAAATACTACAAGTTTGGAAGCCGCCATT
CAGCAGAATCACAAATTCTGAAACACCTTTTAAAGAACCTGTTTAAAATCTTCTGTTTAGATGGGGTCAAAGGAGATTTGTTGATTGACATCGG
ATCAGGTCCCACAATTTACCAACTTCTGTCCGCTTGTGAGAGTTTTAAGGAGATCGTCGTGACGGACTATTCAGATCAAAATCTGCAAGAACTG
GAGAAATGGTTAAAGAAAGAACCAGAGGCGTTCGATTGGAGCCCAGTTGTGACCTATGTCTGTGATCTTGAAGGAAACCGCGTAAAGGGTCCTG
AAAAGGAGGAAAAGCTGCGCCAAGCCGTAAAGCAAGTATTAAAATGTGATGTTACTCAAAGCCAACCATTAGGAGCCGTCCCACTGCCCCCGGC
GGATTGTGTATTGTCAACCCTTTGCTTGGACGCAGCTTGTCCTGACTTGCCTACATATTGTCGTGCATTACGCAACTTAGGAAGTTTGCTTAAA
CCAGGAGGGTTTCTGGTCATCATGGgTctgTTGAAGgCgTCCTACTACATGATTGGGGAACAGAAGTTCTCAgCgTTACCTTTGGGCCGTGAAG
CCGTAGAAGCGGCCGTAAAGGAGGCGGGGTACACCATTGAATGGTTCGAGGTTATTTCACAGTCCTtCAGCTCCACAATGGCAAATAACGAGGG
CTTATTTTCGCTGGTTGCTCGCAAACTGAGTCGTCCTCTTCTCGAGCACCACCACCACCACCACTGA

MESGFTSKDTYLSHFNPRDYLEKYYKFGSRHSAESQILKHLLKNLFKIFCLDGVKGDLLIDIGSGPTIYQLLSACESFKEIVVTDYSDQNLQEL
EKWLKKEPEAFDWSPVVTYVCDLEGNRVKGPEKEEKLRQAVKQVLKCDVTQSQPLGAVPLPPADCVLSTLCLDAACPDLPTYCRALRNLGSLLK
PGGFLVIMGLLKASYYMIGEQKFSALPLGREAVEAAVKEAGYTIEWFEVISQSFSSTMANNEGLFSLVARKLSRPLLEHHHHHH

>v36
ATGGAGAGTGGATTTACGAGTAAGGACACTTATTTGAGTCATTTTAACCCGCGCGACTACTTAGAAAAATtCTACAAGTTTGGAAGCCGCCATT
CAGCAGAATCACAAATTCTGAAACACCTTTTAAAGAACCTGTTTAAAATCTTCTGTTTAGATGGGGTCAAAGGAGATTTGTTGATTGACATCGG
ATCAGGTCCCACAATTTACCAACTTCTGTCCGCTTGTGAGAGTTTTAAGGAGATCGTCGTGACGGACTATTCAGATCAAAATCTGCAAGAACTG
GAGAAATGGTTAAAGAAAGAACCAGAGGCGTTCGATTGGAGCCCAGTTGTGACCTATGTCTGTGATCTTGAAGGAAACCGCGTAAAGGGTCCTG
AAAAGGAGGAAAAGCTGCGCCAAGCCGTAAAGCAAGTATTAAAATGTGATGTTACTCAAAGCCAACCATTAGGAGCCGTCCCACTGCCCCCGGC
GGATTGTGTATTGTCAACCCTTTGCTTGtgCGCAGCTTGTCCTGACTTGCCTACATATTGTCGTGCATTACGCAACTTAGGAAGTTTGCTTAAA
CCAGGAGGGTTTCTGGTCATCATGGATaCCTTGAAGTgcTCCTACTACATGATTGGGGAACAGAAGTTCTCAgCgTTACCTTTGGGCCGTGAAG
CCGTAGAAGCGGCCGTAAAGGAGGCGGGGTACACCATTGAATGGTTCGAGGTTATTTCACAGTCCTACAGCTCCACAATGGCAAATAACGAGGG
CTTATTTTCGCTGGTTGCTCGCAAACTGAGTCGTCCTCTTCTCGAGCACCACCACCACCACCACTGA

MESGFTSKDTYLSHFNPRDYLEKFYKFGSRHSAESQILKHLLKNLFKIFCLDGVKGDLLIDIGSGPTIYQLLSACESFKEIVVTDYSDQNLQEL
EKWLKKEPEAFDWSPVVTYVCDLEGNRVKGPEKEEKLRQAVKQVLKCDVTQSQPLGAVPLPPADCVLSTLCLCAACPDLPTYCRALRNLGSLLK
PGGFLVIMDTLKCSYYMIGEQKFSALPLGREAVEAAVKEAGYTIEWFEVISQSYSSTMANNEGLFSLVARKLSRPLLEHHHHHH

>v37
ATGGAGAGTGGATTTACGAGTAAGGACACTTATTTGAGTCATTTTAACCCGCGCGACTACTTAGAAAAATACTACAAGTTTGGAAGCCGCCATT
CAGCAGAATCACAAATTCTGAAACACCTTTTAAAGAACCTGTTTAAAATCTTCTGTTTAGATGGGGTCAAAGGAGATTTGTTGATTGACATCGG
ATCAGGTCCCACAATTTACCAACTTCTGTCCGCTTGTGAGAGTTTTAAGGAGATCGTCGTGACGGACTATTCAGATCAAAATCTGCAAGAACTG
GAGAAATGGTTAAAGAAAGAACCAGAGGCGTTCGATTGGAGCCCAGTTGTGACCTATGTCTGTGATCTTGAAGGAAACCGCGTAAAGGGTCCTG
AAAAGGAGGAAAAGCTGCGCCAAGCCGTAAAGCAAGTATTAAAATGTGATGTTACTCAAAGCCAACCATTAGGAGCCGTCCCACTGCCCCCGGC
GGATTGTGTATTGTCAACCCTTTGCTTGcACGCAGCTTGTCCTGACTTGCCTACATATTGTCGTGCATTACGCAACTTAGGAAGTTTGCTTAAA
CCAGGAGGGTTTCTGGTCATCATGGATaCCTTGAAGgaATCCTACTACATGATTGGGGAACAGAAGTTCTCAgCgTTACCTTTGGGCCGTGAAG
CCGTAGAAGCGGCCGTAAAGGAGGCGGGGTACACCATTGAATGGTTCGAGGTTATTTCACAGTCCTggAGCTCCACAATGGCAAATAACGAGGG
CTTATTTTCGCTGGTTGCTCGCAAACTGAGTCGTCCTCTTCTCGAGCACCACCACCACCACCACTGA

MESGFTSKDTYLSHFNPRDYLEKYYKFGSRHSAESQILKHLLKNLFKIFCLDGVKGDLLIDIGSGPTIYQLLSACESFKEIVVTDYSDQNLQEL
EKWLKKEPEAFDWSPVVTYVCDLEGNRVKGPEKEEKLRQAVKQVLKCDVTQSQPLGAVPLPPADCVLSTLCLHAACPDLPTYCRALRNLGSLLK
PGGFLVIMDTLKESYYMIGEQKFSALPLGREAVEAAVKEAGYTIEWFEVISQSWSSTMANNEGLFSLVARKLSRPLLEHHHHHH

>v38
ATGGAGAGTGGATTTACGAGTAAGGACACTTATTTGAGTCATTTTAACCCGCGCGACTtCTTAGAAAAATACTACAAGTTTGGAAGCCGCCATT
CAGCAGAATCACAAATTCTGAAACACCTTTTAAAGAACCTGTTTAAAATCTTCTGTTTAGATGGGGTCAAAGGAGATTTGTTGATTGACATCGG
ATCAGGTCCCACAATTTACCAACTTCTGTCCGCTTGTGAGAGTTTTAAGGAGATCGTCGTGACGGACTATTCAGATCAAAATCTGCAAGAACTG
GAGAAATGGTTAAAGAAAGAACCAGAGGCGTTCGATTGGAGCCCAGTTGTGACCTATGTCTGTGATCTTGAAGGAAACCGCGTAAAGGGTCCTG
AAAAGGAGGAAAAGCTGCGCCAAGCCGTAAAGCAAGTATTAAAATGTGATGTTACTCAAAGCCAACCATTAGGAGCCGTCCCACTGCCCCCGGC
GGATTGTGTATTGTCAACCCTTTGCTTGtgCGCAGCTTGTCCTGACTTGCCTACATATTGTCGTGCATTACGCAACTTAGGAAGTTTGCTTAAA
CCAGGAGGGTTTCTGGTCATCATGGATGCCTTGAAGatgTCCTACTACATGATTGGGGAACAGAAGTTCTCATCTTTACCTTTGGGCCGTGAAG
CCGTAGAAGCGGCCGTAAAGGAGGCGGGGTACACCATTGAATGGTTCGAGGTTATTTCACAGTCCTtCAGCTCCACAATGGCAAATgcCGAGGG
CTTATTTTCGCTGGTTGCTCGCAAACTGAGTCGTCCTCTTCTCGAGCACCACCACCACCACCACTGA

MESGFTSKDTYLSHFNPRDFLEKYYKFGSRHSAESQILKHLLKNLFKIFCLDGVKGDLLIDIGSGPTIYQLLSACESFKEIVVTDYSDQNLQEL
EKWLKKEPEAFDWSPVVTYVCDLEGNRVKGPEKEEKLRQAVKQVLKCDVTQSQPLGAVPLPPADCVLSTLCLCAACPDLPTYCRALRNLGSLLK
PGGFLVIMDALKMSYYMIGEQKFSSLPLGREAVEAAVKEAGYTIEWFEVISQSFSSTMANAEGLFSLVARKLSRPLLEHHHHHH

>v39
ATGGAGAGTGGATTTACGAGTAAGGACACTTATTTGAGTCATTTTAACCCGCGCGACTtCTTAGAAAAATACTACAAGTTTGGAAGCCGCCATT
CAGCAGAATCACAAATTCTGAAACACCTTTTAAAGAACCTGTTTAAAATCTTCTGTTTAGATGGGGTCAAAGGAGATTTGTTGATTGACATCGG
ATCAGGTCCCACAATTTACCAACTTCTGTCCGCTTGTGAGAGTTTTAAGGAGATCGTCGTGACGGACTATTCAGATCAAAATCTGCAAGAACTG
GAGAAATGGTTAAAGAAAGAACCAGAGGCGTTCGATTGGAGCCCAGTTGTGACCTATGTCTGTGATCTTGAAGGAAACCGCGTAAAGGGTCCTG
AAAAGGAGGAAAAGCTGCGCCAAGCCGTAAAGCAAGTATTAAAATGTGATGTTACTCAAAGCCAACCATTAGGAGCCGTCCCACTGCCCCCGGC
GGATTGTGTATTGTCAACCCTTTGCTTGcACGCAGCTTGTCCTGACTTGCCTACATATTGTCGTGCATTACGCAACTTAGGAAGTTTGCTTAAA
CCAGGAGGGTTTCTGGTCATCATGGATatgTTGAAGTCATCCTACTACATGATTGGGGAACAGAAGTTCTCAgCgTTACCTTTGGGCCGTGAAG
CCGTAGAAGCGGCCGTAAAGGAGGCGGGGTACACCATTGAATGGTTCGAGGTTATTTCACAGTCCTACAGCTCCACAATGGCAAATgcCGAGGG
CTTATTTTCGCTGGTTGCTCGCAAACTGAGTCGTCCTCTTCTCGAGCACCACCACCACCACCACTGA

MESGFTSKDTYLSHFNPRDFLEKYYKFGSRHSAESQILKHLLKNLFKIFCLDGVKGDLLIDIGSGPTIYQLLSACESFKEIVVTDYSDQNLQEL
EKWLKKEPEAFDWSPVVTYVCDLEGNRVKGPEKEEKLRQAVKQVLKCDVTQSQPLGAVPLPPADCVLSTLCLHAACPDLPTYCRALRNLGSLLK
PGGFLVIMDMLKSSYYMIGEQKFSALPLGREAVEAAVKEAGYTIEWFEVISQSYSSTMANAEGLFSLVARKLSRPLLEHHHHHH

>v40
ATGGAGAGTGGATTTACGAGTAAGGACACTTATTTGAGTCATTTTAACCCGCGCGACTACTTAGAAAAATACTACAAGTTTGGAAGCCGCCATT
CAGCAGAATCACAAATTCTGAAACACCTTTTAAAGAACCTGTTTAAAATCTTCTGTTTAGATGGGGTCAAAGGAGATTTGTTGATTGACATCGG
ATCAGGTCCCACAATTTACCAACTTCTGTCCGCTTGTGAGAGTTTTAAGGAGATCGTCGTGACGGACTATTCAGATCAAAATCTGCAAGAACTG
GAGAAATGGTTAAAGAAAGAACCAGAGGCGTTCGATTGGAGCCCAGTTGTGACCTATGTCTGTGATCTTGAAGGAAACCGCGTAAAGGGTCCTG
AAAAGGAGGAAAAGCTGCGCCAAGCCGTAAAGCAAGTATTAAAATGTGATGTTACTCAAAGCCAACCATTAGGAGCCGTCCCACTGCCCCCGGC
GGATTGTGTATTGTCAACCCTTTGCTTGcACGCAGCTTGTCCTGACTTGCCTACATATTGTCGTGCATTACGCAACTTAGGAAGTTTGCTTAAA
CCAGGAGGGTTTCTGGTCATCATGGATctgTTGAAGgCgTCCTACTACATGATTGGGGAACAGAAGTTCTCATgtTTACCTTTGGGCCGTGAAG
CCGTAGAAGCGGCCGTAAAGGAGGCGGGGTACACCATTGAATGGTTCGAGGTTATTTCACAGTCCTACAGCTCCACAATGGCAAATtgCGAGGG
CTTATTTTCGCTGGTTGCTCGCAAACTGAGTCGTCCTCTTCTCGAGCACCACCACCACCACCACTGA

MESGFTSKDTYLSHFNPRDYLEKYYKFGSRHSAESQILKHLLKNLFKIFCLDGVKGDLLIDIGSGPTIYQLLSACESFKEIVVTDYSDQNLQEL
EKWLKKEPEAFDWSPVVTYVCDLEGNRVKGPEKEEKLRQAVKQVLKCDVTQSQPLGAVPLPPADCVLSTLCLHAACPDLPTYCRALRNLGSLLK
PGGFLVIMDLLKASYYMIGEQKFSCLPLGREAVEAAVKEAGYTIEWFEVISQSYSSTMANCEGLFSLVARKLSRPLLEHHHHHH

>v41
ATGGAGAGTGGATTTACGAGTAAGGACACTTATTTGAGTCATTTTAACCCGCGCGACTtCTTAGAAAAATACTACAAGTTTGGAAGCCGCCATT
CAGCAGAATCACAAATTCTGAAACACCTTTTAAAGAACCTGTTTAAAATCTTCTGTTTAGATGGGGTCAAAGGAGATTTGTTGATTGACATCGG
ATCAGGTCCCACAATTTACCAACTTCTGTCCGCTTGTGAGAGTTTTAAGGAGATCGTCGTGACGGACTATTCAGATCAAAATCTGCAAGAACTG
GAGAAATGGTTAAAGAAAGAACCAGAGGCGTTCGATTGGAGCCCAGTTGTGACCTATGTCTGTGATCTTGAAGGAAACCGCGTAAAGGGTCCTG
AAAAGGAGGAAAAGCTGCGCCAAGCCGTAAAGCAAGTATTAAAATGTGATGTTACTCAAAGCCAACCATTAGGAGCCGTCCCACTGCCCCCGGC
GGATTGTGTATTGTCAACCCTTTGCTTGcACGCAGCTTGTCCTGACTTGCCTACATATTGTCGTGCATTACGCAACTTAGGAAGTTTGCTTAAA
CCAGGAGGGTTTCTGGTCATCATGGATGCCTTGAAGcgtTCCTACTACATGATTGGGGAACAGAAGTTCTCATCTTTACCTTTGGGCCGTGAAG
CCGTAGAAGCGGCCGTAAAGGAGGCGGGGTACACCATTGAATGGTTCGAGGTTATTTCACAGTCCTggAGCTCCACAATGGCAAATgcCGAGGG
CTTATTTTCGCTGGTTGCTCGCAAACTGAGTCGTCCTCTTCTCGAGCACCACCACCACCACCACTGA

MESGFTSKDTYLSHFNPRDFLEKYYKFGSRHSAESQILKHLLKNLFKIFCLDGVKGDLLIDIGSGPTIYQLLSACESFKEIVVTDYSDQNLQEL
EKWLKKEPEAFDWSPVVTYVCDLEGNRVKGPEKEEKLRQAVKQVLKCDVTQSQPLGAVPLPPADCVLSTLCLHAACPDLPTYCRALRNLGSLLK
PGGFLVIMDALKRSYYMIGEQKFSSLPLGREAVEAAVKEAGYTIEWFEVISQSWSSTMANAEGLFSLVARKLSRPLLEHHHHHH

>v42
ATGGAGAGTGGATTTACGAGTAAGGACACTTATTTGAGTCATTTTAACCCGCGCGACcACTTAGAAAAATACTACAAGTTTGGAAGCCGCCATT
CAGCAGAATCACAAATTCTGAAACACCTTTTAAAGAACCTGTTTAAAATCTTCTGTTTAGATGGGGTCAAAGGAGATTTGTTGATTGACATCGG
ATCAGGTCCCACAATTTACCAACTTCTGTCCGCTTGTGAGAGTTTTAAGGAGATCGTCGTGACGGACTATTCAGATCAAAATCTGCAAGAACTG
GAGAAATGGTTAAAGAAAGAACCAGAGGCGTTCGATTGGAGCCCAGTTGTGACCTATGTCTGTGATCTTGAAGGAAACCGCGTAAAGGGTCCTG
AAAAGGAGGAAAAGCTGCGCCAAGCCGTAAAGCAAGTATTAAAATGTGATGTTACTCAAAGCCAACCATTAGGAGCCGTCCCACTGCCCCCGGC
GGATTGTGTATTGTCAACCCTTTGCTTGcACGCAGCTTGTCCTGACTTGCCTACATATTGTCGTGCATTACGCAACTTAGGAAGTTTGCTTAAA
CCAGGAGGGTTTCTGGTCATCATGGATatgTTGAAGgCgTCCTACTACATGATTGGGGAACAGAAGTTCTCATCTTTACCTTTGGGCCGTGAAG
CCGTAGAAGCGGCCGTAAAGGAGGCGGGGTACACCATTGAATGGTTCGAGGTTATTTCACAGTCCTACAGCTCCACAATGGCAAATtgCGAGGG
CTTATTTTCGCTGGTTGCTCGCAAACTGAGTCGTCCTCTTCTCGAGCACCACCACCACCACCACTGA

MESGFTSKDTYLSHFNPRDHLEKYYKFGSRHSAESQILKHLLKNLFKIFCLDGVKGDLLIDIGSGPTIYQLLSACESFKEIVVTDYSDQNLQEL
EKWLKKEPEAFDWSPVVTYVCDLEGNRVKGPEKEEKLRQAVKQVLKCDVTQSQPLGAVPLPPADCVLSTLCLHAACPDLPTYCRALRNLGSLLK
PGGFLVIMDMLKASYYMIGEQKFSSLPLGREAVEAAVKEAGYTIEWFEVISQSYSSTMANCEGLFSLVARKLSRPLLEHHHHHH

>v43
ATGGAGAGTGGATTTACGAGTAAGGACACTTATTTGAGTCATTTTAACCCGCGCGACTACTTAGAAAAATACTACAAGTTTGGAAGCCGCCATT
CAGCAGAATCACAAATTCTGAAACACCTTTTAAAGAACCTGTTTAAAATCTTCTGTTTAGATGGGGTCAAAGGAGATTTGTTGATTGACATCGG
ATCAGGTCCCACAATTTACCAACTTCTGTCCGCTTGTGAGAGTTTTAAGGAGATCGTCGTGACGGACTATTCAGATCAAAATCTGCAAGAACTG
GAGAAATGGTTAAAGAAAGAACCAGAGGCGTTCGATTGGAGCCCAGTTGTGACCTATGTCTGTGATCTTGAAGGAAACCGCGTAAAGGGTCCTG
AAAAGGAGGAAAAGCTGCGCCAAGCCGTAAAGCAAGTATTAAAATGTGATGTTACTCAAAGCCAACCATTAGGAGCCGTCCCACTGCCCCCGGC
GGATTGTGTATTGTCAACCCTTTGCTTGtgCGCAGCTTGTCCTGACTTGCCTACATATTGTCGTGCATTACGCAACTTAGGAAGTTTGCTTAAA
CCAGGAGGGTTTCTGGTCATCATGGATatgTTGAAGaCcTCCTACTACATGATTGGGGAACAGAAGTTCTCAaCcTTACCTTTGGGCCGTGAAG
CCGTAGAAGCGGCCGTAAAGGAGGCGGGGTACACCATTGAATGGTTCGAGGTTATTTCACAGTCCTACAGCTCCACAATGGCAAATAACGAGGG
CTTATTTTCGCTGGTTGCTCGCAAACTGAGTCGTCCTCTTCTCGAGCACCACCACCACCACCACTGA

MESGFTSKDTYLSHFNPRDYLEKYYKFGSRHSAESQILKHLLKNLFKIFCLDGVKGDLLIDIGSGPTIYQLLSACESFKEIVVTDYSDQNLQEL
EKWLKKEPEAFDWSPVVTYVCDLEGNRVKGPEKEEKLRQAVKQVLKCDVTQSQPLGAVPLPPADCVLSTLCLCAACPDLPTYCRALRNLGSLLK
PGGFLVIMDMLKTSYYMIGEQKFSTLPLGREAVEAAVKEAGYTIEWFEVISQSYSSTMANNEGLFSLVARKLSRPLLEHHHHHH

>v44
ATGGAGAGTGGATTTACGAGTAAGGACACTTATTTGAGTCATTTTAACCCGCGCGACTACTTAGAAAAATACTACAAGTTTGGAAGCCGCCATT
CAGCAGAATCACAAATTCTGAAACACCTTTTAAAGAACCTGTTTAAAATCTTCTGTTTAGATGGGGTCAAAGGAGATTTGTTGATTGACATCGG
ATCAGGTCCCACAATTTACCAACTTCTGTCCGCTTGTGAGAGTTTTAAGGAGATCGTCGTGACGGACTATTCAGATCAAAATCTGCAAGAACTG
GAGAAATGGTTAAAGAAAGAACCAGAGGCGTTCGATTGGAGCCCAGTTGTGACCTATGTCTGTGATCTTGAAGGAAACCGCGTAAAGGGTCCTG
AAAAGGAGGAAAAGCTGCGCCAAGCCGTAAAGCAAGTATTAAAATGTGATGTTACTCAAAGCCAACCATTAGGAGCCGTCCCACTGCCCCCGGC
GGATTGTGTATTGTCAACCCTTTGCTTGcACGCAGCTTGTCCTGACTTGCCTACATATTGTCGTGCATTACGCAACTTAGGAAGTTTGCTTAAA
CCAGGAGGGTTTCTGGTCATCATGGATatgTTGAAGTgcTCCTACTtCATGATTGGGGAACAGAAGTTCTCAcaTTTACCTTTGGGCCGTGAAG
CCGTAGAAGCGGCCGTAAAGGAGGCGGGGTACACCATTGAATGGTTCGAGGTTATTTCACAGTCCTACAGCTCCACAATGGCAAATAACGAGGG
CTTATTTTCGCTGGTTGCTCGCAAACTGAGTCGTCCTCTTCTCGAGCACCACCACCACCACCACTGA

MESGFTSKDTYLSHFNPRDYLEKYYKFGSRHSAESQILKHLLKNLFKIFCLDGVKGDLLIDIGSGPTIYQLLSACESFKEIVVTDYSDQNLQEL
EKWLKKEPEAFDWSPVVTYVCDLEGNRVKGPEKEEKLRQAVKQVLKCDVTQSQPLGAVPLPPADCVLSTLCLHAACPDLPTYCRALRNLGSLLK
PGGFLVIMDMLKCSYFMIGEQKFSHLPLGREAVEAAVKEAGYTIEWFEVISQSYSSTMANNEGLFSLVARKLSRPLLEHHHHHH

>v45
ATGGAGAGTGGATTTACGAGTAAGGACACTTATTTGAGTCATTTTAACCCGCGCGACTtCTTAGAAAAATACTACAAGTTTGGAAGCCGCCATT
CAGCAGAATCACAAATTCTGAAACACCTTTTAAAGAACCTGTTTAAAATCTTCTGTTTAGATGGGGTCAAAGGAGATTTGTTGATTGACATCGG
ATCAGGTCCCACAATTTACCAACTTCTGTCCGCTTGTGAGAGTTTTAAGGAGATCGTCGTGACGGACTATTCAGATCAAAATCTGCAAGAACTG
GAGAAATGGTTAAAGAAAGAACCAGAGGCGTTCGATTGGAGCCCAGTTGTGACCTATGTCTGTGATCTTGAAGGAAACCGCGTAAAGGGTCCTG
AAAAGGAGGAAAAGCTGCGCCAAGCCGTAAAGCAAGTATTAAAATGTGATGTTACTCAAAGCCAACCATTAGGAGCCGTCCCACTGCCCCCGGC
GGATTGTGTATTGTCAACCCTTTGCTTGcACGCAGCTTGTCCTGACTTGCCTACATATTGTCGTGCATTACGCAACTTAGGAAGTTTGCTTAAA
CCAGGAGGGTTTCTGGTCATCATGGATatgTTGAAGTCATCCTACTACATGATTGGGGAACAGAAGTTCTCATCTTTACCTTTGGGCCGTGAAG
CCGTAGAAGCGGCCGTAAAGGAGGCGGGGTACACCATTGAATGGTTCGAGGTTATTTCACAGTCCTtCAGCTCCACAATGGCAAATtgCGAGGG
CTTATTTTCGCTGGTTGCTCGCAAACTGAGTCGTCCTCTTCTCGAGCACCACCACCACCACCACTGA

MESGFTSKDTYLSHFNPRDFLEKYYKFGSRHSAESQILKHLLKNLFKIFCLDGVKGDLLIDIGSGPTIYQLLSACESFKEIVVTDYSDQNLQEL
EKWLKKEPEAFDWSPVVTYVCDLEGNRVKGPEKEEKLRQAVKQVLKCDVTQSQPLGAVPLPPADCVLSTLCLHAACPDLPTYCRALRNLGSLLK
PGGFLVIMDMLKSSYYMIGEQKFSSLPLGREAVEAAVKEAGYTIEWFEVISQSFSSTMANCEGLFSLVARKLSRPLLEHHHHHH

>v46
ATGGAGAGTGGATTTACGAGTAAGGACACTTATTTGAGTCATTTTAACCCGCGCGACTACTTAGAAAAATACTACAAGTTTGGAAGCCGCCATT
CAGCAGAATCACAAATTCTGAAACACCTTTTAAAGAACCTGTTTAAAATCTTCTGTTTAGATGGGGTCAAAGGAGATTTGTTGATTGACATCGG
ATCAGGTCCCACAATTTACCAACTTCTGTCCGCTTGTGAGAGTTTTAAGGAGATCGTCGTGACGGACTATTCAGATCAAAATCTGCAAGAACTG
GAGAAATGGTTAAAGAAAGAACCAGAGGCGTTCGATTGGAGCCCAGTTGTGACCTATGTCTGTGATCTTGAAGGAAACCGCGTAAAGGGTCCTG
AAAAGGAGGAAAAGCTGCGCCAAGCCGTAAAGCAAGTATTAAAATGTGATGTTACTCAAAGCCAACCATTAGGAGCCGTCCCACTGCCCCCGGC
GGATTGTGTATTGTCAACCCTTTGCTTGGACGCAGCTTGTCCTGACTTGCCTACATATTGTCGTGCATTACGCAACTTAGGAAGTTTGCTTAAA
CCAGGAGGGTTTCTGGTCATCATGGgTatgTTGAAGTCATCCTACTACATGATTGGGGAACAGAAGTTCTCAgCgTTACCTTTGGGCCGTGAAG
CCGTAGAAGCGGCCGTAAAGGAGGCGGGGTACACCATTGAATGGTTCGAGGTTATTTCACAGTCCTtCAGCTCCACAATGtgcAATAACGAGGG
CTTATTTTCGCTGGTTGCTCGCAAACTGAGTCGTCCTCTTCTCGAGCACCACCACCACCACCACTGA

MESGFTSKDTYLSHFNPRDYLEKYYKFGSRHSAESQILKHLLKNLFKIFCLDGVKGDLLIDIGSGPTIYQLLSACESFKEIVVTDYSDQNLQEL
EKWLKKEPEAFDWSPVVTYVCDLEGNRVKGPEKEEKLRQAVKQVLKCDVTQSQPLGAVPLPPADCVLSTLCLDAACPDLPTYCRALRNLGSLLK
PGGFLVIMGMLKSSYYMIGEQKFSALPLGREAVEAAVKEAGYTIEWFEVISQSFSSTMCNNEGLFSLVARKLSRPLLEHHHHHH

>v47
ATGGAGAGTGGATTTACGAGTAAGGACACTTATTTGAGTCATTTTAACCCGCGCGACTACTTAGAAAAATACTACAAGTTTGGAAGCCGCCATT
CAGCAGAATCACAAATTCTGAAACACCTTTTAAAGAACCTGTTTAAAATCTTCTGTTTAGATGGGGTCAAAGGAGATTTGTTGATTGACATCGG
ATCAGGTCCCACAATTTACCAACTTCTGTCCGCTTGTGAGAGTTTTAAGGAGATCGTCGTGACGGACTATTCAGATCAAAATCTGCAAGAACTG
GAGAAATGGTTAAAGAAAGAACCAGAGGCGTTCGATTGGAGCCCAGTTGTGACCTATGTCTGTGATCTTGAAGGAAACCGCGTAAAGGGTCCTG
AAAAGGAGGAAAAGCTGCGCCAAGCCGTAAAGCAAGTATTAAAATGTGATGTTACTCAAAGCCAACCATTAGGAGCCGTCCCACTGCCCCCGGC
GGATTGTGTATTGTCAACCCTTTGCTTGGAaGCAGCTTGTCCTGACTTGCCTACATATTGTCGTGCATTACGCAACTTAGGAAGTTTGCTTAAA
CCAGGAGGGTTTCTGGTCATCATGGgTGCCTTGAAGaacTCCTACTACATGATTGGGGAACAGAAGTTCTCAgCgTTACCTTTGGGCCGTGAAG
CCGTAGAAGCGGCCGTAAAGGAGGCGGGGTACACCATTGAATGGTTCGAGGTTATTTCACAGTCCTtCAGCTCCACAATGGCAAATAACGAGGG
CTTATTTTCGCTGGTTGCTCGCAAACTGAGTCGTCCTCTTCTCGAGCACCACCACCACCACCACTGA

MESGFTSKDTYLSHFNPRDYLEKYYKFGSRHSAESQILKHLLKNLFKIFCLDGVKGDLLIDIGSGPTIYQLLSACESFKEIVVTDYSDQNLQEL
EKWLKKEPEAFDWSPVVTYVCDLEGNRVKGPEKEEKLRQAVKQVLKCDVTQSQPLGAVPLPPADCVLSTLCLEAACPDLPTYCRALRNLGSLLK
PGGFLVIMGALKNSYYMIGEQKFSALPLGREAVEAAVKEAGYTIEWFEVISQSFSSTMANNEGLFSLVARKLSRPLLEHHHHHH

>v48
ATGGAGAGTGGATTTACGAGTAAGGACACTTATTTGAGTCATTTTAACCCGCGCGACTtCTTAGAAAAATACTACAAGTTTGGAAGCCGCCATT
CAGCAGAATCACAAATTCTGAAACACCTTTTAAAGAACCTGTTTAAAATCTTCTGTTTAGATGGGGTCAAAGGAGATTTGTTGATTGACATCGG
ATCAGGTCCCACAATTTACCAACTTCTGTCCGCTTGTGAGAGTTTTAAGGAGATCGTCGTGACGGACTATTCAGATCAAAATCTGCAAGAACTG
GAGAAATGGTTAAAGAAAGAACCAGAGGCGTTCGATTGGAGCCCAGTTGTGACCTATGTCTGTGATCTTGAAGGAAACCGCGTAAAGGGTCCTG
AAAAGGAGGAAAAGCTGCGCCAAGCCGTAAAGCAAGTATTAAAATGTGATGTTACTCAAAGCCAACCATTAGGAGCCGTCCCACTGCCCCCGGC
GGATTGTGTATTGTCAACCCTTTGCTTGtgCGCAGCTTGTCCTGACTTGCCTACATATTGTCGTGCATTACGCAACTTAGGAAGTTTGCTTAAA
CCAGGAGGGTTTCTGGTCATCATGGATatgTTGAAGTgcTCCTACTACATGATTGGGGAACAGAAGTTCTCATCTTTACCTTTGGGCCGTGAAG
CCGTAGAAGCGGCCGTAAAGGAGGCGGGGTACACCATTGAATGGTTCGAGGTTATTTCACAGTCCTACAGCTCCACAATGGCAAATtgCGAGGG
CTTATTTTCGCTGGTTGCTCGCAAACTGAGTCGTCCTCTTCTCGAGCACCACCACCACCACCACTGA

MESGFTSKDTYLSHFNPRDFLEKYYKFGSRHSAESQILKHLLKNLFKIFCLDGVKGDLLIDIGSGPTIYQLLSACESFKEIVVTDYSDQNLQEL
EKWLKKEPEAFDWSPVVTYVCDLEGNRVKGPEKEEKLRQAVKQVLKCDVTQSQPLGAVPLPPADCVLSTLCLCAACPDLPTYCRALRNLGSLLK
PGGFLVIMDMLKCSYYMIGEQKFSSLPLGREAVEAAVKEAGYTIEWFEVISQSYSSTMANCEGLFSLVARKLSRPLLEHHHHHH

>v49
ATGGAGAGTGGATTTACGAGTAAGGACACTTATTTGAGTCATTTTAACCCGCGCGACTACTTAGAAAAATACTACAAGTTTGGAAGCCGCCATT
CAGCAGAATCACAAATTCTGAAACACCTTTTAAAGAACCTGTTTAAAATCTTCTGTTTAGATGGGGTCAAAGGAGATTTGTTGATTGACATCGG
ATCAGGTCCCACAATTTACCAACTTCTGTCCGCTTGTGAGAGTTTTAAGGAGATCGTCGTGACGGACTATTCAGATCAAAATCTGCAAGAACTG
GAGAAATGGTTAAAGAAAGAACCAGAGGCGTTCGATTGGAGCCCAGTTGTGACCTATGTCTGTGATCTTGAAGGAAACCGCGTAAAGGGTCCTG
AAAAGGAGGAAAAGCTGCGCCAAGCCGTAAAGCAAGTATTAAAATGTGATGTTACTCAAAGCCAACCATTAGGAGCCGTCCCACTGCCCCCGGC
GGATTGTGTATTGTCAACCCTTTGCTTGtgCGCAGCTTGTCCTGACTTGCCTACATATTGTCGTGCATTACGCAACTTAGGAAGTTTGCTTAAA
CCAGGAGGGTTTCTGGTCATCATGGATGCCTTGAAGTgcTCCTACTACATGATTGGGGAACAGAAGTTCTCAcaTTTACCTTTGGGCCGTGAAG
CCGTAGAAGCGGCCGTAAAGGAGGCGGGGTACACCATTGAATGGTTCGAGGTTATTTCACAGTCCTggAGCTCCACAATGGCAAATgcCGAGGG
CTTATTTTCGCTGGTTGCTCGCAAACTGAGTCGTCCTCTTCTCGAGCACCACCACCACCACCACTGA

MESGFTSKDTYLSHFNPRDYLEKYYKFGSRHSAESQILKHLLKNLFKIFCLDGVKGDLLIDIGSGPTIYQLLSACESFKEIVVTDYSDQNLQEL
EKWLKKEPEAFDWSPVVTYVCDLEGNRVKGPEKEEKLRQAVKQVLKCDVTQSQPLGAVPLPPADCVLSTLCLCAACPDLPTYCRALRNLGSLLK
PGGFLVIMDALKCSYYMIGEQKFSHLPLGREAVEAAVKEAGYTIEWFEVISQSWSSTMANAEGLFSLVARKLSRPLLEHHHHHH

>v50
ATGGAGAGTGGATTTACGAGTAAGGACACTTATTTGAGTCATTTTAACCCGCGCGACcACTTAGAAAAATtCTACAAGTTTGGAAGCCGCCATT
CAGCAGAATCACAAATTCTGAAACACCTTTTAAAGAACCTGTTTAAAATCTTCTGTTTAGATGGGGTCAAAGGAGATTTGTTGATTGACATCGG
ATCAGGTCCCACAATTTACCAACTTCTGTCCGCTTGTGAGAGTTTTAAGGAGATCGTCGTGACGGACTATTCAGATCAAAATCTGCAAGAACTG
GAGAAATGGTTAAAGAAAGAACCAGAGGCGTTCGATTGGAGCCCAGTTGTGACCTATGTCTGTGATCTTGAAGGAAACCGCGTAAAGGGTCCTG
AAAAGGAGGAAAAGCTGCGCCAAGCCGTAAAGCAAGTATTAAAATGTGATGTTACTCAAAGCCAACCATTAGGAGCCGTCCCACTGCCCCCGGC
GGATTGTGTATTGTCAACCCTTTGCTTGcACGCAGCTTGTCCTGACTTGCCTACATATTGTCGTGCATTACGCAACTTAGGAAGTTTGCTTAAA
CCAGGAGGGTTTCTGGTCATCATGGATGCCTTGAAGcgtTCCTACTACATGATTGGGGAACAGAAGTTCTCATCTTTACCTTTGGGCCGTGAAG
CCGTAGAAGCGGCCGTAAAGGAGGCGGGGTACACCATTGAATGGTTCGAGGTTATTTCACAGTCCTACAGCTCCACAATGGCAAATAACGAGGG
CTTATTTTCGCTGGTTGCTCGCAAACTGAGTCGTCCTCTTCTCGAGCACCACCACCACCACCACTGA

MESGFTSKDTYLSHFNPRDHLEKFYKFGSRHSAESQILKHLLKNLFKIFCLDGVKGDLLIDIGSGPTIYQLLSACESFKEIVVTDYSDQNLQEL
EKWLKKEPEAFDWSPVVTYVCDLEGNRVKGPEKEEKLRQAVKQVLKCDVTQSQPLGAVPLPPADCVLSTLCLHAACPDLPTYCRALRNLGSLLK
PGGFLVIMDALKRSYYMIGEQKFSSLPLGREAVEAAVKEAGYTIEWFEVISQSYSSTMANNEGLFSLVARKLSRPLLEHHHHHH

>cthHMT
ATGCTTGGTATGGACGCTGATACGGCAAGTTTTTGGGAGGAGAAATATCGTGCAGATTTGACTGCCTGGGACCGTGGCGGCGTATCACCGGCTT
TGGAACATTGGCTTGCAGAAGGCGCACTGAAGCCCGGCCGTATCTTAATTCCAGGTTGCGGATACGGGCATGAGGTCCTTGCCTTGGCACGCCG
TGGATTCGAAGTTTGGGGCTTGGACATCGCCCTTACTCCCGTTCGCCGTTTGCAAGAGAAACTGGCTCAGGCAGGTTTAACAGCCCACGTAGTC
GAAGGAGACGTTCGCACCTGGCAGCCGGAACAACCTTTTGATGCGGTGTATGAACAAACTTGTTTGTGCGCTTTGAGTCCAGAAGACTGGCCTC
GTTATGAGGCACAATTATGCCGTTGGTTACGTCCCGGCGGTCGTCTTTTCGCGCTGTGGATGCAGACCGACCGTCCTGGAGGACCACCATATCA
TTGTGGACTTGAAGCCATGCGCGTACTGTTTGCTTTAGAACGTTGGCGTTGGGTCGAACCCCCACAACGCACTGTACCCCATCCTACTGGTTTT
TTTGAGTATGCTGCCATTTTAGAGCGTCTTGTTCTGGAACATCATCACCACCATCATTAA

MLGMDADTASFWEEKYRADLTAWDRGGVSPALEHWLAEGALKPGRILIPGCGYGHEVLALARRGFEVWGLDIALTPVRRLQEKLAQAGLTAHVV
EGDVRTWQPEQPFDAVYEQTCLCALSPEDWPRYEAQLCRWLRPGGRLFALWMQTDRPGGPPYHCGLEAMRVLFALERWRWVEPPQRTVPHPTGF
FEYAAILERLVLEHHHHHH

>athHMT
ATGGCGGAGGAGCAACAAAATTCCGATCAGTCCAATGGTGGTAATGTGATCCCAACCCCCGAGGAAGTAGCAACCTTCTTGCACAAGACAGTCG
AGGAGGGCGGCTGGGAGAAGTGCTGGGAGGAGGAAATTACACCATGGGACCAGGGTCGCGCCACGCCGTTAATCGTGCATTTAGTGGATACATC
AAGTCTTCCCCTTGGCCGCGCATTGGTGCCAGGGTGTGGTGGCGGACACGATGTCGTAGCCATGGCCTCGCCAGAGCGTTTCGTTGTCGGCCTG
GATATCTCGGAAAGTGCGCTTGCGAAGGCAAATGAAACATATGGCAGCTCACCTAAAGCTGAATATTTCTCGTTTGTCAAAGAGGATGTCTTTA
CCTGGCGTCCCACCGAATTGTTTGACTTGATTTTTGACTATGTTTTTTTTGTGCAATTGAGCCGGAAATGCGTCCTGCGTGGGCTAAGTCCAT
GTATGAGCTTCTGAAACCAGACGGCGAGTTAATCACCCTGATGTACCCCATCACCGATCATGTAGGTGGCCCCCCATACAAGGTAGATGTTAGC
ACATTCGAGGAAGTGTTAGTGCCTATCGGGTTCAAAGCAGTGAGCGTTGAGGAGAACCCTCACGCAATCCCCACACGCAAGGGAAAAGAGAAAC
TTGGTCGCTGGAAAAAAATCAATCTGGAACATCATCACCACCATCATTAA

MAEEQQNSDQSNGGNVIPTPEEVATFLHKTVEEGGWEKCWEEEITPWDQGRATPLIVHLVDTSSLPLGRALVPGCGGGHDVVAMASPERFVVGL
DISESALAKANETYGSSPKAEYFSFVKEDVFTWRPTELFDLIFDYVFFCAIEPEMRPAWAKSMYELLKPDGELITLMYPITDHVGGPPYKVDVS
TFEEVLVPIGFKAVSVEENPHAIPTRKGKEKLGRWKKINLEHHHHHH

>aclHMT
ATGTCAACGCCCAGTTTAATTCCATCCGGTGTGCACGAAGTATTAGCAAAATACAAAGACGGTAACTATGTCGACGGGTGGGCCGAACTGTGGG
ATAAATCTAAGGGTGACCGTCTTCCATGGGATCGTGGTTTTCCGAACCCTGCGTTGGAAGATACCCTGATTCAGAAACGTGCCATCATCGGAGG
TCCCTTGGGCCAAGACGCTCAGGGCAAGACATACCGCAAAAAAGCTCTGGTCCCAGGCTGCGGTCGTGGAGTAGATGTGTTGTTATTGGCATCG
TTCGGTTATGACGCTTATGGTTTAGAATACTCTGCAACGGCGGTCGATGTATGCCAAGAGGAGCAAGCCAAGAACGGGGATCAATATCCTGTAC
GCGATGCAGAAATCGGCCAGGGCAAGATTACGTTCGTTCAAGGTGATTTCTTTGAGGACACGTGGCTTGAAAAGCTTAACCTTACCCGCAATTG
TTTCGACGTTATCTACGACTACACGTTCTTCTGTGCATTGAACCCGAGCATGCGCCCTCAGTGGGCTTTGCGCCATACCCAACTTCTTGCCGAC
TCTCCGCGCGGACATTTAATCTGTTTGGAGTTTCCCCGCCATAAAGACCCTTCTGTACAGGGGCCACCATGGGGCTCGGCGTCAGAAGCCTACC
GCGCCCATCTGTCACATCCAGGGGAGGAAATTCCCTACGATGCGTCACGTCAATGCCAGTTTGACAGCAGCAAAGCACCCAGTGCGCAGGGCTT
GGAACGTGTAGCATATTGGCAACCAGAGCGCACGCATGAAGTAGGGAAAAACGAGAAAGGTGAGGTTCAAGATCGCGTATCCATCTGGCAGCGT
CCTCCCCAAAGTTCCCTGCTGGAACATCATCACCACCATCATTAA

MSTPSLIPSGVHEVLAKYKDGNYVDGWAELWDKSKGDRLPWDRGFPNPALEDTLIQKRAIIGGPLGQDAQGKTYRKKALVPGCGRGVDVLLLAS
FGYDAYGLEYSATAVDVCQEEQAKNGDQYPVRDAEIGQGKITFVQGDFFEDTWLEKLNLTRNCFDVIYDYTFFCALNPSMRPQWALRHTQLLAD
SPRGHLICLEFPRHKDPSVQGPPWGSASEAYRAHLSHPGEEIPYDASRQCQFDSSKAPSAQGLERVAYWQPERTHEVGKNEKGEVQDRVSIWQR
PPQSSLLEHHHHHH

>bmaHMT
ATGTCTACTGTAGCTAATATCGCTCCCGTATTCACGGGCGACTGCAAAACTATCCCAACGCCCGAAGAGTGCGCTACCTTCTTATACAAGGTCG
TCAATTCCGGTGGGTGGGAGAAGTGTTGGGTGGAGGAAGTGATCCCGTGGGATCTTGGAGTGCCTACTCCGTTAGTATTACATCTTGTAAAGAA
TAACGCCCTGCCAAACGGTAAAGGTCTTGTACCAGGATGTGGCGGGGGCTATGATGTAGTGGCGATGGCAAACCCGGAGCGTTTCATGGTCGGG
CTGGATATTAGTGAGAATGCTCTTAAGAAGGCCCGTGAAACTTTCTCGACCATGCCTAATAGTAGCTGCTTCAGCTTCGTCAAGGAGGATGTCT
TTACCTGGCGTCCAGAGCAACCGTTTGATTTTATTTTCGACTACGTTTTCTTTTGCGCTATCGACCCCAAAATGCGTCCGGCATGGGGTAAAGC
CATGTATGAATTACTGAAGCCTGACGGAGAGTTAATCACGTTGATGTATCCAATTACAAACCATGAGGGAGGACCTCCATTCTCTGTCAGCGAA
TCCGAGTACGAAAAGGTCCTGGTGCCCTTGGGGTTTAAACAGCTGTCTTTAGAAGACTACAGTGATCTTGCCGTCGAACCTCGTAAGGGCAAGG
AGAAGTTGGCCCGTTGGAAGAAAATGAACAACCTGGAACATCATCACCACCATCATTAA

MSTVANIAPVFTGDCKTIPTPEECATFLYKVVNSGGWEKCWVEEVIPWDLGVPTPLVLHLVKNNALPNGKGLVPGCGGGYDVVAMANPERFMVG
LDISENALKKARETFSTMPNSSCFSFVKEDVFTWRPEQPFDFIFDYVFFCAIDPKMRPAWGKAMYELLKPDGELITLMYPITNHEGGPPFSVSE
SEYEKVLVPLGFKQLSLEDYSDLAVEPRKGKEKLARWKKMNNLEHHHHHH

>bxeHMT
ATGTCCGACCCGACCCAACCTGCCGTTCCAGATTTCGAAACCCGTGATCCCAATTCCCCTGCCTTCTGGGATGAGCGTTTCGAGCGTCGCTTTA
CACCTTGGGACCAGGCCGGAGTTCCTGCGGCTTTTCAGTCTTTTGCAGCGCGCCATTCAGGCGCTGCTGTATTGATCCCTGGTTGTGGTTCTGC
CTATGAGGCCGTGTGGTTAGCCGGGCAGGGAAACCCTGTTCGCGCTATCGATTTTTCACCTGCTGCTGTAGCGGCAGCACATGAACAGTTAGGG
GCGCAGCACGCGCAGTTAGTGGAGCAAGCTGATTTTTTCACGTATGAACCGCCGTTTACACCTGCTTGGATTTATGAGCGCGCATTTCTGTGTG
CATTGCCCTTAGCGCGTCGCGCCGACTATGCCCACCGCATGGCAGATCTTCTGCCGGGCGGTGCTTTATTAGCCGGCTTTTTCTTCCTTGGTGC
AACTCCCAAAGGACCACCGTTTGGAATCGAGCGTGCAGAGCTGGACGCATTGTTAACTCCATACTTCGACCTTATTGAAGACGAGGCAGTCCAC
GACTCCATCGCGGTTTTTGCTGGACGTGAGCGCTGGTTGACATGGCGCCGCCGCGCCCTGGAACATCATCACCACCATCATTAA

MSDPTQPAVPDFETRDPNSPAFWDERFERRFTPWDQAGVPAAFQSFAARHSGAAVLIPGCGSAYEAVWLAGQGNPVRAIDFSPAAVAAAHEQLG
AQHAQLVEQADFFTYEPPFTPAWIYERAFLCALPLARRADYAHRMADLLPGGALLAGFFFLGATPKGPPFGIERAELDALLTPYFDLIEDEAVH
DSIAVFAGRERWLTWRRRALEHHHHHH

>selHMT
ATGACTAATGCCGTTAACCAAGCTCAATTTTGGGAGCAGCGTTATCAAGAAGGGTCCGATCGTTGGGACCTTGGGCAGGCGGCGCCTGTCTGGC
GTTCACTTTTGGCGGGGACTAATGCCCCGGCACCTGGTCGCATTGCAGTATTAGGCTGTGGACGCGGTCACGATGCTCGCTTATTTGCCGAGCA
AGGCTTCGAGGTTGTCGGCTTTGACTTTGCGCCATCCGCCATTGCCGCAGCACAAGCGTTGGCCCAGGGAACTACAGCGCAGTTTCTTCAGCGT
GATATTTTTGCGTTGCCTCAAGAATTCGCGGGTCAGTTCGACACTGTTCTGGAGCATACATGCTTTTGCGCCATTGATCCTGACCGCCGTGCCG
AGTATGTTGAGGTTGTGCGCCAAATTTTGAAACCTAAGGGGTGCCTTCTTGGCTTGTTTTGGTGTCATGATCGTCCGTCCGGCCCTCCCTATGG
ATGTTCTTTAACAGAACTGCGCGATCGTTTCGCTCAGGGCTGGCAAGAAGAGCAGTTAGAATCTGTCACAGAATCCGTGGAGGGTCGCCGTGGT
GAGGAATACTTGGGACGTTGGCGTCGTCTGGATCTGGAACATCATCACCACCATCATTAA

MTNAVNQAQFWEQRYQEGSDRWDLGQAAPVWRSLLAGTNAPAPGRIAVLGCGRGHDARLFAEQGFEVVGFDFAPSAIAAAQALAQGTTAQFLQR
DIFALPQEFAGQFDTVLEHTCFCAIDPDRRAEYVEVVRQILKPKGCLLGLFWCHDRPSGPPYGCSLTELRDRFAQGWQEEQLESVTESVEGRRG
EEYLGRWRRLDLEHHHHHH

X. References

[1] D. G. Gibson, L. Young, R.-Y. Chuang, J. C. Venter, C. A. Hutchison, H. O. Smith, *Nat. Methods* **2009**, *6*, 343–345.

[2] A. Aiyar, Y. Xiang, J. Leis, *Methods Mol. Biol.* **1996**, *57*, 177–191.

[3] T. Baba, T. Ara, M. Hasegawa, Y. Takai, Y. Okumura, M. Baba, K. A. Datsenko, M. Tomita, B. L. Wanner, H. Mori, *Mol. Syst. Biol.* **2006**, *2*, 2006.0008.

[4] O. Khersonsky, R. Lipsh, Z. Avizemer, Y. Ashani, M. Goldsmith, H. Leader, O. Dym, S. Rogotner, D. L. Trudeau, J. Prilusky, P. Amengual-Rigo, V. Guallar, D. S. Tawfik, S. J. Fleishman, *Mol. Cell.* **2018**, *72*, 178–186.

[5] N. J. P. Broom, J. S. Elder, P. C. T. Hannan, J. E. Pons, P. J. O'Hanlon, G. Walker, J. Wilson, P. Woodall, *J. Antibiot. (Tokyo).* **1995**, *48*, 1336–1344.

[6] O. Ovadia, S. Greenberg, J. Chatterjee, B. Laufer, F. Opperer, H. Kessler, C. Gilon, A. Hoffman, *Mol. Pharm.* **2011**, *8*, 479–487.

[7] A. Gutmann, C. Krump, L. Bungaruang, B. Nidetzky, *Chem. Commun.* **2014**, *50*, 5465–5468.

[8] L. A. Wessjohann, J. Keim, B. Weigel, M. Dippe, *Curr. Opin. Chem. Biol.* **2013**, *17*, 229–235.

[9] M. D. Shepherd, T. Liu, C. Méndez, J. A. Salas, J. Rohr, *Appl. Environ. Microbiol.* **2011**, *77*, 435–441.

[10] L. a Wessjohann, B. Sontag, *Angew. Chem. Int. Ed.* **1996**, *35*, 1697–1699.

[11] J. D. Rudolf, C. D. Poulter, *ACS Chem. Biol.* **2013**, *8*, 2707–2714.

[12] C. Dürr, D. Hoffmeister, S. E. Wohlert, K. Ichinose, M. Weber, U. Von Mulert, J. S. Thorson, A. Bechthold, *Angew. Chem. Int. Ed.* **2004**, *43*, 2962–2965.

[13] M. Mittler, A. Bechthold, G. E. Schulz, *J. Mol. Biol.* **2007**, *372*, 67–76.

[14] T. D. Huber, B. R. Johnson, J. Zhang, J. S. Thorson, *Curr. Opin. Biotechnol.* **2016**, *42*, 189–197.

[15] M. Thomsen, S. B. Vogensen, J. Buchardt, M. D. Burkart, R. P. Clausen, *Org. Biomol. Chem.* **2013**, *11*, 7606–7610.

[16] S. Mordhorst, J. Siegrist, M. Müller, M. Richter, J. N. Andexer, *Angew. Chem. Int. Ed.* **2017**, *56*, 4037–4041.

[17] A. J. Herbert, S. A. Shepherd, V. A. Cronin, M. R. Bennett, R. Sung, J. Micklefield, *Angew. Chem. Int. Ed.* **2020**, anie.202004963.

[18] C. Liao, F. P. Seebeck, *Nat. Catal.* **2019**, *2*, 696-702.

[19] H. S. Loring, P. R. Thompson, *Biochem.* **2018**, 57, 5524-5532.

[20] S. P. Ivonin, B. B. Kurpil', E. B. Rusanov, O. O. Grygorenko, D. M. Volochnyuk, *Tetrahedron Lett.* **2014**, *55*, 2187–2189.

[21] W. Li, D. Yuan, G. Wang, Y. Zhao, J. Xie, S. Li, C. Zhu, *J. Am. Chem. Soc.* **2019**, *141*, 3187–3197.
